# Unbounded and revocable hierarchical identity-based encryption with adaptive security, decryption key exposure resistant, and short public parameters

**DOI:** 10.1371/journal.pone.0195204

**Published:** 2018-04-12

**Authors:** Qianqian Xing, Baosheng Wang, Xiaofeng Wang, Jing Tao

**Affiliations:** College of Computer, National University of Defense Technology, Changsha, Hunan, China; King Saud University, SAUDI ARABIA

## Abstract

Revocation functionality and hierarchy key delegation are two necessary and crucial requirements to identity-based cryptosystems. Revocable hierarchical identity-based encryption (RHIBE) has attracted a lot of attention in recent years, many RHIBE schemes have been proposed but shown to be either insecure or bounded where they have to fix the maximum hierarchical depth of RHIBE at setup. In this paper, we propose a new unbounded RHIBE scheme with decryption key exposure resilience and with short public system parameters, and prove our RHIBE scheme to be adaptively secure. Our system model is scalable inherently to accommodate more levels of user adaptively with no adding workload or restarting the system. By carefully designing the hybrid games, we overcome the subtle obstacle in applying the dual system encryption methodology for the unbounded and revocable HIBE. To the best of our knowledge, this is the first construction of adaptively secure unbounded RHIBE scheme.

## 1 Introduction

Revocation functionality is indispensable to (H)IBE since there are threats of leaking a secret key by hacking or legal situation of expiration of contract for using system. In those seminal works [1] [2], it has also been pointed out that providing an efficient key hierarchy delegation mechanism for IBE is essential. To satisfing both hierarchical key delegation and user revocation, revocable hierarchical identity-based encryption (RHIBE) has been paid attention. Unfortunately most of existing RHIBEs proposed [1] [3] [4] [5] [6] [7] are either insecure or bounded where they have to fix the maximum hierarchical depth of RHIBE at setup. Bounded (R)HIBE schemes restrict the maximum hierarchy of (R)HIBE, i.e., they need to declare the max level in the public parameters at setup phase. It is highly impossible to set the maximum hierarchy properly in practice: too small to accommodate enough users or too large that wastes identity space needlessly and increase keys computation unnecessarily.

In contrast, the unbounded RHIBE is more scalable to achieve efficient and dynamic user management. Ryu proposed an unbounded RHIBE scheme [7] inspired by an universe KP-ABE [8]. But it only achieves selective-ID security. In selective-ID security notion, the reduction algorithm requires the challenge identity before the setup phase in the proof [1, 3]. That means the adversary holds no information before giving the challenge ID, but the simulator can exploit the challenge information submitted by the adversary to construct the trick public parameters and other keys in games. That is a weaker security notion.

Adaptive-ID security represents full security notion that an adversary gives the challenge identify when he has learnt the public information. Lee [5] considered the adaptively secure RHIBE but his scheme don’t support the property of unbounded hierarchical key delegation. Xing [9] claimed to achive the first adaptively secure and unbounded RHIBE, but its security proof that uses the dual system encryption technique has some flaws. Therefore, the construction of an adaptively secure unbounded RHIBE scheme is still an unsolved open problem.

### 1.1 Our techniques

The dual system encryption framework [10] is usually for proving the adaptive security of HIBEs in composite-order bilinear groups. To achieve the adaptive security in the framework, the notion of semi-functionality is introduced [10] [11] and the proof strategy is that a normal challenge ciphertext is changed to be semi-functional, and then each normal private key is changed to be semi-functional one by one through hybrid games.

There is a paradox that need to be overcome. Since a normal ciphertext can be decrypted by a semi-functional private key but a semi-functional ciphertext cannot be decrypted by a semi-functional private key, a simulator can check whether a private key is normal or semi-functional by decrypting a semi-functional ciphertext(note that a simulator can generate a ciphertext and a private key for any identity). To overcome the obstacle, the nominally semi-functional type of private keys is introduced: the challenge semi-functional private key is constructed as a nominally semi-functional private key so that the semi-functional ciphertext of the same identity the simulator generates always can be decrypted by it. In addition, a detailed information theoretic argument should be given to argue that a nominally semi-functional key is indistinguishable from a semi-functional key.

Although the dual system encryption is maturing to exploit in normal HIBEs to achieve the adaptive security, it is more complex when dealing with revocable HIBE schemes. In HIBE, the essential restriction for the information theoretic argument is that an adversary cannot query a private key for *ID* that is a prefix of the challenge identity ID*. However, the restriction do not exist in RHIBEs. The private key of any prefix of ID* and the update key for the challenge time T* are both allowed to query for the adversary in RHIBEs. Recall that the simulator of an HIBE scheme can change the normal-private key to a semi-functional private key by using a nominally semi-functional key and the constraint *ID* ∉ *Prefix*(ID*) of the security model. The nominally semi-functional key is indistinguishable from a semi-functional key by an information theoretic argument using the constraint *ID* ∉ *Prefix*(ID*). However, in the case of (U-)RHIBE, a simple method cannot change the normal-private key to the semi-functional private key since the adversary can query and achieve the private key for any *ID* ∈ *Prefix*(ID*).

Moreover, an unbounded RHIBE scheme has so low entropy context that it is hard to execute an information-theoretic argument, which is different with those bounded RHIBE schemes. So the dual system encryption method in Lee-RHIBE [5] does not work. Although Lewko and Waters [12] has proposed a nested dual system encryption approach to allow a sufficient information-theoretic argument in a very localized context for unbounded HIBEs, the trival applying to a revocable extention scheme is inappropriate to hold the paradox information theoretic argument. Unfortunately Xing and Wang [9] have neglected this important change, so that the proof of their unbounded RHIBE scheme is non-rigorous with flaws. Obviously the attacker can distinguish between the oracles they design for the game hoppings in [9], which is not as they claimed in Lemma 4.

To circumvent the subtle obstacle and apply the dual system encryption methodology for our adaptively secure unbounded RHIBE with decryption key exposure resistance, our strategy is threehold:

(1) We use a modular design strategy like [13] and construct the private keys and update keys from smaller component keys. A private key consists of many HIBE private keys that are related to a path in a binary tree and an update key also consists of many IBE private keys that are related to a cover set in a binary tree. The HIBE and IBE private keys can be grouped together if they are related to the same node in a binary tree. So we change to deal with the transformation of component HIBE and IBE keys in the hybrid games instead of directly with the private keys and update keys of RHIBE which cannot be simply changed from normal keys to semi-functional keys.

(2) We design a nested dual system encryption for revocable and hierarchical IBE schemes with the concept of ephemeral semi-functionality for secret keys, update keys, decryption keys and ciphertexts. To demonstrate a hybrid process of games to chellenge keys and ciphertexts, we define several oracles to simulate the different forms of the component HIBE and IBE keys which construct the semi-functional or ephemeral semi-functional secret keys, update keys and decryption keys.

(3) For showing an information theoretic argument under RHIBE model successfully, we firstly classify the behavior of an adversary as two types under the restriction of the RHIBE security model. The Type-1 adversary is restricted to queries on the secret keys of any hierarchical identity satistying ${ID|}_{k}\notin Prefix\left(ID_{l}^{*}\right)$, so we carefully re-design a sequence of hybrid games to show several times of information theoretic arguments successfully for the secret keys and avoid a potential paradox for the update keys. The Type-2 adversary is restricted to queries on the update keys on the time *T* ∉ T*, so we carefully re-design the other sequence of hybrid games to show several times of information theoretic argument successfully for the update keys and avoid a potential paradox for the secret keys.

### 1.2 Our result

We propose the first adaptively secure unbounded RHIBE in composite-order bilinear groups under simple static assumptions. It removes the limitation of the maximum hierarchical depth in the encryption system and accommodate more levels of user adaptively without adding workload or restarting the system. Our RHIBE scheme also supports decryption key exposure resistance by the key-randomization method which meets the strong security notion for R(H)IBE [14].

Compared to existing RHIBE schemes, it is the first RHIBE to achieve simultaneously adaptive-ID security, decryption key exposure resistance and unbounded key delegation, as shown in Table 1. In Table 2, we discuss the comparison about the efficiency of key space and decryption computation, noted that *l* is the maximum level of the hierarchy, *h* is the level of a user in the hierarchy, *N* is the number of maximum users in each level, *r* is the number of revoked users, *t*_*e*_ is the cost for performing a bilinear pairing, |*G*| and |*G*_*T*_| are the sizes of one element in *G* and *G*_*T*_ respectively. Our RHIBE scheme has the short and constant public parameter which is independent with the maximum level of the system hierarchy. Moreover, our RHIBE reduces the size of the update key from *O*(*hr*log(*N*/*r*))to *O*(*h* + *r*log(*N*/*r*)).

**Table 1:** Comparisons among RHIBE schemes.

Game	Unbounded delegation	Adaptive-ID security	DKE resist.	Assumption
Seo(2013) [1]	×	×	×	DBDH
Seo(2015) [3]	×	×	√	*q*-Type
Seo(2015) [6]	×	*	√	static
Ryu(2015) [7]	√	×	√	*q*-RW2
Lee(2016) [13]	×	×	√	*q*-Type
Lee(2016) [5]	×	√	√	static
Xing(2016) [9]	√	*	√	static
Our RHIBE	√	√	√	static

Note: Security marked with * has flaws in its proof.

**Table 2:** Comparisons among RHIBE schemes.cont.

Game	Dec. cost	CT Size	PP size	SK size	UK Size
Seo(2013) [1]	(*h* + 2)*t*_*e*_	*O*(*l*)	(*l* + 4)|*G*|	*O*(*l*^2^log*N*)	*O*(*r*log(*N*/*r*))
Seo(2015) [3]	3*t*_*e*_	*O*(1)	(*l* + 6)|*G*|	*O*(*l*log*N*)	*O*(*lr*log(*N*/*r*))
Seo(2015) [6]	3*t*_*e*_	*O*(1)	(*l* + 6)|*G*| + |*G*_*T*_|	*O*(*l*log*N*)	*O*(*lr*log(*N*/*r*))
Ryu(2015) [7]	(2*h* + 2)*t*_*e*_	*O*(*h*)	7|*G*| + |*G*_*T*_|	*O*(*h*log*N*)	*O*(*hr*log(*N*/*r*))
Lee(2016) [13]	(2*h* + 3)*t*_*e*_	*O*(*l*)	6|*G*| + |*G*_*T*_|	*O*(log*N*)	*O*(*l* + *r*log(*N*/*r*))
Lee(2016) [5]	4*t*_*e*_	*O*(*l*)	(*l* + 5)|*G*| + |*G*_*T*_|	*O*(*l*log*N*)	*O*(*l* + *r*log(*N*/*r*))
Xing(2016) [9]	(3*h* + 2)*t*_*e*_	*O*(*h*)	7|*G*| + |*G*_*T*_|	*O*(*h*logN)	*O*(*hr*log(*N*/*r*))
Our RHIBE	(4*h* + 4)*t*_*e*_	*O*(*h*)	9|*G*| + |*G*_*T*_|	*O*(*h*logN)	*O*(*h* + *r*log(*N*/*r*))

### 1.3 Related works

#### Efficient user revocation in RHIBE

An efficient tree-based key updating technique called the complete subtree (CS) method is a specific instance of the subset cover framework of Naor et al. [15]. In the scalable RIBEs using the CS method [16] [17] [14] [18] [19], every user holds a secret key composed of *logN* subkeys, where *N* is the number of all users, and only one subkey of a non-revoked user can be used to generate a decryption key. If we directly extend this mechanism to RHIBE scheme, the second-level user need to prepare (*logN*)^2^ subkeys since for every subkey of his parent he needs to generate *logN* subkeys respectively, which results to (*logN*)^*l*^ subkeys for an *l*-level user. Tsai et al. simply set the update key as another secret key in their RHIBE scheme [4]. Their construction is just as a trivial combination of two concurrent HIBE system, one for the derivation of secret keys and another for update keys. Lack of any efficient method of update and revocation, the size of the update key depends on the size of users linearly instead of logarithmically. Moreover, his approach require a new key center for update keys (called delegated revocation authority, DRA). That double deployment of key centers increases the system cost. Seo and Emura proposed a revocable HIBE scheme [1] with (*l*^2^*logN*)-size secret keys for a user, where *l* is the maximum hierarchical level. This history preserving update method leads to a lengthy history information in an update key and requires the recursive definition of secret keys and update keys. Afterward Seo proposed a RHIBE with (*l* ⋅ *logN*)-size secret keys for a user by a history-free update method. Recently, Lee and Park [13] proposed a new RHIBE scheme with shorter private keys and update keys by combining a new HIBE scheme that has short intermediate private keys and the CS scheme in a modular way, where the size of the secret key is (*logN*) and the size of the update key is (*l* + *rlog*(*N*/*r*)). Another revocation method called the subset difference (SD) method [20] was utilized to construct the RHIBE in [3] [13] [5]. Although this method has better performance in the transmission complexity, it has larger secret key size than the CS method.

#### Security model of R(H)IBE

Decryption key exposure resistance (DKER) has be considered by Seo [14], which discusses about the case where several decryption keys *dk*_I*, *T*_ for the target identity I* are leaked to an adversary but the target decryption key *dk*_I*, T*_ is not exposed. Another attacks should be considered like insiders attack [3]. Since the hierarchical structure in RHIBE determines that every user as a low-level KGC hold the state information about his low-level children users, a stronger security model than RIBE should be considered where it allows an insider adversary to access at least their own state information. The key re-randomization method [3] is an operable way to resist this attack and also decryption key enclosure attack mentioned in [3, 14].

#### Adaptive security of R(H)IBE

By employing dual system encryption methodology [10, 11], the adaptive-ID security can be directly proved in (H)IBE. But the security model of revocable HIBE is different from general HIBEs, since the system of RHIBE just not allow the decryption key query of the challenge identity and its ancestor at the challenge time, but allows the secret key query of the challenge identity and its ancestor identity. Therefore, the dual system encryption of RHIBE is more complex than general dual system of HIBE. Those adaptive-ID secure RHIBEs [6] [5] employed the dual system encryptions which are applicable to bounded schemes. Their proof strategy cannot be employed to unbounded (R)HIBE schemes, cause the limited entropy available in the public parameters in unbounded schemes makes it difficult to construct the nominally semi-functional key without information-theoretic exposure. By applying the dual system encryption methodology in prime-order, Yohei [21] realizes an RIBE scheme with constant-size public parameter under static assumptions in prime-order groups.

## 2 Preliminaries

### 2.1 Revocable HIBE

**Definition 1** *We define a RHIBE scheme π* = (*Setup*, *GenKey*, *DeriveKey*, *UpdateKey*, *Encrypt*, *Decrypt*, *Revoke*) *as following*:

1. ***Setup***(1^λ^): *It takes a security parameter* λ, *and outputs a master public key PP*, *a master secret key MK*, *initial state ST*_0_, *and an empty revocation list RL*. *Note that we don’t require the maximum number of users in each level as an input parameter*, *unlike the defination by all the bounded RHIBEs*.
2. ***GenKey***(*ID*|_*k*_, *ST*_*ID*|_*k*−1__, *PP*): *This algorithm takes as input ST*_*ID*|_*k*−1__ *and an identity ID*|_*k*_ *outputs the secret key SK*_*ID*|_*k*__, *and updates ST*_*ID*|_*k*−1__.
3. ***UpdateKey***(*T*, *RL*_*ID*|_*k*−1__, *DK*_*ID*|_*k*−1_,*T*_, *ST*_*ID*|_*k*−1__, *PP*): *This algorithm takes as input the revocation list RL*_*ID*|_*k*−1__, *state information ST*_*ID*|_*k*−1__, *the decryption key DK*_*ID*|_*k*−1_,*T*_,*and a time period T*. *Then*, *it outputs the update key UK*_*ID*|_*k*−1_,*T*_.
4. ***DeriveKey***(*SK*_*ID*|_*k*__, *UK*_*ID*|_*k*−1_,*T*_, *PP*): *This algorithm takes as input SK*_*ID*|_*k*__ *of ID*|_*k*_ *and UK*_*ID*|_*k*−1_,*T*_, *and outputs the decryption key DK*_*ID*|_*k*_, *T*_ *of ID*|_*k*_ *at time T if ID*|_*k*_ *is not revoked at T by the parent*, *else outputs* ⊥.
5. ***Encrypt***(*ID*|_*l*_, *T*, *M*, *PP*): *This algorithm takes as input a message M*, *ID*|_*l*_ *and the current time T and outputs the ciphertext CT*.
6. ***Decrypt***(*CT*_*ID*|_*l*_, *T*_, *DK*_*ID*′|_*k*_, *T*′_, *PP*): *This algorithm takes as input CT*_*ID*|_*l*_, *T*_ *and DK*_*ID*′|_*k*_, *T*′_, *and outputs the message if ID*′|_*k*_ *is a prefix of ID*|_*l*_ *and T T* = *T*′, *else outputs* ⊥.
7. ***Revoke***(*RL*_*ID*|_*k*−1__, *ST*_*ID*|_*k*−1__, *ID*|_*k*_, *T*): *This algorithm takes as input ID*|_*k*_ *and T*, *updates RL*_*ID*|_*k*−1__ *managed by ID*|_*k*−1_, *who is the parent user of ID*|_*k*_, *by adding* (*ID*|_*k*_, *T*).

**Definition 2** *We define an experiment under the adaptive-ID security against chosen plaintext attacks model in [5]*, *as named* “*IND-RID-CPA*” *security*.

$$\begin{array}{c} \begin{array}{c} Exp_{\pi ,A}^{IND-RID-CPA}\left(\lambda \right):\\ \left(MK,PP,RL\varepsilon ,ST\varepsilon \right)\leftarrow Setup\left(1^{\lambda }\right);\\ \left(M_{0}^{*},M_{1}^{*},ID{|}_{k}^{*},T^{*},ST\right)\leftarrow A^{O}\left(Find,PP\right);\\ b\overset{R}{\leftarrow }\left\{0,1\right\};CT^{*}\leftarrow Encrypt\left(PP,ID{|}_{k}^{*},T^{*},M_{b}^{*}\right);\\ b^{\prime }\leftarrow A^{O}\left(Guess,CT^{*},ST\right);Return\;1\;if\;b^{\prime }=b\;and\;0\;otherwise. \end{array} \end{array}$$

In the above experiment, *O* is a set of oracles {*SKGen*_*Q*_(⋅), *KeyUp*_*Q*_(⋅, ⋅), *Revoke*_*Q*_(⋅, ⋅), *DKGen*_*Q*_(⋅, ⋅)} defined as follows:

- *SKGen*_*Q*_(⋅): For *ID*|_*k*_ ∈ $I^{k}$, it returns *SK*_*ID*|_*k*__ (by running *GenKey*(*ID*|_*k*_, *ST*_*ID*|_*k*−1__, *PP*)→ *SK*_*ID*|_*k*__).
- *KeyUp*_*Q*_(⋅, ⋅): For *T* ∈ T and *BT*_*ID*|_*k*−1__, it returns *KU*_*T*, *ID*|_*k*−1__ (by running UpdateKey(*T*, *RL*_*ID*|_*k*−1__, *DK*_*ID*|_*k*−1__, *ST*_*ID*|_*k*−1__, *PP*) → *KU*_*t*_).
- *Revoke*_*Q*_(⋅, ⋅): For *ID*|_*k*_ ∈ $I^{k}$ and *T* ∈ T, it returns the updated revocation list RL (by running Revoke(*RL*_*ID*|_*k*−1__, *ST*_*ID*|_*k*−1__, *ID*|_*k*_, *T*)).
- *DKGen*_*Q*_(⋅, ⋅): For *ID*|_*k*_ ∈ $I^{k}$ and *T* ∈ T, it returns *DK*_*ID*|_*k*_, *T*_ (by running DeriveKey(*SK*_*ID*|_*k*__, *UK*_*ID*|_*k*−1_,*T*_, *PP*)→*DK*_*ID*|_*k*_, *T*_).

A is allowed to issue the above oracles with the following restrictions:

1. *Revoke*_*Q*_(⋅, ⋅) can be queried on time *T* if *KeyUp*_*Q*_(⋅) was queried on *T*.
2. *DKGen*_*Q*_(⋅, ⋅) cannot be queried on time *T* before *KeyUp*_*Q*_(⋅) was queried on *T*.
3. If A requested a private key query for $ID_{k}^{*}$ that is a prefix of $ID_{l}^{*}$ where *k* ≤ *l*, then the identity $ID_{k}^{*}$ or one of its ancestors should be revoked at some time *T* where *T* ≤ T*.
4. *A* cannot request a decryption key query for the challenge identity ID*|_*l*_ or its ancestors on the challenge time T*.
5. A cannot request a revocation query for *ID*|_*k*_ on time T if he already requested an update key query for *ID*|_*k*_ in time T.
6. A must query to KeyUp(⋅, ⋅) and Revoke(⋅, ⋅) for same identity in increasing order of time.

The advantage of A is defined as $Adv_{A}^{RHIBE}\left(\lambda \right)=\left|Pr\left(b=b^{\prime }\right)-0.5\right|$. We say that RHIBE is IND-RID-CPA secure if for all PPT adversary A, his advantage $Adv_{A}^{RHIBE}\left(\lambda \right)$ is negligible in the security parameter λ.

### 2.2 Complexity assumptions

We generate $(n,G,G_{T},e)\leftarrow G$ where *G* and *G*_*T*_ be cyclic groups with order *N* and *p* = *p*_1_ *p*_2_ *p*_3_, *p*_1_, *p*_2_, *p*_3_ are distinct prime numbers, *e*: *G*×*G*→ *G*_*T*_ is an efficient, nondegenerate bilinear map. We denote the subgroup of *G* with order *p*_*i*_ as *G*_*p*_*i*__. We define a function $Adv_{G,A}\left(\lambda \right)=\left|Pr\left[A\left(D,T_{1}\right)\right]-Pr\left[A\left(D,T_{2}\right)\right]\right|$ for any PPT algorithm A and parameters *D*, *T*_1_, *T*_2_.

**Assumption 1**. Let $g\overset{R}{\leftarrow }G_{p_{1}}$, D=(G,g), $T_{1}\overset{R}{\leftarrow }G_{p_{1}p_{2}}$, $T_{2}\overset{R}{\leftarrow }G_{p_{1}}$, we say that G satisfies Assumption 1 if $Adv_{G,A}\left(\lambda \right)$ is a negligible function of λ for any PPT algorithm is A.

**Assumption 2**. Let $g\overset{R}{\leftarrow }G_{p_{1}}$, $g_{2},X_{2},Y_{2}\overset{R}{\leftarrow }G_{p_{2}}$, $g_{3}\overset{R}{\leftarrow }G_{p_{3}}$, $\alpha ,s\overset{R}{\leftarrow }Z_{n}$, *T*_1_ be *e*(*g*, *g*)^*αs*^, $T_{2}\overset{R}{\leftarrow }G_{T}$, *D* = $\left(G,g,g_{2},g_{3},g^{\alpha }X_{2},g^{s}Y_{2}\right)$, we say that G satisfies Assumption 2 if $Adv_{G,A}\left(\lambda \right)$ is a negligible function of λ for any PPT algorithm is A.

**Assumption 3**. Let $g,X_{1}\overset{R}{\leftarrow }G_{p_{1}}$, $g_{2}\overset{R}{\leftarrow }G_{p_{2}}$, $X_{3}\overset{R}{\leftarrow }G_{p_{3}}$, $T_{1}\overset{R}{\leftarrow }G_{p_{1}}$, $T_{2}\overset{R}{\leftarrow }G_{p_{1}p_{3}}$, *D* = $\left(G,g,g_{2},X_{1}X_{3}\right)$, we say that G satisfies Assumption 3 if $Adv_{G,A}\left(\lambda \right)$ is a negligible function of λ for any PPT algorithm is A.

**Assumption 4**. Let $g,X_{1}\overset{R}{\leftarrow }G_{p_{1}}$, $X_{2},Y_{2}\overset{R}{\leftarrow }G_{p_{2}}$, $g_{3},Y_{3}\overset{R}{\leftarrow }G_{p_{3}}$, $T_{1}\overset{R}{\leftarrow }G_{p_{1}p_{3}}$, $T_{2}\overset{R}{\leftarrow }G$, *D* = $\left(G,g,g_{3},X_{1}X_{2},Y_{2}Y_{3}\right)$, we say that G satisfies Assumption 4 if $Adv_{G,A}\left(\lambda \right)$ is a negligible function of λ for any PPT algorithm is A.

## 3 Design of U-RHIBE system

We firstly describe the key encapsulation mechanism (KEM) version of the unbounded HIBE scheme [12] and its 1-level (H)IBE scheme that are used as the building blocks of our RHIBE schemes. Let $GS=\left(\left(N=p_{1}p_{2}p_{3},G,G_{T},e\right),g,g_{2},g_{3}\right)\leftarrow G\left(\lambda \right)$ be the bilinear group, where λ is a security parameter and *g*_2_ denotes a generator of *G*_*p*_2__, *g*_3_ denotes a generator of *G*_*p*_3__ and *g* be a generator of *G*_*p*_1__.

### 3.1 HIBE scheme

We define a key-group function *κ*(*I*, *y*, *r*) as the group elements

$$\begin{array}{c} \kappa \left(I,y,r\right)=(w^{y},g^{y},g^{r},{\left(u^{I}h\right)}^{r}v^{y}) \end{array}$$

and an expression *g*^λ^ *κ*(*I*, *y*, *r*) as

$$\begin{array}{c} g^{\lambda }\kappa \left(I,y,r\right)=\left(g^{\lambda }w^{y},g^{y},g^{r},{\left(u^{I}h\right)}^{r}v^{y}\right) \end{array}$$

**HIBE.Setup**(*GS*): It selects $u,h,w,v\overset{R}{\leftarrow }G_{p_{1}}$ and $\alpha \overset{R}{\leftarrow }Z_{p}$. It outputs a master key *MK* = *α* and public parameters *PP* = ((*p*, *G*, *G*_*T*_, *e*), *g*, *u*, *h*, *w*, *v*, Ω = *e*(*g*, *g*)^*α*^).

**HIBE.GenKey**(*ID*|_*k*_, *MK*, *PP*): Let the identity ${ID|}_{k}=\left(I_{1},\ldots ,I_{k}\right)\in I^{k}$, and $I={\left\{0,1\right\}}^{\lambda }$ be the identity space. It chooses $\lambda_{1}\cdots \lambda_{k},r_{1}\cdots r_{k},y_{1}\cdots y_{k}\overset{R}{\leftarrow }Z_{p}$ where λ_1_ + ⋯ + λ_*k*_ = *α* and outputs a private key $SK_{{ID|}_{k}}=\left\{K_{i}=g^{\lambda_{i}}\kappa \left(I_{i},y_{i},r_{i}\right),i=1\cdots k\right\}$.

**HIBE.RandKey**(*ID*|_*k*_, *SK*_*ID*|_*k*__, *PP*): Let $SK_{{ID|}_{k}}=\left({\left\{K_{i,0}^{\prime },K_{i,1}^{\prime },K_{i,2}^{\prime },K_{i,3}^{\prime }\right\}}_{i=1}^{k}\right)$. It chooses $\lambda_{1}\cdots \lambda_{k},r_{1}\cdots r_{k},y_{1}\cdots y_{k}\overset{R}{\leftarrow }Z_{p}$ where λ_1_ + ⋯ + λ_*k*_ = 0 and outputs a re-randomized private key $SK_{{ID|}_{k}}={\left\{K_{i,0}^{\prime }g^{\lambda_{i}}w^{y_{i}},K_{i,1}^{\prime }g^{y_{i}},K_{i,2}^{\prime }g^{r_{i}},{\left(u^{I_{i}}h\right)}^{r_{i}}v^{y_{i}}\cdot K_{i,3}^{\prime }\right\}}_{i=1}^{k})$.

**HIBE.Delegate**(*ID*|_*k*_, *SK*_*ID*|_*k*−1__, *PP*): Let $SK_{{ID|}_{k-1}}=\left({\left\{K_{i,0}^{\prime },K_{i,1}^{\prime },K_{i,2}^{\prime },K_{i,3}^{\prime }\right\}}_{i=1}^{k-1}\right)$. It chooses $\lambda_{1}\cdots \lambda_{k},y_{k},r_{k}\overset{R}{\leftarrow }Z_{p}$ where λ_1_ + ⋯ + λ_*k*_ = 0 and creates a temporal delegated private key $TSK_{{ID|}_{k}}=\left({\left\{K_{i,0}^{\prime }g^{\lambda_{i}},K_{i,1}^{\prime },K_{i,2}^{\prime },K_{i,3}^{\prime }\right\}}_{i=1}^{k-1},g^{\lambda_{k}}\kappa \left(I_{k},y_{k},r_{k}\right)\right)$. Next, it outputs a delegated private key *SK*_*ID*|_*k*__ by running HIBE.RandKey(*ID*|_*k*_, *TSK*_*ID*|_*k*__, *PP*).

**HIBE.Encaps**(*ID*|_*l*_, *s*, *PP*): Let *ID*|_*l*_ = (*I*_1_, …, *I*_*l*_) ∈ *I*^*l*^. It chooses $t_{1},\cdots ,t_{k}\overset{R}{\leftarrow }Z_{p}$ and outputs a ciphertext $CT_{{ID|}_{l}}=\left(g^{s},{\left\{w^{s}v^{t_{i}},{\left(u^{I_{i}}h\right)}^{t_{i}},g^{t_{i}}\right\}}_{i=1}^{l}\right)$ and a session key *EK* = Ω^*s*^.

**HIBE.Decaps**(*CT*_*ID*|_*l*__, *SK*_*ID*′|_*k*__, *PP*): Let $CT_{{ID|}_{l}}=\left(C_{0},{\left\{C_{i,1},C_{i,2},C_{i,3}\right\}}_{i=1}^{l}\right)$, $SK_{ID^{\prime }{|}_{k}}=\left(K_{0},{\left\{K_{i,1},K_{i,2},K_{i,3}\right\}}_{i=1}^{k}\right)$. If *ID*′|_*k*_ is a prefix of *ID*|_*l*_, it outputs a session key $EK=\prod_{i=1}^{k}(e\left(C_{0},K_{i,0}\right)e\left(C_{i,3},K_{i,3}\right)/(e\left(C_{i,1},K_{i,1}\right)$ *e*(*C*_*i*,2_, *K*_*i*,2_))). Otherwise, it outputs ⊥.

Additionally, we introduce two algorithms for our modular RHIBE construction, the ChangeKey algorithm and the MergeKey algorithm, which are defined similarly with the algorithms in [5].

**HIBE.ChangeKey**(*SK*_*ID*|_*K*__, *δ*, *PP*): Let $SK_{{ID|}_{k}}=\left({\left\{K_{i,0}^{\prime },K_{i,1}^{\prime },K_{i,2}^{\prime },K_{i,3}^{\prime }\right\}}_{i=1}^{k}\right)$. It chooses $\lambda_{1}\cdots \lambda_{k}\overset{R}{\leftarrow }Z_{p}$ where λ_1_ + ⋯ + λ_*k*_ = *δ* and sets $TSK=({\left\{K_{i,0}g^{\lambda_{i}},K_{i,1},K_{i,2},K_{i,3}\right\}}_{j=1}^{k})$. It outputs a new private key *SK*_*ID*|_*K*__ ← *HIBE*.*RandKey*(*ID*|_*k*_, *TSK*_(*n*)_, *PP*).

**HIBE.MergeKey**$\left(SK_{{ID|}_{K}}^{\left(1\right)},SK_{{ID|}_{K}}^{\left(2\right)},\eta ,PP\right)$: Let $SK_{{ID|}_{k}}^{\left(1\right)}=(\{K_{i,0}^{\prime },K_{i,1}^{\prime },K_{i,2}^{\prime },$ $K_{i,3}^{\prime }{\}}_{i=1}^{k})$ and $SK_{{ID|}_{k}}^{\left(2\right)}=\left({\left\{K_{i,0}^{\prime \prime },K_{i,1}^{\prime \prime },K_{i,2}^{\prime \prime },K_{i,3}^{\prime \prime }\right\}}_{i=1}^{k}\right)$ be two private keys for the same identity *ID*|_*K*_. It computes a temporal private key $TSK=({\left\{K_{i,0}^{\prime }\cdot K_{i,0}^{\prime \prime },K_{i,1}^{\prime }\cdot K_{i,1}^{\prime \prime },K_{i,2}^{\prime }\cdot K_{i,2}^{\prime \prime },K_{i,3}^{\prime }\cdot K_{i,3}^{\prime \prime }\right\}}_{i=1}^{k})$. Next, it outputs a merged private key *SK*_*ID*|_*K*__ ← *HIBE*.*ChangeKey*(*TSK*, *η*, *PP*). Note that the master key part is *α*_1_ + *α*_2_ + *η* if the master key parts of $SK_{{ID|}_{K}}^{\left(1\right)}$ and $SK_{{ID|}_{K}}^{\left(2\right)}$ are *α*_1_ and *α*_2_ respectively.

### 3.2 IBE scheme

A trivial extension to RHIBE from the HIBE in [12] constructs the decryption key of (*T*, *ID*|_*k*_) as $\left\{D_{0}=g^{\lambda_{0}}\kappa \left(T,y_{0},r_{0}\right),D_{i}=g^{\lambda_{i}}\kappa \left(I_{i},y_{i},r_{i}\right),i=1\cdots k|\sum_{i=0}^{k}\lambda_{i}=\alpha \right\}$. It remains some problem in the proof of RHIBE model, where the information theoretic argument is not easy to show as of the model of HIBE. So we modify the construction by defining a new update-key-group function as

$$\begin{array}{c} \kappa_{T}\left(T,y,r\right)=\left(w_{0}^{y},g^{y},g^{r},{\left(u_{0}^{T}h_{0}\right)}^{r}v_{0}^{y}\right) \end{array}$$ (1)

and *D*_0_ = $g^{\lambda_{0}}\kappa_{T}\left(T,y_{0},r_{0}\right)$, which is constructed from the component IBE secret key.

**IBE.Setup**(*GS*): It selects $u,h,w,v\overset{R}{\leftarrow }G_{p_{1}}$ and $\alpha \overset{R}{\leftarrow }Z_{p}$. It outputs a master key *MK* = *β* and public parameters *PP* = ((*p*, *G*, *G*_*T*_, *e*), *g*, *u*_0_, *h*_0_, *w*_0_, *v*_0_, Ω = *e*(*g*, *g*)^*β*^).

**IBE.GenKey**(*T*, *MK*, *PP*): This algorithm takes as input a time *T* and the master key *MK*, and the public parameters *PP*. It chooses $r,y\overset{R}{\leftarrow }Z_{p}$ and outputs a IBE secret key *SK*_*T*_ = *g*^*α*^ *κ*_*T*_(*T*, *y*, *r*).

**IBE.RandKey**(*T*, *SK*_*T*_, *PP*): Let the private key be $SK_{T}=\left(K_{0}^{\prime },K_{1}^{\prime },K_{2}^{\prime },K_{3}^{\prime }\right)$. It chooses $r,y\overset{R}{\leftarrow }Z_{p}$ and outputs a re-randomized private key $SK_{T}=\left(K_{0}=K_{0}^{\prime }w_{0}^{y},K_{1}=K_{1}^{\prime }g^{y},K_{2}=K_{2}^{\prime }g^{r},K_{3}=K_{3}^{\prime }{\left(u_{0}^{T}h_{0}\right)}^{r}v_{0}^{y}\right)$.

**IBE.Encaps**(*T*, *s*, *PP*): It chooses $t\overset{R}{\leftarrow }Z_{p}$ and outputs a ciphertext $CT_{T}=\left(C_{0}=g^{s},C_{1}=w_{0}^{s}v_{0}^{t},C_{2}={\left(u_{0}^{T}h_{0}\right)}^{t},C_{3}=g^{t}\right)$ and the session key *EK* = Ω^*s*^.

**IBE.Decaps**(*CT*_*T*_, *SK*_*T*′_, *PP*): Let the ciphertext *CT*_*T*_ = (*C*_0_, *C*_1_, *C*_2_, *C*_3_), the private key *SK*_*T*_ = (*K*_0_, *K*_1_, *K*_2_, *K*_3_). If *T* = *T*′, it outputs a session key *EK* = *e*(*C*_0_, *K*_0_)*e*(*C*_3_, *K*_3_)/(*e*(*C*_1_, *K*_1_) *e*(*C*_2_, *K*_2_)). Otherwise, it outputs ⊥.

The contruction of IBE.ChangeKey and IBE.MergeKey is similar with HIBE.ChangeKey and HIBE.MergeKey and we omit them here.

### 3.3 The CS method

We exploit the complete subtree (CS) method to construct our RHIBE scheme. We follow the definition of the CS scheme in the work of Lee and Park [22].

**CS.Setup**(*N*_*max*_): Let *N*_*max*_ = 2^*n*^. It first sets a full binary tree BT of depth *n*. Each user is assigned to a different leaf node in BT. The collection *S* is defined as {*S*_*i*_} where *S*_*i*_ is the set of all leaves in a subtree $T_{i}$ with a subroot $v_{i}\in BT$. It outputs the full binary tree BT.

**CS.Assign**(BT,ID): Let *v*_*ID*_ be a leaf node of BT that is assigned to the user *ID*. Let (*v*_*k*_0__, *v*_*k*_1__, ⋯, *v*_*k*_*n*__) be the path from the root node *v*_*k*_0__ = *v*_0_ to the leaf node *v*_*k*_*n*__ = *v*_*ID*_. For all *j* ∈ {*k*_0_, ⋯, *k*_*n*_}, it adds *S*_*j*_ into *PV*_*ID*_. It outputs the private set *PV*_*ID*_ = {*S*_*j*_}.

**CS.Cover**(BT,R): It first computes the Steiner tree *ST*(*R*). Let $T_{k_{1}},\cdots ,T_{k_{m}}$ be all the subtrees of BT that hang off *ST*(*R*), that is all subtrees whose roots *v*_*k*_1__, ⋯, *v*_*k*_*m*__ are not in *ST*(*R*) but adjacent to nodes of outdegree 1 in *ST*(*R*). For all *i* ∈ {*k*_1_, ⋯, *k*_*m*_}, it adds *S*_*i*_ into *CV*_*R*_. It outputs a covering set *CV*_*R*_ = {*S*_*i*_}.

**CS.Match**(*CV*_*R*_, *PV*_*ID*_): It finds a subset *S*_*k*_ with *S*_*k*_ ∈ *CV*_*R*_ and *S*_*k*_ ∈ *PV*_*ID*_. If there is such a subset, it outputs *S*_*k*_. Otherwise, it outputs ⊥.

### 3.4 Construction

**RHIBE.Setup**(1^λ^, *N*_*max*_): The Setup algorithm takes a security parameter λ and a maximum number of users for each level *N*_*max*_ as input. It firstly runs G to obtains two groups *G*, *G*_*T*_ of order *p* = *p*_1_*p*_2_*p*_3_, where *p*_1_, *p*_2_, *p*_3_ are distinct primes, and a bilinear map *e*: *G*×*G*→*G*_*T*_. It sets *GS* = ((*N*, *G*, *G*_*T*_, *e*), *g*, *g*_2_, *g*_3_) where *g*, *g*_2_ and *g*_3_ denote the generators of *G*_*p*_1__, *G*_*p*_2__, and *G*_*p*_3__ in order. It selects a random exponent *α* ∈ *Z*_*p*_, set Ω be *e*(*g*, *g*)^*α*^. It outputs a master key *MK* = *α* and public parameters *PP* = (*PP*_*HIBE*_, *PP*_*IBE*_, Ω, *N*_*max*_), where *PP*_*HIBE*_ ← *HIBE*.*Setup*(*GS*), and *PP*_*IBE*_ ← *IBE*.*Setup*(*GS*).

**RHIBE.GenKey**(*ID*|_*k*_, *ST*_*ID*|_*k*−1__, *PP*): This algorithm takes as input an identity *ID*|_*k*_ = (*I*_1_, …, *I*_*k*_) ∈ $I^{k}$, the state *ST*_*ID*|_*k*−1__ which contains *BT*_*ID*|_*k*−1__.

1. If *ST*_*ID*|_*k*−1__ is empty, it obtains *BT*_*ID*|_*k*−1__ ← *CS*.*Setup*(*N*_*max*_) and then it sets *ST*_*ID*|_*k*−1__ = (*BT*_*ID*|_*k*−1__, *β*_*ID*_*k*−1__, *z*_*ID*_*k*−1__), where *β*_*ID*_*k*−1__ is a false master key and *z*_*ID*_*k*−1__ is a PRF key.
2. It first assigns *ID*|_*k*_ to a random leaf node *v* ∈ *BT*_*ID*|_*k*−1__ and obtains a node set *Path*(*ID*|_*k*_) ← *CS*.*Assign*(*BT*_*ID*|_*k*−1__, *ID*|_*k*_) for *ID*|_*k*_. For each *S*_*θ*_ ∈ *Path*, it computes *γ*_*θ*_ = **PRF** (*z*_*ID*_*k*−1__, *L*_*θ*_) where *L*_*θ*_ = **Label** (*S*_*θ*_) and obtains an HIBE private key *SK*_*HIBE*,*S*_*θ*__ ← *HIBE*.*GenKey*(*ID*|_*k*_, *γ*_*θ*_, *PP*). Finally, it outputs a private key *SK*_*ID*|_*k*__ = (*Path*, {*SK*_*HIBE*,*S*_*θ*__}_*S*_*θ*_∈*Path*_). Note that the master key part of *SK*_*HIBE*,*S*_*θ*__ is *γ*_*θ*_.

**RHIBE.UpdateKey**(*T*, *RL*_*ID*|_*k*−1__, *DK*_*ID*|_*k*−1__, *ST*_*ID*|_*k*−1__, *PP*): let $DK_{{ID|}_{k-1},T}=\left(RSK_{{HIBE,ID|}_{k-1}},RSK_{IBE,T}^{\prime }\right)$, the state *ST*_*ID*|_*k*−1__ = (*BT*_*ID*|_*k*−1__, *β*_*ID*|_*k*−1__, *z*_*ID*|_*k*−1__) with *k* ≥ 1.

1. It first obtains a randomized decryption key *RDK*_*ID*|_*k*−1_,*T*_ as (*RSK*_*IBE*_, *RSK*_*HIBE*_)←*RHIBE*.*RandDK*(*DK*_*ID*|_*k*_,*T*_, −*β*_*ID*|_*k*−1__, *PP*).
2. It derives the set of revoked identities *R* at time *T* from *RL*_*ID*|_*k*−1__. Next, it obtains a covering set *CV*_*R*_ = {*S*_*i*_} by running **CS.Cover**(*BT*_*ID*|_*k*−1__, *R*).
3. For each *S*_*i*_ ∈ *CV*_*R*_, it computes *γ*_*i*_ = **PRF**(*z*_*ID*_*k*−1__, *L*_*i*_) where *L*_*i*_ = **Label**(*S*_*i*_) and obtains an IBE private key $SK_{IBE,S_{i}}^{\prime }\leftarrow IBE.GenKey\left(T,-\gamma_{i},PP\right)$. Then It computes $SK_{IBE,S_{i}}\leftarrow IBE.MergeKey\left(SK_{IBE,S_{i}}^{\prime },RSK_{IBE},\beta_{ID_{k-1}},PP\right)$
4. It finally outputs an update key *UK*_*ID*|_*k*−1_,*T*_ = (*CV*_*R*_, {*SK*_*IBE*,*S*_*i*__, *RSK*_*HIBE*_}_*S*_*i*_ ∈ *CV*_*R*__). Note that the master key parts of *RSK*_*HIBE*_ and *SK*_*IBE*, *S*_*i*__ are *η*′ and *α* − *η*′ − *γ*_*i*_ for some random *η*′ respectively.

**RHIBE.DeriveKey**(*SK*_*ID*|_*k*__, *UK*_*ID*|_*k*−1_,*T*_, *PP*): This algorithm takes as input a private key *SK*_*ID*|_*k*__ = (*Path*, {*SK*_*HIBE*,*S*_*θ*__}_*S*_*θ*_ ∈ *Path*_) for an identity *ID*|_*k*_, an update key $UK_{{ID|}_{k-1},T}=\left(CV_{R},{\left\{SK_{IBE,S_{i}},RSK_{{HIBE,ID|}_{k-1}}^{\prime }\right\}}_{S_{i}\in CV_{R}}\right)$ for time *T*.

1. If *K* = 0, then *SK*_*ID*|_0__ = *MK* = *α* and *UK*_*ID*|_−1_,*T*_ is empty. It selects a random exponent *η* ∈ *Z*_*p*_. It then obtains *RSK*_*HIBE*, *ID*|_0__ ← *HIBE*.*GenKey*(*ID*|_0_, *η*, *PP*) and *RSK*_*IBE*,*T*_ ← *IBE*.*GenKey*(*T*, *α* − *η*, *PP*). It outputs a decryption key *DK*_*ID*|_0_,*T*_ = (*RSK*_*IBE*, *T*_, *RSK*_*HIBE*, *ID*|_0__).
2. If *k* ≥ 1, then if *ID*|_*k*_ ∉ *RL*_*ID*|_*k*−1__, then it obtains (*S*_*i*_, *S*_*i*_) by running **CS.Match**(*CV*_*R*_, *Path*). Otherwise, it outputs ⊥. It derives *SK*_*HIBE*,*S*_*i*__ from *SK*_*ID*|_*k*__ and *SK*_*IBE*,*S*_*i*__ from *UK*_*ID*|_*k*−1_,*T*_.
3. It obtains $RSK_{HIBE,{ID|}_{k}}^{\prime \prime }\leftarrow HIBE.Delegate{\left(ID\right|}_{k},RSK_{{HIBE,ID|}_{k-1}}^{\prime },$ *PP*) since *ID*|_*k*−1_ ∈ Prefix(*ID*|_*k*_). Next, it selects a random exponent *η* ∈ *Z*_*p*_, obtains $RSK_{{HIBE,ID|}_{k}}\leftarrow HIBE.MergeKey(RSK_{{HIBE,ID|}_{k}}^{\prime \prime },$ *SK*_*HIBE*,*S*_*i*__, *η*, *PP*) and obtains *RSK*_*IBE*,*T*_ ← *IBE*.*ChangeKey*(*SK*_*IBE*,*S*_*i*__, −*η*, *PP*) respectively. Finally, it outputs a decryption key *DK*_*ID*|_*k*_,*T*_ = (*RSK*_*IBE*,*T*_, *RSK*_*HIBE*, *ID*|_*k*__).

Note that the master key parts of *RSK*_*HIBE*, *ID*|_*k*__ and *RSK*_*IBE*, *T*_ are *η*′ and *α* − *η*′ for some random *η*′ respectively.

**RHIBE.RandDK**$\left(DK_{{ID|}_{k},T}^{\prime },\beta ,PP\right)$: Let $DK_{{ID|}_{k},T}^{\prime }=\left(RSK_{IBE,T}^{\prime },RSK_{{HIBE,ID|}_{k}}^{\prime }\right)$, and *β* ∈ *Z*_*p*_ be an exponent. It first selects a random exponent *η* and obtains $RSK_{{HIBE,ID|}_{k}}\leftarrow HIBE.ChangeKey\left(RSK_{{HIBE,ID|}_{k}}^{\prime },\eta ,PP\right)$ and $RSK_{IBE,T}\leftarrow IBE.ChangeKey(RSK_{IBE,T}^{\prime },-\eta +\beta ,$ *PP*). It outputs a re-randomized decryption key *DK*_*ID*|_*k*_,*T*_ = (*RSK*_*IBE*,*T*_, *RSK*_*HIBE*, *ID*|_*k*__).

**RHIBE.Encrypt**(*ID*|_*l*_, *T*, *M*, *PP*): This algorithm takes as input an identity ${ID|}_{l}=\left(I_{1},\ldots ,I_{l}\right)\in I^{l}$, time *T*, a message M∈M. It chooses a random exponent *t* ∈ *Z*_*p*_. Next it obtains (*CH*_*HIBE*,*ID*|_*l*__, *EK*_*HIBE*_) ← *HIBE*.*Encaps*(*ID*|_*l*_, *t*, *PP*). It also obtains (*CH*_*IBE*,*T*_, *EK*_*IBE*_) ← *IBE*.*Encaps*(*T*, *t*, *PP*). It outputs a ciphertext *CT*_*ID*|_*k*_,*T*_ = (*CH*_*IBE*,*T*_, *CH*_*HIBE*,*ID*|_*l*__, *C* = Ω^*t*^ ⋅ *M*).

**RHIBE.Decrypt**(*CT*_*ID*|_*l*_,*T*_, *DK*_*ID*′|_*k*_,*T*′_, *PP*): This algorithm takes as input a ciphertext *CT*_*ID*|_*l*_,*T*_ = (*CH*_*IBE*,*T*_, *CH*_*HIBE*,*ID*|_*l*__, *C*), a decryption key *DK*_*ID*′|_*k*_,*T*′_ = (*RSK*_*IBE*,*T*′_, *RSK*_*HIBE*,*ID*′|_*k*__). If *ID*′|_*k*_ is a prefix of *ID*|_*l*_ and *T* = *T*′, then it obtains *EK*_*HIBE*_ ← *HIBE*.*Decaps*(*CH*_*HIBE*,*ID*|_*l*__, *RSK*_*HIBE*,*ID*|_*k*__, *PP*) and *EK*_*IBE*_ ← *IBE*.*Decaps*(*CH*_*IBE*,*T*_, *RSK*_*IBE*,*T*_, *PP*). Otherwise, it outputs ⊥. It outputs an encrypted message by computing *M* = *C* ⋅ (*EK*_*HIBE*_ ⋅ *EK*_*IBE*_)^−1^.

**RHIBE.Revoke**(*ID*|_*k*_, *T*, *RL*_*ID*|_*k*−1__, *ST*_*ID*|_*k*−1__): This algorithm takes as input an identity *ID*|_*k*_, revocation time *T*, the revocation list *RL*_*ID*|_*k*−1__, and the state *ST*_*ID*|_*k*−1__. If (*ID*|_*k*_, −) ∉ *ST*_*ID*|_*k*−1__, then it outputs ⊥ since the private key of *ID*|_*k*_ was not generated. Otherwise, it adds (*ID*|_*k*_, *T*) to *RL*_*ID*|_*k*−1__ and outputs the updated revocation list *RL*_*ID*|_*k*−1__.

### 3.5 Correctness

If a user is not revoked at time *T*, the RHIBE.DeriveKey algorithm correctly derive his decryption key *DK*_*ID*|_*k*_,*T*_ as

$$\begin{array}{c} \left(g^{\alpha -\prod_{i=1}^{k}\lambda_{i}}w_{0}^{b_{0}},g^{b_{0}},g^{r_{0}},{\left(u_{0}^{T}h_{0}\right)}^{r_{0}}v_{0}^{b_{0}},{\left\{g^{\lambda_{i}}w^{b_{i}},g^{b_{i}},g^{r_{i}},{\left(u^{I_{i}}h\right)}^{r_{i}}v^{b_{i}}\right\}}_{i=1}^{k}\right) \end{array}$$

The RHIBE.Decrypt algorithm takes *CT*_*ID*|_*l*_,*T*_ as input, where

$$\begin{array}{c} CT_{{ID|}_{l},T}=\left(g^{s},w_{0}^{s}v_{0}^{t},{\left(u_{0}^{T}h_{0}\right)}^{t},g^{t},{\left\{w^{s}v^{t_{i}},{\left(u^{I_{i}}h\right)}^{t_{i}},g^{t_{i}}\right\}}_{i=1}^{l}\right), \end{array}$$

and computes *B* = *C*/*M* as

$$\begin{array}{c} B=\frac{e\left(g^{s},g^{\alpha }w_{0}^{b_{0}}\prod_{i=1}^{k}w^{b_{i}}\right)e\left(g^{t_{0}},{\left(u_{0}^{I_{0}}h_{0}\right)}^{r_{0}}v_{0}^{b_{0}}\right)}{e\left(w_{0}^{s}v_{0}^{t_{0}},g^{b_{0}}\right)e\left({\left(u_{0}^{T}h_{0}\right)}^{t_{0}},g^{r_{0}}\right)}\prod_{i=1}^{k}\frac{e(g^{t_{i}},{\left(u^{I_{i}}h\right)}^{r_{i}}v^{b_{i}})}{\left(e\left(w^{s}v^{t_{i}},g^{b_{i}}\right)e\left({\left(u^{I_{i}}h\right)}^{t_{i}},g^{r_{i}}\right)\right)}=\Omega^{s} \end{array}$$

## 4 Security analysis

We use the dual system encryption proof techinique to prove the adaptive security of our U-RHIBE. We adopt the concept of *ephemeral semi-functionality* [12] and design a new nested dual system encryption for unbounded RHIBEs. As an intermediary transforming stage between the normal and semi-functional distributions, the ephemeral semi-functionality helps us to overcome the challenge presented by low entropy in the public parameters.

**Theorem 1** *Our unbounded RHIBE scheme is IND-RID-CPA secure if Assumption 1–4 hold*.

***Proof*** We firstly define the semi-functional type and the ephemeral semi-functional types of keys and ciphertexts in Sec.4.1 which represent the types of keys and ciphertexts answered to the queries in the challenge game. Secondly we conduct the security proof by the indistinguishabilities of a sequence of hybrid games that we define in Sec.4.2.

### 4.1 Definition of (ephemeral) semi-functional keys and ciphertexts

For constructing the different types of ciphertexts, secret keys, update keys and decryption keys, the challenger B is initially given renadom elements *g*, *u*, *v*, *w*, *u*_0_, *v*_0_, *w*_0_ ∈ *G*_*p*_1__, *g*_2_ ∈ *G*_*p*_2__, *g*_3_ ∈ *G*_*p*_3__, as well as random exponents *ψ*_1_, *ψ*_2_, *σ*_1_, *σ*_2_, *a*′, *b*′, *s*, *δ*_1_, *δ*_2_, *γ*.

We define the semi-functional ciphertext and five types of ephemeral semi-functional ciphertexts of a normal ciphertext *CT*_*ID*|_*l*_,*T*_ by changing the *C*_0_ element into *G*_*p*_1_*p*_2__ and the *l* + 1 numbers of the ciphertext-element-groups (*C*_*i*,1_, *C*_*i*,2_, *C*_*i*,3_) into different types. The definations of ephemeral semi-functional ciphertexts called **ESF-1-CT**, **ESF-2^k^-CT**, **ESF-3^k^-CT**, **ESF-4^k^-CT** and **ESF-5-CT** where 0 ≤ *k* ≤ *l* are in Appendix.A. In the definations of the semi-functional ciphertext, we add *G*_*p*_2__ term on the first element of all ciphertext-element-groups.

**RHIBE.EncryptSF**: It firstly obtains the normal ciphertext *CT*_*ID*|_*l*_,*T*_ = (*C*, *C*_0_, ${\left\{C_{i,1},C_{i,2},C_{i,3}\right\}}_{i=0}^{l}$) for an identity ${ID|}_{l}=\left(I_{1},\ldots ,I_{k}\right)\in I^{k}$, a time T∈T and a message M∈M. It chooses exponents *γ*, *δ*_1_, *δ*_2_ ∈ **Z**_*p*_ and outputs the SF-CT ${\tilde{CT}}_{{ID|}_{l},T}$ as

$$\begin{array}{c} \left(C,C_{0}\cdot g_{2}^{\gamma },C_{0,1}\cdot g_{2}^{\delta_{2}},C_{0,2},C_{0,3},{\left\{C_{i,1}\cdot g_{2}^{\delta_{1}},C_{i,2},C_{i,3}\right\}}_{i=1}^{l}\right) \end{array}$$

As we mentioned before, our normal secret key and update key cannot be simply changed to semi-functional keys as same as in [11] one by one owing to the inefficiency of the information theoretic argument in our scheme. And we divide secret keys and update keys into samll component keys which are group together if they are related to the same node in a binary tree.

We only change the last element-group of our normal secret key for constructing the semi-functional secret key and the ephemeral semi-functional secret key like in [11]. We define one type of semi-functional secret key and five types of ephemeral semi-functional secret key. The defination of ephemeral semi-functional secret key called **ESF-1-SK**, **ESF-2-SK**, **ESF-3-SK**, **ESF-4-SK** and **ESF-5-SK** are in Appendix.A. In the defination of the semi-functional secret key, we add *G*_*p*_2_*p*_3__ term on the first 2 elements and the last element of the last element-group.

**RHIBE.SKeySF** $\left({ID|}_{j},ST_{{ID|}_{j-1}},PP,\theta \right)\to {\tilde{SK}}_{HIBE,S_{\theta }}$: It constructs the correlative sub-key $SK_{HIBE,S_{\theta }}=\left(\{{K_{i,0},K_{i,1},K_{i,2},K_{i,3}\}}_{i=1}^{j}\right)$ to the node *θ* ∈ *Path*(*ID*_*j*_) in the *BT*_*ID*|_*j*−1__ as follows: It chooses random exponents *y*′, *r* ∈ **Z**_*p*_ and choose *σ*_1_, *ψ*_1_ ∈ **Z**_*p*_, then it constructs *κ*^*sf*^(*I*_*j*_, *y*′, *r*) for the last element-group as

$$\begin{array}{c} \left(w^{y^{\prime }}{\left(g_{2}g_{3}\right)}^{y^{\prime }\psi_{1}},g^{y^{\prime }}{\left(g_{2}g_{3}\right)}^{y^{\prime }},g^{r},v^{y^{\prime }}{\left(g_{2}g_{3}\right)}^{y^{\prime }\sigma_{1}}{\left(u^{I_{j}}h\right)}^{r}\right) \end{array}$$

And the contruction of the other element-groups follows the construction of *SK*_*HIBE*,*S*_*θ*__ in RHIBE.GenKey.

We define one type of semi-functional update key and five types of ephemeral semi-functional update key. The defination of ephemeral semi-functional update key called **ESF-1-UK**, **ESF-2-UK**, **ESF-3-UK**, **ESF-4-UK** and **ESF-5-UK** are in Appendix.A. The constructions from the normal component update key to the (ephemeral) semi-functional component update keys are similar to that of secret keys, expect that we change the first element group of normal component update key to different types.

**RHIBE.UpdateKeySF** $\left(T,ST_{{ID|}_{k-1}},RL_{{ID|}_{k-1},T},PP,\theta \right)\to \tilde{TUK}$: It constructs the correlative component key $TUK_{{ID|}_{j-1},T,\theta }=\left({\left\{U_{i,0},U_{i,1},U_{i,2},U_{i,3}\right\}}_{i=0}^{k-1}\right)$ to the node *θ* ∈ *KUNode* as follows: It chooses random exponents *y*′, *r* ∈ **Z**_*p*_ and choose *σ*_2_, *ψ*_2_ ∈ **Z**_*p*_, then it constructs $\kappa_{T}^{sf}\left(T,y^{\prime },r\right)$ of the first element-group (*U*_0,0_, *U*_0,1_, *U*_0,2_, *U*_0,3_) as

$$\begin{array}{c} \left(w_{0}^{y^{\prime }}{\left(g_{2}g_{3}\right)}^{y^{\prime }\psi_{2}},g^{y^{\prime }}{\left(g_{2}g_{3}\right)}^{y^{\prime }},g^{r},v_{0}^{y^{\prime }}{\left(g_{2}g_{3}\right)}^{y^{\prime }\sigma_{2}}{\left(u_{0}^{T}h_{0}\right)}^{r}\right) \end{array}$$

And the contruction of the other element-groups follows the construction of *RSK*_*HIBE*_ and *SK*_*IBE*,*S*_*θ*__ in RHIBE.UpdateKey.

**RHIBE.DeriveKeySF**: Let ${\tilde{SK}}_{ID_{k}}$ be a semi-functional secret key generated by the RHIBE.GenKeySF algorithm and ${\tilde{UK}}_{{ID|}_{k-1},T}$ be a semi-functional update key for time *T* generated by the RHIBE.UpdateKeySF algorithm. If *ID*|_*k*_ ∉ *RL*_*ID*|_*k*−1__, then it finds a unique node θ* by running CS.Match(*CV*_*R*_(*BT*_*ID*|_*k*−1__, *RL*_*ID*|_*k*−1__, *T*), *Path*(*ID*|_*k*_)). Otherwise, it outputs ⊥. It derives ${\tilde{PSK}}_{\theta^{*}}=\left({\left\{{\tilde{K}}_{i,0},{\tilde{K}}_{i,1},{\tilde{K}}_{i,2},{\tilde{K}}_{i,3}\right\}}_{i=1}^{k}\right)$ from ${\tilde{SK}}_{{ID|}_{k}}$ and ${\tilde{TUK}}_{\theta^{*}}=\left({\left\{{\tilde{U}}_{i,0},{\tilde{U}}_{i,1},{\tilde{U}}_{i,2},{\tilde{U}}_{i,3}\right\}}_{i=0}^{k-1}\right)$ from ${\tilde{UK}}_{{ID|}_{k-1},T}$ for the node θ*. Then the semi-functional decryption key ${\tilde{DK}}_{{ID|}_{k},T}$ is ${\tilde{DK}}_{{ID|}_{k},T}=\left({\left\{{\tilde{D}}_{i,0},{\tilde{D}}_{i,1},{\tilde{D}}_{i,2},{\tilde{D}}_{i,3}\right\}}_{i=0}^{k}\right)$ as

$$\begin{array}{c} \left({\tilde{U}}_{0,0},{\tilde{U}}_{0,1},{\tilde{U}}_{0,2},{\tilde{U}}_{0,3},\{{{\tilde{U}}_{i,0}{\tilde{K}}_{i,0},{\tilde{U}}_{i,1}{\tilde{K}}_{i,1},{\tilde{U}}_{i,2}{\tilde{K}}_{i,2},{\tilde{U}}_{i,3}{\tilde{K}}_{i,3}\}}_{i=1}^{k-1},{\tilde{K}}_{k,0},{\tilde{K}}_{k,1},{\tilde{K}}_{k,2},{\tilde{K}}_{k,3}\right) \end{array}$$

Then we re-randomize it by running RHIBE.RandDK and output it.

### 4.2 Sequence of games

We define a squence of games to verify the advantage in distinguishing *G*_*Real*_ and *G*_*Final*_ is negligible. In Table 3, we give the types of key in the queries and the challenge cipertext in every game, and the decryption situation according to the types of keys and ciphertexts.

**Table 3:** Defination of games.

Games	Oracles	Key Types in Queries			Challenge Ciphertext			
		SK	UK	DK	*Normal*	*SF*	*ESF* − 1	*ESF* − 5
*G*_*real*_		*Normal*	*Normal*	*Normal*	√			
*G*_*C*_		*Normal*	*Normal*	*Normal*		√		
*G*_*C*′_	*O*_0_	*Normal*	*Normal*	*Normal*		√		
*G*_*E*−*S*_	*O*_1_	*ESF* − 2	*Normal*	*Normal*		√		
*G*_*E*−*U*_	*O*_1^+^_	*Normal*	*ESF* − 2	*Normal*				
*G*_*E*−*S*′_	*O*_2_	*ESF* − 2	*Normal*	*Normal*			◯	
*G*_*E*−*U*′_	*O*_2+_	*Normal*	*ESF* − 2	*Normal*				
*G*_*ESF*′_	*O*_3_	*ESF* − 2	*ESF* − 2	*Normal*			◯	
*G*_*SF*″_	*O*_4_	*SF*	*SF*	*Normal*		◯		
*G*_*E*−*D*_	*O*_5_	*SF*	*SF*	*ESF* − 2		◯		
*G*_*ESF*_	*O*_6_	*SF*	*SF*	*ESF* − 2			⨀	
*G*_*SF*′_	*O*_7_	*SF*	*SF*	*SF*		⨀		
*G*_*SF*_		*SF*	*SF*	*SF*		⨀		

The decryption situation according to the type of keys and ciphertexts in different games is for the challenger B to check whether the keys are nominally semi-functional keys. √ means that the decryption key answered by the query *DKGen*_*Q*_ and the derived decryption key from the corresponding secret key and update key outputed by *SKGen*_*Q*_ and *KeyUp*_*Q*_ both are able to decrypt the ciphertext. ◯ means that only the decryption key answered by the query *DKGen*_*Q*_ is able to decrypt the ciphertext. ⨀ means that neither the queried decryption key nor the derived decryption key is able to decrypt the ciphertext.

**G**_**Real**_: It is the original game in which all seceret keys, update keys, decryption keys and ciphertexts are normal.

**G**_**C**_: The challenge ciphertext is changed to be semi-functional and all other keys are still normal.

**G**_**C′**_: This game is exactly like *Game*_*C*_, except for a added restriction about the challenge key identity vector. We explain the restriction in Sec.4.6.

**G**_**E−S**_: The secret keys are changed to ESF-2. The update keys and decryption keys are still normal. The challenge ciphertext is semi-functional. This game is used in the proof of the security against Type-1 adversary.

**G**_**E−U**_: The update keys are changed to ESF-2. The secret keys and decryption keys are still normal. The challenge ciphertext is semi-functional. This game is used in the proof of the security against Type-2 adversary.

**G**_**E−S′**_: This game is almost as same as *G*_*E*−*S*_ except the challenge ciphertext is chaged to ESF-1. This game is used in the proof of the security against Type-1 adversary.

**G**_**E−U′**_: This game is almost as same as *G*_*E*−*U*_ where the update keys are ESF-2, the secret keys and decryption keys are normal, except the challenge ciphertext is chaged to ESF-1. This game is used in the proof of the security against Type-2 adversary.

**G**_**ESF′**_: The update keys and secret keys are all changed to ESF-2. The challenge ciphertext is changed to ESF-1. The decryption keys are still normal.

**G**_**SF″**_: All secret keys, update keys, and challenge ciphertext are changed to semi-functional. The decryption keys are still normal.

**G**_**E−D**_: The decryption keys are changed to ESF-2. The other keys and the challenge ciphertext are still semi-functional.

**G**_**ESF**_: The challenge ciphertext is changed to ESF-1. The update keys and secret keys are all still semi-functional. The decryption keys are still ESF-2.

**G**_**SF′**_: The challenge ciphertext is changed to semi-functional. The decryption keys are changed to be semi-functional. That is, all secret keys, update keys, decryption keys, and challenge ciphertext are now semi-functional. This game is exactly like *G*_*SF*_, except for a added restriction about the challenge key identity vector. We explain the restriction in Sec.4.6.

**G**_**SF**_: The challenge ciphertext and all keys are semi-functional.

**G**_**Final**_: The session key is changed to be random and so the adversary has no advantage to distinguish the challenge massage.

Let $Adv_{A}^{RHIBE}$ be the advantage of A in the real game. From the all the lemmas in this section, we obtain the following equation

$$\begin{array}{c} \begin{array}{c} Adv_{A}^{RHIBE}\left(\lambda \right)\leq |Adv_{A}^{G_{Real}}\left(\lambda \right)-Adv_{A}^{G_{C}}\left(\lambda \right)|+|Adv_{A}^{G_{C}}\left(\lambda \right)-Adv_{A}^{G_{C^{\prime }}}\left(\lambda \right)|\\ \;+|Adv_{A}^{G_{C^{\prime }}}\left(\lambda \right)-Adv_{A}^{G_{SF^{\prime }}}\left(\lambda \right)|+|Adv_{A}^{G_{SF^{\prime }}}\left(\lambda \right)-Adv_{A}^{G_{SF}}\left(\lambda \right)|\\ \;+|Adv_{A}^{G_{SF}}\left(\lambda \right)-Adv_{A}^{G_{Final}}\left(\lambda \right)|\\ \;\leq Adv_{B}^{A1}\left(\lambda \right)+Adv_{B}^{A2}\left(\lambda \right)\\ \;+\left(O\left(q_{n}logN_{max}\right)+O\left(q_{n}r_{max}logN_{max}\right)+O\left(l\right)\right)\left(Adv_{B}^{A3}+Adv_{B}^{A4}\right) \end{array} \end{array}$$

### 4.3 Definition of oracles

We introduce seven oracles which answer queries from the challenger B by sampling various distributions of group elements from a composite order bilinear group. The outputs of Oracle *O*_*i*_ will allow a simulator to produce different type of secret keys, update keys and decryption keys, different type of ciphertext and challenge keys for one corresponding game demonstrated in Table 3.

All oracles are defined with respect to a bilinear group *G* of order *p* = *p*_1_*p*_2_*p*_3_ and initially choose random elements *g*, *u*, *v*, *w*, *u*_0_, *v*_0_, *w*_0_ ∈ *G*_*p*_1__, *g*_2_ ∈ *G*_*p*_2__, *g*_3_ ∈ *G*_*p*_3__ as well as random exponents *ψ*_1_, *ψ*_2_, *σ*_1_, *σ*_2_, *a*′, *b*′, *s*, *δ*_1_, *δ*_2_, *γ* ∈ *Z*_*n*_. They provide the attacker with a description of the group *G*, as well as the group elements

$$\begin{array}{c} \begin{array}{c} g,u,v,w,g^{s}g_{2}^{\gamma },w^{y}{\left(g_{2}g_{3}\right)}^{y\psi_{1}},g^{y}{\left(g_{2}g_{3}\right)}^{y},v^{y}{\left(g_{2}g_{3}\right)}^{y\sigma_{1}},\\ u_{0},v_{0},w_{0},w_{0}^{y_{0}}{\left(g_{2}g_{3}\right)}^{y_{0}\psi_{2}},g^{y_{0}}{\left(g_{2}g_{3}\right)}^{y_{0}},v_{0}^{y_{0}}{\left(g_{2}g_{3}\right)}^{y_{0}\sigma_{2}} \end{array} \end{array}$$ (2)

Every oracle is allowed to simulate the semi-functional ciphertexts, normal and semi-functional (H)IBE private keys according to the provided group elements in Eq 2. We define the oracles from *O*_0_ to *O*_4_ in which the simulators will be allowed to produce a normal challenge decryption key. The outputs of Oracle *O*_0_ will allow a simulator to produce a semi-functional challenge ciphertext, a normal challenge (H)IBE private key. The outputs of Oracle *O*_1_ will allow a simulator to produce a semi-functional challenge ciphertext, a type-2 ephemeral semi-functional (ESF-2) challenge HIBE private key and a normal challenge IBE private key. The outputs of Oracle *O*_1^+^_ will allow a simulator to produce a semi-functional challenge ciphertext, an type-2 ephemeral semi-functional (ESF-2) challenge IBE private key an normal challenge HIBE private key. The outputs of Oracle *O*_3_ will allow a simulator to produce a type-1 ephemeral semi-functional(ESF-1) ciphertext, and a type-2 ephemeral semi-functional(ESF-2) challenge (H)IBE private key. Finally, the outputs of Oracle *O*_4_ will allow a simulator to produce a semi-functional challenge ciphertext, and a semi-functional challenge (H)IBE private key.

We define the oracles from *O*_5_ to *O*_7_ in which the simulators will be allowed to produce a semi-functional challenge (H)IBE key. The outputs of Oracle *O*_5_ will allow a simulator to produce a semi-functional ciphertext, and an ephemeral semi-functional challenge decryption key. The outputs of Oracle *O*_6_ will allow a simulator to produce an type-1 ephemeral semi-functional(ESF-1) ciphertext, and a type-2 ephemeral semi-functional(ESF-2) challenge decryption key. Finally, the outputs of Oracle *O*_7_ will allow a simulator to produce a semi-functional ciphertext, and a semi-functional challenge decryption key.

**Oracle** *O*_0_ The first oracle, which we will denote by *O*_0_, responds to queries as follows. Upon receiving a challenge HIBE-key-type query for *I* ∈ *Z*_*n*_, it chooses *r*, *y*′ ∈ *Z*_*n*_ randomly and returns the group elements

$$\begin{array}{c} \left(w^{y^{\prime }},g^{y^{\prime }},v^{y^{\prime }}{\left(u^{I}h\right)}^{r},g^{r}\right) \end{array}$$ (3)

to the attacker. Upon receiving a challenge IBE-key-type query for *T* ∈ *Z*_*n*_, it chooses *r*′, *y*″ ∈ *Z*_*n*_ randomly and returns the group elements

$$\begin{array}{c} \left(w_{0}^{y^{\prime \prime }},g^{y^{\prime \prime }},v_{0}^{y^{\prime \prime }}{\left(u_{0}^{T}h_{0}\right)}^{r^{\prime }},g^{r^{\prime }}\right) \end{array}$$ (4)

to the attacker. Upon receiving a challenge decryption-key-type query for *I* ∈ *Z*_*n*_ and *T* ∈ *Z*_*n*_, it chooses *r*, *y*′, *r*′, *y*″ ∈ *Z*_*n*_ randomly and returns the group elements

$$\begin{array}{c} \left(w^{y^{\prime }},g^{y^{\prime }},v^{y^{\prime }}{\left(u^{I}h\right)}^{r},g^{r},w_{0}^{y^{\prime \prime }},g^{y^{\prime \prime }},v_{0}^{y^{\prime \prime }}{\left(u_{0}^{T}h_{0}\right)}^{r^{\prime }},g^{r^{\prime }}\right) \end{array}$$ (5)

to the attacker. Upon receiving a ciphertext-type query for I* ∈ *Z*_*n*_, it chooses *t* ∈ *Z*_*n*_ randomly and returns the group elements

$$\begin{array}{c} \left(w^{s}g_{2}^{\delta_{1}}v^{t},g^{t},{\left(u^{I^{*}}h\right)}^{t}\right) \end{array}$$ (6)

to the attacker. Upon receiving a ciphertext-type query for T* ∈ *Z*_*n*_, it chooses *t*_0_ ∈ *Z*_*n*_ randomly and returns the group elements

$$\begin{array}{c} \left(w_{0}^{s}g_{2}^{\delta_{2}}v_{0}^{t_{0}},g^{t_{0}},{\left(u_{0}^{T^{*}}h_{0}\right)}^{t_{0}}\right) \end{array}$$ (7)

to the attacker.

**Oracle** *O*_1_ The next oracle, which we will denote by *O*_1_, responds to queries as follows. Upon receiving a challenge HIBE-key-type query for *I* ∈ *Z*_*n*_, it chooses *r*″, *y*‴ ∈ *Z*_*n*_ randomly, and also chooses *X*_2_, *Y*_2_ ∈ *G*_*p*_2__, *X*_3_, *Y*_3_ ∈ *G*_*p*_3__ randomly. It returns the group elements

$$\begin{array}{c} \left(w^{y^{\prime \prime \prime }},g^{y^{\prime \prime \prime }},v^{y^{\prime \prime \prime }}{\left(u^{I}h\right)}^{r^{\prime \prime }}X_{2}X_{3},g^{r^{\prime \prime }}Y_{2}Y_{3}\right) \end{array}$$ (8)

to the attacker. It responds to a ciphertext-type query, a challenge IBE-key-type query and a challenge decryption-key-type query in the same way as *O*_0_.

**Oracle** *O*_1^+^_ The oracle *O*_1^+^_ responds to queries as follows. Upon receiving a challenge IBE-key-type query for *T* ∈ *Z*_*n*_, it chooses *r*″, *y*‴ ∈ *Z*_*n*_ randomly, and also chooses *X*_2_, *Y*_2_ ∈ *G*_*p*_2__, *X*_3_, *Y*_3_ ∈ *G*_*p*_3__ randomly. It returns the group elements

$$\begin{array}{c} \left(w_{0}^{y^{\prime \prime \prime }},g^{y^{\prime \prime \prime }},v_{0}^{y^{\prime \prime \prime }}{\left(u_{0}^{T}h_{0}\right)}^{r^{\prime \prime }}X_{2}X_{3},g^{r^{\prime \prime }}Y_{2}Y_{3}\right) \end{array}$$ (9)

to the attacker. It responds to a ciphertext-type query, a challenge HIBE-key-type query and a challenge decryption-key-type query in the same way as *O*_0_.

**Oracle** *O*_2_ The next oracle, which we will denote by *O*_2_, responds to queries as follows. Upon receiving a challenge HIBE-key-type query and a challenge IBE-key-type query, it responds in the same way as *O*_1_. Upon receiving a ciphertext-type query for I* ∈ *Z*_*n*_, it chooses *t* ∈ *Z*_*n*_ randomly and returns the group elements

$$\begin{array}{c} \left(w^{s}g_{2}^{\delta_{1}}v^{t}g_{2}^{\sigma_{1}t},g^{t}g_{2}^{t},{\left(u^{I^{*}}h\right)}^{t}g_{2}^{t(a^{\prime }I^{*}+b^{\prime })}\right) \end{array}$$ (10)

to the attacker. Upon receiving a ciphertext-type query for T* ∈ *Z*_*n*_, it chooses *t*_0_ ∈ *Z*_*n*_ randomly and returns the group elements

$$\begin{array}{c} \left(w_{0}^{s}g_{2}^{\delta_{2}}v_{0}^{t_{0}}g_{2}^{\sigma_{2}t_{0}},g^{t_{0}}g_{2}^{t_{0}},{\left(u_{0}^{T^{*}}h_{0}\right)}^{t_{0}}g_{2}^{t_{0}\left(a^{\prime }T^{*}+b^{\prime }\right)}\right) \end{array}$$ (11)

to the attacker. It responds to a challenge decryption-key-type query in the same way as *O*_0_.

**Oracle** *O*_2^+^_ The next oracle, which we will denote by *O*_2^+^_, responds to queries as follows. Upon receiving a challenge HIBE-key-type query and a challenge IBE-key-type query, it responds in the same way as *O*_1^+^_. Upon receiving a ciphertext-type query for I* ∈ *Z*_*n*_, it chooses *t* ∈ *Z*_*n*_ randomly and returns the group elements

$$\begin{array}{c} \left(w^{s}g_{2}^{\delta_{1}}v^{t}g_{2}^{\sigma_{1}t},g^{t}g_{2}^{t},{\left(u^{I^{*}}h\right)}^{t}g_{2}^{t(a^{\prime }I^{*}+b^{\prime })}\right) \end{array}$$ (12)

to the attacker. Upon receiving a ciphertext-type query for T* ∈ *Z*_*n*_, it chooses *t*_0_ ∈ *Z*_*n*_ randomly and returns the group elements

$$\begin{array}{c} \left(w_{0}^{s}g_{2}^{\delta_{2}}v_{0}^{t_{0}}g_{2}^{\sigma_{2}t_{0}},g^{t_{0}}g_{2}^{t_{0}},{\left(u_{0}^{T^{*}}h_{0}\right)}^{t_{0}}g_{2}^{t_{0}\left(a^{\prime }T^{*}+b^{\prime }\right)}\right) \end{array}$$ (13)

to the attacker. It responds to a challenge decryption-key-type query in the same way as *O*_0_.

**Oracle** *O*_3_ The next oracle, which we will denote by *O*_3_, responds to queries as follows. Upon receiving a challenge HIBE-key-type query and a ciphertext-type query, it responds in the same way as *O*_2_. Upon receiving a challenge IBE-key-type query for *I* ∈ *Z*_*n*_, it chooses *r*″, *y*‴ ∈ *Z*_*n*_ randomly, and also chooses *X*_2_, *Y*_2_ ∈ *G*_*p*_2__, *X*_3_, *Y*_3_ ∈ *G*_*p*_3__ randomly. It returns the group elements

$$\begin{array}{c} \left(w_{0}^{y^{\prime \prime \prime }},g^{y^{\prime \prime \prime }},v_{0}^{y^{\prime \prime \prime }}{\left(u_{0}^{T}h_{0}\right)}^{r^{\prime \prime }}X_{2}X_{3},g^{r^{\prime \prime }}Y_{2}Y_{3}\right) \end{array}$$ (14)

to the attacker. It responds to a challenge decryption-key-type query in the same way as *O*_0_.

**Oracle** *O*_4_ The next oracle, which we will denote by *O*_4_, responds to ciphertext-type queries in the same way as *O*_0_, and responds to a challenge HIBE-key-type query for *I* ∈ *Z*_*n*_, by choosing *r*, *y*′ ∈ *Z*_*n*_ randomly and returns the group elements

$$\begin{array}{c} \left(w^{y^{\prime }}{\left(g_{2}g_{3}\right)}^{y^{\prime }\psi_{1}},g^{y^{\prime }}{\left(g_{2}g_{3}\right)}^{y^{\prime }},v^{y^{\prime }}{\left(g_{2}g_{3}\right)}^{y^{\prime }\sigma_{1}}{\left(u^{I}h\right)}^{r},g^{r}\right) \end{array}$$ (15)

to the attacker. Upon receiving a challenge IBE-key-type query for *T* ∈ *Z*_*n*_, it chooses *r*′, *y*″ ∈ *Z*_*n*_ randomly and returns the group elements

$$\begin{array}{c} \left(w_{0}^{y^{\prime \prime }}{\left(g_{2}g_{3}\right)}^{y^{\prime \prime }\psi_{2}},g^{y^{\prime \prime }}{\left(g_{2}g_{3}\right)}^{y^{\prime \prime }},v_{0}^{y^{\prime \prime }}{\left(g_{2}g_{3}\right)}^{y^{\prime \prime }\sigma_{2}}{\left(u_{0}^{T}h_{0}\right)}^{r^{\prime }},g^{r^{\prime }}\right) \end{array}$$ (16)

to the attacker. It responds to a challenge decryption-key-type query in the same way as *O*_0_.

**Oracle** *O*_5_ The next oracle, which we will denote by *O*_5_, responds to queries as follows. Upon receiving a challenge decryption-key-type query for *I*, *T* ∈ *Z*_*n*_, it chooses *r*, *y*′, *r*′, *y*″ ∈ *Z*_*n*_ randomly, and also chooses $X_{2},Y_{2},X_{2}^{\prime },Y_{2}^{\prime }\in G_{p_{2}},X_{3},Y_{3},X_{3}^{\prime },Y_{3}^{\prime }\in G_{p_{3}}$ randomly. It returns the group elements

$$\begin{array}{c} \left(w^{y^{\prime }},g^{y^{\prime }},v^{y^{\prime }}{\left(u^{I}h\right)}^{r}X_{2}X_{3},g^{r}Y_{2}Y_{3},w_{0}^{y^{\prime \prime }},g^{y^{\prime \prime }},v_{0}^{y^{\prime \prime }}{\left(u_{0}^{T}h_{0}\right)}^{r^{\prime }}X_{2}^{\prime }X_{3}^{\prime },g^{r^{\prime }}Y_{2}^{\prime }Y_{3}^{\prime }\right) \end{array}$$ (17)

to the attacker. It responds to a ciphertext-type query and a challenge (H)IBE-key-type query in the same way as *O*_4_.

**Oracle** *O*_6_ The next oracle, which we will denote by *O*_6_, responds to queries as follows. Upon receiving a ciphertext-type query for I* ∈ *Z*_*n*_, it chooses *t* ∈ *Z*_*n*_ randomly and returns the group elements

$$\begin{array}{c} \left(w^{s}g_{2}^{\delta_{1}}v^{t}g_{2}^{\sigma_{1}t},g^{t}g_{2}^{t},{\left(u^{I^{*}}h\right)}^{t}g_{2}^{t(a^{\prime }I^{*}+b^{\prime })}\right) \end{array}$$ (18)

to the attacker. Upon receiving a ciphertext-type query for T* ∈ *Z*_*n*_, it chooses *t*_0_ ∈ *Z*_*n*_ randomly and returns the group elements

$$\begin{array}{c} \left(w_{0}^{s}g_{2}^{\delta_{2}}v_{0}^{t_{0}}g_{2}^{\sigma_{2}t_{0}},g^{t_{0}}g_{2}^{t_{0}},{\left(u_{0}^{T^{*}}h_{0}\right)}^{t_{0}}g_{2}^{t_{0}\left(a^{\prime }T^{*}+b^{\prime }\right)}\right) \end{array}$$ (19)

to the attacker. It responds to a decryption-type query and a challenge (H)IBE-key-type query in the same way as *O*_5_.

**Oracle** *O*_7_ The last oracle, which we will denote by *O*_7_, responds to ciphertext-type queries in the same way as *O*_0_, and responds to a challenge decryption-key-type query for *I*, *T* ∈ *Z*_*n*_, by choosing *r*, *y*′, *r*′, *y*″ ∈ *Z*_*n*_ randomly and returns the group elements

$$\begin{array}{c} \begin{array}{c} (w^{y^{\prime }}{\left(g_{2}g_{3}\right)}^{y^{\prime }\psi_{1}},g^{y^{\prime }}{\left(g_{2}g_{3}\right)}^{y^{\prime }},v^{y^{\prime }}{\left(g_{2}g_{3}\right)}^{y^{\prime }\sigma_{1}}{\left(u^{I}h\right)}^{r},g^{r},\\ w_{0}^{y^{\prime \prime }}{\left(g_{2}g_{3}\right)}^{y^{\prime \prime }\psi_{2}},g^{y^{\prime \prime }}{\left(g_{2}g_{3}\right)}^{y^{\prime \prime }},v_{0}^{y^{\prime \prime }}{\left(g_{2}g_{3}\right)}^{y^{\prime \prime }\sigma_{2}}{\left(u_{0}^{T}h_{0}\right)}^{r^{\prime }},g^{r^{\prime }}) \end{array} \end{array}$$

to the attacker. It responds to a challenge (H)IBE-key-type query in the same way as *O*_6_.

We define the advantage of an attacker A in distinguishing between *O*_*i*_ and *O*_*j*_ to be $|Pr\left[A\left(O_{i}\right)=1\right]-Pr\left[A\left(O_{j}\right)=1\right]|$. Here, we assume that A interacts with either *O*_*i*_ or *O*_*j*_, and then outputs a bit 0 or 1 encoding its guess of which oracle it interacted with.

### 4.4 Indistinguishability of *G*_*C*′_ and *G*_*SF*″_

#### 4.4.1 Strategy for the indistinguishability of *G*_*C*′_ and *G*_*SF*″_

For the proof of the indistinguishability of *G*_*C*′_ and *G*_*SF*′_, we cannot use the simple nested dual system in U-HIBE [11] that change a normal private key(or normal update key) to an ephemeral semi-fuctional private key(or semi-functional update key) one by one since the adversary of RHIBE can query a private key for *ID*|_*k*_ ∈ *Prefix*(ID*|_*l*_) and an update key for T*.

To solve this problem, we firstly use a modular design strategy like [13] and construct the private keys and update keys from smaller component keys. A secret key *SK*_*ID*|_*k*__ consists of many HIBE private keys which are represented as {*SK*_*HIBE*,*S*_*θ*__}_*S*_*θ*_∈*Path*_ and an update key *UK*_*ID*|_*k*−1_,*T*,*R*_ consists a randomized decryption key *RSK*_*HIBE*_ and many IBE private keys {*SK*_*IBE*,*S*_*i*__}_*S*_*i*_∈*CV*_*R*__ where each HIBE private key (or an IBE private key) is associated with a node *S*_*j*_ in *BT*_*ID*|_*k*−1__. The HIBE and IBE private keys can be grouped together if they are related to the same node *S*_*j*_ in *BT*_*ID*|_*k*−1__ and a correct decryption key is constructed form the grouped (H)IBE private key.

To uniquely identify a node *S*_*j*_ ∈ *BT*_*ID*|_*k*−1__, we define a node identifier *NID* of this node as a string *ID*|_*k*−1_||*L*_*j*_ where *L*_*j*_ = *Label*(*v*_*j*_). To prove the indistinguishability of *G*_*C*′_ and *G*_*SF*″_, we change normal HIBE private keys and normal IBE private keys that are related to the same node identifier *NID* into (ephemeral) semi-functional keys by defining additional hybrid games. This additional hybrid games are performed for all node identifiers that are used in the key queries of the adversary.

**Fig 1:**
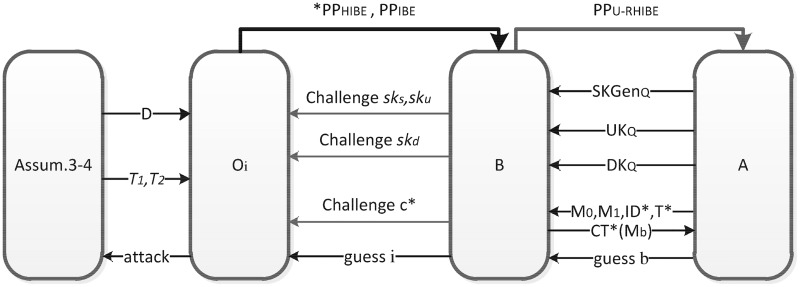
The query process in the proof of the indistinguishability of *G*_*C*′_ and *G*_*SF*′_. *The group elements that the oracle simulator gives to the challenger B are not only the public parameters *PP*_*HIBE*_ and *PP*_*IBE*_, but also the group elements for constructing the (ephemeral) semi-functional keys and ciphertexts and the public elements given by the assumptions.

Secondly, we give the equivalent model in which the challenger B answers the secret(update, and decryption) key queries of the adversery A by requesting the associated (H)IBE private keys from an oracle simulator O, shown in Fig 1. When the adversary A queries B for the secret key, update key or decryption key for some identity and some time period, *B* constructs the key by the (H)IBE-challenge-key or decryption-challenge-key it queries from the oracle simulator O. O adaptively answers B the corresponding group elements which it constructs by using the public paremeters given by some complexity assumption. Therefore, under the complexity assumptions, the oracle *O*_*i*_ that O chooses to answer B is indistinguishable and consequently the adversary A cannot distinguish whether A is playing the real RHIBE game or other variation games based on all the answers A recieves after the adaptive queries to B.

For additional hybrid games that change HIBE private keys (or IBE private keys) that are related to the same node identifier *NID* = *ID*|_*k*−1_||*L*_*j*_ from normal keys to semi-functional keys, we need to define an index pair (*i*_*n*_, *i*_*c*_) for an HIBE private key (or an IBE private key) that is related to the node *v*_*j*_ ∈ *BT*_*ID*|_*k*−1__ where *i*_*n*_ is a node index and *i*_*c*_ is a counter index. Suppose that an HIBE private key (or an IBE private key) is related to a node *NID*. The node index *i*_*n*_ for the HIBE private key (or the IBE private key) is assigned as follows: If the node *v*_*j*_ ∈ *BT*_*ID*|_*k*−1__ with a node identifier *NID* appears first time in key queries, then we set in as the number of distinct node identifiers in previous key queries plus one. If the node identifier *NID* already appeared before in key queries, then we set *i*_*n*_ as the value $i_{n}^{\prime }$ of previous HIBE private key (or IBE private key) with the same node identifier. The counter index *i*_*c*_ of an HIBE private key is assigned as follows: If the node identifier NID appears first time in HIBE private key queries, then we set *i*_*c*_ as one. If the node identifier NID appeared before in HIBE private key queries, then we set *i*_*c*_ as the number of HIBE private keys with the same node identifier that appeared before plus one. Similarly, we assigns the counter index *i*_*c*_ of an IBE private key.

Thirdly, we divide the behavior of an adversary as two types: Type-1 and Type-2. We next show that the semi-functional key invariance property holds for two types of the adversary. Let $ID_{l}^{*}$ be the challenge hierarchical identity and T* be the challenge time. For a challenge node *v* with the node index *h* in the hybrid games from *Game*_*C*_ and *Game*_*SF*_, the adversary types are formally defined as follows:

1. **Type-1**: An adversary is Type-1 if it queries on a hierarchical identity ${ID|}_{k}\notin Prefix\left(ID_{l}^{*}\right)$ for all HIBE private keys with the node index *h*, and it queries on time *T* = T* for at least one IBE private key with the node index *h*.
2. **Type-2**: An adversary is Type-2 if it queries on time *T* ∉ T* for all IBE private keys with the node index *h*. Note that it may query on a hierarchical identity *ID*|_*k*_ ∈ *Prefix*(ID*|_*l*_) for at least one HIBE private key with *h*, or it may query on a hierarchical identity ${ID|}_{j}\notin Prefix\left(ID_{l}^{*}\right)$ for all HIBE private keys with *h*.

We prove our dual system encryption RHIBE scheme via a hybrid argument over the sequence of games in Table 3. For the different type of adversary, the squence of games is basicly the same except that:

1. For the Type-1 adversary, we prove the indistinguishability of *G*_*C*′_ and *G*_*ESF*′_ by the transition from *G*_*C*′_ to *G*_*EK*−*S*_, and to *G*_*ESF*′_ without the attacker’s advantage changing by a non-negligible amount.
2. For the Type-2 adversary, we prove the indistinguishability of *G*_*C*′_ and *G*_*ESF*′_ by the transition from *G*_*C*′_ to *G*_*EK*−*U*_, and to *G*_*ESF*′_ without the attacker’s advantage changing by a non-negligible amount.

**Theorem 2** *Under Assumptions 3 and 4*, *our dual system encryption RHIBE scheme has the equation*

$$\begin{array}{c} \begin{array}{c} |Adv_{A}^{G_{C^{\prime }}}\left(\lambda \right)-Adv_{A}^{G_{SF^{\prime \prime }}}\left(\lambda \right)|\leq \left(O\left(q_{n}logN_{max}\right)+O\left(q_{n}r_{max}logN_{max}\right)\right)\left(Adv_{B}^{A3}+Adv_{B}^{A4}\right)\\ +O\left(l\right)\left(Adv_{B}^{A3}+Adv_{B}^{A4}\right) \end{array} \end{array}$$ (20)

We will prove these indistinguishabilities between games *G*_*C*′_, *G*_*E*−*S*_ (or *G*_*E*−*U*_), *G*_*E*−*S*′_ (or *G*_*E*−*U*′_), *G*_*ESF*′_, and *G*_*SF*″_ by going through several intermediary oracles. The main properties of our oracles are summarized in Tables 4 and 5 for the Type-1 adversary and Table 6 for the Type-2 adversary respectively. We intend these tables to be used only as a quick reference guide, not as a definition. We give a complete proof for the Type-1 adversary, and a brief explanation of the proof for the Type-2 adversary is demonstrated then.

**Table 4:** Simulation of challenge keys and cipertext in oracles for the proof of the indistinguishability between *G*_*C*′_ and *G*_*SF*″_ under Type-1 adversary.

Oracle	CT-Type Response	SK-Type Response	UK-Type Response	DK-Type Response
***O***_**0**_	SF	Normal	Normal	Normal
*O*_1/2_	SF	ESF-1	Normal	Normal
***O***_**1**_	SF	ESF-2	Normal	Normal
$O_{i}^{*}$	ESF-2^*i*^	ESF-2	Normal	Normal
$O_{i}^{\prime }$	ESF-3^*i*^	ESF-2	Normal	Normal
$O_{i}^{\prime \prime }$	ESF-4^*i*^	ESF-2	Normal	Normal
***O***_**2**_	ESF-1	ESF-2	Normal	Normal
*O*_5/2_	ESF-1	ESF-2	ESF-1	Normal
***O***_**3**_	ESF-1	ESF-2	ESF-2	Normal
*O*_3.1_	ESF-1	ESF-3	ESF-2	Normal
*O*_3.2_	ESF-1	ESF-3	ESF-3	Normal
†*O*_3.3_	ESF-5	ESF-3	ESF-3	Normal
†*O*_3.4_	ESF-5	ESF-4	ESF-3	Normal
†*O*_3.5_	ESF-5	ESF-4	ESF-4	Normal
*O*_3.6_	ESF-1	ESF-4	ESF-4	Normal
*O*_7/2′_	ESF-1	ESF-4	ESF-5	Normal
$\tilde{O}{}_{i}^{*}$	ESF-2^*i*^	ESF-4	SF	Normal
$\tilde{O}{}_{i}^{\prime }$	ESF-3^*i*^	ESF-4	SF	Normal
$\tilde{O}{}_{i}^{\prime \prime }$	ESF-4^*i*^	ESF-4	SF	Normal
*O*_7/2_	SF	ESF-5	SF	Normal
***O***_**4**_	SF	SF	SF	Normal

Note: oracles marked with † initialize with an extra *G*_*p*_3__ term on $g^{s}g_{2}^{\gamma }$.

**Table 5:** Defination of games between *G*_*ESF*′_ and *G*_*SF*″_.

Games	Oracles	Keys in Queries			Challenge Ciphertext			
		SK	UK	DK	*Normal*	*SF*	*ESF* − 1	*ESF* − 5
*G*_*ESF*′_	*O*_3_	*ESF* − 2	*ESF* − 2	*Normal*			◯	
*G*_*ESF*′−1_	*O*_3.1_	*ESF* − 3	*ESF* − 2	*Normal*			◯	
*G*_*ESF*′−2_	*O*_3.2_	*ESF* − 3	*ESF* − 3	*Normal*			◯	
*G*_*ESF*′−3_	*O*_3.3_	*ESF* − 3	*ESF* − 3	*Normal*				◯
*G*_*ESF*′−4_	*O*_3.4_	*ESF* − 4	*ESF* − 3	*Normal*				◯
*G*_*ESF*′−5_	*O*_3.5_	*ESF* − 4	*ESF* − 4	*Normal*				◯
*G*_*ESF*′−6_	*O*_3.6_	*ESF* − 4	*ESF* − 4	*Normal*			◯	
*G*_*ESF*′−7_	${\tilde{O}}_{q_{c}}^{*}$	*ESF* − 4	*SF*	*Normal*			◯	
*G*_*ESF*′−8_	${\tilde{O}}_{0}^{*}$	*ESF* − 4	*SF*	*Normal*		◯		
*G*_*SF*″_	*O*_4_	*SF*	*SF*	*Normal*		◯		

**Table 6:** Simulation of challenge keys and cipertext in oracles under Type-2 adversary for the proof of the indistinguishability between *G*_*C*′_ and *G*_*SF*″_.

Oracle	CT-Type Response	SK-Type Response	UK-Type Response	DK-Type Response
***O***_**0**_	SF	Normal	Normal	Normal
*O*_1/2^+^_	SF	Normal	ESF-1	Normal
***O***_**1**^+^_	SF	Normal	ESF-2	Normal
$O_{i^{+}}^{*}$	ESF-2^*i*^	Normal	ESF-2	Normal
$O_{i^{+}}^{\prime }$	ESF-3^*i*^	Normal	ESF-2	Normal
$O_{i^{+}}^{\prime \prime }$	ESF-4^*i*^	Normal	ESF-2	Normal
***O***_**2**^+^_	ESF-1	Normal	ESF-2	Normal
*O*_5/2^+^_	ESF-1	ESF-1	ESF-2	Normal
***O***_**3**_	ESF-1	ESF-2	ESF-2	Normal
*O*_3.1^+^_	ESF-1	ESF-2	ESF-3	Normal
*O*_3.2_	ESF-1	ESF-3	ESF-3	Normal
†*O*_3.3_	ESF-5	ESF-3	ESF-3	Normal
†*O*_3.4^+^_	ESF-5	ESF-3	ESF-4	Normal
†*O*_3.5_	ESF-5	ESF-4	ESF-4	Normal
*O*_3.6_	ESF-1	ESF-4	ESF-4	Normal
*O*_3.7^+^_	ESF-1	ESF-5	ESF-4	Normal
$\tilde{O}{}_{i^{+}}^{*}$	ESF-2^*i*^	SF	ESF-4	Normal
$\tilde{O}{}_{i^{+}}^{\prime }$	ESF-3^*i*^	SF	ESF-4	Normal
$\tilde{O}{}_{i^{+}}^{\prime \prime }$	ESF-4^*i*^	SF	ESF-4	Normal
*O*_7/2^+^_	SF	SF	ESF-5	Normal
***O***_**4**_	SF	SF	SF	Normal

Note: oracles marked with † initialize with an extra *G*_*p*_3__ term on $g^{s}g_{2}^{\gamma }$.

#### 4.4.2 Type-1 adversary

As defined before, the Type-1 adversary is restricted to queries on a hierarchical identity ${ID|}_{k}\notin Prefix\left(ID_{l}^{*}\right)$. By quering for all HIBE private keys with any node index *h* where the node is on the path from the root to the leaf node *v*_*ID*|_*k*__ in the tree *BT*_*ID*|_*k*−1__, the adversary derives the secret key of *ID*|_*k*_.

So we could show an information theoretic argument for the HIBE private keys from normal to ephemeral semi-functional HIBE keys, then to semi-functional HIBE keys. At the meanwhile, by adaptively transforming the types of IBE private keys sooner or later than the transformation of HIBE private keys, we avoid a potential paradox for the update keys.

From the flollowing Lemma 1, to Lemma 20, we obtain the advantage of Type-1 adversary to distinguish between *G*_*C*′_ and *G*_*SF*″_ under Type-1 adversary as

$$\begin{array}{c} \begin{array}{c} Adv_{A}^{G_{C^{\prime }}}-Adv_{A}^{G_{SF^{\prime \prime }}}\leq |Adv_{A}^{G_{C^{\prime }}}-Adv_{A}^{G_{E-S}}|+|Adv_{A}^{G_{E-S}}-Adv_{A}^{G_{E-S^{\prime }}}|\\ \;+|Adv_{A}^{G_{E-S^{\prime }}}-Adv_{A}^{G_{ESF^{\prime }}}|+|Adv_{A}^{G_{ESF^{\prime }}}-Adv_{A}^{G_{SF^{\prime \prime }}}|\\ \;\leq \left(O\left(q_{n}\left(q_{s}+q_{e}\right)\right)+O\left(l\right)\right)\left(Adv_{B}^{A3}+Adv_{B}^{A4}\right)\\ \;\leq \left(O\left(q_{n}logN_{max}+q_{n}r_{max}logN_{max}\right)+O\left(l\right)\right)\left(Adv_{B}^{A3}+Adv_{B}^{A4}\right) \end{array} \end{array}$$ (21)

We give the proof of those lemmas in Appendix.B.

**(1) Indistinguishability of** *G*_*C*′_ **and** *G*_*E*−*S*_

For the security proof of the indistinguishability of *G*_*C*′_ and *G*_*E*−*S*_, we define a sequence of additional hybrid games *G*_*C*′,1_, …, *G*_*C*′,*h*_, …, *G*_*C*′,*q*_*n*__, where *G*_*C*′_ = *G*_*C*′,0_ and *q*_*n*_ is the number of all node identifiers that are used in HIBE private keys and IBE private keys of an adversary. In the game *G*_*C*′,*h*_ for 1 ≤ *h* ≤ *q*_*n*_, the challenge ciphertext is semi-functional, all IBE private keys are normal, HIBE private keys with a node index *i*_*n*_ ≤ *h* are of ESF-2, the remaining HIBE private keys with a node index *i*_*n*_ > *h* are normal.

**Oracle** *O*_1/2_ This oracle initializes in the same way as *O*_0_, *O*_1_ and provides the attacker with initial group elements from the same distribution. Upon receiving a challenge HIBE-key-type query for *I* ∈ *Z*_*n*_, it chooses *r*′, *y*′ ∈ *Z*_*n*_ randomly, and also chooses *X*_3_, *Y*_3_ ∈ *G*_*p*_3__ randomly. It returns the group elements

$$\begin{array}{c} \left(w^{y^{\prime }},g^{y^{\prime }},v^{y^{\prime }}{\left(u^{I}h\right)}^{r^{\prime }}X_{3},g^{r^{\prime }}Y_{3}\right) \end{array}$$ (22)

to the attacker. It responds to a ciphertext-type query or a challenge IBE-key-type query in the same way as *O*_0_.

We define hybrid games *H*_1,1_, *H*_1,2_, ⋯, *H*_*h*_*c*_,1_, *H*_*h*_*c*_,2_, …, *H*_*q*_*s*_,1_, *H*_*q*_*s*_,2_ where *H*_0,2_ = *G*_*C*′,*h*_ and *H*_*q*_*s*_,2_ = *G*_*C*′,*h*+1_, and *q*_*s*_ is the maximun number of HIBE private key queries for the node index *h*. The games are formally defined as follows:

**Game** *H*_*h*_*c*_,1_ This game *H*_*h*_*c*_,1_ for 1 ≤ *h*_*c*_ ≤ *q*_*s*_ is almost the same as *G*_2,*h*−1_ except the generation of HIBE private keys and IBE private keys with the node *θ*_*h*_ of the index *h*. An IBE private key with an index pair (*h*, *i*_*c*_) is generated as normal. An HIBE private key with an index pair (*h*, *i*_*c*_) is generated as follows:

1. *i*_*c*_ < *h*_*c*_: It generates a ESF-2 *SK*_*HIBE*,*θ*_*h*__.
2. *i*_*c*_ = *h*_*c*_: It generates a ESF-1 *SK*_*HIBE*,*θ*_*h*__ by using the element groups in Eq 22.
3. *i*_*c*_ > *h*_*c*_: It simply generates a normal HIBE private key.

**Game** *H*_*h*_*c*_,2_ This game *H*_*h*_*c*_,1_ for 1 ≤ *h*_*c*_ ≤ *q*_*s*_ is almost the same as *H*_*h*_*c*_,1_ except the generation of HIBE private key with an index pair (*h*, *i*_*c*_) and *i*_*c*_ = *h*_*c*_ is generated as a ESF-2 *SK*_*HIBE*,*θ*_*h*__ by using the element groups in Eq 8.

**Lemma 1** *Under Assumptions 3*, *no PPT attacker can distinguish between O*_0_ *and O*_1/2_ *with non-negligible advantage*. *So no PPT attacker can distinguish between H*_*h*_*c*_−1,2_ *and H*_*h*_*c*_,1_ *with non-negligible advantage*.

**Lemma 2** *Under Assumptions 4*, *no PPT attacker can distinguish between O*_1/2_ *and O*_1_ *with non-negligible advantage*. *So no PPT attacker can distinguish between H*_*h*_*c*_,1_ *and H*_*h*_*c*_,2_ *with non-negligible advantage*.

Let $Adv_{A}^{G_{C^{\prime },h}}$ be the advantage of A in a game *G*_*C*′,*h*_. From the Lemma 1, 2, we obtain the following equation

$$\begin{array}{c} \begin{array}{c} Adv_{A}^{G_{C^{\prime },h-1}}-Adv_{A}^{G_{C^{\prime },h}}\leq \sum_{h_{c}=1}^{q_{s}}(|Adv_{A}^{H_{h_{c}-1,2}}-Adv_{A}^{H_{h_{c},1}}|+|Adv_{A}^{H_{h_{c},1}}-Adv_{A}^{H_{h_{c},2}}\left|\right)\\ \leq O\left(q_{s}\right)\left(Adv_{B}^{A3}+Adv_{B}^{A4}\right) \end{array} \end{array}$$

So we obtain the following equation

$$\begin{array}{c} Adv_{A}^{G_{C^{\prime }}}-Adv_{A}^{G_{E-S}}\leq \sum_{h=1}^{q_{n}}\left(Adv_{A}^{G_{C^{\prime },h-1}}-Adv_{A}^{G_{C^{\prime },h}}\right)\leq O\left(q_{n}q_{s}\right)\left(Adv_{B}^{A3}+Adv_{B}^{A4}\right) \end{array}$$ (23)

**(2) Indistinguishability of** *G*_*E*−*S*_ **and** *G*_*E*−*S*′_

We now prove the indistinguishability of *G*_*E*−*S*_ and *G*_*E*−*S*′_ in a hybrid argument using polynomially many steps. We let *q*_*c*_ denote the number of ciphertext-type queries made by a PPT attacker A. Firstly we define hybrid games *S*_−1,1_, *S*_0,2_, *S*_0,3_, *S*_0,1_, *S*_1,2_, *S*_1,3_, *S*_1,1_⋯, *S*_*k*,2_, *S*_*k*,3_, *S*_*k*,1_, …, *S*_*q*_*c*_−1,2_, *S*_*q*_*c*_−1,3_, *S*_*q*_*c*_−1,1_, where *S*_−1,1_ = *G*_*E*−*S*_ and *S*_*q*_*c*_−1,1_ = *G*_*E*−*S*′_. The games are formally defined as follows:

**Game** *S*_*k*,1_ This game *S*_*k*,1_ for 0 ≤ *k* ≤ *q*_*c*_ is almost the same as *G*_*E*−*S*_ except the generation of the challenge ciphertext. The challenge ciphertext of $\left(T^{*},I_{1}^{*},\ldots ,I_{q_{c}-1}^{*}\right)$ is generated as EST-2^k^-CT outputed by **EncryptESF-2**^k^ defined in AppendixA.

**Game** *S*_*k*,2_ This game *S*_*k*,2_ for 0 ≤ *k* ≤ *q*_*c*_ − 1 is almost the same as *G*_*E*−*S*_ except the generation of the challenge ciphertext. The challenge ciphertext of $\left(T^{*},I_{1}^{*},\ldots ,I_{q_{c}-1}^{*}\right)$ is generated as EST-3^k^-CT outputed by **EncryptESF-3**^k^ defined in AppendixA.

**Game** *S*_*k*,3_ This game *S*_*k*,3_ for 0 ≤ *k* ≤ *q*_*c*_ − 1 is almost the same as *G*_*E*−*S*_ except the generation of the challenge ciphertext. The challenge ciphertext of $\left(T^{*},I_{1}^{*},\ldots ,I_{q_{c}-1}^{*}\right)$ is generated as EST-4^k^-CT outputed by **EncryptESF-4**^k^ defined in AppendixA.

We will define additional oracles $O_{i}^{*}$ for each *i* from 0 to *q*_*c*_ − 1, $O_{i}^{\prime }$ for each *i* from 0 to *q*_*c*_ − 1, and $O_{i}^{\prime \prime }$ for each *i* from 0 to *q*_*c*_ − 1, to sample various distributions of group elements used for constructing the various types of ciphertexts in Game *S*_*k*,1_, Game *S*_*k*,2_ and Game *S*_*k*,3_.

**Oracle** $O_{i}^{*}$ This oracle initializes in the same way as *O*_1_, *O*_2_ and provides the attacker with initial group elements from the same distribution. It also responds to challenge key-type queries in the same way as *O*_1_, *O*_2_. It keeps a counter of ciphertext-type queries which is initially equal to zero. It increments this counter after each response to a ciphertext-type query. In response to the *j*th ciphertext-type query for some $I_{j}^{*}$, if *j* ≤ *i*, it responds exactly like *O*_2_. If *j* > *i*, it responds exactly like *O*_1_. In particular, $O_{0}^{*}$ is identical to *O*_1_ and $O_{q}^{*}$ is identical to *O*_2_.

**Oracle** $O_{i}^{\prime }$ This oracle acts the same as $O_{i}^{*}$ except in its response to the *i*^*th*^ ciphertext-type query. For the *i*^*th*^ ciphertext-type query for identity I*, it chooses a random *t* ∈ *Z*_*N*_ and random elements *X*_3_, *Y*_3_ ∈ *G*_*p*_3__ and responds with:

$$\begin{array}{c} \left(w^{s}g_{2}^{\delta_{1}}v^{t}X_{3}^{\sigma_{1}},g^{t}X_{3},{\left(u^{I_{j}^{*}}h\right)}^{t}Y_{3}\right) \end{array}$$ (24)

If *i* = 0, the *i*^*th*^ ciphertext-type query is for time T*. It chooses a random *t*_0_ ∈ *Z*_*N*_ and random elements $X_{3}^{\prime },Y_{3}^{\prime }\in G_{p_{3}}$ and responds with:

$$\begin{array}{c} \left(w_{0}^{s}g_{2}^{\delta_{2}}v_{0}^{t_{0}}{X_{3}^{\prime }}^{\sigma_{2}},g^{t_{0}}X_{3}^{\prime },{\left(u_{0}^{T^{*}}h_{0}\right)}^{t_{0}}Y_{3}^{\prime }\right) \end{array}$$ (25)

**Oracle** $O_{i}^{\prime \prime }$ This oracle acts the same as $O_{i}^{*}$ except in its response to the *i*^*th*^ ciphertext-type query. For the *i*^*th*^ ciphertext-type query for identity I*, it chooses a random *t* ∈ *Z*_*N*_ and random elements *X*_3_, *Y*_3_ ∈ *G*_*p*_3__ and responds with:

$$\begin{array}{c} \left(w^{s}g_{2}^{\delta_{1}}v^{t}X_{3}^{\sigma_{1}},g^{t}g_{2}^{t}X_{3},{\left(u^{I_{j}^{*}}h\right)}^{t}g_{2}^{t(a^{\prime }I_{j}^{*}+b^{\prime })}Y_{3}\right) \end{array}$$ (26)

If *i* = 0, the *i*^*th*^ ciphertext-type query is for time T*. It chooses a random *t*_0_ ∈ *Z*_*N*_ and random elements $X_{3}^{\prime },Y_{3}^{\prime }\in G_{p_{3}}$ and responds with:

$$\begin{array}{c} \left(w_{0}^{s}g_{2}^{\delta_{2}}v_{0}^{t_{0}}{X_{3}^{\prime }}^{\sigma_{2}},g^{t_{0}}g_{2}^{t_{0}}X_{3}^{\prime },{\left(u_{0}^{T^{*}}h_{0}\right)}^{t_{0}}g_{2}^{t_{0}\left(a^{\prime }I_{j}^{*}+b^{\prime }\right)}Y_{3}^{\prime }\right) \end{array}$$ (27)

**Lemma 3** *Under Assumptions 3*, *no PPT attacker can distinguish between* $O_{k-1}^{*}$ *and* $O_{k}^{\prime }$ *with non-negligible advantage*. *So no PPT attacker can distinguish between S*_*k*−1,1_ *and S*_*k*,2_ *with non-negligible advantage*.

**Lemma 4** *Under Assumptions 4*, *no PPT attacker can distinguish between* $O_{k}^{\prime }$ *and* $O_{k}^{\prime \prime }$ *with non-negligible advantage*. *So no PPT attacker can distinguish between S*_*k*,2_ *and S*_*k*,3_ *with non-negligible advantage*.

**Lemma 5** *Under Assumptions 3*, *no PPT attacker can distinguish between* $O_{k}^{\prime \prime }$ *and* $O_{k}^{*}$ *with non-negligible advantage*. *So no PPT attacker can distinguish between S*_*k*,3_ *and S*_*k*,1_ *with non-negligible advantage*.

Let $Adv_{A}^{S_{k,1}}$, $Adv_{A}^{S_{k,2}}$ and $Adv_{A}^{S_{k,3}}$ be the advantage of A in the games *S*_*k*,1_, *S*_*k*,2_ and *S*_*k*,3_. From the Lemma 3, 4, 5, we obtain the following equation

$$\begin{array}{c} \begin{array}{c} Adv_{A}^{G_{E-S}}-Adv_{A}^{G_{E-S^{\prime }}}\leq \sum_{k=0}^{q_{c}-1}(|Adv_{A}^{S_{k-1,1}}-Adv_{A}^{S_{k,2}}|+|Adv_{A}^{S_{k,2}}-Adv_{A}^{S_{k,3}}|\\ +|Adv_{A}^{S_{k,3}}-Adv_{A}^{S_{k,1}}\left|\right)\\ \leq q_{c}\left(2Adv_{B}^{A3}+Adv_{B}^{A4}\right) \end{array} \end{array}$$ (28)

**(3) Indistinguishability of** *G*_*E*−*S*′_ **and** *G*_*ESF*′_

For the security proof of the indistinguishability of *G*_*E*−*S*′_ and *G*_*ESF*′_, we define a sequence of additional hybrid games *G*_*S*′,1_, …, *G*_*S*′,*h*_, …, *G*_*S*′,*q*_*n*__, where *G*_*E*−*S*′_ = *G*_*S*′,0_ and *q*_*n*_ is the number of all node identifiers that are used in HIBE private keys and IBE private keys of an adversary. In the game *G*_*S*′,*h*_ for 1 ≤ *h* ≤ *q*_*n*_, the challenge ciphertext is semi-functional, all IBE private keys are ESF-2, IBE private keys with a node index *i*_*n*_ ≤ *h* are of ESF-2, the remaining HIBE private keys with a node index *i*_*n*_ > *h* are normal.

**Oracle** *O*_5/2_ This oracle initializes in the same way as *O*_2_, *O*_3_ and provides the attacker with initial group elements from the same distribution. Upon receiving a challenge IBE-key-type query for *T* ∈ *Z*_*n*_, it chooses *r*′, *y*′ ∈ *Z*_*n*_ randomly, and also chooses *X*_3_, *Y*_3_ ∈ *G*_*p*_3__ randomly. It returns the group elements

$$\begin{array}{c} \left(w_{0}^{y^{\prime }},g^{y^{\prime }},v_{0}^{y^{\prime }}{\left(u_{0}^{T}h_{0}\right)}^{r^{\prime }}X_{3},g^{r^{\prime }}Y_{3}\right) \end{array}$$ (29)

to the attacker. It responds to a ciphertext-type query or a challenge HIBE-key-type query in the same way as *O*_2_.

We define hybrid games *E*_1,1_, *E*_1,2_, ⋯, *E*_*h*_*c*_,1_, *H*_*h*_*c*_,2_, …, *E*_*q*_*s*_,1_, *E*_*q*_*s*_,2_ where *E*_0,2_ = *G*_*S*′,*h*_ and *E*_*q*_*e*_,2_ = *G*_*S*′,*h*+1_, and *q*_*e*_ is the maximun number of IBE private key queries for the node index *h*. The games are formally defined as follows:

**Game** *E*_*h*_*c*_,1_ This game *E*_*h*_*c*_,1_ for 1 ≤ *h*_*c*_ ≤ *q*_*e*_ is almost the same as *G*_*S*′,*h*_ except the generation of HIBE private keys and IBE private keys with the node index *h*. An HIBE private key with an index pair (*h*, *i*_*c*_) is generated as ESF-2. An IBE private key with an index pair (*h*, *i*_*c*_) is generated as follows:

1. *i*_*c*_ < *h*_*c*_: It generates a normal $SK_{IBE,h}^{\prime }$ and converts the key to a ESF-2 *SK*_*IBE*,*h*_.
2. *i*_*c*_ = *h*_*c*_: It generates a normal $SK_{IBE,h}^{\prime }$ and converts the key to a ESF-1 *SK*_*IBE*,*h*_ by using the element groups in Eq 29.
3. *i*_*c*_ > *h*_*c*_: It simply generates a normal IBE private key.

**Game** *E*_*h*_*c*_,2_ This game *E*_*h*_*c*_,2_ for 1 ≤ *h*_*c*_ ≤ *q*_*e*_ is almost the same as *E*_*h*_*c*_,1_ except the generation of IBE private key with an index pair (*h*, *i*_*c*_) and *i*_*c*_ = *h*_*c*_ is generated as a ESF-2 *SK*_*IBE*,*h*_ by using the element groups in Eq 14.

**Lemma 6** *Under Assumptions 3*, *no PPT attacker can distinguish between O*_2_ *and O*_5/2_ *with non-negligible advantage*. *So no PPT attacker can distinguish between E*_*h*_*c*_−1,2_ *and E*_*h*_*c*_,1_ *with non-negligible advantage*.

**Lemma 7** *Under Assumptions 4*, *no PPT attacker can distinguish between O*_5/2_ *and O*_3_ *with non-negligible advantage*. *So no PPT attacker can distinguish between E*_*h*_*c*_,1_ *and E*_*h*_*c*_,2_ *with non-negligible advantage*.

Let $Adv_{A}^{G_{E^{\prime },h}}$ be the advantage of A in a game *G*_*E*′,*h*_. From the Lemma 6, 7, we obtain the following equation

$$\begin{array}{c} \begin{array}{c} Adv_{A}^{G_{E^{\prime },h-1}}-Adv_{A}^{G_{E^{\prime },h}}\leq \sum_{h_{c}=1}^{q_{e}}(|Adv_{A}^{E_{h_{c},1}}-Adv_{A}^{E_{h_{c},2}}|+|Adv_{A}^{E_{h_{c}-1,2}}-Adv_{A}^{E_{h_{c},1}}\left|\right)\\ \leq O\left(q_{e}\right)\left(Adv_{B}^{A3}+Adv_{B}^{A4}\right) \end{array} \end{array}$$

So we obtain the following equation

$$\begin{array}{c} Adv_{A}^{G_{E-S^{\prime }}}-Adv_{A}^{G_{ESF^{\prime }}}\leq \sum_{h=1}^{q_{n}}\left(Adv_{A}^{G_{E^{\prime },h-1}}-Adv_{A}^{G_{E^{\prime },h}}\right)\leq O\left(q_{n}q_{e}\right)\left(Adv_{B}^{A3}+Adv_{B}^{A4}\right) \end{array}$$ (30)

**(4) Indistinguishability of** *G*_*ESF*′_ **and** *G*_*SF*″_

For the security proof of the indistinguishability of *G*_*ESF*′_ and *G*_*SF*″_, we define a sequence of games *G*_*ESF*′−1_, ⋯, *G*_*ESF*′−5_ to change the type of secret keys and update keys from ESF-2 to ESF-4 and the type of ciphertexts from ESF-1 to ESF-5 and *G*_*ESF*′−6_, ⋯, *G*_*ESF*′−8_ to change the type of update keys to semi-functional and the type of ciphertexts back to semi-functional. In Table 5, we give the types of key in the queries and the challenge cipertext in every game, and the decryption situation according to the types of keys and ciphertexts.

**G**_**ESF′−1**_: The secret keys are changed to ESF-3. The update keys are still ESF-2. The challenge ciphertext is still ESF-1. The decryption keys are still normal.

**G**_**ESF′−2**_: The update keys are changed to ESF-3. The secret keys are ESF-3. The challenge ciphertext is still ESF-1. The decryption keys are still normal.

**G**_**ESF′−3**_: The challenge ciphertext is changed to ESF-5. The secret keys and the update keys are still ESF-3. The decryption keys are still normal.

**G**_**ESF′−4**_: The secret keys are changed to ESF-4. The update keys are still ESF-3. The challenge ciphertext is ESF-5. The decryption keys are still normal.

**G**_**ESF′−5**_: The update keys are changed to ESF-4. The secret keys are ESF-4. The challenge ciphertext is ESF-5. The decryption keys are still normal.

**G**_**ESF′−6**_: The challenge ciphertext is changed to ESF-1. The secret keys and the update keys are ESF-4. The decryption keys are still normal.

**G**_**ESF′−7**_: The update keys are changed to semi-functional update keys. The secret keys are ESF-4. The challenge ciphertext is ESF-1. The decryption keys are still normal.

**G**_**ESF′−8**_: The challenge ciphertext is changed to semi-functional. The secret keys are ESF-4. The update keys are semi-functional update keys. The decryption keys are still normal.

We firstly prove the indistinguishabilities between *G*_*ESF*′_ to *G*_*ESF*′−1_, *G*_*ESF*′−1_ to *G*_*ESF*′−8_. And then we prove the indistinguishability of *G*_*ESF*′−8_ and *G*_*SF*″_.

**Indistinguishability of** *G*_*ESF*′_ **and** *G*_*ESF*′−1_. For the security proof of the indistinguishability of *G*_*ESF*′_ and *G*_*ESF*′−1_, we define a sequence of games additional hybrid games *G*_*F*′,1_, …, *G*_*F*′,*h*_, …, *G*_*F*′,*q*_*n*__, where *G*_*ESF*′_ = *G*_*F*′,0_ and *q*_*n*_ is the number of all node identifiers that are used in HIBE private keys and IBE private keys of an adversary. In the game *G*_*F*′,*h*_ for 1 ≤ *h* ≤ *q*_*n*_, the challenge ciphertext is ESF-1, all IBE private keys are ESF-2, HIBE private keys with a node index *i*_*n*_ ≤ *h* are of ESF-3, the remaining HIBE private keys with a node index *i*_*n*_ > *h* are ESF-2.

**Oracle** *O*_3.1_ This oracle initializes in the same way as *O*_3_ and provides the attacker with initial group elements from the same distribution. Upon receiving a challenge HIBE-key-type query for *I* ∈ *Z*_*n*_, it chooses *r*, *y*′ ∈ *Z*_*n*_ randomly, and also chooses *X*_2_, *Y*_2_ ∈ *G*_*p*_2__ and *X*_3_, *Y*_3_ ∈ *G*_*p*_3__ randomly. It returns the group elements

$$\begin{array}{c} \left(w^{y^{\prime }}g_{3}^{y^{\prime }\psi },g^{y^{\prime }}g_{3}^{y^{\prime }},v^{y^{\prime }}{\left(u^{I_{j}}h\right)}^{r}X_{2}X_{3},g^{r}Y_{2}Y_{3}\right) \end{array}$$ (31)

to the attacker. It responds to a ciphertext-type query or a challenge IBE-key-type query in the same way as *O*_3_.

We define games *F*_1_, ⋯, *F*_*h*_*c*__, …, *F*_*q*_*s*__ where *F*_0_ = *G*_*S*′,*h*_ and *F*_*q*_*s*__ = *G*_*S*′,*h*+1_, and *q*_*s*_ is the maximun number of HIBE private key queries for the node index *h*. The games are formally defined as follows:

**Game** *F*_*h*_*c*__ This game *F*_*h*_*c*__ for 1 ≤ *h*_*c*_ ≤ *q*_*s*_ is almost the same as *G*_*F*′,*h*_ except the generation of HIBE private keys and IBE private keys with the node index *h*. An IBE private key with an index pair (*h*, *i*_*c*_) is generated as ESF-2. An HIBE private key with an index pair (*h*, *i*_*c*_) is generated as follows:

1. *i*_*c*_ ≤ *h*_*c*_: It generates a ESF-3 *SK*_*HIBE*,*h*_ by using the element groups in Eq 31.
2. *i*_*c*_ > *h*_*c*_: It simply generates a ESF-2 HIBE private key.

**Lemma 8** *Under Assumptions 3*, *no PPT attacker can distinguish between O*_3_ *and O*_3.1_ *with non-negligible advantage*. *So no PPT attacker can distinguish between F*_*h*_*c*_−1_ *and F*_*h*_*c*__ *with non-negligible advantage*.

Let $Adv_{A}^{G_{F^{\prime },h}}$ be the advantage of A in a game *G*_*F*′,*h*_. From the Lemma 8, we obtain the following equation

$$\begin{array}{c} Adv_{A}^{G_{ESF^{\prime }}}-Adv_{A}^{G_{ESF^{\prime }-1}}\leq \sum_{h=1}^{q_{n}}\left(Adv_{A}^{G_{F^{\prime },h-1}}-Adv_{A}^{G_{F^{\prime },h}}\right)\leq O\left(q_{n}q_{s}\right)\left(Adv_{B}^{A3}\right) \end{array}$$ (32)

**Indistinguishability of** *G*_*ESF*′−1_ **and** *G*_*ESF*′−2_. For the security proof of the indistinguishability of *G*_*ESF*′−1_ and *G*_*ESF*′−2_, we define a sequence of games *G*_*F*′−1,1_, …, *G*_*F*′−1,*h*_, …, *G*_*F*′−1,*q*_*n*__, where *G*_*ESF*′−1_ = *G*_*F*′−1,0_ and *q*_*n*_ is the number of all node identifiers that are used in HIBE private keys and IBE private keys of an adversary. In the game *G*_*F*′−1,*h*_ for 1 ≤ *h* ≤ *q*_*n*_, the challenge ciphertext is ESF-1, all HIBE private keys are ESF-3, IBE private keys with a node index *i*_*n*_ ≤ *h* are of ESF-3, the remaining HIBE private keys with a node index *i*_*n*_ > *h* are ESF-2.

**Oracle** *O*_3.2_ This oracle initializes in the same way as *O*_3.1_ and provides the attacker with initial group elements from the same distribution. Upon receiving a challenge IBE-key-type query for *T* ∈ *Z*_*n*_, it chooses *r*, *y*′ ∈ *Z*_*n*_ randomly, and also chooses *X*_2_, *Y*_2_ ∈ *G*_*p*_2__ and *X*_3_, *Y*_3_ ∈ *G*_*p*_3__ randomly. It returns the group elements

$$\begin{array}{c} \left(w_{0}^{y^{\prime }}g_{3}^{y^{\prime }\psi },g^{y^{\prime }}g_{3}^{y^{\prime }},v_{0}^{y^{\prime }}{\left(u_{0}^{T}h_{0}\right)}^{r}X_{2}X_{3},g^{r}Y_{2}Y_{3}\right) \end{array}$$ (33)

to the attacker. It responds to a ciphertext-type query or a challenge HIBE-key-type query in the same way as *O*_3.1_.

We define hybrid games *F*1_1_, ⋯, *F*1_*h*_*c*__, …, *F*1_*q*_*e*__ where *F*1_0_ = *G*_*F*′−1,*h*_ and *F*1_*q*_*e*__ = *G*_*F*′−1,*h*+1_, and *q*_*e*_ is the maximun number of IBE private key queries for the node index *h*. The games are formally defined as follows:

**Game** *F*1_*h*_*c*__ This game *F*1_*h*_*c*__ for 1 ≤ *h*_*c*_ ≤ *q*_*s*_ is almost the same as *G*_*F*′−1,*h*_ except the generation of HIBE private keys and IBE private keys with the node index *h*. A HIBE private key with an index pair (*h*, *i*_*c*_) is generated as ESF-3. An IBE private key with an index pair (*h*, *i*_*c*_) is generated as follows:

1. *i*_*c*_ ≤ *h*_*c*_: It generates a ESF-3 *SK*_*IBE*,*h*_*c*__.
2. *i*_*c*_ > *h*_*c*_: It simply generates a ESF-2 IBE private key.

**Lemma 9** *Under Assumptions 3*, *no PPT attacker can distinguish between O*_3.1_ *and O*_3.2_ *with non-negligible advantage*. *So no PPT attacker can distinguish between F*1_*h*_*c*_−1_ *and F*1_*h*_*c*__ *with non-negligible advantage*.

Let $Adv_{A}^{G_{F^{\prime }-1,h}}$ be the advantage of A in a game *G*_*F*′−1,*h*_. From the Lemma 9, we obtain the following equation

$$\begin{array}{c} Adv_{A}^{G_{ESF^{\prime }-1}}-Adv_{A}^{G_{ESF^{\prime }-2}}\leq \sum_{h=1}^{q_{n}}\left(Adv_{A}^{G_{F^{\prime }-1,h-1}}-Adv_{A}^{G_{F^{\prime }-1,h}}\right)\leq O\left(q_{n}q_{e}\right)\left(Adv_{B}^{A3}\right) \end{array}$$ (34)

**Indistinguishability of** *G*_*ESF*′−2_ **and** *G*_*ESF*′−3_. For the security proof of the indistinguishability of *G*_*ESF*′−2_ and *G*_*ESF*′−3_, we define the oracle below.

**Oracle** *O*_3.3_ This oracle initializes a bit differently from the other oracles. It fixes random elements *g*, *u*, *h*, *v*, *w*, *u*_0_, *h*_0_, *v*_0_, *w*_0_ ∈ *G*_*p*_1__, *g*_2_ ∈ *G*_*p*_2__, *g*_3_ ∈ *G*_*p*_3__. It chooses random exponents $s,\gamma ,\delta_{1},\delta_{2},y,y_{0},\psi ,\sigma_{1},\sigma_{2},a^{\prime },b^{\prime },t_{3},t_{3}^{\prime },t_{3}^{\prime \prime }\in Z_{N}$. It initially provides the attacker with the group elements:

$$\begin{array}{c} \begin{array}{c} (g,u,h,v,w,g^{s}{\left(g_{2}g_{3}\right)}^{\gamma },w^{y}{\left(g_{2}g_{3}\right)}^{y\psi },g^{y}{\left(g_{2}g_{3}\right)}^{y},v^{y}{\left(g_{2}g_{3}\right)}^{y\sigma_{1}},\\ u_{0},h_{0},v_{0},w_{0},w_{0}^{y_{0}}{\left(g_{2}g_{3}\right)}^{y_{0}\psi },g^{y_{0}}{\left(g_{2}g_{3}\right)}^{y_{0}},v_{0}^{y_{0}}{\left(g_{2}g_{3}\right)}^{y_{0}\sigma_{2}}) \end{array} \end{array}$$ (35)

What differs from the previous oracles here is the added $g_{3}^{\gamma }$ and term: notice that this is uniformly random in *G*_*p*_3__, since *γ* is random modulo *p*_3_ (and uncorrelated from its value modulo *p*_2_). This oracle answers the challenge-key type query in the same way as *O*_3.2_. To answer a ciphertext-type query for *I*, it chooses random values *t* ∈ *Z*_*N*_ and responds with:

$$\begin{array}{c} \left(w^{s}g_{2}^{\delta_{1}}v^{t}g_{2}^{\sigma_{1}t}g_{3}^{t_{3}},g^{t}g_{2}^{t}g_{3}^{t_{3}^{\prime }},{\left(u^{I}h\right)}^{t}g_{2}^{t(a^{\prime }I+b^{\prime })}g_{3}^{t_{3}^{\prime \prime }}\right) \end{array}$$ (36)

To answer a ciphertext-type query for *T*, it chooses random values *t* ∈ *Z*_*N*_ and responds with:

$$\begin{array}{c} \left(w_{0}^{s}g_{2}^{\delta_{2}}v_{0}^{t}g_{2}^{\sigma_{2}t}g_{3}^{t_{3}},g^{t}g_{2}^{t}g_{3}^{t_{3}^{\prime }},{\left(u_{0}^{T}h_{0}\right)}^{t}g_{2}^{t(a^{\prime }T+b^{\prime })}g_{3}^{t_{3}^{\prime \prime }}\right) \end{array}$$ (37)

It is crucial to note that these *G*_*p*_3__ terms arethe same for each ciphertext-type query response.

**Lemma 10** *Under Assumptions 4*, *no PPT attacker can distinguish between O*_3.2_ *and O*_3.3_ *with non-negligible advantage*. *So no PPT attacker can distinguish between G*_*ESF*′−2_ *and G*_*ESF*′−3_ *with non-negligible advantage*.

From the Lemma 10, we obtain the following equation

$$\begin{array}{c} Adv_{A}^{G_{ESF^{\prime }-2}}-Adv_{A}^{G_{ESF^{\prime }-3}}\leq \left(Adv_{B}^{A4}\right) \end{array}$$ (38)

**Indistinguishability of** *G*_*ESF*′−3_ **and** *G*_*ESF*′−4_: For the security proof of the indistinguishability of *G*_*ESF*′−3_ and *G*_*ESF*′−4_, we define a sequence of games additional hybrid games *G*_*F*′−3,1_, …, *G*_*F*′−3,*h*_, …, *G*_*F*′−3,*q*_*n*__, where *G*_*ESF*′−3_ = *G*_*F*′−3,0_ and *q*_*n*_ is the number of all node identifiers that are used in HIBE private keys and IBE private keys of an adversary. In the game *G*_*F*′−3,*h*_ for 1 ≤ *h* ≤ *q*_*n*_, the challenge ciphertext is ESF-5, all IBE private keys are ESF-3, HIBE private keys with a node index *i*_*n*_ ≤ *h* are of ESF-4, the remaining HIBE private keys with a node index *i*_*n*_ > *h* are ESF-3.

**Oracle** *O*_3.4_ This oracle initializes in the same way with *O*_3.3_ and provides the attacker the same initial elements as *O*_3.3_. This oracle answers the ciphertext-type query and IBE key- type query in the same way as *O*_3.3_. To answer a challenge HIBE private key type query for *I*, it chooses random values *y*, *r* ∈ *Z*_*N*_, *X*_2_, *Y*_2_ ∈ *G*_*p*_2__ randomly, and *X*_3_, *Y*_3_ ∈ *G*_*p*_3__ and responds with:

$$\begin{array}{c} \left(w^{y^{\prime }}{\left(g_{2}g_{3}\right)}^{y^{\prime }\psi },g^{y^{\prime }}{\left(g_{2}g_{3}\right)}^{y^{\prime }},v^{y^{\prime }}{\left(u^{I}h\right)}^{r}X_{2}X_{3},g^{r}Y_{2}Y_{3}\right) \end{array}$$ (39)

We define hybrid games *F*3_1_, ⋯, *F*3_*h*_*c*__, …, *F*3_*q*_*s*__ where *F*3_0_ = *G*_*F*′−3, *h*_ and *F*3_*q*_*s*__ = *G*_*F*′−3,*h*+1_, and *q*_*s*_ is the maximun number of HIBE private key queries for the node index *h*. The games are formally defined as follows:

**Game** *F*3_*h*_*c*__ This game *F*3_*h*_*c*__ for 1 ≤ *h*_*c*_ ≤ *q*_*s*_ is almost the same as *G*_*F*′−3,*h*_ except the generation of HIBE private keys and IBE private keys with the node index *h*. An IBE private key with an index pair (*h*, *i*_*c*_) is generated as ESF-3. An HIBE private key with an index pair (*h*, *i*_*c*_) is generated as follows:

1. *i*_*c*_ ≤ *h*_*c*_: It generates a ESF-4 *SK*_*HIBE*,*h*_ by using the element groups in Eq 39.
2. *i*_*c*_ > *h*_*c*_: It simply generates a ESF-3 HIBE private key.

**Lemma 11** *Under Assumptions 4*, *no PPT attacker can distinguish between O*_3.3_ *and O*_3.4_ *with non-negligible advantage*. *So no PPT attacker can distinguish between G*_*ESF*′−3_ *and G*_*ESF*′−4_ *with non-negligible advantage*.

Let $Adv_{A}^{G_{F^{\prime }-3,h}}$ be the advantage of A in a game *G*_*F*′−3,*h*_. From the Lemma 11, we obtain the following equation

$$\begin{array}{c} Adv_{A}^{G_{ESF^{\prime }-3}}-Adv_{A}^{G_{ESF^{\prime }-4}}\leq \sum_{h=1}^{q_{n}}\left(Adv_{A}^{G_{F^{\prime }-3,h-1}}-Adv_{A}^{G_{F^{\prime }-3,h}}\right)\leq O\left(q_{n}q_{s}\right)\left(Adv_{B}^{A4}\right) \end{array}$$ (40)

**Indistinguishability of** *G*_*ESF*′−4_ **and** *G*_*ESF*′−5_. For the security proof of the indistinguishability of *G*_*ESF*′−4_ and *G*_*ESF*′−5_, we define a sequence of games additional hybrid games *G*_*F*′−4,1_, …, *G*_*F*′−4,*h*_, …, *G*_*F*′−4,*q*_*n*__, where *G*_*ESF*′−4_ = *G*_*F*′−4,0_ and *q*_*n*_ is the number of all node identifiers that are used in HIBE private keys and IBE private keys of an adversary. In the game *G*_*F*′−4,*h*_ for 1 ≤ *h* ≤ *q*_*n*_, the challenge ciphertext is ESF-5, all HIBE private keys are ESF-4, IBE private keys with a node index *i*_*n*_ ≤ *h* are of ESF-4, the remaining IBE private keys with a node index *i*_*n*_ > *h* are ESF-3.

**Oracle** *O*_3.5_ This oracle initializes in the same way with *O*_3.4_ and provides the attacker the same initial elements as *O*_3.4_. This oracle answers the ciphertext-type query and HIBE key- type query in the same way as *O*_3.4_. To answer a challenge IBE private key type query for *T*, it chooses random values *y*, *r* ∈ *Z*_*N*_, *X*_2_, *Y*_2_ ∈ *G*_*p*_2__ randomly, and *X*_3_, *Y*_3_ ∈ *G*_*p*_3__ and responds with:

$$\begin{array}{c} \left(w_{0}^{y^{\prime }}{\left(g_{2}g_{3}\right)}^{y^{\prime }\psi },g^{y^{\prime }}{\left(g_{2}g_{3}\right)}^{y^{\prime }},v_{0}^{y^{\prime }}{\left(u_{0}^{T}h_{0}\right)}^{r}X_{2}X_{3},g^{r}Y_{2}Y_{3}\right) \end{array}$$ (41)

We define hybrid games *F*4_1_, ⋯, *F*4_*h*_*c*__, …, *F*4_*q*_*s*__ where *F*4_0_ = *G*_*F*′−4,*h*_ and *F*4_*q*_*e*__ = *G*_*F*′−4,*h*+1_, and *q*_*e*_ is the maximun number of IBE private key queries for the node index *h*. The games are formally defined as follows:

**Game** *F*4_*h*_*c*__ This game *F*4_*h*_*c*__ for 1 ≤ *h*_*c*_ ≤ *q*_*e*_ is almost the same as *G*_*F*′−4,*h*_ except the generation of HIBE private keys and IBE private keys with the node index *h*. An HIBE private key with an index pair (*h*, *i*_*c*_) is generated as ESF-4. An IBE private key with an index pair (*h*, *i*_*c*_) is generated as follows:

1. *i*_*c*_ ≤ *h*_*c*_: It generates a ESF-4 *SK*_*IBE*,*h*_ by using the element groups in Eq 41.
2. *i*_*c*_ > *h*_*c*_: It simply generates a ESF-3 IBE private key.

**Lemma 12** *Under Assumptions 4*, *no PPT attacker can distinguish between O*_3.4_ *and O*_3.5_ *with non-negligible advantage*. *So no PPT attacker can distinguish between G*_*ESF*′−4_ *and G*_*ESF*′−5_ *with non-negligible advantage*.

Let $Adv_{A}^{G_{F^{\prime }-4,h}}$ be the advantage of A in a game *G*_*F*′−4,*h*_. From the Lemma 12, we obtain the following equation

$$\begin{array}{c} Adv_{A}^{G_{ESF^{\prime }-4}}-Adv_{A}^{G_{ESF^{\prime }-5}}\leq \sum_{h=1}^{q_{n}}\left(Adv_{A}^{G_{F^{\prime }-4,h-1}}-Adv_{A}^{G_{F^{\prime }-4,h}}\right)\leq O\left(q_{n}q_{e}\right)\left(Adv_{B}^{A4}\right) \end{array}$$ (42)

**Indistinguishability of** *G*_*ESF*′−5_ **and** *G*_*ESF*′−6_. For the security proof of the indistinguishability of *G*_*ESF*′−5_ and *G*_*ESF*′−6_, we define the oracle below.

**Oracle** *O*_3.6_ This oracle fixes random elements *g*, *u*, *h*, *v*, *w*, *u*_0_, *h*_0_, *v*_0_, *w*_0_ ∈ *G*_*p*_1__, *g*_2_ ∈ *G*_*p*_2__, *g*_3_ ∈ *G*_*p*_3__. It chooses random exponents $s,\gamma ,\delta_{1},\delta_{2},y,y_{0},\psi ,\sigma_{1},\sigma_{2},a^{\prime },b^{\prime },t_{3},t_{3}^{\prime },t_{3}^{\prime \prime }\in Z_{N}$. It initially provides the attacker with the group elements:

$$\begin{array}{c} \begin{array}{c} (g,u,h,v,w,g^{s}{\left(g_{2}g_{3}\right)}^{\gamma },w^{y}{\left(g_{2}g_{3}\right)}^{y\psi },g^{y}{\left(g_{2}g_{3}\right)}^{y},v^{y}{\left(g_{2}g_{3}\right)}^{y\sigma_{1}},\\ u_{0},h_{0},v_{0},w_{0},w_{0}^{y_{0}}{\left(g_{2}g_{3}\right)}^{y_{0}\psi },g^{y_{0}}{\left(g_{2}g_{3}\right)}^{y_{0}},v_{0}^{y_{0}}{\left(g_{2}g_{3}\right)}^{y_{0}\sigma_{2}}) \end{array} \end{array}$$ (43)

What differs from the previous oracles here is the added $g_{3}^{\gamma }$ and term: notice that this is uniformly random in *G*_*p*_3__, since *γ* is random modulo *p*_3_ (and uncorrelated from its value modulo *p*_2_). This oracle answers the challenge-key type query in the same way as *O*_3.2_. To answer a ciphertext-type query for *I*, it chooses random values *t* ∈ *Z*_*N*_ and responds with:

$$\begin{array}{c} \left(w^{s}g_{2}^{\delta_{1}}v^{t}g_{2}^{\sigma_{1}t}g_{3}^{t_{3}},g^{t}g_{2}^{t}g_{3}^{t_{3}^{\prime }},{\left(u^{I}h\right)}^{t}g_{2}^{t(a^{\prime }I+b^{\prime })}g_{3}^{t_{3}^{\prime \prime }}\right) \end{array}$$ (44)

To answer a ciphertext-type query for *T*, it chooses random values *t* ∈ *Z*_*N*_ and responds with:

$$\begin{array}{c} \left(w_{0}^{s}g_{2}^{\delta_{2}}v_{0}^{t}g_{2}^{\sigma_{2}t}g_{3}^{t_{3}},g^{t}g_{2}^{t}g_{3}^{t_{3}^{\prime }},{\left(u_{0}^{T}h_{0}\right)}^{t}g_{2}^{t(a^{\prime }T+b^{\prime })}g_{3}^{t_{3}^{\prime \prime }}\right) \end{array}$$ (45)

It is crucial to note that these *G*_*p*_3__ terms arethe same for each ciphertext-type query response.

**Lemma 13** *Under Assumptions 4*, *no PPT attacker can distinguish between O*_3.5_ *and O*_3.6_ *with non-negligible advantage*. *So no PPT attacker can distinguish between G*_*ESF*′−5_ *and G*_*ESF*′−6_ *with non-negligible advantage*.

From the Lemma 13, we obtain the following equation

$$\begin{array}{c} Adv_{A}^{G_{ESF^{\prime }-5}}-Adv_{A}^{G_{ESF^{\prime }-6}}\leq \left(Adv_{B}^{A4}\right) \end{array}$$ (46)

**Indistinguishability of** *G*_*ESF*′−6_ **and** *G*_*ESF*′−7_. For the security proof of the indistinguishability of *G*_*ESF*′−6_ and *G*_*ESF*′−7_, we define a sequence of games *G*_*F*′−6,1_, …, *G*_*F*′−6,*h*_, …, *G*_*F*′−6,*q*_*n*__, where *G*_*ESF*′−6_ = *G*_*F*′−6,0_ and *q*_*n*_ is the number of all node identifiers that are used in HIBE private keys and IBE private keys of an adversary. In the game *G*_*F*′−6,*h*_ for 1 ≤ *h* ≤ *q*_*n*_, the challenge ciphertext is ESF-1, all HIBE private keys are ESF-4, IBE private keys with a node index *i*_*n*_ ≤ *h* are semi-functional, the remaining IBE private keys with a node index *i*_*n*_ > *h* are ESF-4.

**Oracle** *O*_7/2′_ This oracle initializes in the same way as *O*_3.6_ and provides the attacker with initial group elements from the same distribution. Upon receiving a challenge IBE-key-type query for *T* ∈ *Z*_*n*_, it chooses *r*, *y*′ ∈ *Z*_*n*_ randomly, and also chooses *X*_2_, *Y*_2_ ∈ *G*_*p*_2__ and *X*_3_, *Y*_3_ ∈ *G*_*p*_3__ randomly. It returns the group elements

$$\begin{array}{c} \left(w_{0}^{y^{\prime }}{\left(g_{2}g_{3}\right)}^{y^{\prime }\psi },g^{y^{\prime }}{\left(g_{2}g_{3}\right)}^{y^{\prime }},v_{0}^{y^{\prime }}{\left(u_{0}^{T}h_{0}\right)}^{r}X_{2}X_{3},g^{r}Y_{3}\right) \end{array}$$ (47)

to the attacker. It responds to a ciphertext-type query or a challenge HIBE-key-type query in the same way as *O*_3.6_.

We define hybrid games *F*6_1,1_, *F*6_1,2_, ⋯, *F*6_*h*_*c*_,1_, *F*6_*h*_*c*_,2_, …, *F*6_*q*_*s*_,1_, *F*6_*q*_*s*_,2_ where *F*6_0,2_ = *G*_*F*′−6,*h*_ and *F*6_*q*_*e*_,2_ = *G*_*F*′−6,*h*+1_, and *q*_*e*_ is the maximun number of IBE private key queries for the node index *h*. The games are formally defined as follows:

**Game** *F*6_*h*_*c*_,1_ This game *F*6_*h*_*c*_,1_ for 1 ≤ *h*_*c*_ ≤ *q*_*e*_ is almost the same as *G*_*F*′−6,*h*_ except the generation of HIBE private keys and IBE private keys with the node index *h*. An HIBE private key with an index pair (*h*, *i*_*c*_) is generated as ESF-4. An IBE private key with an index pair (*h*, *i*_*c*_) is generated as follows:

1. *i*_*c*_ < *h*_*c*_: It generates a semi-functional key *SK*_*IBE*,*h*_.
2. *i*_*c*_ = *h*_*c*_: It generates a normal $SK_{IBE,h}^{\prime }$ and converts the key to a ESF-5 *SK*_*IBE*,*h*_ by using the element groups in Eq 47.
3. *i*_*c*_ > *h*_*c*_: It generates a ESF-4 IBE private key.

**Game** *F*6_*h*_*c*_,2_ This game *F*6*h*_*c*_, 2 for 1 ≤ *h*_*c*_ ≤ *q*_*e*_ is almost the same as *F*6_*h*_*c*_,1_ except the generation of IBE private key with an index pair (*h*, *i*_*c*_) and *i*_*c*_ = *h*_*c*_ is generated as a semi-functional *SK*_*IBE*,*h*_.

**Lemma 14** *Under Assumptions 4*, *no PPT attacker can distinguish between O*_3.6_ *and O*_7/2′_ *with non-negligible advantage*. *So no PPT attacker can distinguish between F*6_*i*−1,2_ *and F*6_*i*,1_ *with non-negligible advantage*.

**Oracle** ${\tilde{O}}_{i}^{*}$ This oracle initializes in the same way as $O_{i}^{*}$, and provides the attacker with initial group elements from the same distribution. It also responds to a ciphertext-type query as same as $O_{i}^{*}$. It responds to a HIBE-key-type query in the same way as *O*_3.6_. Upon receiving a challenge IBE-key-type query for *T* ∈ *Z*_*n*_, it chooses *r*, *y*′ ∈ *Z*_*n*_ randomly, and returns the group elements

$$\begin{array}{c} \left(w_{0}^{y^{\prime }}{\left(g_{2}g_{3}\right)}^{y^{\prime }\psi },g^{y^{\prime }}{\left(g_{2}g_{3}\right)}^{y^{\prime }},v_{0}^{y^{\prime }}{\left(g_{2}g_{3}\right)}^{y^{\prime }\sigma_{2}}{\left(u_{0}^{T}h_{0}\right)}^{r},g^{r}{\left(g_{2}g_{3}\right)}^{y^{\prime }}\right) \end{array}$$ (48)

to the attacker.

**Lemma 15** *Under Assumptions 3*, *no PPT attacker can distinguish between O*_7/2′_ *and* ${\tilde{O}}_{q_{c}}^{*}$ *with non-negligible advantage*. *So no PPT attacker can distinguish between F*6_*i*,1_ *and F*6_*i*,2_ *with non-negligible advantage*.

Let $Adv_{A}^{G_{E^{\prime },h}}$ be the advantage of A in a game *G*_*E*′,*h*_. From the Lemma 14, 15, we obtain the following equation

$$\begin{array}{c} \begin{array}{c} Adv_{A}^{G_{F^{\prime }-6,h-1}}-Adv_{A}^{G_{F^{\prime }-6,h}}\leq \sum_{h_{c}=1}^{q_{e}}(|Adv_{A}^{F6_{h_{c},1}}-Adv_{A}^{F6_{h_{c},2}}|+|Adv_{A}^{F6_{h_{c}-1,2}}-Adv_{A}^{F6_{h_{c},1}}\left|\right)\\ \leq O\left(q_{e}\right)\left(Adv_{B}^{A3}+Adv_{B}^{A4}\right) \end{array} \end{array}$$

So we obtain the following equation

$$\begin{array}{c} Adv_{A}^{G_{ESF^{\prime }-6}}-Adv_{A}^{G_{ESF^{\prime }-7}}\leq \sum_{h=1}^{q_{n}}\left(Adv_{A}^{G_{E^{\prime },h-1}}-Adv_{A}^{G_{E^{\prime },h}}\right)\leq O\left(q_{n}q_{e}\right)\left(Adv_{B}^{A3}+Adv_{B}^{A4}\right) \end{array}$$ (49)

**Indistinguishability of** *G*_*ESF*′−7_ **and** *G*_*ESF*′−8_. We now prove the indistinguishability of *G*_*ESF*′−7_ and *G*_*ESF*′−8_ in a hybrid argument using polynomially many steps. We let *q*_*c*_ denote the number of ciphertext-type queries made by a PPT attacker A. Firstly we define hybrid games $S_{-1,1}^{\prime },S_{0,2}^{\prime },S_{0,3}^{\prime },S_{0,1}^{\prime }$, $S_{1,2}^{\prime },S_{1,3}^{\prime },S_{1,1}^{\prime }\ldots$, $S_{k,2}^{\prime },S_{k,3}^{\prime },S_{k,1}^{\prime },\ldots ,S_{q_{c}-1,2}^{\prime },S_{q_{c}-1,3}^{\prime },S_{q_{c}-1,1}^{\prime }$, where $S_{-1,1}^{\prime }=G_{ESF^{\prime }-7}$ and $S_{q_{c}-1,1}^{\prime }=G_{ESF^{\prime }-8}$. The games are formally defined as follows:

**Game** $S_{k,1}^{\prime }$ This game $S_{k,1}^{\prime }$ for 0 ≤ *k* ≤ *q*_*c*_ is almost the same as *G*_*ESF*′−7_ except the generation of the challenge ciphertext. The challenge ciphertext of $\left(T^{*},I_{1}^{*},\ldots ,I_{q_{c}-1}^{*}\right)$ is generated as EST-2^k^-CT outputed by **EncryptESF-2**^k^.

**Game** $S_{k,2}^{\prime }$ This game $S_{k,2}^{\prime }$ for 0 ≤ *k* ≤ *q*_*c*_ − 1 is almost the same as *G*_*ESF*′−7_ except the generation of the challenge ciphertext. The challenge ciphertext of $\left(T^{*},I_{1}^{*},\ldots ,I_{q_{c}-1}^{*}\right)$ is generated as EST-3^k^-CT outputed by **EncryptESF-3**^k^.

**Game** $S_{k,3}^{\prime }$ This game $S_{k,3}^{\prime }$ for 0 ≤ *k* ≤ *q*_*c*_ − 1 is almost the same as *G*_*ESF*′−7_ except the generation of the challenge ciphertext. The challenge ciphertext of $\left(T^{*},I_{1}^{*},\ldots ,I_{q_{c}-1}^{*}\right)$ is generated as EST-4^k^-CT outputed by **EncryptESF-4**^k^.

We will define additional oracles ${\tilde{O}}_{i}^{*}$ for each *i* from 0 to *q*_*c*_ − 1, ${\tilde{O}}_{i}^{\prime }$ for each *i* from 0 to *q*_*c*_ − 1, and ${\tilde{O}}_{i}^{\prime \prime }$ for each *i* from 0 to *q*_*c*_ − 1.

**Oracle** ${\tilde{O}}_{i}^{\prime }$ This oracle acts the same as ${\tilde{O}}_{i}^{*}$ except that its response to the ciphertext-type query is as same as $O_{i}^{\prime }$.

**Oracle** ${\tilde{O}}_{i}^{\prime \prime }$ This oracle acts the same as ${\tilde{O}}_{i}^{*}$ except that its response to the ciphertext-type query is as same as $O_{i}^{\prime \prime }$.

**Lemma 16** *Under Assumptions 3*, *no PPT attacker can distinguish between* ${\tilde{O}}_{k}^{*}$ *and* ${\tilde{O}}_{k}^{\prime \prime }$ *with non-negligible advantage*. *So no PPT attacker can distinguish between* $S_{k,1}^{\prime }$ *and* $S_{k,3}^{\prime }$ *with non-negligible advantage*.

**Lemma 17** *Under Assumptions 4*, *no PPT attacker can distinguish between* ${\tilde{O}}_{k}^{\prime \prime }$ *and* ${\tilde{O}}_{k}^{\prime }$ *with non-negligible advantage*. *So no PPT attacker can distinguish between* $S_{k,3}^{\prime }$ *and* $S_{k,2}^{\prime }$ *with non-negligible advantage*.

**Lemma 18** *Under Assumptions 3*, *no PPT attacker can distinguish between* ${\tilde{O}}_{k}^{\prime }$ *and* ${\tilde{O}}_{k-1}^{*}$ *with non-negligible advantage*. *So no PPT attacker can distinguish between* $S_{k,2}^{\prime }$ *and* $S_{k-1,1}^{\prime }$ *with non-negligible advantage*.

Let $Adv_{A}^{S_{k,1}^{\prime }}$, $Adv_{A}^{S_{k,2}^{\prime }}$ and $Adv_{A}^{S_{k,3}^{\prime }}$ be the advantage of A in the games $S_{k,1}^{\prime }$, $S_{k,2}^{\prime }$ and $S_{k,3}^{\prime }$. From the Lemma 16, 17, 18, we obtain the following equation

$$\begin{array}{c} \begin{array}{c} Adv_{A}^{G_{ESF^{\prime }-7}}-Adv_{A}^{G_{ESF^{\prime }-8}}\leq \sum_{k=0}^{q_{c}-1}(|Adv_{A}^{S_{k-1,1}^{\prime }}-Adv_{A}^{S_{k,2}^{\prime }}|+|Adv_{A}^{S_{k,2}^{\prime }}-Adv_{A}^{S_{k,3}^{\prime }}|\\ +|Adv_{A}^{S_{k,3}^{\prime }}-Adv_{A}^{S_{k,1}^{\prime }}\left|\right)\\ \leq q_{c}\left(2Adv_{B}^{A3}+Adv_{B}^{A4}\right) \end{array} \end{array}$$ (50)

**Indistinguishability of** *G*_*ESF*′−8_ **and** *G*_*SF*″_. For the security proof of the indistinguishability of *G*_*ESF*′−8_ and *G*_*SF*″_, we define a sequence of games *G*_*F*′−8,1_, …, *G*_*F*′−8,*h*_, …, *G*_*F*′−8,*q*_*n*__, where *G*_*ESF*′−8_ = *G*_*F*′−8,0_ and *q*_*n*_ is the number of all node identifiers that are used in HIBE private keys and IBE private keys of an adversary. In the game *G*_*F*′−8,*h*_ for 1 ≤ *h* ≤ *q*_*n*_, the challenge ciphertext is semi-functional, all IBE private keys are semi-functional, HIBE private keys with a node index *i*_*n*_ ≤ *h* are semi-functional, the remaining HIBE private keys with a node index *i*_*n*_ > *h* are ESF-4.

**Oracle** *O*_7/2_ This oracle initializes in the same way as ${\tilde{O}}_{0}^{*}$ and provides the attacker with initial group elements from the same distribution. Upon receiving a challenge HIBE-key-type query for *I* ∈ *Z*_*n*_, it chooses *r*, *y*′ ∈ *Z*_*n*_ randomly, and also chooses *X*_2_, *Y*_2_ ∈ *G*_*p*_2__ and *X*_3_, *Y*_3_ ∈ *G*_*p*_3__ randomly. It returns the group elements

$$\begin{array}{c} \left(w^{y^{\prime }}{\left(g_{2}g_{3}\right)}^{y^{\prime }\psi },g^{y^{\prime }}{\left(g_{2}g_{3}\right)}^{y^{\prime }},v^{y^{\prime }}{\left(u^{I}h\right)}^{r}X_{2}X_{3},g^{r}Y_{3}\right) \end{array}$$ (51)

to the attacker. It responds to a ciphertext-type query or a challenge IBE-key-type query in the same way as ${\tilde{O}}_{0}^{*}$.

We define hybrid games *I*_1,1_, *I*_1,2_, ⋯, *I*_*h*_*c*_,1_, *I*_*h*_*c*_,2_, …, *I*_*q*_*s*_,1_, *I*_*q*_*s*_,2_ where *I*_0,2_ = *G*_*F*′−8,*h*_ and *I*_*q*_*s*_,2_ = *G*_*F*′−8,*h*+1_, and *q*_*s*_ is the maximun number of HIBE private key queries for the node index *h*. The games are formally defined as follows:

**Game** *I*_*h*_*c*_,1_ This game *I*_*h*_*c*_,1_ for 1 ≤ *h*_*c*_ ≤ *q*_*s*_ is almost the same as *G*_*F*′−8,*h*_ except the generation of HIBE private keys and IBE private keys with the node index *h*. An IBE private key with an index pair (*h*, *i*_*c*_) is generated as a semi-functional key. An HIBE private key with an index pair (*h*, *i*_*c*_) is generated as follows:

1. *i*_*c*_ < *h*_*c*_: It generates a semi-functional *SK*_*HIBE*,*h*_.
2. *i*_*c*_ = *h*_*c*_: It generates a ESF-5 *SK*_*HIBE*,*h*_.
3. *i*_*c*_ > *h*_*c*_: It generates a ESF-4 *SK*_*HIBE*,*h*_.

**Game** *H*_*h*_*c*_,2_ This game *H*_*h*_*c*_,1_ for 1 ≤ *h*_*c*_ ≤ *q*_*s*_ is almost the same as *H*_*h*_*c*_,1_ except the generation of HIBE private key with an index pair (*h*, *i*_*c*_) and *i*_*c*_ = *h*_*c*_ is generated as a semi-functional *SK*_*HIBE*,*h*_.

**Lemma 19** *Under Assumptions 4*, *no PPT attacker can distinguish between* ${\tilde{O}}_{0}^{*}$ *and O*_7/2_ *with non-negligible advantage*. *So no PPT attacker can distinguish between I*_*h*_*c*_−1,2_ *and I*_*h*_*c*_,1_ *with non-negligible advantage*.

**Lemma 20** *Under Assumptions 3*, *no PPT attacker can distinguish between O*_7/2_ *and O*_4_ *with non-negligible advantage*. *So no PPT attacker can distinguish between I*_*h*_*c*_,1_ *and I*_*h*_*c*_,2_ *with non-negligible advantage*.

Let $Adv_{A}^{G_{E^{\prime },h}}$ be the advantage of A in a game *G*_*E*′,*h*_. From the Lemma 19, 20, we obtain the following equations

$$\begin{array}{c} \begin{array}{c} Adv_{A}^{G_{F^{\prime }-8,h-1}}-Adv_{A}^{G_{F^{\prime }-8,h}}\leq \sum_{h_{c}=1}^{q_{s}}(|Adv_{A}^{I_{h_{c},1}}-Adv_{A}^{I_{h_{c},2}}|+|Adv_{A}^{I_{h_{c}-1,2}}-Adv_{A}^{I_{h_{c},1}}\left|\right)\\ \leq O\left(q_{s}\right)\left(Adv_{B}^{A3}+Adv_{B}^{A4}\right) \end{array} \end{array}$$

and

$$\begin{array}{c} Adv_{A}^{G_{ESF^{\prime }-8}}-Adv_{A}^{G_{SF^{\prime \prime }}}\leq \sum_{h=1}^{q_{n}}\left(Adv_{A}^{G_{F^{\prime }-8,h-1}}-Adv_{A}^{G_{F^{\prime }-8,h}}\right)\leq O\left(q_{n}q_{s}\right)\left(Adv_{B}^{A3}+Adv_{B}^{A4}\right) \end{array}$$ (52)

So we obtain the following equation

$$\begin{array}{c} \begin{array}{c} Adv_{A}^{G_{ESF^{\prime }}}-Adv_{A}^{G_{SF^{\prime \prime }}}\leq |(Adv_{A}^{G_{ESF^{\prime }}}-Adv_{A}^{G_{ESF^{\prime }-1}}|+\\ \;\sum_{i=1}^{7}|(Adv_{A}^{G_{ESF^{\prime }-i}}-Adv_{A}^{G_{ESF^{\prime }-i+1}}|+|(Adv_{A}^{G_{ESF^{\prime }-8}}-Adv_{A}^{G_{SF^{\prime \prime }}}|\\ \leq O\left(q_{n}q_{s}\right)\left(Adv_{B}^{A3}\right)+O\left(q_{n}q_{e}\right)\left(Adv_{B}^{A3}\right)+\left(Adv_{B}^{A4}\right)+O\left(q_{n}q_{s}\right)\left(Adv_{B}^{A4}\right)+\\ O\left(q_{n}q_{e}\right)\left(Adv_{B}^{A4}\right)+\left(Adv_{B}^{A4}\right)+O\left(q_{n}q_{e}\right)\left(Adv_{B}^{A3}+Adv_{B}^{A4}\right)+\\ O\left(q_{c}\right)\left(2Adv_{B}^{A3}+Adv_{B}^{A4}\right)+O\left(q_{n}q_{s}\right)\left(Adv_{B}^{A3}+Adv_{B}^{A4}\right)\\ \leq \left(O\left(q_{n}\left(q_{s}+q_{e}\right)\right)+O\left(l\right)\right)\left(Adv_{B}^{A3}+Adv_{B}^{A4}\right) \end{array} \end{array}$$ (53)

According to the equations Eqs 23, 28, 30, 53, we obtain the following equation

$$\begin{array}{c} \begin{array}{c} Adv_{A}^{G_{C^{\prime }}}-Adv_{A}^{G_{SF^{\prime \prime }}}\leq |(Adv_{A}^{G_{C^{\prime }}}-Adv_{A}^{G_{E-S}}|+|(Adv_{A}^{G_{E-S}}-Adv_{A}^{G_{E-S^{\prime }}}|\\ \;+|(Adv_{A}^{G_{E-S^{\prime }}}-Adv_{A}^{G_{ESF^{\prime }}}|+|(Adv_{A}^{G_{ESF^{\prime }}}-Adv_{A}^{G_{SF^{\prime \prime }}}|\\ \;\leq \left(O\left(q_{n}\left(q_{s}+q_{e}\right)\right)+O\left(l\right)\right)\left(Adv_{B}^{A3}+Adv_{B}^{A4}\right)\\ \;\leq \left(O\left(q_{n}logN_{max}\right)+O\left(q_{n}r_{max}logN_{max}\right)+O\left(l\right)\right)\left(Adv_{B}^{A3}+Adv_{B}^{A4}\right) \end{array} \end{array}$$ (54)

#### 4.4.3 Type-2 adversary

The Type-2 adversary is restricted to queries on the update keys on the time *T* ∉ T*. So we could show an information theoretic argument for the update keys and avoid a potential paradox for the secret keys, in the similar way of the situation of the Type-1 adversary in Sec.4.4.2.

The proof strategy for the indistinguishabilities between games *G*_*C*′_ to *G*_*EK*−*U*_, and to *G*_*ESF*′_ under the Type-2 adversary is by going through several intermediary oracles in Table 6, where the type settings of the update keys and the secret keys in every oracle and game respectively are swaped compared to the setting in Sec.4.4.2. The proof of every respective lemma is similar to the proof for the Type-1 adversary, and finally we obtain the advantage between *G*_*C*′_ and *G*_*ESF*′_ under the Type-2 adversary as same in Eq 54.

### 4.5 Indistinguishability of *G*_*SF*″_ and *G*_*SF*′_

In the game *G*_*SF*″_, the type of ciphertexts, secret keys and update keys are all semi-functional, except the decryption keys are normal. In this section, we give the proof of the indistinguishability of *G*_*SF*″_ and *G*_*SF*′_ via a hybrid argument over the sequence of games *G*_*SF*″_, *G*_*E*−*D*_, *G*_*ESF*_ and *G*_*SF*′_ to transform the type of decryption keys from normal to ephemeral semi-functional, and then to semi-functional.

The hybrid argument we conduct for the indistinguishability of *G*_*SF*″_ and *G*_*SF*′_ is following the process similar to the argument for the indistinguishability of *G*_*C*′_ and *G*_*SF*″_. But it is simpler since the transformation of challenge type only happens to the decryption keys and the challenge ciphertexts. So we just treat the decryption keys as a secret key of the identity (*T*, *id*_1_, ⋯, *id*_*j*_) and follow the proof strategy in the nested dual system encryption of the unbounded HIBE [12].

We show the oracles for proving the the indistinguishability of *G*_*SF*″_ and *G*_*SF*′_ in Table 7 which answer queries from the challenger B by sampling various distributions of group elements to construct the decryption keys, challenge ciphertexts and also the secret keys and update keys.

**Table 7:** Simulation of challenge keys and cipertext in oracles for the proof of the indistinguishability between *G*_*SF*″_ and *G*_*SF*′_.

Oracle	CT-Type Response	SK-Type Response	UK-Type Response	DK-Type Response
***O***_**4**_	SF	SF	SF	Normal
*O*_9/2_	SF	SF	SF	ESF-1
***O***_**5**_	SF	SF	SF	ESF-2
${Ò}_{i}^{*}$	ESF-2^*i*^	SF	SF	ESF-2
${Ò}_{i}^{\prime }$	ESF-3^*i*^	SF	SF	ESF-2
${Ò}_{i}^{\prime \prime }$	ESF-4^*i*^	SF	SF	ESF-2
***O***_**6**_	ESF-1	SF	SF	ESF-2
*O*_6.1_	ESF-1	SF	SF	ESF-3
†*O*_6.2_	ESF-5	SF	SF	ESF-3
†*O*_6.3_	ESF-5	SF	SF	ESF-4
${Ő}_{i}^{*}$	ESF-2^*i*^	SF	SF	ESF-4
${Ő}_{i}^{\prime }$	ESF-3^*i*^	SF	SF	ESF-4
${Ő}_{i}^{\prime \prime }$	ESF-4^*i*^	SF	SF	ESF-4
*O*_13/2_	SF	SF	SF	ESF-5
***O***_**7**_	SF	SF	SF	SF

Note: oracles marked with † initialize with an extra *G*_*p*_3__ term on $g^{s}g_{2}^{\gamma }$.

Since these oracles initially provide the attacker with a description of the group *G*, as well as the group elements

$$\begin{array}{c} \begin{array}{c} g,u,v,w,g^{s}g_{2}^{\gamma },w^{y}{\left(g_{2}g_{3}\right)}^{y\psi },g^{y}{\left(g_{2}g_{3}\right)}^{y},v^{y}{\left(g_{2}g_{3}\right)}^{y\sigma },\\ u_{0},v_{0},w_{0},w_{0}^{y_{0}}{\left(g_{2}g_{3}\right)}^{y_{0}\psi },g^{y_{0}}{\left(g_{2}g_{3}\right)}^{y_{0}},v_{0}^{y_{0}}{\left(g_{2}g_{3}\right)}^{y_{0}\sigma } \end{array} \end{array}$$

So the simulation of semi-functional secret keys and update keys are achievable in all oracles and games.

**(1) Indistinguishability of** *G*_*SF*″_ **and** *G*_*E*−*D*_

For the security proof of the indistinguishability of *G*_*SF*″_ and *G*_*E*−*D*_, we define a sequence of additional hybrid games *J*_1,1_, *J*_1,2_, ⋯, *J*_*h*_*d*_,1_, *J*_*h*_*d*_,2_, …, *J*_*q*_*d*_,1_, *J*_*q*_*d*_,2_ where *J*_0,2_ = *G*_*SF*″_ and *J*_*q*_*d*_,2_ = *G*_*E*−*D*_, and *q*_*d*_ is the number of decryption key queries of an adversary. The games and a additional oracle *O*_9/2_ used in the proof are formally defined as follows:

**Oracle** *O*_9/2_ This oracle initializes in the same way as *O*_4_, *O*_5_ and provides the attacker with initial group elements from the same distribution. Upon receiving a challenge decryption-key-type query for *I*, *T* ∈ *Z*_*n*_, it chooses *r*, *y*′, *r*′, *y*″ ∈ *Z*_*n*_ randomly, and also chooses $X_{2},Y_{2},X_{2}^{\prime },Y_{2}^{\prime }\in G_{p_{2}},X_{3},Y_{3},X_{3}^{\prime },Y_{3}^{\prime }\in G_{p_{3}}$ randomly. It returns the group elements

$$\begin{array}{c} \left(w^{y^{\prime }},g^{y^{\prime }},v^{y^{\prime }}{\left(u^{I}h\right)}^{r}X_{3},g^{r}Y_{3},w_{0}^{y^{\prime \prime }},g^{y^{\prime \prime }},v_{0}^{y^{\prime \prime }}{\left(u_{0}^{T}h_{0}\right)}^{r^{\prime }}X_{3}^{\prime },g^{r^{\prime }}Y_{3}^{\prime }\right) \end{array}$$ (55)

to the attacker. It responds to a ciphertext-type query and a challenge (H)IBE-key-type query in the same way as *O*_4_.

**Game** *J*_*h*_*d*_,1_ This game *J*_*h*_*d*_,1_ for 1 ≤ *h*_*d*_ ≤ *q*_*d*_ is almost the same as *G*_*EF*″_ except the generation of the decryption keys *DK*_*ID*|_*k*_,*T*_.

1. *i*_*d*_ < *h*_*d*_: It generates a normal $DK_{{ID|}_{k},T}^{\prime }$ and converts the key to a ESF-2 *DK*_*ID*|_*k*_,*T*_ by using the element groups in Eq 17.
2. *i*_*d*_ = *h*_*d*_: It generates a normal $DK_{{ID|}_{k},T}^{\prime }$ and converts the key to a ESF-1 *DK*_*ID*|_*k*_,*T*_ by using the element groups in Eq 55.
3. *i*_*d*_ > *h*_*d*_: It simply generates a normal decryption key.

**Game** *J*_*h*_*d*_,2_ This game *J*_*h*_*d*_,1_ for 1 ≤ *h*_*d*_ ≤ *q*_*d*_ is almost the same as *J*_*h*_*d*_,1_ except the generation of the $h_{d}^{st}$ decryption key is generated as a ESF-2 *DK*_*ID*|_*k*_, *T*_ by using the element groups in Eq 17. The first *h*_*d*_ decryption keys are generated as ESF-2 and the remaining decryption keys are generated as normal.

**Lemma 21** *Under Assumptions 3*, *no PPT attacker can distinguish between O*_4_ *and O*_9/2_ *with non-negligible advantage*. *So no PPT attacker can distinguish between J*_*h*_*d*_−1,2_ *and J*_*h*_*d*_,1_ *with non-negligible advantage*.

**Lemma 22** *Under Assumptions 4*, *no PPT attacker can distinguish between O*_9/2_ *and O*_5_ *with non-negligible advantage*. *So no PPT attacker can distinguish between J*_*h*_*d*_,1_ *and J*_*h*_*d*_,2_ *with non-negligible advantage*.

From the Lemma 21, 22, we obtain the following equation

$$\begin{array}{c} \begin{array}{c} Adv_{A}^{G_{SF^{\prime \prime }}}-Adv_{A}^{G_{E-D}}\leq \sum_{h_{d}=1}^{q_{d}}(|Adv_{A}^{J_{h_{d}-1,2}}-Adv_{A}^{J_{h_{d},1}}|+|Adv_{A}^{J_{h_{d},1}}-Adv_{A}^{J_{h_{d},2}}\left|\right)\\ \leq O\left(q_{d}\right)\left(Adv_{B}^{A3}+Adv_{B}^{A4}\right) \end{array} \end{array}$$ (56)

**(2) Indistinguishability of** *G*_*E*−*D*_ **and** *G*_*ESF*_

We now prove the indistinguishability of *G*_*E*−*D*_ and *G*_*ESF*_ in a hybrid argument using polynomially many steps. We let *q*_*c*_ denote the number of ciphertext-type queries made by a PPT attacker A. Firstly we define hybrid games *L*_−1,1_, *L*_0,2_, *L*_0,3_, *L*_0,1_, *L*_1,2_, *L*_1,3_, *L*_1,1_⋯, *L*_*k*,2_, *L*_*k*,3_, *L*_*k*,1_, …, *L*_*q*_*c*_−1,2_, *L*_*q*_*c*_−1,3_, *L*_*q*_*c*_−1,1_, where *L*_−1,1_ = *G*_*E*−*D*_ and *L*_*q*_*c*_−1,1_ = *G*_*ESF*_. The games are formally defined as follows:

**Game** *L*_*k*,1_ This game *L*_*k*,1_ for 0 ≤ *k* ≤ *q*_*c*_ is almost the same as *G*_*E*−*D*_ except the generation of the challenge ciphertext. The challenge ciphertext of $\left(T^{*},I_{1}^{*},\ldots ,I_{q_{c}-1}^{*}\right)$ is generated as EST-2^k^-CT outputed by **EncryptESF-2**^k^.

**Game** *L*_*k*,2_ This game *L*_*k*,2_ for 0 ≤ *k* ≤ *q*_*c*_ − 1 is almost the same as *G*_*E*−*D*_ except the generation of the challenge ciphertext. The challenge ciphertext of $\left(T^{*},I_{1}^{*},\ldots ,I_{q_{c}-1}^{*}\right)$ is generated as EST-3^k^-CT outputed by **EncryptESF-3**^k^.

**Game** *L*_*k*,3_ This game *L*_*k*,3_ for 0 ≤ *k* ≤ *q*_*c*_ − 1 is almost the same as *G*_*E*−*D*_ except the generation of the challenge ciphertext. The challenge ciphertext of $\left(T^{*},I_{1}^{*},\ldots ,I_{q_{c}-1}^{*}\right)$ is generated as EST-4^k^-CT outputed by **EncryptESF-4**^k^.

We will define additional oracles ${Ò}_{i}^{*}$ for each *i* from 0 to *q*_*c*_ − 1, ${Ò}_{i}^{\prime }$ for each *i* from 0 to *q*_*c*_ − 1, and ${Ò}_{i}^{\prime \prime }$ for each *i* from 0 to *q*_*c*_ − 1.

**Oracle** ${Ò}_{i}^{*}$ This oracle initializes in the same way as *O*_5_, *O*_6_ and provides the attacker with initial group elements from the same distribution. It also responds to challenge key-type queries in the same way as *O*_5_, *O*_6_. It keeps a counter of ciphertext-type queries which is initially equal to zero. It increments this counter after each response to a ciphertext-type query. In response to the *j*^*th*^ ciphertext-type query for some $I_{j}^{*}$, if *j* ≤ *i*, it responds exactly like *O*_6_. If *j* > *i*, it responds exactly like *O*_5_. In particular, ${Ò}_{0}^{*}$ is identical to *O*_5_ and ${Ò}_{q}^{*}$ is identical to *O*_6_.

**Oracle** ${Ò}_{i}^{\prime }$ This oracle acts the same as ${Ò}_{i}^{*}$ except in its response to the *i*^*th*^ ciphertext-type query. For the *i*^*th*^ ciphertext-type query for identity I*, it chooses a random *t* ∈ *Z*_*N*_ and random elements *X*_3_, *Y*_3_ ∈ *G*_*p*_3__ and responds with:

$$\begin{array}{c} \left(w^{s}g_{2}^{\delta_{1}}v^{t}X_{3}^{\sigma_{1}},g^{t}X_{3},{\left(u^{I_{j}^{*}}h\right)}^{t}Y_{3}\right) \end{array}$$ (57)

If *i* = 0, the *i*^*th*^ ciphertext-type query is for time T*. It chooses a random *t*_0_ ∈ *Z*_*N*_ and random elements $X_{3}^{\prime },Y_{3}^{\prime }\in G_{p_{3}}$ and responds with:

$$\begin{array}{c} \left(w_{0}^{s}g_{2}^{\delta_{2}}v_{0}^{t_{0}}{X_{3}^{\prime }}^{\sigma_{2}},g^{t_{0}}X_{3}^{\prime },{\left(u_{0}^{T^{*}}h_{0}\right)}^{t_{0}}Y_{3}^{\prime }\right) \end{array}$$ (58)

**Oracle** ${Ò}_{i}^{\prime \prime }$ This oracle acts the same as ${Ò}_{i}^{*}$ except in its response to the ith ciphertext-type query. For the *i*^*th*^ ciphertext-type query for identity I*, it chooses a random *t* ∈ *Z*_*N*_ and random elements *X*_3_, *Y*_3_ ∈ *G*_*p*_3__ and responds with:

$$\begin{array}{c} \left(w^{s}g_{2}^{\delta_{1}}v^{t}X_{3}^{\sigma_{1}},g^{t}g_{2}^{t}X_{3},{\left(u^{I_{j}^{*}}h\right)}^{t}g_{2}^{t(a^{\prime }I_{j}^{*}+b^{\prime })}Y_{3}\right) \end{array}$$ (59)

If *i* = 0, the *i*^*th*^ ciphertext-type query is for time T*. It chooses a random *t*_0_ ∈ *Z*_*N*_ and random elements $X_{3}^{\prime },Y_{3}^{\prime }\in G_{p_{3}}$ and responds with:

$$\begin{array}{c} \left(w_{0}^{s}g_{2}^{\delta_{2}}v_{0}^{t_{0}}{X_{3}^{\prime }}^{\sigma_{2}},g^{t_{0}}g_{2}^{t_{0}}X_{3}^{\prime },{\left(u_{0}^{T^{*}}h_{0}\right)}^{t_{0}}g_{2}^{t_{0}\left(a^{\prime }I_{j}^{*}+b^{\prime }\right)}Y_{3}^{\prime }\right) \end{array}$$ (60)

**Lemma 23** *Under Assumptions 3*, *no PPT attacker can distinguish between* ${Ò}_{k-1}^{*}$ *and* ${Ò}_{k}^{\prime }$ *with non-negligible advantage*. *So no PPT attacker can distinguish between L*_*k*−1,1_ *and L*_*k*,2_ *with non-negligible advantage*.

**Lemma 24** *Under Assumptions 4*, *no PPT attacker can distinguish between* ${Ò}_{k}^{\prime }$ *and* ${Ò}_{k}^{\prime \prime }$ *with non-negligible advantage*. *So no PPT attacker can distinguish between L*_*k*,2_ *and L*_*k*,3_ *with non-negligible advantage*.

**Lemma 25** *Under Assumptions 3*, *no PPT attacker can distinguish between* ${Ò}_{k}^{\prime \prime }$ *and* ${Ò}_{k}^{*}$ *with non-negligible advantage*. *So no PPT attacker can distinguish between L*_*k*,3_ *and L*_*k*,1_ *with non-negligible advantage*.

From the Lemma 23, 24, 25, we obtain the following equation

$$\begin{array}{c} \begin{array}{c} Adv_{A}^{G_{E-D}}-Adv_{A}^{G_{ESF}}\leq \sum_{k=0}^{q_{c}-1}(|Adv_{A}^{L_{k-1,1}}-Adv_{A}^{L_{k,2}}|+|Adv_{A}^{L_{k,2}}-Adv_{A}^{L_{k,3}}|\\ +|Adv_{A}^{L_{k,3}}-Adv_{A}^{L_{k,1}}\left|\right)\\ \leq O\left(q_{c}\right)\left(2Adv_{B}^{A3}+Adv_{B}^{A4}\right) \end{array} \end{array}$$ (61)

**(3) Indistinguishability of** *G*_*ESF*_ **and** *G*_*SF*′_

For the security proof of the indistinguishability of *G*_*ESF*_ and *G*_*SF*′_, we define a sequence of games *G*_*ESF*−1_, *G*_*ESF*−2_, *G*_*ESF*−3_ to change the type of decryption keys from ESF-2 to ESF-4 and the type of ciphertexts from ESF-1 to ESF-5 and *G*_*ESF*−4_, *G*_*ESF*−5_, *G*_*ESF*−6_ to change the type of decryption keys to semi-functional and the type of ciphertexts back to semi-functional. In Table 8, we give the types of key in the queries and the challenge cipertext in every game, and the decryption situation according to the types of keys and ciphertexts.

**Table 8:** Defination of games between *G*_*ESF*_ and *G*_*SF*′_.

Games	Oracles	Keys in Queries			Challenge Ciphertext			
		SK	UK	DK	*Normal*	*SF*	*ESF* − 1	*ESF* − 5
*G*_*ESF*_	*O*_6_	*SF*	*SF*	*ESF* − 2			◯	
*G*_*ESF*−1_	*O*_6.1_	*SF*	*SF*	*ESF* − 3			◯	
*G*_*ESF*−2_	*O*_6.2_	*SF*	*SF*	*ESF* − 3				◯
*G*_*ESF*−3_	*O*_6.3_	*SF*	*SF*	*ESF* − 4				◯
*G*_*ESF*−4_	${Ő}_{q_{c}}^{*}$	*SF*	*SF*	*ESF* − 4			◯	
*G*_*ESF*−5_	${Ő}_{0}^{*}$	*SF*	*SF*	*ESF* − 4		◯		
*G*_*SF*′_	*O*_7_	*SF*	*SF*	*SF*		◯		

**G**_**ESF−1**_: The decryption keys are changed to ESF-3. The challenge ciphertext is still ESF-1. The secret keys and update keys are still semi-functional.

**G**_**ESF−2**_: The challenge ciphertext is changed to ESF-5. The secret keys and the update keys are still semi-functional. The decryption keys are still ESF-3.

**G**_**ESF−3**_: The decryption keys are changed to ESF-4. The challenge ciphertext is ESF-5. The secret keys and update keys are still semi-functional.

**G**_**ESF−4**_: The challenge ciphertext is changed to ESF-1. The secret keys and the update keys are semi-functional. The decryption keys are still ESF-4.

**G**_**ESF−5**_: The challenge ciphertext is changed to semi-functional. The secret keys and the update keys are semi-functional. The decryption keys are still ESF-4.

We firstly prove the indistinguishabilities between *G*_*ESF*_ to *G*_*ESF*−1_, *G*_*ESF*−1_ to *G*_*ESF*−5_. And then we prove the indistinguishability of *G*_*ESF*−5_ and *G*_*SF*′_.

**Indistinguishability of** *G*_*ESF*_ **and** *G*_*ESF*−1_. For the security proof of the indistinguishability of *G*_*ESF*_ and *G*_*ESF*−1_, we define games *G*_*F*,1_, ⋯, *G*_*F*,*h*_*d*__, …, *G*_*F*,*q*_*d*__ where *G*_*F*,0_ = *G*_*ESF*_ and *G*_*F*,*q*_*d*__ = *G*_*ESF*−1_, and *q*_*d*_ is the number of decryption key queries of an adversary. In the game *G*_*F*,*h*_ for 1 ≤ *h* ≤ *q*_*d*_, the challenge ciphertext is ESF-1, all (H)IBE private keys are semi-functional, the *i*^*st*^ queried decryption key where *i* ≤ *h* are of ESF-3, the remaining decryption keys with *i* > *h* are ESF-2.

**Oracle** *O*_6.1_ This oracle initializes in the same way as *O*_6_ and provides the attacker with initial group elements from the same distribution. Upon receiving a challenge decryption-key-type query for *I*, *T* ∈ *Z*_*n*_, it chooses *r*, *y*′, *r*′, *y*″ ∈ *Z*_*n*_ randomly, and also chooses $X_{2},X_{2}^{\prime },Y_{2},Y_{2}^{\prime }\in G_{p_{2}}$ and $X_{3},X_{3}^{\prime },Y_{3},Y_{3}^{\prime }\in G_{p_{3}}$ randomly. It returns the group elements

$$\begin{array}{c} \begin{array}{c} (w^{y^{\prime }}g_{3}^{y^{\prime }\psi_{1}},g^{y^{\prime }}g_{3}^{y^{\prime }},v^{y^{\prime }}{\left(u^{I}h\right)}^{r}X_{2}X_{3},g^{r}Y_{2}Y_{3},\\ w_{0}^{y^{\prime \prime }}g_{3}^{y^{\prime \prime }\psi_{2}},g^{y^{\prime \prime }}g_{3}^{y^{\prime \prime }},v_{0}^{y^{\prime \prime }}{\left(u_{0}^{T}h_{0}\right)}^{r^{\prime }}X_{2}^{\prime }X_{3}^{\prime },g^{r^{\prime }}Y_{2}^{\prime }Y_{3}^{\prime }) \end{array} \end{array}$$ (62)

to the attacker. It responds to a ciphertext-type query or a challenge (H)IBE-key-type query in the same way as *O*_6_.

**Lemma 26** *Under Assumptions 3*, *no PPT attacker can distinguish between O*_6_ *and O*_6.1_ *with non-negligible advantage*. *So no PPT attacker can distinguish between G*_*F*,*h*_*d*_−1_ *and G*_*F*,*h*_*d*__ *with non-negligible advantage*.

Let $Adv_{A}^{G_{F^{\prime },h}}$ be the advantage of A in a game *G*_*F*′,*h*_. From the Lemma 27, we obtain the following equation

$$\begin{array}{c} Adv_{A}^{G_{ESF}}-Adv_{A}^{G_{ESF-1}}\leq \sum_{h=1}^{q_{d}}\left(Adv_{A}^{G_{F^{\prime },h-1}}-Adv_{A}^{G_{F^{\prime },h}}\right)\leq O\left(q_{d}\right)\left(Adv_{B}^{A3}\right) \end{array}$$ (63)

**Indistinguishability of** *G*_*ESF*−1_ **and** *G*_*ESF*−2_. For the security proof of the indistinguishability of *G*_*ESF*−1_ and *G*_*ESF*−2_, we define the oracle below.

**Oracle** *O*_6.2_ This oracle initializes a bit differently from the other oracles. It fixes random elements *g*, *u*, *h*, *v*, *w*, *u*_0_, *h*_0_, *v*_0_, *w*_0_ ∈ *G*_*p*_1__, *g*_2_ ∈ *G*_*p*_2__, *g*_3_ ∈ *G*_*p*_3__. It chooses random exponents $s,\gamma ,\delta_{1},\delta_{2},y,y_{0},\psi ,\sigma_{1},\sigma_{2},a^{\prime },b^{\prime },t_{3},t_{3}^{\prime },t_{3}^{\prime \prime }\in Z_{N}$. It initially provides the attacker with the group elements:

$$\begin{array}{c} \begin{array}{c} (g,u,h,v,w,g^{s}{\left(g_{2}g_{3}\right)}^{\gamma },w^{y}{\left(g_{2}g_{3}\right)}^{y\psi },g^{y}{\left(g_{2}g_{3}\right)}^{y},v^{y}{\left(g_{2}g_{3}\right)}^{y\sigma_{1}},\\ u_{0},h_{0},v_{0},w_{0},w_{0}^{y_{0}}{\left(g_{2}g_{3}\right)}^{y_{0}\psi },g^{y_{0}}{\left(g_{2}g_{3}\right)}^{y_{0}},v_{0}^{y_{0}}{\left(g_{2}g_{3}\right)}^{y_{0}\sigma_{2}}) \end{array} \end{array}$$ (64)

What differs from the previous oracles here is the added $g_{3}^{\gamma }$ and term: notice that this is uniformly random in *G*_*p*_3__, since *γ* is random modulo *p*_3_ (and uncorrelated from its value modulo *p*_2_). This oracle answers the challenge-key type query in the same way as *O*_6.1_. To answer a ciphertext-type query for *I*, it chooses random values *t* ∈ *Z*_*N*_ and responds with:

$$\begin{array}{c} \left(w^{s}g_{2}^{\delta_{1}}v^{t}g_{2}^{\sigma_{1}t}g_{3}^{t_{3}},g^{t}g_{2}^{t}g_{3}^{t_{3}^{\prime }},{\left(u^{I}h\right)}^{t}g_{2}^{t(a^{\prime }I+b^{\prime })}g_{3}^{t_{3}^{\prime \prime }}\right) \end{array}$$ (65)

To answer a ciphertext-type query for *T*, it chooses random values *t* ∈ *Z*_*N*_ and responds with:

$$\begin{array}{c} \left(w_{0}^{s}g_{2}^{\delta_{2}}v_{0}^{t}g_{2}^{\sigma_{2}t}g_{3}^{t_{3}},g^{t}g_{2}^{t}g_{3}^{t_{3}^{\prime }},{\left(u_{0}^{T}h_{0}\right)}^{t}g_{2}^{t(a^{\prime }T+b^{\prime })}g_{3}^{t_{3}^{\prime \prime }}\right) \end{array}$$ (66)

It is crucial to note that these *G*_*p*_3__ terms arethe same for each ciphertext-type query response.

**Lemma 27** *Under Assumptions 4*, *no PPT attacker can distinguish between O*_6.1_ *and O*_6.2_ *with non-negligible advantage*. *So no PPT attacker can distinguish between G*_*ESF*−1_ *and G*_*ESF*−2_ *with non-negligible advantage*.

From the Lemma 27, we obtain the following equation

$$\begin{array}{c} Adv_{A}^{G_{ESF-1}}-Adv_{A}^{G_{ESF-2}}\leq \left(Adv_{B}^{A4}\right) \end{array}$$ (67)

**Indistinguishability of** *G*_*ESF*−2_ **and** *G*_*ESF*−3_: For the security proof of the indistinguishability of *G*_*ESF*−2_ and *G*_*ESF*−3_, we define a sequence of games additional hybrid games *G*_*F*−2,1_, …, *G*_*F*−2,*h*_, …, *G*_*F*−2,*q*_*d*__, where *G*_*ESF*−2_ = *G*_*F*−2,0_ and *q*_*d*_ is the number of decryption key queries of an adversary. In the game *G*_*F*−2,*h*_ for 1 ≤ *h* ≤ *q*_*d*_, the challenge ciphertext is ESF-5, all (H)IBE private keys are semi-functional, the *i*^*st*^ queried decryption key where *i* ≤ *h* are of ESF-4, the remaining decryption keys with *i* > *h* are ESF-3.

**Oracle** *O*_6.3_ This oracle initializes in the same way with *O*_6.2_ and provides the attacker the same initial elements as *O*_6.2_. This oracle answers the ciphertext-type query and (H)IBE key- type query in the same way as *O*_6.2_. To answer a challenge decryption key type query for *I*, *I*, it chooses random values $y,r,y^{\prime },r^{\prime }\in Z_{N},X_{2},Y_{2},X_{2}^{\prime },Y_{2}^{\prime }\in G_{p_{2}}$ randomly, and $X_{3},Y_{3},X_{3}^{\prime },Y_{3}^{\prime }\in G_{p_{3}}$ and responds with:

$$\begin{array}{c} \begin{array}{c} (w^{y}{\left(g_{2}g_{3}\right)}^{y\psi_{1}},g^{y}{\left(g_{2}g_{3}\right)}^{y},v^{y}{\left(u^{I}h\right)}^{r}X_{2}X_{3},g^{r}Y_{2}Y_{3},\\ w_{0}^{y^{\prime }}{\left(g_{2}g_{3}\right)}^{y^{\prime }\psi_{2}},g^{y^{\prime }}{\left(g_{2}g_{3}\right)}^{y^{\prime }},v_{0}^{y^{\prime }}{\left(u_{0}^{T}h_{0}\right)}^{r^{\prime }}X_{2}^{\prime }X_{3}^{\prime },g^{r^{\prime }}Y_{2}^{\prime }Y_{3}^{\prime }) \end{array} \end{array}$$ (68)

**Lemma 28** *Under Assumptions 4*, *no PPT attacker can distinguish between O*_6.2_ *and O*_6.3_ *with non-negligible advantage*. *So no PPT attacker can distinguish between G*_*F*−2,*h*_ *and G*_*F*−2,*h*+1_ *with non-negligible advantage*.

Let $Adv_{A}^{G_{F-2,h}}$ be the advantage of A in a game *G*_*F*−2,*h*_. From the Lemma 28, we obtain the following equation

$$\begin{array}{c} Adv_{A}^{G_{ESF-2}}-Adv_{A}^{G_{ESF-3}}\leq \sum_{h=1}^{q_{d}}\left(Adv_{A}^{G_{F-2,h-1}}-Adv_{A}^{G_{F-2,h}}\right)\leq O\left(q_{d}\right)\left(Adv_{B}^{A4}\right) \end{array}$$ (69)

**Indistinguishability of** *G*_*ESF*−3_ **and** *G*_*ESF*−4_. For the security proof of the indistinguishability of *G*_*ESF*−3_ and *G*_*ESF*−4_, we define the oracle below.

**Oracle** ${Ő}_{i}^{*}$ This oracle initializes in the same way as ${Ò}_{i}^{*}$, and provides the attacker with initial group elements from the same distribution. It also responds to a ciphertext-type query as same as ${Ò}_{i}^{*}$. It responds to the decryption-key-type and (H)IBE-key-type queries in the same way as *O*_6.3_.

**Lemma 29** *Under Assumptions 4*, *no PPT attacker can distinguish between O*_6.3_ *and* ${Ő}_{q_{c}}^{*}$ *with non-negligible advantage*. *So no PPT attacker can distinguish between G*_*ESF*−3_ *and G*_*ESF*−4_ *with non-negligible advantage*.

From the Lemma 29, we obtain the following equation

$$\begin{array}{c} Adv_{A}^{G_{ESF-3}}-Adv_{A}^{G_{ESF-4}}\leq \left(Adv_{B}^{A4}\right) \end{array}$$ (70)

**Indistinguishability of** *G*_*ESF*−4_ **and** *G*_*ESF*−5_. We now prove the indistinguishability of *G*_*ESF*−4_ and *G*_*ESF*−5_ in a hybrid argument using polynomially many steps. We let *q*_*c*_ denote the number of ciphertext-type queries made by a PPT attacker A. Firstly we define hybrid games $L_{-1,1}^{\prime },L_{0,2}^{\prime },L_{0,3}^{\prime },L_{0,1}^{\prime }$, $L_{1,2}^{\prime },L_{1,3}^{\prime },L_{1,1}^{\prime }\ldots$, $L_{k,2}^{\prime },L_{k,3}^{\prime },L_{k,1}^{\prime },\ldots ,L_{q_{c}-1,2}^{\prime },L_{q_{c}-1,3}^{\prime },L_{q_{c}-1,1}^{\prime }$, where $L_{-1,1}^{\prime }=G_{ESF-4}$ and $L_{q_{c}-1,1}^{\prime }=G_{ESF-5}$. The games are formally defined as follows:

**Game** $L_{k,1}^{\prime }$ This game $L_{k,1}^{\prime }$ for 0 ≤ *k* ≤ *q*_*c*_ is almost the same as *G*_*ESF*−4_ except the generation of the challenge ciphertext. The challenge ciphertext of $\left(T^{*},I_{1}^{*},\ldots ,I_{q_{c}-1}^{*}\right)$ is generated as EST-2^k^-CT outputed by **EncryptESF-2**^k^.

**Game** $L_{k,2}^{\prime }$ This game $L_{k,2}^{\prime }$ for 0 ≤ *k* ≤ *q*_*c*_ − 1 is almost the same as *G*_*ESF*−4_ except the generation of the challenge ciphertext. The challenge ciphertext of $\left(T^{*},I_{1}^{*},\ldots ,I_{q_{c}-1}^{*}\right)$ is generated as EST-3^k^-CT outputed by **EncryptESF-3**^k^.

**Game** $L_{k,3}^{\prime }$ This game $L_{k,3}^{\prime }$ for 0 ≤ *k* ≤ *q*_*c*_ − 1 is almost the same as *G*_*ESF*−4_ except the generation of the challenge ciphertext. The challenge ciphertext of $\left(T^{*},I_{1}^{*},\ldots ,I_{q_{c}-1}^{*}\right)$ is generated as EST-4^k^-CT outputed by **EncryptESF-4**^k^.

We will define additional oracles ${Ő}_{i}^{\prime }$ for each *i* from 0 to *q*_*c*_ − 1, and ${Ő}_{i}^{\prime \prime }$ for each *i* from 0 to *q*_*c*_ − 1.

**Oracle** ${Ő}_{i}^{\prime }$ This oracle acts the same as ${Ő}_{i}^{*}$ except that its response to the ciphertext-type query is as same as ${Ò}_{i}^{\prime }$.

**Oracle** ${Ő}_{i}^{\prime \prime }$ This oracle acts the same as ${Ő}_{i}^{*}$ except that its response to the ciphertext-type query is as same as ${Ò}_{i}^{\prime \prime }$.

**Lemma 30** *Under Assumptions 3*, *no PPT attacker can distinguish between* ${Ő}_{k}^{*}$ *and* ${Ő}_{k}^{\prime \prime }$ *with non-negligible advantage*. *So no PPT attacker can distinguish between* $L_{k,1}^{\prime }$ *and* $L_{k,3}^{\prime }$ *with non-negligible advantage*.

**Lemma 31** *Under Assumptions 4*, *no PPT attacker can distinguish between* ${Ő}_{k}^{\prime \prime }$ *and* ${Ő}_{k}^{\prime }$ *with non-negligible advantage*. *So no PPT attacker can distinguish between* $L_{k,3}^{\prime }$ *and* $L_{k,2}^{\prime }$ *with non-negligible advantage*.

**Lemma 32** *Under Assumptions 3*, *no PPT attacker can distinguish between* ${Ő}_{k}^{\prime }$ *and* ${Ő}_{k-1}^{*}$ *with non-negligible advantage*. *So no PPT attacker can distinguish between* $L_{k,2}^{\prime }$ *and* $L_{k-1,1}^{\prime }$ *with non-negligible advantage*.

Let $Adv_{A}^{L_{k,1}^{\prime }}$, $Adv_{A}^{L_{k,2}^{\prime }}$ and $Adv_{A}^{L_{k,3}^{\prime }}$ be the advantage of A in the games $L_{k,1}^{\prime }$, $L_{k,2}^{\prime }$ and $L_{k,3}^{\prime }$. From the Lemma 30, 31, 32, we obtain the following equation

$$\begin{array}{c} \begin{array}{c} Adv_{A}^{G_{ESF-4}}-Adv_{A}^{G_{ESF-5}}\leq \sum_{k=0}^{q_{c}-1}(|Adv_{A}^{L_{k-1,1}^{\prime }}-Adv_{A}^{L_{k,2}^{\prime }}|+|Adv_{A}^{L_{k,2}^{\prime }}-Adv_{A}^{L_{k,3}^{\prime }}|\\ +|Adv_{A}^{L_{k,3}^{\prime }}-Adv_{A}^{L_{L,1}^{\prime }}\left|\right)\\ \leq q_{c}\left(2Adv_{B}^{A3}+Adv_{B}^{A4}\right) \end{array} \end{array}$$ (71)

**Indistinguishability of** *G*_*ESF*−5_ **and** *G*_*SF*′_. For the security proof of the indistinguishability of *G*_*ESF*−5_ and *G*_*SF*′_, we define hybrid games $J_{1,1}^{\prime }$, $J_{1,2}^{\prime },\ldots ,J_{h,1}^{\prime },J_{h,2}^{\prime },\ldots ,J_{q_{d},1}^{\prime },J_{q_{d},2}^{\prime }$ where $J_{0,2}^{\prime }=G_{ESF-5}$ and $J_{q_{d},2}^{\prime }=G_{SF^{\prime }}$, and *q*_*d*_ is the number of decryption key queries of an adversary. The oracle and games are formally defined as follows:

**Oracle** *O*_13/2_ This oracle initializes in the same way as ${Ő}_{0}^{*}$ and provides the attacker with initial group elements from the same distribution. Upon receiving a challenge decryption-key-type query for *I*, *T* ∈ *Z*_*n*_, it chooses *r*, *y*′, *r*′, *y*″ ∈ *Z*_*n*_ randomly, and also chooses $X_{2},Y_{2},X_{2}^{\prime },Y_{2}^{\prime }\in G_{p_{2}}$ and $X_{3},Y_{3},X_{3}^{\prime },Y_{3}^{\prime }\in G_{p_{3}}$ randomly. It returns the group elements

$$\begin{array}{c} \begin{array}{c} (w^{y^{\prime }}{\left(g_{2}g_{3}\right)}^{y^{\prime }\psi_{1}},g^{y^{\prime }}{\left(g_{2}g_{3}\right)}^{y^{\prime }},v^{y^{\prime }}{\left(u^{I}h\right)}^{r}X_{2}X_{3},g^{r}Y_{3},\\ w_{0}^{y^{\prime \prime }}{\left(g_{2}g_{3}\right)}^{y^{\prime \prime }\psi_{2}},g^{y^{\prime \prime }}{\left(g_{2}g_{3}\right)}^{y^{\prime \prime }},v_{0}^{y^{\prime \prime }}{\left(u_{0}^{T}h_{0}\right)}^{r^{\prime }}X_{2}^{\prime }X_{3}^{\prime },g^{r^{\prime }}Y_{3}^{\prime }) \end{array} \end{array}$$ (72)

to the attacker. It responds to a ciphertext-type query or a challenge (H)IBE-key-type query in the same way as ${Ő}_{0}^{*}$.

**Game** $J_{h,1}^{\prime }$ This game $J_{h,1}^{\prime }$ for 1 ≤ *h* ≤ *q*_*d*_ is almost the same as *G*_*ESF*−5_ except the generation of decryption keys. The *i*^*st*^ queried decryption key is generated as follows:

1. *i* < *h*: It generates a semi-functional *DK*_*ID*|_*k*_,*T*_.
2. *i* = *h*: It generates a ESF-5 *DK*_*ID*|_*k*_,*T*_.
3. *i* > *h*: It generates a ESF-4 *DK*_*ID*|_*k*_,*T*_.

**Game** $J_{h,2}^{\prime }$ This game $J_{h,1}^{\prime }$ for 1 ≤ *h* ≤ *q*_*d*_ is almost the same as $J_{h,1}^{\prime }$ except the generation of the *h*^*st*^ queried decryption key is generated as a semi-functional *DK*_*ID*|_*k*_,*T*_.

**Lemma 33** *Under Assumptions 4*, *no PPT attacker can distinguish between* ${Ő}_{0}^{*}$ *and O*_13/2_ *with non-negligible advantage*. *So no PPT attacker can distinguish between* $J_{h-1,2}^{\prime }$ *and* $J_{h,1}^{\prime }$ *with non-negligible advantage*.

**Lemma 34** *Under Assumptions 3*, *no PPT attacker can distinguish between O*_13/2_ *and O*_7_ *with non-negligible advantage*. *So no PPT attacker can distinguish between* $J_{h,1}^{\prime }$ *and* $J_{h,2}^{\prime }$ *with non-negligible advantage*.

Let $Adv_{A}^{G_{E^{\prime },h}}$ be the advantage of A in a game *G*_*E*′,*h*_. From the Lemma 33, 34, we obtain the following equations

$$\begin{array}{c} \begin{array}{c} Adv_{A}^{G_{ESF-5}}-Adv_{A}^{G_{SF^{\prime }}}\leq \sum_{h_{c}=1}^{q_{d}}(|Adv_{A}^{J_{h,1}^{\prime }}-Adv_{A}^{J_{h,2}^{\prime }}|+|Adv_{A}^{J_{h-1,2}^{\prime }}-Adv_{A}^{J_{h,1}^{\prime }}\left|\right)\\ \leq O\left(q_{d}\right)\left(Adv_{B}^{A3}+Adv_{B}^{A4}\right) \end{array} \end{array}$$

So we obtain the following equation

$$\begin{array}{c} \begin{array}{c} Adv_{A}^{G_{ESF}}-Adv_{A}^{G_{SF^{\prime }}}\leq |(Adv_{A}^{G_{ESF}}-Adv_{A}^{G_{ESF-1}}|+\\ \;\sum_{i=1}^{5}|(Adv_{A}^{G_{ESF-i}}-Adv_{A}^{G_{ESF-i+1}}|+|(Adv_{A}^{G_{ESF-5}}-Adv_{A}^{G_{SF^{\prime }}}|\\ \leq O\left(q_{d}\right)\left(Adv_{B}^{A3}\right)+\left(Adv_{B}^{A4}\right)+O\left(q_{d}\right)\left(Adv_{B}^{A4}\right)+\left(Adv_{B}^{A4}\right)+\\ q_{c}\left(2Adv_{B}^{A3}+Adv_{B}^{A4}\right)+O\left(q_{d}\right)\left(Adv_{B}^{A3}+Adv_{B}^{A4}\right)\\ \leq \left(O\left(q_{d}\right)+O\left(l\right)\right)\left(Adv_{B}^{A3}+Adv_{B}^{A4}\right) \end{array} \end{array}$$ (73)

According to the equations Eqs 56, 61, 73, we obtain the following equation

$$\begin{array}{c} \begin{array}{c} Adv_{A}^{G_{SF^{\prime \prime }}}-Adv_{A}^{G_{SF^{\prime }}}\leq |(Adv_{A}^{G_{SF^{\prime \prime }}}-Adv_{A}^{G_{E-D}}|\\ \;+|(Adv_{A}^{G_{E-D}}-Adv_{A}^{G_{ESF}}|+|(Adv_{A}^{G_{ESF}}-Adv_{A}^{G_{SF^{\prime }}}|\\ \;\leq \left(O\left(q_{d}\right)+O\left(l\right)\right)\left(Adv_{B}^{A3}+Adv_{B}^{A4}\right) \end{array} \end{array}$$ (74)

### 4.6 Indistinguishability of *G*_*C*_ and *G*_*C*′_ and Indistinguishability of *G*_*SF*′_ and *G*_*SF*_

**Lemma 35** *Under Assumptions 3 and 4*, *for any PPT attacker* A, *the difference in* A’s *advantage between G*_*θ*_ *and G*_*θ*′_ *is negligible*, *where θ* ∈ {*C*, *SF*}.

***Proof*** We suppose there exists a PPT attacker A and a symbol of *θ* ∈ {*C*, *SF*} such that A’s advantage changes non-negligibly between Game RHIBE_*θ*_ and Game RHIBE_*θ*′_. We will either create a PPT algorithm B that breaks Assumption 3 with non-negligible advantage or a PPT algorithm B that breaks Assumption 4 with non-negligible advantage.

While playing Game RHIBE_*θ*_ under Type-1 adversary, A produces two values *I*, *I*′ ∈ *Z*_*n*_ which are unequal modulo *n* but are equal modulo *p*_3_, with non-negligible probability. We let *A* denote *gcd*(*I* − *I*′, *n*), and we let *B* denote *n*/*A*. We then have that *p*_3_ divides *A*, and *B* ≠ 1.

While playing Game RHIBE_*θ*_ under Type-2 adversary, A produces two values *T*, *T*′ ∈ *Z*_*n*_ which are unequal modulo *n* but are equal modulo *p*_3_, with non-negligible probability. We let *A* denote *gcd*(*T* − *T*′, *n*), and we let *B* denote *n*/*A*. We then have that *p*_3_ divides *A*, and *B* ≠ 1.

We consider two possible cases: 1) *p*_1_ divides *B* and 2) *A* = *p*_1_*p*_3_, *B* = *p*_2_. At least one of these cases must occur with non-negligible probability.

If case 1) occurs with non-negligible probability, we can create a B which breaks Assumption 3 with non-negligible advantage. B receives *g*, *g*_2_, *X*_1_ *X*_3_, *T*. It can use these terms to simulate Game RHIBE_*β*_ with A as follows. It picks values *a*, *b*, *c*, *d*, *a*_0_, *b*_0_, *c*_0_, *d*_0_ ∈ *Z*_*N*_ uniformly at random and sets *u* = *g*^*a*^, *h* = *g*^*b*^, *v* = *g*^*c*^, *w* = *g*^*d*^, $u_{0}=g^{a_{0}}$, $h_{0}=g^{b_{0}}$, $v_{0}=g^{c_{0}}$, $w_{0}=g^{d_{0}}$, and gives A the following public parameters:

$$\begin{array}{c} \begin{array}{c} PP=(g,u,h,v,w,u_{0},h_{0},v_{0},w_{0},\Omega =e{\left(g,g\right)}^{\alpha }) \end{array} \end{array}$$

We note that B knows the master secret key *α*, so it can easily make normal secret keys, normal update keys and normal decryption keys. Since B also knows *g*_2_, it can easily make semi-functional ciphertexts. So B can play Game RHIBE_*C*_ and Game RHIBE_*C*′_ with A.

To make the three kinds of semi-functional keys, B uses *X*_1_, *X*_3_ and *g*_2_. More precisely, to make a semi-functional decryption key for *T* and (*I*_1_, ⋯, *I*_*j*_), it also chooses random values $y_{1},\cdots ,y_{j-1},y_{j}^{\prime },r_{0},r_{1},\cdots \ldots ,r_{j}\in Z_{n}$. It forms the decryption key as:

$$\begin{array}{c} \begin{array}{c} D_{0,0}=g^{\alpha -\sum_{i=1}^{j}\lambda_{i}}{\left(X_{1}X_{3}g_{2}\right)}^{d_{0}y_{0,\theta }^{\prime }},D_{0,1}={\left(X_{1}X_{3}g_{2}\right)}^{y_{0,\theta }^{\prime }},D_{0,2}=g^{r_{0,\theta }},\\ D_{0,3}={\left(X_{1}X_{3}g_{2}\right)}^{c_{0}y_{0,\theta }^{\prime }}{\left(u_{0}^{T}h_{0}\right)}^{r_{0,\theta }}\\ D_{i,0}=g^{\lambda_{i}}w^{y_{i,\theta }},D_{i,1}=g^{y_{i,\theta }},D_{i,2}=g^{r_{i,\theta }},U_{i,3}={\left(u^{I_{i}}h\right)}^{r_{i,\theta }}v^{y_{i,\theta }},i\in \left\{1,\cdots ,j-1\right\}\\ D_{j,0}=g^{\lambda_{j}}{\left(X_{1}X_{3}g_{2}\right)}^{dy_{j,\theta }^{\prime }},D_{j,1}={\left(X_{1}X_{3}g_{2}\right)}^{y_{j,\theta }^{\prime }},D_{j,2}=g^{r_{j,\theta }},\\ D_{j,3}={\left(X_{1}X_{3}g_{2}\right)}^{cy_{j,\theta }^{\prime }}{\left(u^{I_{j}}h\right)}^{r_{j,\theta }} \end{array} \end{array}$$

To make the semi-functional update key of (*I*_1_, ⋯, *I*_*j*−1_) and *T* for each *θ* with its value *γ*_*θ*_ in KUNode(*BT*_*ID*|_*j*−1__, *T*, *RL*_*ID*|_*j*−1__), B chooses random values $y_{1,\theta },\cdots ,y_{j-2,\theta },y_{0,\theta }^{\prime },r_{0,\theta },r_{1,\theta },\cdots ,r_{j-1,\theta }\in Z_{n}$. B forms the challenge update key for A as:

$$\begin{array}{c} \begin{array}{c} U_{0,0}=g^{\alpha -\gamma_{\theta }-\sum_{i=1}^{j-1}\lambda_{i}}{\left(X_{1}X_{3}g_{2}\right)}^{d_{0}y_{0,\theta }^{\prime }},U_{0,1}={\left(X_{1}X_{3}g_{2}\right)}^{y_{0,\theta }^{\prime }},U_{0,2}=g^{r_{0,\theta }},\\ U_{0,3}={\left(X_{1}X_{3}g_{2}\right)}^{c_{0}y_{0,\theta }^{\prime }}{\left(u_{0}^{T}h_{0}\right)}^{r_{0,\theta }}\\ U_{i,0}=g^{\lambda_{i}}w^{y_{i,\theta }},U_{i,1}=g^{y_{i,\theta }},U_{i,2}=g^{r_{i,\theta }},U_{i,3}={\left(u^{I_{i}}h\right)}^{r_{i,\theta }}v^{y_{i,\theta }},i\in \left\{1,\cdots ,j-1\right\} \end{array} \end{array}$$

To make the semi-functional secret key of (*I*_1_, ⋯, *I*_*j*_) and *T* for each *θ* with its value *γ*_*θ*_ in Path(*ID*|_*j*_), B chooses random values $y_{1,\theta },\cdots ,y_{j-1,\theta },y_{j,\theta }^{\prime },$ *r*_1, *θ*_, ⋯, *r*_*j*, *θ*_ ∈ *Z*_*n*_. B forms the challenge secret key for A as:

$$\begin{array}{c} \begin{array}{c} K_{i,0}=g^{\lambda_{i}}w^{y_{i,\theta }},K_{i,1}=g^{y_{i,\theta }},K_{i,2}=g^{r_{i,\theta }},K_{i,3}={\left(u^{I_{i}}h\right)}^{r_{i,\theta }}v^{y_{i,\theta }},i\in \left\{1,\cdots ,j-1\right\}\\ K_{j,0}=g^{\gamma_{\theta }-\sum_{i=1}^{j-1}\lambda_{i}}{\left(X_{1}X_{3}g_{2}\right)}^{dy_{j,\theta }^{\prime }},K_{j,1}={\left(X_{1}X_{3}g_{2}\right)}^{y_{j,\theta }^{\prime }},K_{j,2}=g^{r_{j,\theta }},\\ K_{j,3}={\left(X_{1}X_{3}g_{2}\right)}^{cy_{j,\theta }^{\prime }}{\left(u^{I_{j}}h\right)}^{r_{j,\theta }} \end{array} \end{array}$$

So B can play Game RHIBE_*SF*_ and Game RHIBE_*SF*′_ with A. Now, if A fails to produce *I*, *I*′ or *T*, *T*′ such that *gcd*(*I* − *I*′, *n*) = *A* or *gcd*(*T* − *T*′, *n*) = *A* is divisible by *p*_3_ and *p*_1_ divides *B* = *n*/*A*, then B guesses randomly. However, with non-negligible probability, A will produce such an *I*, *I*′ or *T*, *T*′. B can detect this by computing *A* = *gcd*(*I* − *I*′, *n*) or *A* = *gcd*(*T* − *T*′, *n*) and *B* = *n*/*A*, checking that *g*^*B*^ is the identity element (this will occur only if *p*_1_ divides B since g has order *p*_1_ in G) and checking that (*X*_1_ *X*_3_)^*B*^ ≠ 1 (this confirms that *p*_3_ does not divide B, hence it must divide A). When B detects this situation, it can test whether *T* ∈ *G*_*p*_1__ or *T* ∈ *G*_*p*_1_*p*_3__ by testing if *T*^*B*^ is 1. If *T*^*B*^ = 1 holds, then *T* ∈ *G*_*p*_1__. If *T*^*B*^ ≠ 1, then *T* ∈ *G*_*p*_1_*p*_3__. Thus, B achieves non-negligible advantage in breaking Assumption 3.

If case 2) occurs with non-negligible probability, we can create a B which breaks Assumption 4 with non-negligible advantage. B receives *g*, *g*_3_, *X*_1_ *X*_2_, *Y*_2_ *Y*_3_, *T*. It can use these terms to simulate Game RHIBE_*θ*_ with A as follows. It gives A the public parameters like in the case 1. We note that B knows the master secret key *α*, so it can easily make normal keys.

To make a semi-functional ciphertext for *T* and $\left(I_{1}^{*},\cdots ,I_{l}^{*}\right)$ and message *M*, B chooses random values *t*_0_, *t*_1_, ⋯, *t*_*l*_ ∈ *Z*_*n*_ and forms the ciphertext as:

$$\begin{array}{c} \begin{array}{c} C=Me{\left(X_{1}X_{2},g\right)}^{\alpha },C_{0}=X_{1}X_{2},\\ C_{0,1}={\left(X_{1}X_{2}\right)}^{d_{0}}g^{c_{0}t_{0}},C_{0,2}=g^{a_{0}T^{*}+b_{0}},C_{0,3}=g^{t_{0}}\\ C_{i,1}={\left(X_{1}X_{2}\right)}^{d}g^{ct_{i}},C_{i,2}=g^{aI_{i}^{*}+b},C_{i,3}=g^{t_{i}},i\in \left\{1,\cdots ,l\right\} \end{array} \end{array}$$

We note that this will set *σ*_1_ = *c*_1_ modulo *p*_2_ and *σ*_2_ = *c*_2_ modulo *p*_2_. To make a semi-functional decryption key for (*I*_1_, ⋯, *I*_*j*_), B chooses random values *y*_0_, *y*_1_, ⋯, *y*_*j*_, *r*_0_, *r*_1_, ⋯, *r*_*j*_ ∈ *Z*_*n*_. It forms the key as:

$$\begin{array}{c} \begin{array}{c} D_{0,0}=g^{\alpha -\sum_{i=1}^{j}\lambda_{i}}w_{0}^{y_{0}}{\left(Y_{2}Y_{3}\right)}^{y_{0,\theta }\psi_{2}},D_{0,1}=g^{y_{0}}{\left(Y_{2}Y_{3}\right)}^{y_{0}},D_{0,2}=g^{r_{0}},\\ D_{0,3}=v_{0}^{y_{0}}{\left(Y_{2}Y_{3}\right)}^{y_{0}\sigma_{2}}{\left(u_{0}^{T}h_{0}\right)}^{r_{0}}\\ D_{i,0}=g^{\lambda_{i}}w^{y_{i}},D_{i,1}=g^{y_{i}},D_{i,2}=g^{r_{i}},D_{i,3}={\left(u^{I_{i}}h\right)}^{r_{i}}v^{y_{i}},i\in \left\{1,\cdots ,j-1\right\}\\ D_{j,0}=g^{\lambda_{j}}w^{y_{j,\theta }}{\left(Y_{2}Y_{3}\right)}^{y_{j}\psi_{1}},K_{j,1}=g^{y_{j}}{\left(Y_{2}Y_{3}\right)}^{y_{j}},D_{j,2}=g^{r_{j}},\\ D_{j,3}=v^{y_{j}}{\left(Y_{2}Y_{3}\right)}^{y_{j}\sigma_{1}}{\left(u^{I_{j}}h\right)}^{r_{j}} \end{array} \end{array}$$

To make the semi-functional update key of (*I*_1_, ⋯, *I*_*j*−1_) and *T* for each *θ* with its value *γ*_*θ*_ in KUNode(*BT*_*ID*|_*j*−1__, *T*, *RL*_*ID*|_*j*−1__), B chooses random values *y*_0,*θ*_, *y*_1,*θ*_, ⋯, *y*_*j*−1,*θ*_, *r*_0,*θ*_, *r*_1,*θ*_, ⋯, *r*_*j*−1,*θ*_ ∈ *Z*_*n*_. B forms the challenge update key for A as:

$$\begin{array}{c} \begin{array}{c} U_{0,0}=g^{\alpha -\gamma_{\theta }-\sum_{i=1}^{j-1}\lambda_{i}}w_{0}^{y_{0,\theta }}{\left(Y_{2}Y_{3}\right)}^{y_{0,\theta }\psi_{2}},U_{0,1}=g^{y_{0,\theta }}{\left(Y_{2}Y_{3}\right)}^{y_{0,\theta }},U_{0,2}=g^{r_{0,\theta }},\\ U_{0,3}=v_{0}^{y_{0,\theta }}{\left(Y_{2}Y_{3}\right)}^{y_{0,\theta }\sigma_{2}}{\left(u_{0}^{T}h_{0}\right)}^{r_{0,\theta }}\\ U_{i,0}=g^{\lambda_{i}}w^{y_{i,\theta }},U_{i,1}=g^{y_{i,\theta }},U_{i,2}=g^{r_{i,\theta }},U_{i,3}={\left(u^{I_{i}}h\right)}^{r_{i,\theta }}v^{y_{i,\theta }},i\in \left\{1,\cdots ,j-1\right\} \end{array} \end{array}$$

To make the semi-functional secret key of (*I*_1_, ⋯, *I*_*j*_) and *T* for each *θ* with its value *γ*_*θ*_ in Path(*ID*|_*j*_), B chooses random values *y*_1,*θ*_, ⋯, *y*_*j*,*θ*_, *r*_1,*θ*_, ⋯, *r*_*j*,*θ*_ ∈ *Z*_*n*_. B forms the challenge secret key for A as:

$$\begin{array}{c} \begin{array}{c} K_{i,0}=g^{\lambda_{i}}w^{y_{i,\theta }},K_{i,1}=g^{y_{i,\theta }},K_{i,2}=g^{r_{i,\theta }},K_{i,3}={\left(u^{I_{i}}h\right)}^{r_{i,\theta }}v^{y_{i,\theta }},i\in \left\{1,\cdots ,j-1\right\}\\ K_{j,0}=g^{\gamma_{\theta }-\sum_{i=1}^{j-1}\lambda_{i}}w^{y_{j,\theta }}{\left(Y_{2}Y_{3}\right)}^{y_{j,\theta }\psi_{1}},K_{j,1}=g^{y_{j,\theta }}{\left(Y_{2}Y_{3}\right)}^{y_{j,\theta }},K_{j,2}=g^{r_{j,\theta }},\\ K_{j,3}=v^{y_{j,\theta }}{\left(Y_{2}Y_{3}\right)}^{y_{j,\theta }\sigma_{1}}{\left(u^{I_{j}}h\right)}^{r_{j,\theta }} \end{array} \end{array}$$

We note that the semi-functional ciphertext and keys are well-distributed, and share the common value of *σ*_1_ = *c*_1_ modulo *p*_2_ and *σ*_2_ = *c*_2_ modulo *p*_2_ as required. We note that the *G*_*p*_2__ terms on the ciphertext are random because the value of *d* modulo *p*_2_ and *d*_0_ modulo *p*_2_ does not appear elsewhere.

Now, if A fails to produce *I*, *I*′ such that *gcd*(*I* − *I*′, *n*) = *A* or *T*, *T*′ such that *gcd*(*T* − *T*′, *n*) = *A*, where *A* = *p*_1_ *p*_3_ and *B* = *p*_2_, then B guesses randomly. However, with non-negligible probability, A will produce such an *I*, *I*′ or *T*, *T*′. B can detect this by computing *A*, *B* and testing that *g*^*B*^ and $g_{3}^{B}$ are not the identity element (this confirms that *B* = *p*_2_, since it demonstrates the *p*_1_ and *p*_3_ do not divide *B*). Now, B can learn whether *T* has a *G*_*p*_2__ component or not by testing if *T*^*A*^ is the identity element or not. If it is not, then *T* has a *G*_*p*_2__ component. Thus, B achieves non-negligible advantage in breaking Assumption 4.

### 4.7 Indistinguishability of *G*_*Real*_ and *G*_*C*_

**Lemma 36** *If the Assumption 1 holds*, *then no polynomial-time adversary can distinguish G*_*Real*_ *and G*_*C*_.

***Proof***. We assume there is a PPT attacker A such that A achieves a non-negligible difference in advantage between Game *G*_*Real*_ and Game *G*_*C*_. We will create a PPT algorithm B which breaks Assumption 1 with non-negligible advantage. B is given *g* ∈ *G*_*p*_1__ and *T*. B chooses *a*, *b*, *c*, *d*, *a*_0_, *b*_0_, *c*_0_, *d*_0_, *α* randomly from **Z**_*p*_ and set $u=g^{a},h=g^{b},v=g^{c},w=g^{d},u_{0}=g^{a_{0}},h_{0}=g^{b_{0}},v_{0}=g^{c_{0}},w_{0}=g^{d_{0}}$. It gives the public parameters

$$\begin{array}{c} PP=(g,u,h,v,w,u_{0},h_{0},v_{0},w_{0},\Omega =e{\left(g,g\right)}^{\alpha }) \end{array}$$ (75)

to A. Since B knows the master secret key *α*, it can respond to A’s key requests by calling the key generation update and derive algorithm and giving A the resulting keys.

At some point, A provides two messages *M*_0_, *M*_1_ and requests the challenge ciphertext for some identity vector, denoted by $\left({I_{1}}^{*},\ldots ,{I_{l}}^{*}\right)$ at the time T*. B forms the ciphertext as follows. It chooses *t*_0_, *t*_1_, …*t*_*l*_ randomly from **Z**_*p*_ and *β* randomly from {0, 1} and sets:

$$\begin{array}{c} CT=(M_{\beta }e{\left(g,T\right)}^{\alpha },T,T^{d_{0}}v_{0}^{t_{0}},{\left(u_{0}^{T^{*}}h_{0}\right)}^{t_{0}},g^{t_{0}},{\left\{T^{d}v^{t_{i}},{\left(u^{I_{i}^{*}}h\right)}^{t_{i}},g^{t_{i}}\right\}}_{i=1}^{l}) \end{array}$$ (76)

This implicitly sets *g*^*s*^ equal to the *G*_*p*_1__ part of *T*. If *T* ∈ *G*_*p*_1__, then this is a well-distributed normal ciphertext, and B has properly simulated Game *G*_*Real*_. If *T* ∈ *G*_*p*_1_*p*_2__, then this is a well-distributed semi-functional ciphertext (since the values of *d* modulo *p*_2_ and *d*_0_ modulo *p*_2_ are uncorrelated from their values modulo *p*_1_ by the Chinese Remainder Theorem). Hence, B has properly simulated Game *G*_*C*_ in this case. Thus, B can use the output of A to achieve a non-negligible advantage against Assumption 1.

### 4.8 Indistinguishability of *G*_*SF*_ and *G*_*Final*_

**Lemma 37** *If the Assumption 2 holds*, *then no polynomial-time adversary can distinguish G*_*SF*_ *and G*_*Final*_.

***Proof*** We suppose there exists a PPT attacker A who achieves a non-negligible advantage in Game RHIBE_*SF*_. We will create a PPT algorithm B which has a non-negligible advantage against Assumption 2.

B receives *g*, *g*_2_, *g*_3_, *g*^*α*^ *X*_2_, *g*^*s*^ *Y*_2_, *T*. It chooses *a*, *b*, *c*, *d*, *a*_0_, *b*_0_, *c*_0_, *d*_0_ randomly from **Z**_*p*_ and sets $u=g^{a},h=g^{b},v=g^{c},w=g^{d},u_{0}=g^{a_{0}},h_{0}=g^{b_{0}},v_{0}=g^{c_{0}},w_{0}=g^{d_{0}}$. It gives the public parameters

$$\begin{array}{c} PP=(g,u,h,v,w,u_{0},h_{0},v_{0},w_{0},\Omega =e\left(g,g^{\alpha }X_{2}\right)) \end{array}$$ (77)

to A. We note that B does not know the master secret key *α*. For a secret key query for (*I*_1_, ⋯, *I*_*k*_), B will create a semi-functional secret key as follows. It chooses *f*_1_ randomly and *r*_1,*θ*_, ⋯, *r*_*k*,*θ*_, *b*_1,*θ*_, ⋯, *b*_*k*,*θ*_ ∈ **Z**_*p*_ randomly for each node *θ* ∈ *Path*(*ID*_*k*_). The semi-functional secret key *SK*_*ID*|_*k*__ is formed as ({*θ*, *PSK*_*θ*_}_*θ*∈*path*_), in which $PSK_{\theta }=({\left\{{\tilde{K}}_{i,0},{\tilde{K}}_{i,1},{\tilde{K}}_{i,2},{\tilde{K}}_{i,3}\right\}}_{i=1}^{k})$ and we have $\left({\tilde{K}}_{i,0},{\tilde{K}}_{i,1},{\tilde{K}}_{i,2},{\tilde{K}}_{i,3}\right)$ as

$$\{\begin{array}{cc} \left(g^{\lambda_{i}}w^{b_{i,\theta }},g^{b_{i,\theta }},g^{r_{i,\theta }},{\left(u^{I_{i}}h\right)}^{r_{i,\theta }}v^{b_{i,\theta }}\right), & 0\leq i\leq k-1\\ \left(g^{\gamma_{\theta }-\prod_{i=1}^{k-1}}\lambda_{i}w^{b_{j,\theta }}{\left(g_{2}g_{3}\right)}^{f_{1}\left(d+1\right)},g^{b_{i,\theta }}{\left(g_{2}g_{3}\right)}^{f_{1}},g^{r_{i,\theta }},{\left(u^{I_{i}}h\right)}^{r_{i,\theta }}v^{b_{i,\theta }}{\left(g_{2}g_{3}\right)}^{f_{1}c}\right), & i=k \end{array}$$ (78)

This is a well-distributed semi-functional secret key with *ψ*_1,*θ*_ = *d* + 1, *σ*_1,*θ*_ = *c*(*mod* *p*_2_*p*_3_) and *y*_1_ = *f*_1_(*mod* *p*_2_*p*_3_). Notice that *y*_1_ is freshly random modulo *p*_2_ and *p*_3_ for each key, while *σ*_2,*θ*_, *ψ*_2,*θ*_ are the same for all update keys.

For an update key query for (*I*_1_, ⋯, *I*_*j*−1_) and *T*, B generates a semi-functional update key as follows. It chooses $r_{1,\theta },\ldots ,r_{j,\theta },b_{1,\theta },\ldots ,b_{j-1,\theta },b_{0,\theta }^{\prime }\in Z_{p}$ randomly for each node *θ* ∈ *KUNode*(*BT*_*ID*_*j*−1__) and *f*_2_ randomly. And it will implicitly set $b_{0,\theta }=\left(b_{0,\theta }^{\prime }+\alpha \right)$ *mod p*_1_. The semi-functional update key is formed as *UK*_*ID*|_*j*−1_,*T*_ = ({*θ*, *TUK*_*θ*_}_*θ*∈*KUNode*_) and $TUK_{\theta }=\left({\left\{U_{i,0},U_{i,1},U_{i,2},U_{i,3}\right\}}_{i=0}^{j-1}\right)$:

$$\begin{array}{c} \begin{array}{c} U_{0,0}=g^{-\gamma_{\theta }-\prod_{i=1}^{j-1}\lambda_{i}}{\left(g^{\alpha }X_{2}\right)}^{d_{0}+1}w_{0}^{b_{0,\theta }^{\prime }}{\left(g_{2}g_{3}\right)}^{f_{2}\left(d_{0}+1\right)},U_{0,1}=\left(g^{\alpha }X_{2}\right)g^{b_{0,\theta }^{\prime }}{\left(g_{2}g_{3}\right)}^{f_{2}},\\ U_{0,2}=g^{r_{0,\theta }},U_{0,3}={\left(g^{\alpha }X_{2}\right)}^{c_{0}}{\left(u_{0}^{T}h_{0}\right)}^{r_{0,\theta }}v_{0}^{b_{0,\theta }^{\prime }}{\left(g_{2}g_{3}\right)}^{f_{2}c_{0}}\\ U_{i,0}=g^{\lambda_{i}}w^{b_{i,\theta }},U_{i,1}=g^{b_{i,\theta }},U_{i,2}=g^{r_{i,\theta }},U_{i,3}={\left(u^{I_{i}}h\right)}^{r_{i,\theta }}v^{b_{i,\theta }},i\in \left\{1,\cdots ,j-1\right\} \end{array} \end{array}$$ (79)

This is a well-distributed semi-functional update key with *ψ*_2,*θ*_ = *d*_0_ + 1, *σ*_2,*θ*_ = *c*_0_(*mod* *p*_2_*p*_3_) and *y*_2_ = *f*_2_(*mod* *p*_3_), *y*_2_ = (*f*_2_ + *log*_*g*_2__ *X*_2_)(*mod* *p*_2_), then $X_{2}{\left(g_{2}g_{3}\right)}^{f_{2}}=X_{2}{\left(g_{2}\right)}^{f_{2}}\cdot {\left(g_{3}\right)}^{f_{2}}={\left(g_{2}g_{3}\right)}^{y_{2}}$. Notice that *y*_2_ is freshly random modulo *p*_2_ and *p*_3_ for each update key, while *σ*_2,*θ*_, *ψ*_2,*θ*_ are the same for all update keys.

In response to a decryption key query for (*I*_1_, ⋯, *I*_*j*_) and *T*. B generates the semi-functional secret key and the semi-functional update key at first, and derives an semi-functional decryption key which is formed as

$$\begin{array}{c} \begin{array}{c} D_{0,0}=g^{-\prod_{i=1}^{j}\lambda_{i}}{\left(g^{\alpha }X_{2}\right)}^{d_{0}+1}w_{0}^{b_{0}^{\prime }}{\left(g_{2}g_{3}\right)}^{f_{2}\left(d_{0}+1\right)},D_{0,1}=\left(g^{\alpha }X_{2}\right)g^{b_{0}^{\prime }}{\left(g_{2}g_{3}\right)}^{f_{2}},\\ D_{0,2}=g^{r_{0}},D_{0,3}={\left(g^{\alpha }X_{2}\right)}^{c_{0}}{\left(u_{0}^{T}h_{0}\right)}^{r_{0}}v_{0}^{b_{0}^{\prime }}{\left(g_{2}g_{3}\right)}^{f_{2}c_{0}}\\ D_{i,0}=g^{\lambda_{i}}w^{b_{i}},D_{i,1}=g^{b_{i}},D_{i,2}=g^{r_{i}},D_{i,3}={\left(u^{I_{i}}h\right)}^{r_{i}}v^{b_{i}},i\in \left\{1,\cdots ,j-1\right\}\\ D_{j,0}=g^{\lambda_{j}}w^{b_{j}}{\left(g_{2}g_{3}\right)}^{f_{1}\left(d+1\right)},D_{j,1}=g^{b_{j}}{\left(g_{2}g_{3}\right)}^{f_{1}},\\ D_{j,2}=g^{r_{j}},D_{j,3}={\left(u^{I_{j}}h\right)}^{r_{j}}v^{b_{j}}{\left(g_{2}g_{3}\right)}^{f_{1}c} \end{array} \end{array}$$ (80)

This is a well-distributed semi-functional decryption key.

At some point, A provides B with two messages *M*_0_, *M*_1_, a challenge identity vector $\left(I_{1}^{*},\cdots ,I_{l}^{*}\right)$ and a challenge time T*. B creates the challenge ciphertext as follows. It chooses $t_{1},\cdots ,t_{l},\delta_{1}^{\prime },\delta_{2}^{\prime }$ randomly from *Z*_*n*_ and *β* randomly from {0, 1} and sets:

$$\begin{array}{c} C=M_{\beta }T,C_{0}=g^{s}Y_{2},\\ C_{0,1}={\left(g^{s}Y_{2}\right)}^{d_{0}}v_{0}^{t_{0}}g_{2}^{\delta_{2}^{\prime }},C_{0,2}={\left(u_{0}^{T^{*}}h_{0}\right)}^{t_{0}},C_{0,3}=g^{t_{0}}\\ C_{i,1}={\left(g^{s}Y_{2}\right)}^{d}v^{t_{i}}g_{2}^{\delta_{1}^{\prime }},C_{i,2}={\left(u^{I_{j}^{*}}h\right)}^{t_{i}},C_{i,3}=g^{t_{i}},i\in \left\{1,\cdots ,l\right\} \end{array}$$ (81)

If *T* = *e*(*g*, *g*)^*αs*^, this is a well-distributed semi-functional encryption of *M*_*β*_ with $\gamma =log_{g_{2}}Y_{2},\delta_{1}=d\cdot log_{g_{2}}Y_{2}+\delta_{1}^{\prime },\delta_{2}=d_{0}\cdot log_{g_{2}}Y_{2}+\delta_{2}^{\prime }$. Notice that $\delta_{1}^{\prime }$ and $\delta_{2}^{\prime }$ randomize these so that there is no correlation with *d* or *d*_0_ modulo *p*_2_. Hence this is uncorrelated from the exponents modulo *p*_2_ of the semi-functional keys. In this case, B has properly simulated Game RHIBE_*SF*_.

If *T* is a random element of *G*_*T*_, then this is a semi-functional encryption of a random message, and hence the ciphertext contains no information about *β*. In this case, the advantage of A must be zero. Since we have assumed the advantage of A is non-negligible in Game RHIBE_*SF*_, B can use the output of A to obtain a non-negligible advantage against Assumption 2.

This completes the proof of Theorem 1.

## 5 Conclusion

In this paper, we propose a RHIBE scheme by combining the unbounded LW-(H)IBE and the CS method in a modular way in composite bilinear groups. Moreover, our construction has the advantages of decryption key exposure resistance and short system public parameters. Since neither the naive dual system encryption for bounded RHIBEs nor the naive nested dual system encryption for unbounded HIBEs work in our unbounded RHIBE, we carefully re-design the hybrid games to show the information theoretic arguments successfully in the dual system encryption framework. Our RHIBE is the first unbounded RHIBE scheme that achieves the adaptive security.

## A Defination of the ephemeral semi-functional ciphertexts and keys

In the defination of the first type of ephemeral semi-functional ciphertext, we add *G*_*p*_2__ term on every element of all ciphertext-element-groups. We define a sequence of type-2 ephemeral semi-functional ciphertexts with the index 0 ≤ *k* ≤ *l*, every element of the first *k* − 1 ciphertext-element-groups is in *G*_*p*_1_*p*_2__, and only the first elements of the rest of ciphertext-element-groups are added by *G*_*p*_2__ terms. In the defination of the third type of ephemeral semi-functional ciphertext, every element of the first *i* − 1 ciphertext-element-groups is in *G*_*p*_1_*p*_2__; for the *i*^*st*^ ciphertext-element-group, the first element is in *G*_*p*_1_*p*_2_*p*_3__, its rest elements are in *G*_*p*_1_*p*_3__; and for the rest ciphertext-element-groups, we add *G*_*p*_2__ terms on the first elements of them. In the defination of the fourth type of ephemeral semi-functional ciphertext, every elements of the first *i* − 1 ciphertext-element-groups are in *G*_*p*_1_*p*_2__, every elements of the *i*^*st*^ ciphertext-element-group are in *G*_*p*_1_*p*_2_*p*_3__, and for the rest ciphertext-element-groups, we add *G*_*p*_2__ terms on the first elements of them. In the defination of the fifth type of ephemeral semi-functional ciphertext, every element of all ciphertext-element-groups is in *G*_*p*_1_*p*_2_*p*_3__.

**EncryptESF-1** $\left(ID{|}_{j},T,M,PP,\sigma_{1},\sigma_{2}\right)\to {\tilde{CT}}_{E-1}$ Let the normal ciphertext be $CT_{{ID|}_{l}}=\left(C,C_{0},{\left\{C_{i,1},C_{i,2},C_{i,3}\right\}}_{i=0}^{l}\right)$. It chooses *γ*, *δ*_1_, *δ*_2_, *a*′, *b*′, and random *t*_0_, …, *t*_*j*_ ∈ *Z*_*n*_ and forms the **ESF-1-CT** ${\tilde{CT}}_{E-1}$ as

$$\begin{array}{c} \left(C,C_{0}\cdot g_{2}^{\gamma },C_{0,1}\cdot g_{2}^{\delta_{2}+\sigma_{2}t_{0}},C_{0,2}g_{2}^{\left(a^{\prime }T+b^{\prime }\right)t_{0}},C_{0,3}g_{2}^{t_{0}},{\left\{C_{i,1}g_{2}^{\delta_{1}+\sigma_{1}t_{i}},C_{i,2}g_{2}^{\left(a^{\prime }I_{i}+b^{\prime }\right)t_{i}},C_{i,3}g_{2}^{t_{i}}\right\}}_{i=1}^{j}\right) \end{array}$$

**EncryptESF-2^k^** $\left(ID{|}_{j},T,M,PP,\sigma_{1},\sigma_{2},k\right)\to {\tilde{CT}}_{E-2^{k}}$ It chooses *γ*, *δ*_1_, *δ*_2_, *a*′, *b*′, and random *t*_0_, …, *t*_*k*_ ∈ *Z*_*n*_. It forms the first two elements and the first *k* element-groups of ESF-2^k^-CT as same as of ESF-1-CT, and the rest element-groups of ESF-2^k^-CT as same as of SF-CT.

**EncryptESF-3^k^** $\left(ID{|}_{j},T,M,PP,\sigma_{1},\sigma_{2},k\right)\to {\tilde{CT}}_{E-3^{k}}$ It chooses *γ*, *δ*_1_, *δ*_2_, *a*′, *b*′, and random *t*_0_, …, *t*_*k*_ ∈ *Z*_*n*_, random *X*_3_, *Y*_3_ ∈ *G*_*p*_3__. It forms the first two elements and the first *k* − 1 element-groups of ESF-3^k^-CT as same as of ESF-1-CT, and the *k*^*st*^ element-group of ESF-3^k^-CT as

$$\begin{array}{c} \left(C_{i,1}\cdot g_{2}^{\delta_{1}}X_{3}^{\sigma_{1}},C_{i,2}Y_{3},C_{i,3}X_{3}\right) \end{array}$$

and the rest element-groups of ESF-3^k^-CT as same as of SF-CT.

**EncryptESF-4^k^** $\left(ID{|}_{j},T,M,PP,\sigma_{1},\sigma_{2},k\right)\to {\tilde{CT}}_{E-3^{k}}$ It chooses *γ*, *δ*_1_, *δ*_2_, *a*′, *b*′, and random *t*_0_, …, *t*_*k*_ ∈ *Z*_*n*_, random *X*_3_, *Y*_3_ ∈ *G*_*p*_3__. It forms the first two elements and the first *k* − 1 element-groups of ESF-4^k^-CT as same as of ESF-1-CT, and the *k*^*st*^ element-group of ESF-4^k^-CT as

$$\begin{array}{c} \left(C_{i,1}\cdot g_{2}^{\delta_{1}+\sigma_{1}t_{k}}X_{3}^{\sigma_{1}},C_{i,2}g_{2}^{\left(a^{\prime }I_{k}+b^{\prime }\right)t_{k}}Y_{3},C_{i,3}g_{2}^{t_{k}}X_{3}\right) \end{array}$$

and the rest element-groups of ESF-4^k^-CT as same as of SF-CT.

**EncryptESF-5** $\left(ID{|}_{j},T,M,PP,\sigma_{1},\sigma_{2}\right)\to {\tilde{CT}}_{E-5}$ Let the normal ciphertext be $CT_{{ID|}_{l}}=\left(C,C_{0},{\left\{C_{i,1},C_{i,2},C_{i,3}\right\}}_{i=0}^{l}\right)$. It chooses *γ*, *δ*_1_, *δ*_2_, *a*′, *b*′, *g*_3_ ∈ *G*_*p*_3__, and random $t_{0}\ldots t_{j},t_{0}^{\prime }\ldots t_{j}^{\prime },t_{0}^{\prime \prime }\ldots t_{j}^{\prime \prime },t_{0}^{\prime \prime \prime }\ldots t_{j}^{\prime \prime \prime }\in Z_{n}$. It forms the first two elements of ${\tilde{CT}}_{E-5}$ as $C,C_{0}\cdot g_{2}^{\gamma }$, and forms the element-groups of ESF-5-CT as

$$\begin{array}{c} \begin{array}{c} C_{i,1}\cdot g_{2}^{\delta_{1}+\sigma_{1}t_{k}}g_{3}^{t_{i}^{\prime }},C_{i,2}g_{2}^{\left(a^{\prime }I_{k}+b^{\prime }\right)t_{i}}g_{3}^{t_{i}^{\prime \prime }},C_{i,3}g_{2}^{t_{i}}g_{3}^{t_{i}^{\prime \prime \prime }},\\ {\left\{C_{i,1}\cdot g_{2}^{\delta_{1}+\sigma_{1}t_{k}}g_{3}^{t_{i}^{\prime }},C_{i,2}g_{2}^{\left(a^{\prime }I_{k}+b^{\prime }\right)t_{i}}g_{3}^{t_{i}^{\prime \prime }},C_{i,3}g_{2}^{t_{i}}g_{3}^{t_{i}^{\prime \prime \prime }}\right\}}_{i=1}^{j} \end{array} \end{array}$$

In the defination of the first type of ephemeral semi-functional secret key, we add *G*_*p*_3__ term on the last 2 elements of the last element-group. In the defination of the second type of ephemeral semi-functional secret key, we add *G*_*p*_2_*p*_3__ term on the last 2 elements of the last element-group. In the defination of the third type of ephemeral semi-functional secret key, we add *G*_*p*_3__ term on the first 2 elements of the last element-group and add *G*_*p*_2_*p*_3__ term on the last 2 elements of the last element-group. In the defination of the fourth type of ephemeral semi-functional secret key, every element of the last element-group is in *G*_*p*_1_*p*_2_*p*_3__. In the defination of the fifth type of ephemeral semi-functional secret key, the first 2 elements and the last element of the last element-group is in *G*_*p*_1_*p*_2_*p*_3__, and the third element of the last element-group is in *G*_*p*_1_*p*_3__.

**SKeyESF-1** $\left({ID|}_{j},ST_{{ID|}_{j-1}},PP,\theta \right)\to {\tilde{PSK}}_{E-1}$ Let the correlative component key to the node *θ* ∈ *Path*(*ID*_*j*_) in the *BT*_*ID*|_*j*−1__ be $PSK_{{ID|}_{j},\theta }=\left(\{{K_{i,0},K_{i,1},K_{i,2},K_{i,3}\}}_{i=1}^{j}\right)$. It chooses random values *X*_3_, *Y*_3_ ∈ *G*_*p*_3__ and forms the component ESF-1-SK ${\tilde{PSK}}_{E-1}$ by changing the last element-group as

$$\begin{array}{c} \left(K_{j,0},K_{j,1},K_{j,2}Y_{3},K_{j,3}X_{3}\right) \end{array}$$

**SKeyESF-2** $\left({ID|}_{j},ST_{{ID|}_{j-1}},PP,\theta \right)\to {\tilde{PSK}}_{E-2}$ Let the correlative component key to the node *θ* ∈ *Path*(*ID*_*j*_) in the *BT*_*ID*|_*j*−1__ be $PSK_{{ID|}_{j},\theta }=\left(\{{K_{i,0},K_{i,1},K_{i,2},K_{i,3}\}}_{i=1}^{j}\right)$. It chooses random values *X*_2_, *Y*_2_ ∈ *G*_*p*_2__, *X*_3_, *Y*_3_ ∈ *G*_*p*_3__ and forms the component ESF-2-SK ${\tilde{PSK}}_{E-2}$ by changing the last element-group as

$$\begin{array}{c} \left(K_{j,0},K_{j,1},K_{j,2}Y_{2}Y_{3},K_{j,3}X_{2}X_{3}\right) \end{array}$$

**SKeyESF-3** $\left({ID|}_{j},ST_{{ID|}_{j-1}},PP,\theta \right)\to {\tilde{PSK}}_{E-3}$ It chooses chooses *y*′, *r* ∈ *Z*_*p*_ randomly, *X*_2_, *Y*_2_ ∈ *G*_*p*_2__ randomly, and *X*_3_, *Y*_3_ ∈ *G*_*p*_3__ randomly and forms the component seceret key ESF-3-SK ${\tilde{PSK}}_{E-3}$ by constructing *κ*(*I*_*j*_, *y*′, *r*) in the last element-group as

$$\begin{array}{c} \left(w^{y^{\prime }}g_{3}^{y^{\prime }\psi },g^{y^{\prime }}g_{3}^{y^{\prime }},g^{r}Y_{2}Y_{3},v^{y^{\prime }}{\left(u^{I_{j}}h\right)}^{r}X_{2}X_{3}\right) \end{array}$$

And the contruction of the other element-groups follows the construction of *SK*_*HIBE*,*S*_*θ*__ in RHIBE.GenKey.

**SKeyESF-4** $\left({ID|}_{j},ST_{{ID|}_{j-1}},PP,\theta \right)\to {\tilde{PSK}}_{E-4}$ It chooses chooses *y*′, *r* ∈ *Z*_*p*_ randomly, *X*_2_, *Y*_2_ ∈ *G*_*p*_2__ randomly, and *X*_3_, *Y*_3_ ∈ *G*_*p*_3__ randomly and forms the component ESF-4-SK ${\tilde{PSK}}_{E-4}$ by constructing *κ*(*I*_*j*_, *y*′, *r*) in the last element-group as

$$\begin{array}{c} \left(w^{y^{\prime }}{\left(g_{2}g_{3}\right)}^{y^{\prime }\psi },g^{y^{\prime }}{\left(g_{2}g_{3}\right)}^{y^{\prime }},g^{r}Y_{2}Y_{3},v^{y^{\prime }}{\left(u^{I_{j}}h\right)}^{r}X_{2}X_{3}\right) \end{array}$$

And the contruction of the other element-groups follows the construction of *SK*_*HIBE*,*S*_*θ*__ in RHIBE.GenKey.

**SKeyESF-5** $\left({ID|}_{j},ST_{{ID|}_{j-1}},PP,\theta \right)\to {\tilde{PSK}}_{E-5}$ It chooses chooses *y*′, *r* ∈ *Z*_*p*_ randomly, *X*_2_ ∈ *G*_*p*_2__ randomly, and *X*_3_, *Y*_3_ ∈ *G*_*p*_3__ randomly and forms the component ESF-5-SK ${\tilde{PSK}}_{E-5}$ by by constructing *κ*(*I*_*j*_, *y*′, *r*) in the last element-group as

$$\begin{array}{c} \left(w^{y^{\prime }}{\left(g_{2}g_{3}\right)}^{y^{\prime }\psi },g^{y^{\prime }}{\left(g_{2}g_{3}\right)}^{y^{\prime }},g^{r}Y_{3},v^{y^{\prime }}{\left(u^{I_{j}}h\right)}^{r}X_{2}X_{3}\right) \end{array}$$

And the contruction of the other element-groups follows the construction of *SK*_*HIBE*,*S*_*θ*__ in RHIBE.GenKey.

The constructions from the normal component update key to the (ephemeral) semi-functional component update keys are similar to that of secret keys, expect that we change the first element group of normal component update key to different types.

**UKeyESF-1** $\left(T,ST_{{ID|}_{k-1}},RL_{{ID|}_{k-1},T},PP,\theta \right)\to {\tilde{TUK}}_{E-1}$ Let the correlative component key to the node *θ* ∈ *KUNode*(*RL*_*ID*|_*j*−1_,*T*_) be $TUK_{{ID|}_{j-1},T,\theta }=\left(\{{U_{i,0},U_{i,1},U_{i,2},U_{i,3}\}}_{i=0}^{j-1}\right)$. It chooses random values ${\tilde{r^{\prime }}}_{1},{\tilde{r^{\prime }}}_{2}\in Z_{n}$ and forms the ephemeral semi-functional secret key ${\tilde{TUK}}_{E-1}$ by changing the first element group as It chooses random values *X*_3_, *Y*_3_ ∈ *G*_*p*_3__ and forms the component ESF-1-SK ${\tilde{TUK}}_{E-1}$ by changing the first element-group as

$$\begin{array}{c} \left(U_{j,0},U_{j,1},U_{j,2}Y_{3},U_{j,3}X_{3}\right) \end{array}$$

**UKeyESF-2** $\left(T,ST_{{ID|}_{k-1}},RL_{{ID|}_{k-1},T},PP,\theta \right)\to {\tilde{TUK}}_{E-2}$ Let the correlative component key to the node *θ* ∈ *KUNode*(*RL*_*ID*|_*j*−1_,*T*_) be $TUK_{{ID|}_{j-1},T,\theta }=\left(\{{U_{i,0},U_{i,1},U_{i,2},U_{i,3}\}}_{i=0}^{j-1}\right)$. It chooses random values ${\tilde{r^{\prime }}}_{1},{\tilde{r^{\prime }}}_{2}\in Z_{n}$ and forms the ephemeral semi-functional secret key ${\tilde{TUK}}_{E-2}$ by changing the first element group as

$$\begin{array}{c} \left(U_{0},U_{0,1},U_{0,2}{\left(g_{2}g_{3}\right)}^{{\tilde{r^{\prime }}}_{2}},U_{0,3}{\left(g_{2}g_{3}\right)}^{{\tilde{r^{\prime }}}_{1}}\right) \end{array}$$

**UKeyESF-3** $\left(T,ST_{{ID|}_{k-1}},RL_{{ID|}_{k-1},T},PP,\theta \right)\to {\tilde{TUK}}_{E-3}$ It chooses chooses *y*′, *r* ∈ *Z*_*p*_ randomly, *X*_2_, *Y*_2_ ∈ *G*_*p*_2__ randomly, and *X*_3_, *Y*_3_ ∈ *G*_*p*_3__ randomly and forms the component seceret key ESF-3-UK ${\tilde{TUK}}_{E-3}$ by constructing *κ*_*T*_(*T*, *y*′, *r*) of the first element-group as

$$\begin{array}{c} \left(w_{0}^{y^{\prime }}g_{3}^{y^{\prime }\psi },g^{y^{\prime }}g_{3}^{y^{\prime }},g^{r}Y_{2}Y_{3},v_{0}^{y^{\prime }}{\left(u_{0}^{T}h_{0}\right)}^{r}X_{2}X_{3}\right) \end{array}$$

And the contruction of the other element-groups follows the construction of *RSK*_*HIBE*_ and *SK*_*IBE*,*S*_*θ*__ in RHIBE.UpdateKey.

**UKeyESF-4** $\left(T,ST_{{ID|}_{k-1}},RL_{{ID|}_{k-1},T},PP,\theta \right)\to {\tilde{TUK}}_{E-4}$ It chooses chooses *y*′, *r* ∈ *Z*_*p*_ randomly, *X*_2_, *Y*_2_ ∈ *G*_*p*_2__ randomly, and *X*_3_, *Y*_3_ ∈ *G*_*p*_3__ randomly and forms the component ESF-4-UK ${\tilde{TUK}}_{E-4}$ by constructing *κ*_*T*_(*T*, *y*′, *r*) in the first element-group as

$$\begin{array}{c} \left(w_{0}^{y^{\prime }}{\left(g_{2}g_{3}\right)}^{y^{\prime }\psi },g^{y^{\prime }}{\left(g_{2}g_{3}\right)}^{y^{\prime }},g^{r}Y_{2}Y_{3},v_{0}^{y^{\prime }}{\left(u_{0}^{T}h_{0}\right)}^{r}X_{2}X_{3}\right) \end{array}$$

And the contruction of the other element-groups follows the construction of *RSK*_*HIBE*_ and *SK*_*IBE*,*S*_*θ*__ in RHIBE.UpdateKey.

**UKeyESF-5** $\left(T,ST_{{ID|}_{k-1}},RL_{{ID|}_{k-1},T},PP,\theta \right)\to {\tilde{TUK}}_{E-5}$ It chooses chooses *y*′, *r* ∈ *Z*_*p*_ randomly, *X*_2_ ∈ *G*_*p*_2__ randomly, and *X*_3_, *Y*_3_ ∈ *G*_*p*_3__ randomly and forms the component ESF-5-UK ${\tilde{TUK}}_{E-5}$ by by constructing *κ*_*T*_(*T*, *y*′, *r*) in the first element-group as

$$\begin{array}{c} \left(w_{0}^{y^{\prime }}{\left(g_{2}g_{3}\right)}^{y^{\prime }\psi },g^{y^{\prime }}{\left(g_{2}g_{3}\right)}^{y^{\prime }},g^{r}Y_{3},v_{0}^{y^{\prime }}{\left(u_{0}^{T}h_{0}\right)}^{r}X_{2}X_{3}\right) \end{array}$$

And the contruction of the other element-groups follows the construction of *RSK*_*HIBE*_ and *SK*_*IBE*,*S*_*θ*__ in RHIBE.UpdateKey.

**DKeyESF-i** $\left(ID{|}_{j},T,MSK,RL_{{ID|}_{j-1},T},PP\right)\to {\tilde{DK}}_{E}$ The ephemeral semi-functional decryption key generation algorithm firstly retrieves θ* ∈ *KUNode*(*RL*_*ID*|_*j*−1_,*T*_)⋂ *Path*(*ID*|_*j*_), and gets ${\tilde{TUK}}_{E}$ and ${\tilde{PSK}}_{E}$ which are the correlative subkey to the node θ* from *UKeyESF* − *i*(*T*, *ST*_*ID*|_*j*−1__, θ*) and *SKeyESF* − *i*(*ID*|_*j*_, θ*), and then forms the ephemeral semi-functional decryption key ${\tilde{DK}}_{E}$ as same as **DeriveKeySF**.

## B Proof of lemmas

**Lemma 1** *Under Assumptions 3*, *no PPT attacker can distinguish between O*_0_ *and O*_1/2_ *with non-negligible advantage*. *So no PPT attacker can distinguish between H*_*h*_*c*_−1,2_ *and H*_*h*_*c*_,1_ *with non-negligible advantage*.

***Proof*** We assume B interacts with one of *O*_0_, *O*_1/2_. O receives *g*, *g*_2_, *X*_1_ *X*_3_, *Y*_1_ *Y*_3_, *T*. O will simulate either *O*_0_ or *O*_1/2_ with B, depending on the value of *T* (which is either in *G*_*p*_1__ or *G*_*p*_1_*p*_3__). O picks values *a*, *b*, *c*, *d*, *a*_0_, *b*_0_, *c*_0_, *d*_0_ ∈ *Z*_*N*_ uniformly at random and sets *u* = *g*^*a*^, *h* = *g*^*b*^, *v* = *g*^*c*^, *w* = *g*^*d*^, $u_{0}=g^{a_{0}}$, $h_{0}=g^{b_{0}}$, $v_{0}=g^{c_{0}}$, $w_{0}=g^{d_{0}}$. B initially obtains the group elements

$$\begin{array}{c} \begin{array}{c} g,u,h,v,w,g^{s}g_{2}^{\gamma },{\left(X_{1}X_{3}\right)}^{d}g_{2}^{y_{2}d},\left(X_{1}X_{3}\right)g_{2}^{y_{2}},{\left(X_{1}X_{3}\right)}^{c}g_{2}^{cy_{2}}\\ u_{0},h_{0},v_{0},w_{0},{\left(X_{1}X_{3}\right)}^{zd_{0}}g_{2}^{y_{2}^{\prime }d_{0}},{\left(X_{1}X_{3}\right)}^{z}g_{2}^{y_{2}^{\prime }},{\left(X_{1}X_{3}\right)}^{zc_{0}}g_{2}^{c_{0}y_{2}^{\prime }} \end{array} \end{array}$$ (82)

from its oracle simulator who additionally chooses $s,\gamma ,\delta_{1},\delta_{2},y_{2},y_{2}^{\prime },z\in Z_{N}$ randomly.

We note that these are properly distributed, with *y* modulo *p*_1_ implicitly set to the discrete logarithm of *X*_1_ base *g* modulo *p*_1_, equal to *d* modulo *p*_2_ and *p*_3_, *y*_0_ modulo *p*_1_ implicitly set to the discrete logarithm of *Y*_1_ base *g* modulo *p*_1_, equal to *d*_0_ modulo *p*_2_ and *p*_3_, and *σ* equal to *c* modulo *p*_2_ and *p*_3_. Note that the values of *c* modulo *p*_1_, *p*_2_, *p*_3_ are uncorrelated from each other by the Chinese Remainder Theorem, and *v* = *g*_*c*_ only involves the value of *c* modulo *p*_1_.

B chooses *α* ∈ *Z*_*n*_ randomly, and gives A the following public parameters:

$$\begin{array}{c} PP=(g,u,h,v,w,u_{0},h_{0},v_{0},w_{0},\Omega =e{\left(g,g\right)}^{\alpha }) \end{array}$$ (83)

We note that B knows the master secret key *α*. When A requests a normal update key or a normal decryption key, B can responds by using the usual key generation algorithm, since it knows *α*. And also B can respond the semi-functional keys according to the group elements in Eq 82 that have been offered by the oracle simulator.

When A requests the challenge ciphertext for messages *M*_0_, *M*_1_, identity vector $\left(I_{1}^{*},\cdots ,I_{l}^{*}\right)$ and T*, B makes a ciphertext-type query to the oracle for each $I_{i}^{*}$ and T*. When B makes a ciphertext-type query for some identity I*, O responds by choosing a random *t* ∈ *Z*_*N*_ and returning $\left(w^{s}g_{2}^{\delta_{1}}v^{t},g^{t},{\left(u^{I_{i}^{*}}h\right)}^{t}\right)$ to B as same as Eq 6. When B makes a ciphertext-type query for some time T*, O responds by choosing a random *t*_0_ ∈ *Z*_*N*_ and returning $\left(w_{0}^{s}g_{2}^{\delta_{2}}v_{0}^{t_{0}},g^{t_{0}},{\left(u_{0}^{T^{*}}h_{0}\right)}^{t_{0}}\right)$ as same as Eq 7 to B. Then B creats the semi-functional ciphertexts as

$$\begin{array}{c} CT_{ID^{*}{|}_{l},T^{*}}=\left(g^{s}g_{2}^{\gamma },w_{0}^{s}g_{2}^{\delta_{2}}v_{0}^{t},{\left(u_{0}^{T^{*}}h_{0}\right)}^{t},g^{t},{\left\{w^{s}g_{2}^{\delta_{1}}v^{t_{i}},{\left(u^{I_{i}^{*}}h\right)}^{t_{i}},g^{t_{i}}\right\}}_{i=1}^{l}\right), \end{array}$$

When B creats the HIBE private key with the index pair (*h*, *i*_*c*_) for some identity vector (*I*_1_, ⋯, *I*_*j*_) in the index *h* node, the HIBE private key with an index pair (*h*, *i*_*c*_) is generated as follows:

1. *i*_*c*_ < *h*_*c*_: It randomly chooses *y*_1_, ⋯, *y*_*j*_, $\lambda_{1},\cdots ,\lambda_{j-1},r_{1},\cdots ,r_{j-1},r_{j}^{\prime },z,z^{\prime }\in Z_{n}$ and generates a ESF-2-SK *SK*_*HIBE*,*θ*_*h*__.
  $$\begin{array}{c} \begin{array}{c} K_{i,0}=g^{\lambda_{i}}w^{y_{i}},K_{i,1}=g^{y_{i}},K_{i,2}=g^{r_{i}},K_{i,3}={\left(u^{I_{i}}h\right)}^{r_{i}}v^{y_{i}},i\in \left\{1,\cdots ,j-1\right\}\\ K_{j,0}=g^{\gamma_{\theta }-\sum_{i=1}^{j-1}\lambda_{i}}w^{y_{j}},K_{j,1}=g^{y_{j}},K_{j,2}=v^{y_{j}}{\left(X_{1}X_{3}\right)}^{r_{j}^{\prime }\left(aI_{j}+b\right)}g_{2}^{z},K_{j,3}={\left(X_{1}X_{3}\right)}^{r_{j}^{\prime }}g_{2}^{z^{\prime }} \end{array} \end{array}$$
  It implicitly sets $g^{r_{j}}$ to be $X_{1}^{r_{j}^{\prime }}$ and that is a properly distribution ESF-2-SK.
2. *i*_*c*_ = *h*_*c*_: B chooses random values *y*_1_, ⋯, *y*_*j*−1_, λ_1_, ⋯, λ_*j*−1_, *r*_1_, ⋯, *r*_*j*−1_ ∈ *Z*_*n*_. B forms the challenge key as:
  $$\begin{array}{c} \begin{array}{c} K_{i,0}=g^{\lambda_{i}}w^{y_{i}},K_{i,1}=g^{y_{i}},K_{i,2}=g^{r_{i}},K_{i,3}={\left(u^{I_{i}}h\right)}^{r_{i}}v^{y_{i}},i\in \left\{1,\cdots ,j-1\right\}\\ K_{j,0}=g^{\gamma_{\theta }-\sum_{i=1}^{j-1}\lambda_{i}}T_{0},K_{j,1}=T_{1},K_{j,2}=T_{3},K_{j,3}=T_{2} \end{array} \end{array}$$
  where (*T*_0_, *T*_1_, *T*_2_, *T*_3_) is the challenge HIBE key queried to O who chooses a random *y*_0_ ∈ *Z*_*N*_ and returns (*T*_0_, *T*_1_, *T*_2_, *T*_3_) = (*w*^*y*′^, *g*^*y*′^, *v*^*y*′^ *T*^*aI*+*b*^, *T*) to B.
3. *i*_*c*_ > *h*_*c*_: It simply generates a normal HIBE private key.

In the challenge HIBE key, it implicitly sets *g*^*r*^ to be the *G*_*p*_1__ part of *T*. If *T* ∈ *G*_*p*_1__, then this matches the distribution of *O*_0_ (since there are no *G*_*p*_3__ terms here), and so this will be a properly distributed normal key and B is playing Game *H*_*h*_*c*_−1,2_. If *T* ∈ *G*_*p*_1_*p*_3__, then this matches the distribution of *O*_1/2_ (note that a, b modulo *p*_2_ are uniformly random and do not occur elsewhere- so there are random *G*_*p*_3__ terms attached to the last two group elements) and then B is playing Game *H*_*h*_*c*_,1_.

Hence, if a PPT attacker can distinguish between *H*_*h*_*c*_−1,2_ and *H*_*h*_*c*_,1_ with non-negligible advantage, O can distinguish between *O*_0_ and *O*_1/2_ with non-negligible advantage. It means O can gain a non-negligible advantage against Assumption 3.

Thus, under Assumptions 3, no PPT attacker can distinguish between *O*_0_ and *O*_1/2_ with non-negligible advantage and no PPT attacker can distinguish between *H*_*h*_*c*_−1,2_ and *H*_*h*_*c*_,1_ with non-negligible advantage.

**Lemma 2** *Under Assumptions 4*, *no PPT attacker can distinguish between O*_1/2_ *and O*_1_ *with non-negligible advantage*. *So no PPT attacker can distinguish between H*_*h*_*c*_,1_ *and H*_*h*_*c*_,2_ *with non-negligible advantage*.

***Proof*** We assume B interacts with one of *O*_1/2_, and *O*_1_. O receives *g*, *g*_3_, *X*_1_ *X*_2_, *Y*_2_ *Y*_3_, *T*. O will simulate either *O*_1/2_ or *O*_1_ with B, depending on the value of *T* (which is either in *G* or *G*_*p*_1_*p*_3__). O picks values *a*, *b*, *c*, *d*, *a*_0_, *b*_0_, *c*_0_, *d*_0_ ∈ *Z*_*N*_ uniformly at random and sets *u* = *g*^*a*^, *h* = *g*^*b*^, *v* = *g*^*c*^, *w* = *g*^*d*^, $u_{0}=g^{a_{0}}$, $h_{0}=g^{b_{0}}$, $v_{0}=g^{c_{0}}$, $w_{0}=g^{d_{0}}$. B initially obtains the group elements

$$\begin{array}{c} \begin{array}{c} g,u,h,v,w,X_{1}X_{2},w^{y}{\left(Y_{2}Y_{3}\right)}^{y\psi_{1}},g^{y}{\left(Y_{2}Y_{3}\right)}^{y},v^{y}{\left(Y_{2}Y_{3}\right)}^{y\sigma_{1}},\\ u_{0},h_{0},v_{0},w_{0},w_{0}^{y_{0}}{\left(Y_{2}Y_{3}\right)}^{y_{0}\psi_{2}},g^{y_{0}}{\left(Y_{2}Y_{3}\right)}^{y_{0}},v_{0}^{y_{0}}{\left(Y_{2}Y_{3}\right)}^{y_{0}\sigma_{2}} \end{array} \end{array}$$ (84)

from its oracle simulator, where *y*, *ψ*_1_, *ψ*_2_, *σ*_1_, *σ*_2_ ∈ *Z*_*p*_ are randomly chosen. It chooses *α* ∈ *Z*_*n*_ randomly, and gives A the public parameters in Eq 83.

When A requests the challenge ciphertext for messages *M*_0_, *M*_1_, identity vector $\left(I_{1}^{*},\cdots ,I_{l}^{*}\right)$ and T*, B makes a ciphertext-type query to the oracle for each $I_{i}^{*}$ and T*. In response to each query for $I_{i}^{*}$, O responds $\left({\left(X_{1}X_{2}\right)}^{d}v^{t_{i}},g^{t_{i}},{\left(u^{I_{i}^{*}}h\right)}^{t_{i}}\right)$ to B by choosing a random *t*_*i*_ ∈ *Z*_*N*_. In response to the query for T*, O responds $\left({\left(X_{1}X_{2}\right)}^{d_{0}}v_{0}^{t_{0}},g^{t_{0}},{\left(u_{0}^{T^{*}}h_{0}\right)}^{t_{0}}\right)$ to B by choosing a random *t*_0_ ∈ *Z*_*N*_. Then B creats the semi-functional ciphertexts successfully.

When B creats the HIBE private key with the index pair (*h*, *i*_*c*_) for some identity vector (*I*_1_, ⋯, *I*_*j*_) in the index *h* node, the HIBE private key with an index pair (*h*, *i*_*c*_) is generated as follows:

1. *i*_*c*_ < *h*_*c*_: B chooses random values *y*_1_, ⋯, *y*_*j*_, λ_1_, ⋯, λ_*j*−1_, *r*_1_, ⋯, *r*_*j*_, *z*, *z*′ ∈ *Z*_*n*_ and generates a ESF-2 *PSK*_*HIBE*,*h*_.
  $$\begin{array}{c} \begin{array}{c} K_{i,0}=g^{\lambda_{i}}w^{y_{i}},K_{i,1}=g^{y_{i}},K_{i,2}=g^{r_{i}},K_{i,3}={\left(u^{I_{i}}h\right)}^{r_{i}}v^{y_{i}},i\in \left\{1,\cdots ,j-1\right\}\\ K_{j,0}=g^{\gamma_{\theta }-\sum_{i=1}^{j-1}\lambda_{i}}(w^{y_{j}},K_{j,1}=g^{y_{j}},K_{j,2}=v^{y_{j}}{\left(u^{I_{j}}h\right)}^{r_{j}}{\left(Y_{2}Y_{3}\right)}^{z},K_{j,3}=g^{r_{j}}{\left(Y_{2}Y_{3}\right)}^{z^{\prime }} \end{array} \end{array}$$
2. *i*_*c*_ = *h*_*c*_: B chooses random values *y*_1_, ⋯, *y*_*j*−1_, λ_1_, ⋯, λ_*j*−1_, *r*_1_, ⋯, *r*_*j*−1_ ∈ *Z*_*n*_. B forms the challenge key as:
  $$\begin{array}{c} \begin{array}{c} K_{i,0}=g^{\lambda_{i}}w^{y_{i}},K_{i,1}=g^{y_{i}},K_{i,2}=g^{r_{i}},K_{i,3}={\left(u^{I_{i}}h\right)}^{r_{i}}v^{y_{i}},i\in \left\{1,\cdots ,j-1\right\}\\ K_{j,0}=g^{\gamma_{\theta }-\sum_{i=1}^{j-1}\lambda_{i}}T_{0},K_{j,1}=T_{1},K_{j,2}=T_{3},K_{j,3}=T_{2} \end{array} \end{array}$$
  where (*T*_0_, *T*_1_, *T*_2_, *T*_3_) is the challenge HIBE key queried to O who chooses a random *y*_0_ ∈ *Z*_*N*_ and returns (*T*_0_, *T*_1_, *T*_2_, *T*_3_) = (*w*^*y*′^, *g*^*y*′^, *v*^*y*′^ *T*^*aI*+*b*^, *T*) to B.
3. *i*_*c*_ > *h*_*c*_: It simply generates a normal HIBE private key.

As in the previous lemma, this implicitly sets *g*_*r*_ to be the *G*_*p*_1__ part of *T* in the challenge HIBE key. We note that *a*, *b* modulo *p*_2_,*p*_3_ are uniformly random and do not appear elsewhere. Thus, when *T* ∈ *G*_*p*_1_*p*_3__, these last two terms will have random elements of *G*_*p*_3__ attached (matching the distribution of *O*_1/2_) and then B is playing Game *H*_*h*_*c*_,1_. And when *T* ∈ *G*, these last two terms will have random elements in both *G*_*p*_3__ and *G*_*p*_2__ attached (matching the distribution of *O*_1_) and then B is playing Game *H*_*h*_*c*_,2_.

Hence, if a PPT attacker can distinguish between *H*_*h*_*c*_,1_ and *H*_*h*_*c*_,2_ with non-negligible advantage, O can distinguish between *O*_1/2_ and *O*_1_ with non-negligible advantage. It means O can gain a non-negligible advantage against Assumption 4.

Thus, Under Assumptions 4, no PPT attacker can distinguish between *O*_1/2_ and *O*_1_ with non-negligible advantage. Thus, no PPT attacker can distinguish between *H*_*h*_*c*_,1_ and *H*_*h*_*c*_,2_ with non-negligible advantage.

**Lemma 3** *Under Assumptions 3*, *no PPT attacker can distinguish between* $O_{k-1}^{*}$ *and* $O_{k}^{\prime }$ *with non-negligible advantage*. *So no PPT attacker can distinguish between S*_*k*−1,1_ *and S*_*k*,2_ *with non-negligible advantage*.

***Proof*** We assume B interacts with one of $O_{k-1}^{*},O_{k}^{\prime }$. O receives *g*, *g*_2_, *X*_1_ *X*_3_, *Y*_1_ *Y*_3_, *T*. O will simulate either $O_{k-1}^{*}$ or $O_{k}^{\prime }$ with B, depending on the value of *T* (which is either in *G*_*p*_1__ or *G*_*p*_1_*p*_3__). B initially obtains the group elements in Eq 82

$$\begin{array}{c} \begin{array}{c} g,u,h,v,w,g^{s}g_{2}^{\gamma },{\left(X_{1}X_{3}\right)}^{d}g_{2}^{y_{2}d},\left(X_{1}X_{3}\right)g_{2}^{y_{2}},{\left(X_{1}X_{3}\right)}^{c}g_{2}^{cy_{2}}\\ u_{0},h_{0},v_{0},w_{0},{\left(X_{1}X_{3}\right)}^{zd_{0}}g_{2}^{y_{2}^{\prime }d_{0}},{\left(X_{1}X_{3}\right)}^{z}g_{2}^{y_{2}^{\prime }},{\left(X_{1}X_{3}\right)}^{zc_{0}}g_{2}^{c_{0}y_{2}^{\prime }} \end{array} \end{array}$$

from its oracle simulator. It chooses *α* ∈ *Z*_*n*_ randomly, and gives A the public parameters in Eq 83. B can responds by using the normal update key generation and the normal decryption key derivation algorithm, since it knows *α*.

When A makes a secret key query for the identity *ID*|_*j*_ = (*I*_1_, ⋯, *I*_*j*_), then B makes its challenge HIBE-key-type query for *I*_*j*_, O responds as follows. It chooses *y*′, *r*, *r*_1_, *r*_2_ ∈ *Z*_*N*_ randomly and responds with:

$$\begin{array}{c} \left(w^{y^{\prime }},g^{y^{\prime }},v^{y^{\prime }}{\left(X_{1}X_{3}\right)}^{r(aI_{j}+b)}g_{2}^{r_{1}},{\left(X_{1}X_{3}\right)}^{r}g_{2}^{r_{2}}\right) \end{array}$$

When A requests the challenge ciphertext for messages *M*_0_, *M*_1_, identity vector $\left(I_{1}^{*},\cdots ,I_{l}^{*}\right)$ and T*, B makes a ciphertext-type query to the oracle for each $I_{i}^{*}$ and T*. In response to each query for $I_{i}^{*}$ or T*, O gets random *t*_0_, *t*_1_, …, *t*_*l*_ ∈ *Z*_*p*_, chooses *β* ∈ {0, 1} and creats the ciphertext as

$$\begin{array}{c} \left(C=M_{\beta }e{\left(g^{s}g_{2}^{\gamma },g\right)}^{\alpha },C_{0}=g^{s}g_{2}^{\gamma },{\left\{C_{i,1},C_{i,2},C_{i,3}\right\}}_{i=0}^{l}\right) \end{array}$$

where the ciphertext-element-group (*C*_*i*,1_, *C*_*i*,2_, *C*_*i*,3_) is defined as follows:

1. *i* < *k*: If *i* = 0, O and responds with the ciphertext-element-group $\left(w_{0}^{s}g_{2}^{\delta_{2}}v_{0}^{t_{0}}g_{2}^{c_{0}t_{0}},{\left(u_{0}^{T^{*}}h_{0}\right)}^{t_{0}}g_{2}^{\left(a^{\prime }T^{*}+b^{\prime }\right)t_{0}},g^{t_{0}}g_{2}^{t_{0}}\right)$, else the element group is $\left(w^{s}g_{2}^{\delta_{1}}v^{t_{i}}g_{2}^{ct_{i}},{\left(u^{I_{i}^{*}}h\right)}^{t_{i}}g_{2}^{\left(a^{\prime }I_{i}^{*}+b^{\prime }\right)t_{i}},g^{t_{i}}g_{2}^{t_{i}}\right)$;
2. *i* = *k*: The ciphertext-element-group is (*T*_1_, *T*_3_, *T*_2_) = $\left(w^{s}g_{2}^{\delta_{1}}T^{c},T^{\left(aI_{i}^{*}+b\right)},T\right)$;
3. *i* > *k*: The ciphertext-element-group is $\left(w^{s}g_{2}^{\delta_{1}}v^{t_{i}},{\left(u^{I_{i}^{*}}h\right)}^{t_{i}},g^{t_{i}}\right)$.

We must now argue that the challenge key-type query and the *k*^*th*^ ciphertext-type query responses are properly distributed. If *T* ∈ *G*_*p*_1__, then the response to the *k* ciphertext type query is identically distributed to a response from *O*_1_, and the values *a*, *b* modulo *p*_3_ only appear in the response to the challenge key-type query, hence the *G*_*p*_3__ parts on the last two group elements here appear random in *G*_*p*_3__. This will be a properly distributed EST-2^*k*−1^-CT which means that the responses of O properly simulate the responses of $O_{k-1}^{*}$ and B is playing Game *S*_*k*−1,1_.

If *T* ∈ *G*_*p*_1_*p*_3__, then we must argue that *aI* + *b* and $aI_{k}^{*}+b$ both appear to be uniformly random modulo *p*_3_: this follows from pairwise independence of the function *aI* + *b* modulo *p*_3_, since we have restricted the **Type-1** adversary to choose *I* and $I_{k}^{*}$ so that $I\neq I_{k}^{*}$ modulo *p*_3_. This means that the *G*_*p*_3__ components on the last two group elements of the challenge key-type query response and on the *k* ciphertext-type query response are uniformly random in the attacker’s view. In this case, O has produced a properly distributed EST-3^k^-CT which means that O has properly simulated the responses of $O_{k}^{\prime }$ and B is playing Game *S*_*k*,2_.

Particularly, we need overcome the paradox in the game hopping from Game *S*_*q*_*c*_−1,1_ to Game *S*_*q*_*c*_,2_ since the simulator can derive a decryption key and check whether the ciphertext is normal or semi-functional by being decrypted by the semi-functional derived decryption key from secret keys and update keys. For the game hopping from Game *S*_*q*_*c*_−1,1_ to Game *S*_*q*_*c*_,2_, no matter whether *T* ∈ *G*_*p*_1_*p*_3__ or *T* ∈ *G*_*p*_1__, the cipertext- element-group (*T*_1_, *T*_3_, *T*_2_) can be decrypted by the decryption key derived from the ESF-2-SK and normal update key. So the paradox is overcame successfully. (The other paradox need to overcome is in the game hopping from Game *L*_*q*_*c*_−1,1_ to Game *L*_*q*_*c*_,2_. In Lamma 23, the paradox can be overcame In the same way.)

Hence, if a PPT attacker can distinguish any pair between *S*_*k*−1,1_ and *S*_*k*,2_ with non-negligible advantage, O can distinguish the corresponding pair between $O_{k-1}^{*}$ and $O_{k}^{\prime }$ with non-negligible advantage. It means O can use the output of B to achieve a non-negligible advantage against Assumption 3.

Thus, Under Assumptions 3, no PPT attacker can distinguish between $O_{k-1}^{*}$ and $O_{k}^{\prime }$ with non-negligible advantage. Thus, no PPT attacker can distinguish between *S*_*k*−1,1_ and *S*_*k*,2_ with non-negligible advantage.

**Lemma 4** *Under Assumptions 4*, *no PPT attacker can distinguish between* $O_{k}^{\prime }$ *and* $O_{k}^{\prime \prime }$ *with non-negligible advantage*. *So no PPT attacker can distinguish between S*_*k*,2_ *and S*_*k*,3_ *with non-negligible advantage*.

***Proof*** We assume B interacts with one of $O_{k}^{\prime },O_{k}^{\prime \prime }$. O receives *g*, *g*_3_, *X*_1_ *X*_2_, *Y*_2_ *Y*_3_, *T*. O will simulate either $O_{k}^{\prime }$ or $O_{k}^{\prime \prime }$ with B, depending on the value of *T* (which is either in *G* or *G*_*p*_1_*p*_3__). O picks values *a*, *b*, *c*, *d*, *a*_0_, *b*_0_, *c*_0_, *d*_0_ ∈ *Z*_*N*_ uniformly at random and sets *u* = *g*^*a*^, *h* = *g*^*b*^, *v* = *g*^*c*^, *w* = *g*^*d*^, $u_{0}=g^{a_{0}}$, $h_{0}=g^{b_{0}}$, $v_{0}=g^{c_{0}}$, $w_{0}=g^{d_{0}}$. B initially obtains the group elements

$$\begin{array}{c} \begin{array}{c} g,u,v,w,X_{1}X_{2},w^{y}{\left(Y_{2}Y_{3}\right)}^{\psi },g^{y}\left(Y_{2}Y_{3}\right),v^{y}{\left(Y_{2}Y_{3}\right)}^{c},\\ u_{0},v_{0},w_{0},w_{0}^{y_{0}}{\left(Y_{2}Y_{3}\right)}^{z\psi },g^{y_{0}}{\left(Y_{2}Y_{3}\right)}^{z},v_{0}^{y_{0}}{\left(Y_{2}Y_{3}\right)}^{zc_{0}} \end{array} \end{array}$$ (85)

from its oracle simulator where *z*, *y*_0_, *y*, *ψ* ∈ *Z*_*p*_ are randomly chosen. These are properly distributed, with *g*^*s*^ = *X*_1_ and $g_{2}^{\gamma }=X_{2}$. Note that this sets *σ*_1_ equal to *c* modulo *p*_2_ and *p*_3_ and *σ*_2_ equal to *c*_0_ modulo *p*_2_ and *p*_3_. It chooses *α* ∈ *Z*_*n*_ randomly, and gives A the following public parameters in Eq 83. We note that B knows the master secret key *α*. When A requests a normal update key or a normal decryption key, B can responds by using the usual key generation algorithm, since it knows *α*.

When A makes a secret key query for the identity *ID*|_*j*_ = (*I*_1_, ⋯, *I*_*j*_), then B makes its challenge HIBE-key-type query for *I*_*j*_, O responds as follows. It chooses *y*′, *r*, *r*_1_, *r*_2_ ∈ *Z*_*N*_ randomly and responds with:

$$\begin{array}{c} \left(w^{y^{\prime }},g^{y^{\prime }},v^{y^{\prime }}{\left(u^{I_{j}}h\right)}^{r}{\left(Y_{2}Y_{3}\right)}^{r_{1}},g^{r}{\left(Y_{2}Y_{3}\right)}^{r_{2}}\right) \end{array}$$

This has uniformly random terms in *G*_*p*_2__ and *G*_*p*_3__ on the last two elements, since *r*_1_, *r*_2_ are both uniformly random modulo *p*_2_ and *p*_3_.

When A requests the challenge ciphertext for messages *M*_0_, *M*_1_, identity vector $\left(I_{1}^{*},\cdots ,I_{l}^{*}\right)$ and T*, B makes a ciphertext-type query to the oracle for each $I_{i}^{*}$ and T*. In response to each query for $I_{i}^{*}$ or T*, O gets random $t_{0}^{\prime },t_{1}^{\prime },\ldots ,t_{k}^{\prime },t_{k+1}\ldots ,t_{l}\in Z_{p}$, chooses *β* ∈ {0, 1} and creats the ciphertext as

$$\begin{array}{c} \left(C=M_{\beta }e{\left(X_{1}X_{2},g\right)}^{\alpha },C_{0}=X_{1}X_{2},{\left\{C_{i,1},C_{i,2},C_{i,3}\right\}}_{i=0}^{l}\right) \end{array}$$

where the ciphertext-element-group (*C*_*i*,1_, *C*_*i*,2_, *C*_*i*,3_) is defined as follows:

1. *i* < *k*: If *i* = 0, the ciphertext-element-group is
  $$\begin{array}{c} \left({\left(X_{1}X_{2}\right)}^{d_{0}}{\left(X_{1}X_{2}\right)}^{c_{0}t_{0}^{\prime }},{\left(X_{1}X_{2}\right)}^{t_{0}^{\prime }\left(a_{0}T^{*}+b_{0}\right)},{\left(X_{1}X_{2}\right)}^{t_{0}^{\prime }}\right) \end{array}$$
  else the ciphertext-element-group is
  $$\begin{array}{c} \left({\left(X_{1}X_{2}\right)}^{d}{\left(X_{1}X_{2}\right)}^{ct_{i}^{\prime }},{\left(X_{1}X_{2}\right)}^{t_{i}^{\prime }\left(aI_{i}^{*}+b\right)},{\left(X_{1}X_{2}\right)}^{t_{i}^{\prime }}\right) \end{array}$$
  This sets $X_{2}^{d}=g_{2}^{\delta_{1}}$ and $X_{2}^{d_{0}}=g_{2}^{\delta_{2}}$, which is uniformly random because the value of *d* and *d*_0_ modulo *p*_2_ will not appear elsewhere. It implicitly sets $g^{t_{i}}=X_{1}^{t_{i}^{\prime }}$. This is identically distributed to a response from *O*_2_, with *a*′, *b*′ equal to *a*, *b* modulo *p*_2_, and *σ*_1_ = *c* modulo *p*_2_, *σ*_2_ = *c*_0_ modulo *p*_2_. We note that this is in the only context in which the values of *a*, *b* modulo *p*_2_ appear, so this is equivalent to choosing *a*′, *b*′ independently at random.
2. *i* = *k*: If *k* = 0, the ciphertext-element-group is (*T*_1_, *T*_3_, *T*_2_) = $\left({\left(X_{1}X_{2}\right)}^{d_{0}}T^{c_{0}},T^{\left(a_{0}T^{*}+b_{0}\right)},T\right)$; If *k* > 0, the ciphertext-element-group is (*T*_1_, *T*_3_, *T*_2_) = $\left({\left(X_{1}X_{2}\right)}^{d}T^{c},T^{\left(aI_{i}^{*}+b\right)},T\right)$;
3. *i* > *k*: The ciphertext-element-group is $\left({\left(X_{1}X_{2}\right)}^{d}v^{t_{i}},{\left(u^{I_{i}^{*}}h\right)}^{t_{i}},g^{t_{i}}\right)$.

If *T* ∈ *G*_*p*_1_*p*_3__, then the response for ciphertext-type query *i* is identically distributed to a response from $O_{k}^{\prime }$.

In this case, O has produced a properly distributed EST-3^k^-CT and B is playing Game *S*_*k*,2_.

If *T* ∈ *G*, then this response additionally has terms in *G*_*p*_2__ which are appropriately distributed with *c* = *σ*_1_, *a* = *a*′, *b* = *b*′ modulo *p*_2_ or *c*_0_ = *σ*_2_.*a*_0_ = *a*′, *b*_0_ = *b*′ modulo *p*_2_. Thus, the response is identically distributed to a response from $O_{k}^{\prime \prime }$. In this case, O has produced a properly distributed EST-4^k^-CT and B is playing Game *S*_*k*,3_.

Hence, if a PPT attacker can distinguish any pair between *S*_*k*,2_ and *S*_*k*,3_ with non-negligible advantage, O can distinguish the corresponding pair between $O_{k}^{\prime }$ and $O_{k}^{\prime \prime }$ with non-negligible advantage. It means O can use the output of B to achieve a non-negligible advantage against Assumption 4.

Thus, Under Assumptions 4, no PPT attacker can distinguish between $O_{k}^{\prime }$ and $O_{k}^{\prime \prime }$ with non-negligible advantage. Thus, no PPT attacker can distinguish between *S*_*k*,2_ and *S*_*k*,3_ with non-negligible advantage.

**Lemma 5** *Under Assumptions 3*, *no PPT attacker can distinguish between* $O_{k}^{\prime \prime }$ *and* $O_{k}^{*}$ *with non-negligible advantage*. *So no PPT attacker can distinguish between S*_*k*,3_ *and S*_*k*,1_ *with non-negligible advantage*.

***Proof*** We assume B interacts with one of $O_{k}^{\prime \prime },O_{k}^{*}$. O receives *g*, *g*_2_, *X*_1_ *X*_3_, *T*. O will simulate either $O_{k}^{\prime \prime }$ or $O_{k}^{*}$ with B, depending on the value of *T* (which is either in *G*_*p*_1__ or *G*_*p*_1_*p*_3__). B initially obtains the group elements in Eq 82

$$\begin{array}{c} \begin{array}{c} g,u,h,v,w,g^{s}g_{2}^{\gamma },{\left(X_{1}X_{3}\right)}^{d}g_{2}^{y_{2}d},\left(X_{1}X_{3}\right)g_{2}^{y_{2}},{\left(X_{1}X_{3}\right)}^{c}g_{2}^{cy_{2}}\\ u_{0},h_{0},v_{0},w_{0},{\left(X_{1}X_{3}\right)}^{zd_{0}}g_{2}^{y_{2}^{\prime }d_{0}},{\left(X_{1}X_{3}\right)}^{z}g_{2}^{y_{2}^{\prime }},{\left(X_{1}X_{3}\right)}^{zc_{0}}g_{2}^{c_{0}y_{2}^{\prime }} \end{array} \end{array}$$

from its oracle simulator. It chooses *α* ∈ *Z*_*n*_ randomly, and gives A the public parameters in Eq 83. B can responds by using the normal update key generation and the normal decryption key derivation algorithm, since it knows *α*.

When A makes a secret key query for the identity *ID*|_*j*_ = (*I*_1_, ⋯, *I*_*j*_), then B makes its challenge HIBE-key-type query for *I*_*j*_, O responds as follows. It chooses *y*′, *r*, *r*_1_, *r*_2_ ∈ *Z*_*N*_ randomly and responds with:

$$\begin{array}{c} \left(w^{y^{\prime }},g^{y^{\prime }},v^{y^{\prime }}{\left(X_{1}X_{3}\right)}^{r(aI_{j}+b)}g_{2}^{r_{1}},{\left(X_{1}X_{3}\right)}^{r}g_{2}^{r_{2}}\right) \end{array}$$

We note that the *G*_*p*_2__ parts here are uniformly random.

When A requests the challenge ciphertext for messages *M*_0_, *M*_1_, identity vector $\left(I_{1}^{*},\ldots ,I_{l}^{*}\right)$ and T*, B makes a ciphertext-type query to the oracle for each $I_{i}^{*}$ and T*. In response to each query for $I_{i}^{*}$ or T*, O gets random *t*_0_, *t*_1_, …, *t*_*l*_ ∈ *Z*_*p*_, chooses *β* ∈ {0, 1} and creats the ciphertext as

$$\begin{array}{c} \left(C=M_{\beta }e{\left(g^{s}g_{2}^{\gamma },g\right)}^{\alpha },C_{0}=g^{s}g_{2}^{\gamma },{\left\{C_{i,1},C_{i,2},C_{i,3}\right\}}_{i=0}^{l}\right) \end{array}$$

where the ciphertext-element-group (*C*_*i*,1_, *C*_*i*,2_, *C*_*i*,3_) is defined as follows:

1. *i* < *k*: If *i* = 0, O responds with the ciphertext-element-group $\left(w_{0}^{s}g_{2}^{\delta_{2}}v_{0}^{t_{0}}g_{2}^{c_{0}t_{0}},{\left(u_{0}^{T^{*}}h_{0}\right)}^{t_{0}}g_{2}^{\left(a^{\prime }T^{*}+b^{\prime }\right)t_{0}},g^{t_{0}}g_{2}^{t_{0}}\right)$, else the element group is $\left(w^{s}g_{2}^{\delta_{1}}v^{t_{i}}g_{2}^{ct_{i}},{\left(u^{I_{i}^{*}}h\right)}^{t_{i}}g_{2}^{\left(a^{\prime }I_{i}^{*}+b^{\prime }\right)t_{i}},g^{t_{i}}g_{2}^{t_{i}}\right)$. This is identically distributed to a response from *O*_2_.
2. *i* = *k*: O choses *z* ∈ *Z*_*p*_ randomly and responds with the ciphertext-element-group (*T*_1_, *T*_3_, *T*_2_) = $\left(w^{s}g_{2}^{\delta_{1}}T^{c}\cdot g_{2}^{cz},T^{\left(aI_{i}^{*}+b\right)}g_{2}^{z(a^{\prime }I_{i}^{*}+b^{\prime })},Tg_{2}^{z}\right)$ if *k* > 0. Else if *k* = 0, O responds with $\left(w_{0}^{s}g_{2}^{\delta_{2}}T^{c_{0}}\cdot g_{2}^{c_{0}z},T^{\left(a_{0}T^{*}+b_{0}\right)}g_{2}^{z(a^{\prime }T^{*}+b^{\prime })},Tg_{2}^{z}\right)$. We note that the *G*_*p*_2__ parts here are properly distributed, since *σ*_1_ = *c* modulo *p*_2_ and *σ*_2_ = *c*_0_ modulo *p*_2_.
3. *i* > *k*: The ciphertext-element-group is $\left(w^{s}g_{2}^{\delta_{1}}v^{t_{i}},{\left(u^{I_{i}^{*}}h\right)}^{t_{i}},g^{t_{i}}\right)$. This is identically distributed to a response from *O*_1_.

When *T* ∈ *G*_*p*_1__ the values of *a*, *b* modulo *p*_3_ only appear in the response to the challenge key-type query, which means that the *G*_*p*_3__ terms on the last two group elements there are uniformly random. Also, the response to the *k*^*th*^ ciphertext-type query is distributed exactly like a response from *O*_2_. In this case, O has properly simulated the responses of $O_{k}^{*}$ and this will be a properly distributed EST-2^k^-CT and so B is playing Game *S*_*k*,1_.

When *T* ∈ *G*_*p*_1_*p*_3__, we must argue that the values *aI* + *b* and $aI_{k}^{*}+b$ appear uniformly random modulo *p*_3_: this follows by pairwise independence of *aI* + *b* as a function of *I* modulo *p*_3_, since we have restricted the **Type-1** adversary to choose *I* and $I_{k}^{*}$ so that $I\neq I_{k}^{*}$ modulo *p*_3_ and *a*, *b* modulo *p*_3_ only appear in these two values. Hence, O has produced a properly distributed EST-4^k^-CT and O has properly simulated the response of $O_{k}^{\prime \prime }$ in this case. So B is playing Game *S*_*k*,3_. We have thus shown that O can use the output of B to achieve non-negligible advantage against Assumption 3.

Hence, if a PPT attacker can distinguish any pair between *S*_*k*,3_ and *S*_*k*,1_ with non-negligible advantage, O can distinguish the corresponding pair between $O_{k}^{\prime \prime }$ and $O_{k}^{*}$ with non-negligible advantage. It means O can use the output of B to achieve a non-negligible advantage against Assumption 3.

Thus, Under Assumptions 3, no PPT attacker can distinguish between $O_{k}^{\prime \prime }$ and $O_{k}^{*}$ with non-negligible advantage. Thus, no PPT attacker can distinguish between *S*_*k*,3_ and *S*_*k*,1_ with non-negligible advantage.

**Lemma 6** *Under Assumptions 3*, *no PPT attacker can distinguish between O*_2_ *and O*_5/2_ *with non-negligible advantage*. *So no PPT attacker can distinguish between E*_*h*_*c*_−1,2_ *and E*_*h*_*c*_,1_ *with non-negligible advantage*.

***Proof*** We assume B interacts with one of *O*_2_ and *O*_5/2_. O receives *g*, *g*_2_, *X*_1_ *X*_3_, *Y*_1_ *Y*_3_, *T*. O will simulate either *O*_2_ and *O*_5/2_ with B, depending on the value of *T* (which is either in *G*_*p*_1__ or *G*_*p*_1_*p*_3__). O picks values *a*, *b*, *c*, *d*, *a*_0_, *b*_0_, *c*_0_, *d*_0_ ∈ *Z*_*N*_ uniformly at random and sets *u* = *g*^*a*^, *h* = *g*^*b*^, *v* = *g*^*c*^, *w* = *g*^*d*^, $u_{0}=g^{a_{0}}$, $h_{0}=g^{b_{0}}$, $v_{0}=g^{c_{0}}$, $w_{0}=g^{d_{0}}$. B initially obtains the group elements in Eq 82

$$\begin{array}{c} \begin{array}{c} g,u,h,v,w,g^{s}g_{2}^{\gamma },{\left(X_{1}X_{3}\right)}^{d}g_{2}^{y_{2}d},\left(X_{1}X_{3}\right)g_{2}^{y_{2}},{\left(X_{1}X_{3}\right)}^{c}g_{2}^{cy_{2}}\\ u_{0},h_{0},v_{0},w_{0},{\left(X_{1}X_{3}\right)}^{zd_{0}}g_{2}^{y_{2}^{\prime }d_{0}},{\left(X_{1}X_{3}\right)}^{z}g_{2}^{y_{2}^{\prime }},{\left(X_{1}X_{3}\right)}^{zc_{0}}g_{2}^{c_{0}y_{2}^{\prime }} \end{array} \end{array}$$

from its oracle simulator who additionally chooses $s,\gamma ,\delta_{1},\delta_{2},y_{2},y_{2}^{\prime }\in Z_{N}$ randomly.

B chooses *α* ∈ *Z*_*n*_ randomly, and gives A the public parameters in Eq 83. We note that B knows the master secret key *α*. When A requests a normal decryption key, B can responds by using the usual key generation algorithm, since it knows *α*.

When A requests the challenge ciphertext for messages *M*_0_, *M*_1_, identity vector $\left(I_{1}^{*},\ldots ,I_{l}^{*}\right)$ and T*, B makes a ciphertext-type query to the oracle for each $I_{i}^{*}$ and T*. When B makes a ciphertext-type query for some identity I*, O responds by choosing a random *t* ∈ *Z*_*N*_ and returning $\left(w^{s}g_{2}^{\delta_{1}}v^{t}g_{2}^{\sigma_{1}t},g^{t}g_{2}^{t},{\left(u^{I^{*}}h\right)}^{t}g_{2}^{t(a^{\prime }I^{*}+b^{\prime })}\right)$ to B as same as Eq 10. When B makes a ciphertext-type query for some time T*, O responds by choosing a random *t*_0_ ∈ *Z*_*N*_ and returning $\left(w_{0}^{s}g_{2}^{\delta_{2}}v_{0}^{t_{0}}g_{2}^{\sigma_{2}t_{0}},g^{t_{0}}g_{2}^{t_{0}},{\left(u_{0}^{T^{*}}h_{0}\right)}^{t_{0}}g_{2}^{t_{0}\left(a^{\prime }T^{*}+b^{\prime }\right)}\right)$ as same as Eq 11 to B. Then B creats the ESF-1 ciphertexts successfully.

When A requests the secret key of an identity vector *ID*|_*j*_ = (*I*_1_, ⋯, *I*_*j*_), B creats the ESF-2-SK key by the HIBE-type query response from O and the secret key for *ID*|_*j*_ in some node *θ* is

$$\begin{array}{c} \begin{array}{c} S_{i,0}=g^{\lambda_{i}}w^{y_{i}},S_{i,1}=g^{y_{i}},S_{i,2}=g^{r_{i}},S_{i,3}={\left(u^{I_{i}}h\right)}^{r_{i}}v^{y_{i}},i\in \left\{1,\cdots ,j-1\right\}\\ S_{j,0}=g^{\gamma_{\theta }-\sum_{i=1}^{j-1}\lambda_{i}}w^{y_{j}},S_{j,1}=g^{y_{j}},S_{j,2}=v^{y_{j}}{\left(X_{1}X_{3}\right)}^{r_{j}^{\prime }\left(aI_{j}+b\right)}g_{2}^{z},S_{j,3}={\left(X_{1}X_{3}\right)}^{r_{j}^{\prime }}g_{2}^{z^{\prime }} \end{array} \end{array}$$

where *y*_1_, ⋯, *y*_*j*_, $\lambda_{1},\cdots ,\lambda_{j-1},r_{1},\cdots ,r_{j-1},r_{j}^{\prime },z,z^{\prime }\in Z_{n}$ are randomly chosen.

When B creats the IBE private key with the index pair (*h*, *i*_*c*_) for some time T for the identity vector (*I*_1_, ⋯, *I*_*j*−1_) in the index *h* node, the update key with an index pair (*h*, *i*_*c*_) is generated as follows:

1. *i*_*c*_ < *h*_*c*_: It randomly chooses *y*_0_, ⋯, *y*_*j*−1_, λ_1_, ⋯, λ_*j*−1_, $r_{0}^{\prime },r_{1},\cdots ,r_{j-1}$, *z*, *z*′ ∈ *Z*_*n*_ and generates a ESF-2-UK *TUK*_*ID*|_*l*_,*T*,*θ*_*h*__.
  $$\begin{array}{c} \begin{array}{c} U_{0,0}=g^{\alpha -\gamma_{\theta_{h}}-\sum_{i=1}^{j-1}\lambda_{i}}w_{0}^{y_{j}},U_{0,1}=g^{y_{0}},U_{j,2}=v_{0}^{y_{0}}{\left(X_{1}X_{3}\right)}^{r_{0}^{\prime }\left(a_{0}T+b_{0}\right)}g_{2}^{z},\\ U_{0,3}={\left(X_{1}X_{3}\right)}^{r_{j}^{\prime }}g_{2}^{z^{\prime }},\\ U_{i,0}=g^{\lambda_{i}}w^{y_{i}},U_{i,1}=g^{y_{i}},U_{i,2}=g^{r_{i}},U_{i,3}={\left(u^{I_{i}}h\right)}^{r_{i}}v^{y_{i}},i\in \left\{1,\cdots ,j-1\right\} \end{array} \end{array}$$
  It implicitly sets $g^{r_{0}}$ to be $X_{1}^{r_{0}^{\prime }}$ and that is a properly distribution ESF-2-UK.
2. *i*_*c*_ = *h*_*c*_: B chooses random values *y*_1_, ⋯, *y*_*j*−1_, λ_1_, ⋯, λ_*j*−1_, *r*_1_, ⋯, *r*_*j*−1_ ∈ *Z*_*n*_. B forms the challenge key as:
  $$\begin{array}{c} \begin{array}{c} U_{0,0}=g^{\alpha -\gamma_{\theta_{h}}-\sum_{i=1}^{j-1}\lambda_{i}}T_{0},U_{0,1}=T_{1},U_{0,2}=T_{3},U_{0,3}=T_{2}\\ U_{i,0}=g^{\lambda_{i}}w^{y_{i}},U_{i,1}=g^{y_{i}},K_{i,2}=g^{r_{i}},U_{i,3}={\left(u^{I_{i}}h\right)}^{r_{i}}v^{y_{i}},i\in \left\{1,\cdots ,j-1\right\} \end{array} \end{array}$$
  where (*T*_0_, *T*_1_, *T*_2_, *T*_3_) is the challenge IBE key queried to O who chooses a random *y*_0_ ∈ *Z*_*N*_ and returns (*T*_0_, *T*_1_, *T*_2_, *T*_3_) = $\left(w_{0}^{y_{0}},g^{y_{0}},v_{0}^{y_{0}}T^{a_{0}T+b_{0}},T\right)$ to B.
3. *i*_*c*_ > *h*_*c*_: It simply generates a normal IBE private key.

In the challenge IBE key, it implicitly sets *g*^*r*^ to be the *G*_*p*_1__ part of *T*. If *T* ∈ *G*_*p*_1__, then this matches the distribution of *O*_0_ (since there are no *G*_*p*_3__ terms here), and so this will be a properly distributed normal key and B is playing Game *E*_*h*_*c*_−1,2_. If *T* ∈ *G*_*p*_1_*p*_3__, then this matches the distribution of *O*_1/2_ (note that a, b modulo *p*_2_ are uniformly random and do not occur elsewhere- so there are random *G*_*p*_3__ terms attached to the last two group elements) and then B is playing Game *E*_*h*_*c*_,1_.

Hence, if a PPT attacker can distinguish between *E*_*h*_*c*_−1,2_ and *E*_*h*_*c*_,1_ with non-negligible advantage, O can distinguish between *O*_2_ and *O*_5/2_ with non-negligible advantage. It means O can gain a non-negligible advantage against Assumption 3.

Thus, Under Assumptions 3, no PPT attacker can distinguish between *O*_2_ and *O*_5/2_ with non-negligible advantage. Thus, no PPT attacker can distinguish between *E*_*h*_*c*_−1,2_ and *E*_*h*_*c*_,1_ with non-negligible advantage.

**Lemma 7** *Under Assumptions 4*, *no PPT attacker can distinguish between O*_5/2_ *and O*_3_ *with non-negligible advantage*. *So no PPT attacker can distinguish between E*_*h*_*c*_,1_ *and E*_*h*_*c*_,2_ *with non-negligible advantage*.

***Proof*** We assume B interacts with one of *O*_1/2_, and *O*_1_. O receives *g*, *g*_3_, *X*_1_ *X*_2_, *Y*_2_ *Y*_3_, *T*. O will simulate either *O*_1/2_ or *O*_1_ with B, depending on the value of *T* (which is either in *G* or *G*_*p*_1_*p*_3__). O picks values *a*, *b*, *c*, *d*, *a*_0_, *b*_0_, *c*_0_, *d*_0_ ∈ *Z*_*N*_ uniformly at random and sets *u* = *g*^*a*^, *h* = *g*^*b*^, *v* = *g*^*c*^, *w* = *g*^*d*^, $u_{0}=g^{a_{0}}$, $h_{0}=g^{b_{0}}$, $v_{0}=g^{c_{0}}$, $w_{0}=g^{d_{0}}$. B initially obtains the group elements in Eq 84

$$\begin{array}{c} \begin{array}{c} g,u,h,v,w,X_{1}X_{2},w^{y}{\left(Y_{2}Y_{3}\right)}^{y\psi },g^{y}{\left(Y_{2}Y_{3}\right)}^{y},v^{y}{\left(Y_{2}Y_{3}\right)}^{y\sigma },\\ u_{0},h_{0},v_{0},w_{0},w_{0}^{y_{0}}{\left(Y_{2}Y_{3}\right)}^{y_{0}\psi },g^{y_{0}}{\left(Y_{2}Y_{3}\right)}^{y_{0}},v_{0}^{y_{0}}{\left(Y_{2}Y_{3}\right)}^{y_{0}\sigma } \end{array} \end{array}$$

from its oracle simulator, and gives A the public parameters in Eq 83.

When A requests the challenge ciphertext for messages *M*_0_, *M*_1_, identity vector $\left(I_{1}^{*},\cdots ,I_{l}^{*}\right)$ and T*, B makes a ciphertext-type query to the oracle for each $I_{i}^{*}$ and T*. When B makes a ciphertext-type query for some identity I*, O responds by choosing a random *t* ∈ *Z*_*N*_ and returning $\left(w^{s}g_{2}^{\delta_{1}}v^{t}g_{2}^{\sigma_{1}t},g^{t}g_{2}^{t},{\left(u^{I^{*}}h\right)}^{t}g_{2}^{t(a^{\prime }I^{*}+b^{\prime })}\right)$ to B as same as Eq 10. When B makes a ciphertext-type query for some time T*, O responds by choosing a random *t*_0_ ∈ *Z*_*N*_ and returning $\left(w_{0}^{s}g_{2}^{\delta_{2}}v_{0}^{t_{0}}g_{2}^{\sigma_{2}t_{0}},g^{t_{0}}g_{2}^{t_{0}},{\left(u_{0}^{T^{*}}h_{0}\right)}^{t_{0}}g_{2}^{t_{0}\left(a^{\prime }T^{*}+b^{\prime }\right)}\right)$ as same as Eq 11 to B. Then B creats the ESF-1 ciphertexts successfully.

When A requests the secret key of an identity vector *ID*|_*j*_ = (*I*_1_, ⋯, *I*_*j*_), B creats the ESF-2-SK key by the HIBE-type query response from O and the secret key for *ID*|_*j*_ in some node *θ* is

$$\begin{array}{c} \begin{array}{c} S_{i,0}=g^{\lambda_{i}}w^{y_{i}},S_{i,1}=g^{y_{i}},S_{i,2}=g^{r_{i}},S_{i,3}={\left(u^{I_{i}}h\right)}^{r_{i}}v^{y_{i}},i\in \left\{1,\cdots ,j-1\right\}\\ S_{j,0}=g^{\gamma_{\theta }-\sum_{i=1}^{j-1}\lambda_{i}}w^{y_{j}},S_{j,1}=g^{y_{j}},S_{j,2}=v^{y_{j}}{\left(X_{1}X_{2}\right)}^{r_{j}^{\prime }\left(aI_{j}+b\right)}g_{3}^{z},S_{j,3}={\left(X_{1}X_{2}\right)}^{r_{j}^{\prime }}g_{3}^{z^{\prime }} \end{array} \end{array}$$

where *y*_1_, ⋯, *y*_*j*_, $\lambda_{1},\cdots ,\lambda_{j-1},r_{1},\cdots ,r_{j-1},r_{j}^{\prime },z,z^{\prime }\in Z_{n}$ are randomly chosen.

When B creats the IBE private key with the index pair (*h*, *i*_*c*_) for a time *T* in the index *h* node in the binary tree *BT*_*ID*|_*j*_ = (*I*_1_, ⋯, *I*_*j*−1_)_, the update key with an index pair (*h*, *i*_*c*_) is generated as follows:

1. *i*_*c*_ < *h*_*c*_: B chooses random values *y*_1_, ⋯, *y*_*j*−1_, λ_1_, ⋯, λ_*j*−1_, *r*_0_, ⋯, *r*_*j*−1_, *z*, *z*′ ∈ *Z*_*n*_ and generates a ESF-2 *TUK*_*IBE*,*h*_.
  $$\begin{array}{c} \begin{array}{c} U_{0,0}=g^{\gamma_{\theta }-\sum_{i=1}^{j-1}\lambda_{i}}(w_{0}^{y_{0}},U_{0,1}=g^{y_{0}},U_{0,2}=v_{0}^{y_{0}}{\left(u_{0}^{T}h_{0}\right)}^{r_{0}}{\left(Y_{2}Y_{3}\right)}^{z},U_{0,3}=g^{r_{0}}{\left(Y_{2}Y_{3}\right)}^{z^{\prime }}\\ U_{i,0}=g^{\lambda_{i}}w^{y_{i}},U_{i,1}=g^{y_{i}},K_{i,2}=g^{r_{i}},U_{i,3}={\left(u^{I_{i}}h\right)}^{r_{i}}v^{y_{i}},i\in \left\{1,\cdots ,j-1\right\} \end{array} \end{array}$$
2. *i*_*c*_ = *h*_*c*_: B chooses random values *y*_1_, ⋯, *y*_*j*−1_, λ_1_, ⋯, λ_*j*−1_, *r*_1_, ⋯, *r*_*j*−1_ ∈ *Z*_*n*_. B forms the challenge key as:
  $$\begin{array}{c} \begin{array}{c} U_{0,0}=g^{\alpha -\gamma_{\theta_{h}}-\sum_{i=1}^{j-1}\lambda_{i}}T_{0},U_{0,1}=T_{1},U_{0,2}=T_{3},U_{0,3}=T_{2}\\ U_{i,0}=g^{\lambda_{i}}w^{y_{i}},U_{i,1}=g^{y_{i}},U_{i,2}=g^{r_{i}},U_{i,3}={\left(u^{I_{i}}h\right)}^{r_{i}}v^{y_{i}},i\in \left\{1,\cdots ,j-1\right\} \end{array} \end{array}$$
  where (*T*_0_, *T*_1_, *T*_2_, *T*_3_) is the challenge IBE key queried to O who chooses a random *y*_0_ ∈ *Z*_*N*_ and returns (*T*_0_, *T*_1_, *T*_2_, *T*_3_) = $\left(w_{0}^{y_{0}},g^{y_{0}},v^{y_{0}}T^{a_{0}T+b_{0}},T\right)$ to B.
3. *i*_*c*_ > *h*_*c*_: It simply generates a normal HIBE private key.

As in the previous lemma, this implicitly sets *g*_*r*_0__ to be the *G*_*p*_1__ part of *T* in the challenge IBE key. We note that *a*_0_, *b*_0_ modulo *p*_2_, *p*_3_ are uniformly random and do not appear elsewhere. Thus, when *T* ∈ *G*_*p*_1_*p*_3__, these last two terms will have random elements of *G*_*p*_3__ attached (matching the distribution of *O*_5/2_) and then B is playing Game *E*_*h*_*c*_,1_. And when *T* ∈ *G*, these last two terms will have random elements in both *G*_*p*_3__ and *G*_*p*_2__ attached (matching the distribution of *O*_3_) and then B is playing Game *E*_*h*_*c*_,2_.

Hence, if a PPT attacker can distinguish between *E*_*h*_*c*_,1_ and *E*_*h*_*c*_,2_ with non-negligible advantage, O can distinguish between *O*_5/2_ and *O*_3_ with non-negligible advantage. It means O can gain a non-negligible advantage against Assumption 4.

Thus, Under Assumptions 4, no PPT attacker can distinguish between *O*_5/2_ and *O*_3_ with non-negligible advantage. Thus, no PPT attacker can distinguish between *E*_*h*_*c*_,1_ and *E*_*h*_*c*_,2_ with non-negligible advantage.

**Lemma 8** *Under Assumptions 3*, *no PPT attacker can distinguish between O*_3_ *and O*_3.1_ *with non-negligible advantage*. *So no PPT attacker can distinguish between F*_*h*_*c*_−1_ *and F*_*h*_*c*__ *with non-negligible advantage*.

***Proof*** We assume B interacts with one of *O*_3_, *O*_3.1_. O receives *g*, *g*_2_, *X*_1_ *X*_3_, *Y*_1_ *Y*_3_, *T*. O will simulate either *O*_0_ or *O*_1/2_ with B, depending on the value of *T* (which is either in *G*_*p*_1__ or *G*_*p*_1_*p*_3__). O picks values *a*, *b*, *c*, *d*, *a*_0_, *b*_0_, *c*_0_, *d*_0_ ∈ *Z*_*N*_ uniformly at random and sets *u* = *g*^*a*^, *h* = *g*^*b*^, *v* = *g*^*c*^, *w* = *g*^*d*^, $u_{0}=g^{a_{0}}$, $h_{0}=g^{b_{0}}$, $v_{0}=g^{c_{0}}$, $w_{0}=g^{d_{0}}$. B initially obtains the group elements in Eq 82

$$\begin{array}{c} \begin{array}{c} g,u,h,v,w,g^{s}g_{2}^{\gamma },{\left(X_{1}X_{3}\right)}^{d}g_{2}^{y_{2}d},\left(X_{1}X_{3}\right)g_{2}^{y_{2}},{\left(X_{1}X_{3}\right)}^{c}g_{2}^{cy_{2}}\\ u_{0},h_{0},v_{0},w_{0},{\left(X_{1}X_{3}\right)}^{zd_{0}}g_{2}^{y_{2}^{\prime }d_{0}},{\left(X_{1}X_{3}\right)}^{z}g_{2}^{y_{2}^{\prime }},{\left(X_{1}X_{3}\right)}^{zc_{0}}g_{2}^{c_{0}y_{2}^{\prime }} \end{array} \end{array}$$

from its oracle simulator who additionally chooses $s,\gamma ,\delta_{1},\delta_{2},y_{2},y_{2}^{\prime }\in Z_{N}$ randomly. B chooses *α* ∈ *Z*_*n*_ randomly, and gives A the following public parameters in Eq 83. We note that B knows the master secret key *α*.

When A requests the challenge ciphertext for messages *M*_0_, *M*_1_, identity vector $\left(I_{1}^{*},\ldots ,I_{l}^{*}\right)$ and T*, B makes a ciphertext-type query to the oracle for each $I_{i}^{*}$ and T*. When B makes a ciphertext-type query for some identity I*, O responds by choosing a random *t* ∈ *Z*_*N*_ and returning $\left(w^{s}g_{2}^{\delta_{1}}v^{t}g_{2}^{ct},g^{t}g_{2}^{t},{\left(u^{I^{*}}h\right)}^{t}g_{2}^{a^{\prime }I^{*}+b^{\prime }}\right)$ to B. When B makes a ciphertext-type query for some time T*, O responds by choosing a random *t*_0_ ∈ *Z*_*N*_ and returning $\left(w_{0}^{s}g_{2}^{\delta_{2}}v_{0}^{t_{0}}g_{2}^{c_{0}t_{0}},g^{t_{0}}g_{2}^{t_{0}},{\left(u_{0}^{T^{*}}h_{0}\right)}^{t_{0}}g_{2}^{a^{\prime }T^{*}+b^{\prime }}\right)$ to B. Then B creats the ESF-1 ciphertexts successfully.

Upon receiving a challenge IBE-key-type query for *T* ∈ *Z*_*n*_, O chooses *r*_1_, *r*_2_, *r*′, *y*″ ∈ *Z*_*n*_ randomly and returns the group elements

$$\begin{array}{c} \left({\left(g\right)}^{d_{0}y^{\prime \prime }},{\left(g\right)}^{y^{\prime \prime }},{\left(g\right)}^{c_{0}y^{\prime \prime }}{\left(X_{1}X_{3}\right)}^{\left(a_{0}T+b_{0}\right)r^{\prime }}g_{2}^{r_{1}},{\left(X_{1}X_{3}\right)}^{r^{\prime }}g_{2}^{r_{2}}\right) \end{array}$$

to B. And then B creats the ESF-2 update key by using the group elements.

When B creats the HIBE private key with the index pair (*h*, *i*_*c*_) for some identity vector (*I*_1_, ⋯, *I*_*j*_) in the index *h* node, the HIBE private key with an index pair (*h*, *i*_*c*_) is generated as follows:

1. *i*_*c*_ < *h*_*c*_: It randomly chooses $y_{1},\cdots ,y_{j-1},y_{j}^{\prime }$, $\lambda_{1},\cdots ,\lambda_{j-1},r_{1},\cdots ,r_{j-1},r_{j}^{\prime },z,z^{\prime }\in Z_{n}$ and generates a ESF-3-SK *PSK*_*HIBE*,*h*_.
  $$\begin{array}{c} \begin{array}{c} K_{i,0}=g^{\lambda_{i}}w^{y_{i}},K_{i,1}=g^{y_{i}},K_{i,2}=g^{r_{i}},K_{i,3}={\left(u^{I_{i}}h\right)}^{r_{i}}v^{y_{i}},i\in \left\{1,\cdots ,j-1\right\}\\ K_{j,0}=g^{\gamma_{\theta }-\sum_{i=1}^{j-1}\lambda_{i}}{\left(X_{1}X_{3}\right)}^{dy_{j}^{\prime }},K_{j,1}={\left(X_{1}X_{3}\right)}^{y_{j}^{\prime }},\\ K_{j,2}={\left(X_{1}X_{3}\right)}^{cy_{j}^{\prime }}{\left(X_{1}X_{3}\right)}^{r_{j}^{\prime }\left(aI_{j}+b\right)}g_{2}^{z},K_{j,3}={\left(X_{1}X_{3}\right)}^{r_{j}^{\prime }}g_{2}^{z^{\prime }} \end{array} \end{array}$$
  It implicitly sets $g^{y_{j}}$ to be $X_{1}^{y_{j}^{\prime }}$ and $g^{r_{j}}$ to be $X_{1}^{r_{j}^{\prime }}$ and that is a properly distribution ESF-3-SK.
2. *i*_*c*_ = *h*_*c*_: B chooses random values *y*_1_, ⋯, *y*_*j*−1_, λ_1_, ⋯, λ_*j*−1_, *r*_1_, ⋯, *r*_*j*−1_ ∈ *Z*_*n*_. B forms the challenge key as:
  $$\begin{array}{c} \begin{array}{c} K_{i,0}=g^{\lambda_{i}}w^{y_{i}},K_{i,1}=g^{y_{i}},K_{i,2}=g^{r_{i}},K_{i,3}={\left(u^{I_{i}}h\right)}^{r_{i}}v^{y_{i}},i\in \left\{1,\cdots ,j-1\right\}\\ K_{j,0}=g^{\gamma_{\theta }-\sum_{i=1}^{j-1}\lambda_{i}}T_{0},K_{j,1}=T_{1},K_{j,2}=T_{3},K_{j,3}=T_{2} \end{array} \end{array}$$
  where (*T*_0_, *T*_1_, *T*_2_, *T*_3_) is the challenge HIBE key queried to O who chooses a random *r*, *r*_1_, *r*_2_ ∈ *Z*_*N*_ and returns (*T*_0_, *T*_1_, *T*_2_, *T*_3_) = $\left(T^{d},T,T^{c}{\left(X_{1}X_{3}\right)}^{r(aI+b)}g_{2}^{r_{1}},{\left(X_{1}X_{3}\right)}^{r}g_{2}^{r_{2}}\right)$ to B.
3. *i*_*c*_ > *h*_*c*_: It randomly chooses *y*_1_, ⋯, *y*_*j*_, $\lambda_{1},\cdots ,\lambda_{j-1},r_{1},\cdots ,r_{j-1},r_{j}^{\prime },z,z^{\prime }\in Z_{n}$ and generates a ESF-2-SK *PSK*_*HIBE*,*h*_.
  $$\begin{array}{c} \begin{array}{c} K_{i,0}=g^{\lambda_{i}}w^{y_{i}},K_{i,1}=g^{y_{i}},K_{i,2}=g^{r_{i}},K_{i,3}={\left(u^{I_{i}}h\right)}^{r_{i}}v^{y_{i}},i\in \left\{1,\cdots ,j-1\right\}\\ K_{j,0}=g^{\gamma_{\theta }-\sum_{i=1}^{j-1}\lambda_{i}}w^{y_{j}},K_{j,1}=g^{y_{j}},K_{j,2}=v^{y_{j}}{\left(X_{1}X_{3}\right)}^{r_{j}^{\prime }\left(aI_{j}+b\right)}g_{2}^{z},K_{j,3}={\left(X_{1}X_{3}\right)}^{r_{j}^{\prime }}g_{2}^{z^{\prime }} \end{array} \end{array}$$
  It implicitly sets $g^{r_{j}}$ to be $X_{1}^{r_{j}^{\prime }}$ and that is a properly distribution ESF-2-SK.

In the challenge HIBE key, it implicitly sets *g*^*y*′^ to be the *G*_*p*_1__ part of *T*. If *T* ∈ *G*_*p*_1__, then this matches the distribution of *O*_3_ (since there are no *G*_*p*_3__ terms here), and so this will be a properly distributed normal key and B is playing Game *F*_*h*_*c*_−1_. If *T* ∈ *G*_*p*_1_*p*_3__, then this matches the distribution of *O*_3.1_ (note that a, b modulo *p*_2_ are uniformly random and do not occur elsewhere- so there are random *G*_*p*_3__ terms attached to the last two group elements) and then B is playing Game *F*_*h*_*c*__.

Hence, if a PPT attacker can distinguish between *F*_*h*_*c*_−1_ and *F*_*h*_*c*__ with non-negligible advantage, O can distinguish between *O*_3_ and *O*_3.1_ with non-negligible advantage. It means O can gain a non-negligible advantage against Assumption 3.

Thus, Under Assumptions 3, no PPT attacker can distinguish between *O*_3_ and *O*_3.1_ with non-negligible advantage. Thus, no PPT attacker can distinguish between *F*_*h*_*c*_−1_ and *F*_*h*_*c*__ with non-negligible advantage.

**Lemma 9** *Under Assumptions 3*, *no PPT attacker can distinguish between O*_3.1_ *and O*_3.2_ *with non-negligible advantage*. *So no PPT attacker can distinguish between F*1_*h*_*c*_−1_ *and F*1_*h*_*c*__ *with non-negligible advantage*.

***Proof*** The proof of this lemma is almost the same as that of Lemma 8 except the generation of secret keys and update keys.

Upon receiving a challenge HIBE-key-type query for *I* ∈ *Z*_*n*_, O chooses *r*_1_, *r*_2_, *r*′, *y*″ ∈ *Z*_*n*_ randomly and returns the group elements

$$\begin{array}{c} \left({\left(X_{1}X_{3}\right)}^{dy^{\prime \prime }},{\left(X_{1}X_{3}\right)}^{y^{\prime \prime }},{\left(X_{1}X_{3}\right)}^{cy^{\prime \prime }}{\left(X_{1}X_{3}\right)}^{\left(a_{I}+b\right)r^{\prime }}g_{2}^{r_{1}},{\left(X_{1}X_{3}\right)}^{r^{\prime }}g_{2}^{r_{2}}\right) \end{array}$$

to B. And then B creats the ESF-3 update key by using the group elements.

When B creats the IBE private key with the index pair (*h*, *i*_*c*_) for some identity vector (*I*_1_, ⋯, *I*_*j*−1_) and the time *T* in the index *h* node, the IBE private key with an index pair (*h*, *i*_*c*_) is generated as follows:

1. *i*_*c*_ < *h*_*c*_: It randomly chooses $y_{1},\cdots ,y_{j-1},y_{0}^{\prime }$, $\lambda_{1},\cdots ,\lambda_{j-1},r_{1},\cdots ,r_{j-1},r_{0}^{\prime },z,z^{\prime }\in Z_{n}$ and generates a ESF-3-UK *EUK*_*IBE*,*h*_.
  $$\begin{array}{c} \begin{array}{c} U_{0,0}=g^{\alpha -\gamma_{\theta }-\sum_{i=1}^{j-1}\lambda_{i}}{\left(X_{1}X_{3}\right)}^{d_{0}y_{0}^{\prime }},U_{0,1}={\left(X_{1}X_{3}\right)}^{y_{0}^{\prime }},\\ U_{0,2}={\left(X_{1}X_{3}\right)}^{c_{0}y_{0}^{\prime }}{\left(X_{1}X_{3}\right)}^{r_{0}^{\prime }\left(a_{0}T+b_{0}\right)}g_{2}^{z},U_{0,3}={\left(X_{1}X_{3}\right)}^{r_{0}^{\prime }}g_{2}^{z^{\prime }}\\ U_{i,0}=g^{\lambda_{i}}w^{y_{i}},U_{i,1}=g^{y_{i}},U_{i,2}=g^{r_{i}},U_{i,3}={\left(u^{I_{i}}h\right)}^{r_{i}}v^{y_{i}},i\in \left\{1,\cdots ,j-1\right\} \end{array} \end{array}$$
  It implicitly sets $g^{y_{0}}$ to be $X_{1}^{y_{0}^{\prime }}$ and $g^{r_{0}}$ to be $X_{1}^{r_{0}^{\prime }}$ and that is a properly distribution ESF-3-UK.
2. *i*_*c*_ = *h*_*c*_: B chooses random values *y*_1_, ⋯, *y*_*j*−1_, λ_1_, ⋯, λ_*j*−1_, *r*_1_, ⋯, *r*_*j*−1_ ∈ *Z*_*n*_. B forms the challenge key as:
  $$\begin{array}{c} \begin{array}{c} U_{0,0}=g^{\alpha -\gamma_{\theta }-\sum_{i=1}^{j-1}\lambda_{i}}T_{0},\\ U_{0,1}=T_{1},U_{0,2}=T_{3},U_{0,3}=T_{2}\\ U_{i,0}=g^{\lambda_{i}}w^{y_{i}},U_{i,1}=g^{y_{i}},U_{i,2}=g^{r_{i}},U_{i,3}={\left(u^{I_{i}}h\right)}^{r_{i}}v^{y_{i}},i\in \left\{1,\cdots ,j-1\right\} \end{array} \end{array}$$
  where (*T*_0_, *T*_1_, *T*_2_, *T*_3_) is the challenge IBE key queried to O who chooses a random *r*, *r*_1_, *r*_2_ ∈ *Z*_*N*_ and returns (*T*_0_, *T*_1_, *T*_2_, *T*_3_) = $\left(T^{d_{0}},T,T^{c_{0}}{\left(X_{1}X_{3}\right)}^{r(a_{0}T+b_{0})}g_{2}^{r_{1}},{\left(X_{1}X_{3}\right)}^{r}g_{2}^{r_{2}}\right)$ to B.
3. *i*_*c*_ > *h*_*c*_: It randomly chooses *y*_1_, ⋯, *y*_*j*_, $\lambda_{1},\cdots ,\lambda_{j-1},r_{1},\cdots ,r_{j-1},r_{j}^{\prime },z,z^{\prime }\in Z_{n}$ and generates a ESF-2-UK *EUK*_*IBE*,*h*_.
  $$\begin{array}{c} \begin{array}{c} U_{0,0}=g^{\gamma_{\theta }-\sum_{i=1}^{j-1}\lambda_{i}}w_{)}^{y_{0}},U_{0,1}=g^{y_{0}},U_{0,2}=v_{0}^{y_{0}}{\left(X_{1}X_{3}\right)}^{r_{0}^{\prime }\left(a_{0}T+b_{0}\right)}g_{2}^{z},U_{0,3}={\left(X_{1}X_{3}\right)}^{r_{j}^{\prime }}g_{2}^{z^{\prime }}\\ U_{i,0}=g^{\lambda_{i}}w^{y_{i}},U_{i,1}=g^{y_{i}},U_{i,2}=g^{r_{i}},U_{i,3}={\left(u^{I_{i}}h\right)}^{r_{i}}v^{y_{i}},i\in \left\{1,\cdots ,j-1\right\} \end{array} \end{array}$$
  It implicitly sets $g^{r_{0}}$ to be $X_{1}^{r_{0}^{\prime }}$ and that is a properly distribution ESF-2-UK.

In the challenge IBE key, it implicitly sets *g*^*y*′^ to be the *G*_*p*_1__ part of *T*. If *T* ∈ *G*_*p*_1__, then this matches the distribution of *O*_3.1_ (since there are no *G*_*p*_3__ terms here), and so this will be a properly distributed normal key and B is playing Game *F*1_*h*_*c*_−1_. If *T* ∈ *G*_*p*_1_*p*_3__, then this matches the distribution of *O*_3.2_ (note that a, b modulo *p*_2_ are uniformly random and do not occur elsewhere- so there are random *G*_*p*_3__ terms attached to the last two group elements) and then B is playing Game *F*1_*h*_*c*__.

Hence, if a PPT attacker can distinguish between *F*1_*h*_*c*_−1_ and *F*1_*h*_*c*__ with non-negligible advantage, O can distinguish between *O*_3.1_ and *O*_3.2_ with non-negligible advantage. It means O can gain a non-negligible advantage against Assumption 3.

Thus, Under Assumptions 3, no PPT attacker can distinguish between *O*_3.1_ and *O*_3.2_ with non-negligible advantage. Thus, no PPT attacker can distinguish between *F*1_*h*_*c*_−1_ and *F*1_*h*_*c*__ with non-negligible advantage.

**Lemma 10** *Under Assumptions 4*, *no PPT attacker can distinguish between O*_3.2_ *and O*_3.3_ *with non-negligible advantage*. *So no PPT attacker can distinguish between G*_*ESF*′−2_ *and G*_*ESF*′−3_ *with non-negligible advantage*.

***Proof*** We assume B interacts with one of *O*_3.2_, *O*_3.3_. O receives *g*, *g*_2_, *X*_1_ *X*_3_, *Y*_2_ *Y*_3_, *T*. O will simulate either *O*_3.2_ or *O*_3.3_ with B, depending on the value of *T* (which is either in *G* or *G*_*p*_1_*p*_2__). O picks values *a*, *b*, *c*, *d*, *a*_0_, *b*_0_, *c*_0_, *d*_0_ ∈ *Z*_*N*_ uniformly at random and sets *u* = *g*^*a*^, *h* = *g*^*b*^, *v* = *g*^*c*^, *w* = *g*^*d*^, $u_{0}=g^{a_{0}}$, $h_{0}=g^{b_{0}}$, $v_{0}=g^{c_{0}}$, $w_{0}=g^{d_{0}}$. It chooses random values *σ*_1_, *σ*_2_, *y*, *y*′, *t*_3_, *z* ∈ *Z*_*N*_ and then B initially obtains the group elements

$$\begin{array}{c} \begin{array}{c} g,u,h,v,w,T,w^{y}{\left(Y_{2}Y_{3}\right)}^{d},g^{y}\left(Y_{2}Y_{3}\right),v^{y}{\left(Y_{2}Y_{3}\right)}^{\sigma_{1}}\\ u_{0},h_{0},v_{0},w_{0},w_{0}^{y^{\prime }}{\left(Y_{2}Y_{3}\right)}^{zd_{0}},g^{y^{\prime }}{\left(Y_{2}Y_{3}\right)}^{z},v_{0}^{y^{\prime }}{\left(Y_{2}Y_{3}\right)}^{\sigma_{2}} \end{array} \end{array}$$ (86)

We note that this sets *ψ*_1_ = *d* modulo *p*_2_ and *p*_3_. It implicitly sets *g*^*s*^ to be the *G*_*p*_1__ part of *T*. If *T* ∈ *G*_*p*_1_*p*_2__, this is distributed identically to the initial elements provided by *O*_3.2_. If *T* ∈ *G*, this is distributed identically to the initial elements provided by *O*_3.3_.

Upon receiving a challenge IBE-key-type query for *T* ∈ *Z*_*n*_, O chooses *r*_1_, *r*_2_, *r*′, *y*″ ∈ *Z*_*n*_ randomly and returns the group elements

$$\begin{array}{c} \left({\left(g\right)}^{d_{0}y^{\prime \prime }},{\left(g\right)}^{y^{\prime \prime }},{\left(g\right)}^{c_{0}y^{\prime \prime }}{\left(X_{1}X_{3}\right)}^{\left(a_{0}T+b_{0}\right)r^{\prime }}g_{2}^{r_{1}},{\left(X_{1}X_{3}\right)}^{r^{\prime }}g_{2}^{r_{2}}\right) \end{array}$$

to B. And then B creats the ESF-2 update key by using the group elements.

When B creats the HIBE private key with the index pair (*h*, *i*_*c*_) for some identity vector (*I*_1_, ⋯, *I*_*j*_) in the index *h* node, the HIBE private key with an index pair (*h*, *i*_*c*_) is generated as follows:

When A requests the challenge ciphertext for messages *M*_0_, *M*_1_, identity vector $\left(I_{1}^{*},\cdots ,I_{l}^{*}\right)$ and T*, B makes a ciphertext-type query to the oracle for each $I_{i}^{*}$ and T*. When B makes a ciphertext-type query for some identity I*, O responds by choosing a random *t* ∈ *Z*_*N*_ and returning $\left(w^{s}g_{2}^{\delta_{1}}v^{t}g_{2}^{ct},g^{t}g_{2}^{t},{\left(u^{I^{*}}h\right)}^{t}g_{2}^{a^{\prime }I^{*}+b^{\prime }}\right)$ to B. When B makes a ciphertext-type query for some time T*, O responds by choosing a random *t*_0_ ∈ *Z*_*N*_ and returning $\left(w_{0}^{s}g_{2}^{\delta_{2}}v_{0}^{t_{0}}g_{2}^{c_{0}t_{0}},g^{t_{0}}g_{2}^{t_{0}},{\left(u_{0}^{T^{*}}h_{0}\right)}^{t_{0}}g_{2}^{a^{\prime }T^{*}+b^{\prime }}\right)$ to B. Then B creats the ESF-1 ciphertexts successfully.

**Lemma 11** *Under Assumptions 4*, *no PPT attacker can distinguish between O*_3.3_ *and O*_3.4_ *with non-negligible advantage*. *So no PPT attacker can distinguish between G*_*ESF*′−3_ *and G*_*ESF*′−4_ *with non-negligible advantage*.

***Proof*** We assume B interacts with one of *O*_3.3_, *O*_3.4_. O receives *g*, *g*_3_, *X*_1_ *X*_2_, *Y*_2_ *Y*_3_, *T*. O will simulate either *O*_3.3_ or *O*_3.4_ with B, depending on the value of *T* (which is either in *G* or *G*_*p*_1_*p*_3__). O picks values *a*, *b*, *c*, *d*, *a*_0_, *b*_0_, *c*_0_, *d*_0_ ∈ *Z*_*N*_ uniformly at random and sets *u* = *g*^*a*^, *h* = *g*^*b*^, *v* = *g*^*c*^, *w* = *g*^*d*^, $u_{0}=g^{a_{0}}$, $h_{0}=g^{b_{0}}$, $v_{0}=g^{c_{0}}$, $w_{0}=g^{d_{0}}$. B initially obtains the group elements

$$\begin{array}{c} \begin{array}{c} g,u,h,v,w,g^{s}{\left(Y_{2}Y_{3}\right)}^{\gamma },w^{y}{\left(Y_{2}Y_{3}\right)}^{d},g^{y}Y_{2}Y_{3},v^{y}{Y_{2}Y_{3}}^{c}\\ u_{0},h_{0},v_{0},w_{0},w_{0}^{y^{\prime }}{\left(Y_{2}Y_{3}\right)}^{zd_{0}},g^{y^{\prime }}{Y_{2}Y_{3}}^{z},v_{0}^{y^{\prime }}{Y_{2}Y_{3}}^{zc_{0}} \end{array} \end{array}$$ (87)

from its oracle simulator who additionally chooses *s*, *γ*, *y*, *y*′, *z* ∈ *Z*_*N*_ randomly. We note that this is properly distributed and set *ψ*_1_ = *d* modulo *p*_2_ and *p*_3_, *ψ*_2_ = *d*_0_ modulo *p*_2_ and *p*_3_ and *σ*_1_ = *c* modulo *p*_2_ and *p*_3_, *σ*_2_ = *c*_0_ modulo *p*_2_ and *p*_3_.

When A requests the challenge ciphertext for messages *M*_0_, *M*_1_, identity vector $\left(I_{1}^{*},\cdots ,I_{l}^{*}\right)$ and T*, B makes a ciphertext-type query to the oracle for each $I_{i}^{*}$ and T*. When B makes a ciphertext-type query for some identity I*, O responds by choosing a random *t* ∈ *Z*_*N*_ and returning $\left(w^{s}{\left(Y_{2}Y_{3}\right)}^{\delta_{1}}{\left(X_{1}X_{2}\right)}^{ct^{\prime }},{\left(X_{1}X_{2}\right)}^{t^{\prime }}g^{t_{3}},{\left(X_{1}X_{2}\right)}^{aI^{*}+b}g_{3}^{t_{3}^{\prime }}\right)$ to B. When B makes a ciphertext-type query for some time T*, O responds by choosing a random *t*_0_ ∈ *Z*_*N*_ and returning $\left(w_{0}^{s}{\left(Y_{2}Y_{3}\right)}^{\delta_{2}}{\left(X_{1}X_{2}\right)}^{c_{0}t^{\prime }},{\left(X_{1}X_{2}\right)}^{t^{\prime }}g^{t_{3}},{\left(X_{1}X_{2}\right)}^{a_{0}T^{*}+b_{0}}g_{3}^{t_{3}^{\prime }}\right)$ to B. This implicitly sets $g^{t}=X_{1}^{t^{\prime }}$. It also sets *a*′ = *a* and *b*′ = *b* modulo *p*_2_ or *a*′ = *a*_0_ and *b*′ = *b*_0_ modulo *p*_2_,

which are properly distributed because *a*, *b* modulo *p*_2_ and *a*_0_, *b*_0_ modulo *p*_2_ do not appear elsewhere. Then B creats the ESF-5 ciphertexts successfully.

Upon receiving a challenge IBE-key-type query for *T* ∈ *Z*_*n*_, O chooses *r*, *y*′, *z* ∈ *Z*_*n*_ randomly and returns the group elements

$$\begin{array}{c} \left(w_{0}^{y^{\prime }}g_{3}^{y^{\prime }\psi },g^{y^{\prime }}g_{3}^{y^{\prime }},g^{r}\left(Y_{2}Y_{3}\right),v_{0}^{y^{\prime }}{\left(u_{0}^{T}h_{0}\right)}^{r}{\left(Y_{2}Y_{3}\right)}^{z}\right) \end{array}$$

to B. And then B creats the ESF-3 update key by using the group elements.

When B creats the HIBE private key with the index pair (*h*, *i*_*c*_) for some identity vector (*I*_1_, ⋯, *I*_*j*_) in the index *h* node, the HIBE private key with an index pair (*h*, *i*_*c*_) is generated as follows:

1. *i*_*c*_ < *h*_*c*_: It randomly chooses *y*_1_, ⋯, *y*_*j*_, λ_1_, ⋯, λ_*j*−1_, *r*_1_, ⋯, *r*_*j*_, *z*, *z*′ ∈ *Z*_*n*_ and generates a ESF-4-SK *PSK*_*HIBE*,*h*_.
  $$\begin{array}{c} \begin{array}{c} K_{i,0}=g^{\lambda_{i}}w^{y_{i}},K_{i,1}=g^{y_{i}},K_{i,2}=g^{r_{i}},K_{i,3}={\left(u^{I_{i}}h\right)}^{r_{i}}v^{y_{i}},i\in \left\{1,\cdots ,j-1\right\}\\ K_{j,0}=g^{\gamma_{\theta }-\sum_{i=1}^{j-1}\lambda_{i}}w^{y_{j}}{\left(Y_{2}Y_{3}\right)}^{y_{j}\psi_{1}},K_{j,1}=g^{y_{j}}{\left(Y_{2}Y_{3}\right)}^{y_{j}},\\ K_{j,2}=v^{y_{j}}{\left(u^{I_{j}}h\right)}^{r_{j}}{\left(Y_{2}Y_{3}\right)}^{z},K_{j,3}=g^{r_{j}}{\left(Y_{2}Y_{3}\right)}^{z^{\prime }} \end{array} \end{array}$$
  That is a properly distribution ESF-4-SK.
2. *i*_*c*_ = *h*_*c*_: B chooses random values *y*_1_, ⋯, *y*_*j*−1_, λ_1_, ⋯, λ_*j*−1_, *r*_1_, ⋯, *r*_*j*−1_ ∈ *Z*_*n*_. B forms the challenge key as:
  $$\begin{array}{c} \begin{array}{c} K_{i,0}=g^{\lambda_{i}}w^{y_{i}},K_{i,1}=g^{y_{i}},K_{i,2}=g^{r_{i}},K_{i,3}={\left(u^{I_{j}}h\right)}^{r_{i}}v^{y_{i}},i\in \left\{1,\cdots ,j-1\right\}\\ K_{j,0}=g^{\gamma_{\theta }-\sum_{i=1}^{j-1}\lambda_{i}}T_{0},K_{j,1}=T_{1},K_{j,2}=T_{3},K_{j,3}=T_{2} \end{array} \end{array}$$
  where (*T*_0_, *T*_1_, *T*_2_, *T*_3_) is the challenge HIBE key queried to O who chooses a random *r*, *r*_1_, *r*_2_ ∈ *Z*_*N*_ and returns (*T*_0_, *T*_1_, *T*_2_, *T*_3_) = $\left(T^{d},T,T^{c}{\left(u^{I_{j}}h\right)}^{r}{\left(Y_{2}Y_{3}\right)}^{r_{1}},g^{r}{\left(Y_{2}Y_{3}\right)}^{r_{2}}\right)$ to B.
3. *i*_*c*_ > *h*_*c*_: It randomly chooses *y*_1_, ⋯, *y*_*j*_, λ_1_, ⋯, λ_*j*−1_, *r*_1_, ⋯, *r*_*j*_, *z*, *z*′ ∈ *Z*_*n*_ and generates a ESF-3-SK *PSK*_*HIBE*,*h*_.
  $$\begin{array}{c} \begin{array}{c} K_{i,0}=g^{\lambda_{i}}w^{y_{i}},K_{i,1}=g^{y_{i}},K_{i,2}=g^{r_{i}},K_{i,3}={\left(u^{I_{i}}h\right)}^{r_{i}}v^{y_{i}},i\in \left\{1,\cdots ,j-1\right\}\\ K_{j,0}=g^{\gamma_{\theta }-\sum_{i=1}^{j-1}\lambda_{i}}w^{y_{j}}g_{3}^{y_{j}\psi_{1}},K_{j,1}=g^{y_{j}}g_{3}^{y_{j}},\\ K_{j,2}=v^{y_{j}}{\left(u^{I_{j}}h\right)}^{r_{j}}{\left(Y_{2}Y_{3}\right)}^{z},K_{j,3}=g^{r_{j}}{\left(Y_{2}Y_{3}\right)}^{z^{\prime }} \end{array} \end{array}$$
  That is a properly distribution ESF-3-SK.

In the challenge HIBE key, it implicitly sets *g*^*y*′^ to be the *G*_*p*_1__ part of *T*. If *T* ∈ *G*_*p*_2_*p*_3__, then this matches the distribution of *O*_3.3_, and so this will be a properly distributed normal key and B is playing Game *F*3_*h*_*c*_−1_. If *T* ∈ *G*, then this matches the distribution of *O*_3.4_ and then B is playing Game *F*3_*h*_*c*__.

Hence, if a PPT attacker can distinguish between *F*3_*h*_*c*_−1_ and *F*3_*h*_*c*__ with non-negligible advantage, O can distinguish between *O*_3.3_ and *O*_3.4_ with non-negligible advantage. It means O can gain a non-negligible advantage against Assumption 4.

Thus, Under Assumptions 4, no PPT attacker can distinguish between *O*_3.3_ and *O*_3.4_ with non-negligible advantage. Thus, no PPT attacker can distinguish between *F*3_*h*_*c*_−1_ and *F*3_*h*_*c*__ with non-negligible advantage.

**Lemma 12** *Under Assumptions 4*, *no PPT attacker can distinguish between O*_3.4_ *and O*_3.5_ *with non-negligible advantage*. *So no PPT attacker can distinguish between G*_*ESF*′−4_ *and G*_*ESF*′−5_ *with non-negligible advantage*.

***Proof*** The proof of this lemma is almost the same as that of Lemma 11 except the generation of secret keys and update leys.

Upon receiving a challenge HIBE-key-type query for *I* ∈ *Z*_*n*_, O chooses *r*, *y*′, *z*, *z*′ ∈ *Z*_*n*_ randomly and returns the group elements

$$\begin{array}{c} \left(w^{y^{\prime }}{\left(Y_{2}Y_{3}\right)}^{y^{\prime }\psi },g^{y^{\prime }}{\left(Y_{2}Y_{3}\right)}^{y^{\prime }},g^{r}{\left(Y_{2}Y_{3}\right)}^{z},v^{y^{\prime }}{\left(u^{I}h\right)}^{r}{\left(Y_{2}Y_{3}\right)}^{z^{\prime }}\right) \end{array}$$

to B. And then B creats the ESF-4 secret key by using the group elements.

When B creats the IBE private key with the index pair (*h*, *i*_*c*_) for some time T for the identity vector (*I*_1_, ⋯, *I*_*j*−1_) in the index *h* node, the update key with an index pair (*h*, *i*_*c*_) is generated as follows:

1. *i*_*c*_ < *h*_*c*_: It randomly chooses *y*_1_, ⋯, *y*_*j*_, λ_1_, ⋯, λ_*j*−1_, *r*_0_, ⋯, *r*_*j*−1_, *z*, *z*′ ∈ *Z*_*n*_ and generates a ESF-4-UK *TUK*_*IBE*,*h*_.
  $$\begin{array}{c} \begin{array}{c} U_{0,0}=g^{\alpha -\gamma_{\theta }-\sum_{i=1}^{j-1}\lambda_{i}}w_{0}^{y_{0}}{\left(Y_{2}Y_{3}\right)}^{y_{0}\psi_{2}},U_{0,1}=g^{y_{0}}{\left(Y_{2}Y_{3}\right)}^{y_{0}},\\ U_{0,2}=v_{0}^{y_{0}}{\left(u_{0}^{T}h_{0}\right)}^{r_{0}}{\left(Y_{2}Y_{3}\right)}^{z},U_{0,3}=g^{r_{0}}{\left(Y_{2}Y_{3}\right)}^{z^{\prime }},\\ U_{i,0}=g^{\lambda_{i}}w^{y_{i}},U_{i,1}=g^{y_{i}},U_{i,2}=g^{r_{i}},U_{i,3}={\left(u^{I_{i}}h\right)}^{r_{i}}v^{y_{i}},i\in \left\{1,\cdots ,j-1\right\} \end{array} \end{array}$$
  That is a properly distribution ESF-4-UK.
2. *i*_*c*_ = *h*_*c*_: B chooses random values *y*_1_, ⋯, *y*_*j*−1_, λ_1_, ⋯, λ_*j*−1_, *r*_1_, ⋯, *r*_*j*−1_ ∈ *Z*_*n*_. B forms the challenge key as:
  $$\begin{array}{c} \begin{array}{c} U_{0,0}=g^{\alpha -\gamma_{\theta }-\sum_{i=1}^{j-1}\lambda_{i}}T_{0},U_{0,1}=T_{1},U_{0,2}=T_{3},U_{0,3}=T_{2}\\ U_{i,0}=g^{\lambda_{i}}w^{y_{i}},U_{i,1}=g^{y_{i}},U_{i,2}=g^{r_{i}},U_{i,3}={\left(u^{I_{j}}h\right)}^{r_{i}}v^{y_{i}},i\in \left\{1,\cdots ,j-1\right\} \end{array} \end{array}$$
  where (*T*_0_, *T*_1_, *T*_2_, *T*_3_) is the challenge IBE key queried to O who chooses a random *r*, *r*_1_, *r*_2_ ∈ *Z*_*N*_ and returns (*T*_0_, *T*_1_, *T*_2_, *T*_3_) = $\left(T^{d_{0}},T,T^{c_{0}}{\left(u_{0}^{T}h_{0}\right)}^{r}{\left(Y_{2}Y_{3}\right)}^{r_{1}},g^{r}{\left(Y_{2}Y_{3}\right)}^{r_{2}}\right)$ to B.
3. *i*_*c*_ > *h*_*c*_: It randomly chooses *y*_0_, *y*_1_, ⋯, *y*_*j*−1_, λ_1_, ⋯, λ_*j*−1_, *r*_1_, ⋯, *r*_*j*_, *z*, *z*′ ∈ *Z*_*n*_ and generates a ESF-3-UK *TUK*_*IBE*,*h*_.
  $$\begin{array}{c} \begin{array}{c} U_{0,0}=g^{\alpha -\gamma_{\theta }-\sum_{i=1}^{j-1}\lambda_{i}}w_{0}^{y_{0}}g_{3}^{y_{0}\psi_{1}},U_{0,1}=g^{y_{0}}g_{3}^{y_{0}},U_{0,2}=v_{0}^{y_{0}}{\left(u_{0}^{T}h_{0}\right)}^{r_{0}}{\left(Y_{2}Y_{3}\right)}^{z},\\ U_{0,3}=g^{r_{j}}{\left(Y_{2}Y_{3}\right)}^{z^{\prime }}\\ U_{i,0}=g^{\lambda_{i}}w^{y_{i}},U_{i,1}=g^{y_{i}},U_{i,2}=g^{r_{i}},U_{i,3}={\left(u^{I_{i}}h\right)}^{r_{i}}v^{y_{i}},i\in \left\{1,\cdots ,j-1\right\} \end{array} \end{array}$$
  That is a properly distribution ESF-3-UK.

In the challenge IBE key, it implicitly sets *g*^*y*′^ to be the *G*_*p*_1__ part of *T*. If *T* ∈ *G*_*p*_2_*p*_3__, then this matches the distribution of *O*_3.4_, and so this will be a properly distributed normal key and B is playing Game *F*4_*h*_*c*_−1_. If *T* ∈ *G*, then this matches the distribution of *O*_3.5_ and then B is playing Game *F*4_*h*_*c*__.

Hence, if a PPT attacker can distinguish between *F*4_*h*_*c*_−1_ and *F*4_*h*_*c*__ with non-negligible advantage, O can distinguish between *O*_3.4_ and *O*_3.5_ with non-negligible advantage. It means O can gain a non-negligible advantage against Assumption 4.

Thus, Under Assumptions 4, no PPT attacker can distinguish between *O*_3.4_ and *O*_3.5_ with non-negligible advantage. Thus, no PPT attacker can distinguish between *F*4_*h*_*c*_−1_ and *F*4_*h*_*c*__ with non-negligible advantage.

**Lemma 13** *Under Assumptions 4*, *no PPT attacker can distinguish between O*_3.5_ *and O*_3.6_ *with non-negligible advantage*. *So no PPT attacker can distinguish between G*_*ESF*′−5_ *and G*_*ESF*′−6_ *with non-negligible advantage*.

***Proof*** We assume B interacts with one of *O*_3.5_, *O*_3.6_. O receives *g*, *g*_2_, *X*_1_ *X*_3_, *Y*_2_ *Y*_3_, *T*. O will simulate either *O*_3.5_ or *O*_3.6_ with B, depending on the value of *T* (which is either in *G* or *G*_*p*_1_*p*_2__). O picks values *a*, *b*, *c*, *d*, *a*_0_, *b*_0_, *c*_0_, *d*_0_ ∈ *Z*_*N*_ uniformly at random and sets *u* = *g*^*a*^, *h* = *g*^*b*^, *v* = *g*^*c*^, *w* = *g*^*d*^, $u_{0}=g^{a_{0}}$, $h_{0}=g^{b_{0}}$, $v_{0}=g^{c_{0}}$, $w_{0}=g^{d_{0}}$. B initially obtains the group elements

$$\begin{array}{c} \begin{array}{c} g,u,v,w,T,w^{y}{\left(Y_{2}Y_{3}\right)}^{d},g^{y}\left(Y_{2}Y_{3}\right),v^{y}{\left(Y_{2}Y_{3}\right)}_{1}^{\sigma },\\ u_{0},v_{0},w_{0},w_{0}^{y_{0}}{\left(Y_{2}Y_{3}\right)}^{d_{0}},g^{y_{0}}{\left(Y_{2}Y_{3}\right)}^{z},v_{0}^{y_{0}}{\left(Y_{2}Y_{3}\right)}^{\sigma_{2}} \end{array} \end{array}$$ (88)

from its oracle simulator where *z*, *y*_0_, *y*, *σ*_1_, *σ*_2_ ∈ *Z*_*p*_ are randomly chosen. We note that this set *ψ* = *d* modulo *p*_2_ and *p*_3_. If *T* ∈ *G*_*p*_1_*p*_2__, then this matches the initial elements provided by *O*_3.6_. If *T* ∈ *G*, then this matches the initial elements provided by *O*_3.5_. It chooses *α* ∈ *Z*_*n*_ randomly, and gives A the following public parameters in Eq 83. We note that B knows the master secret key *α*. When A requests a normal update key or a normal decryption key, B can responds by using the usual key generation algorithm, since it knows *α*.

When A makes a secret key query for the identity *ID*|_*j*_ = (*I*_1_, ⋯, *I*_*j*_), then B makes its challenge HIBE-key-type query for *I*_*j*_, O responds as follows. It chooses *y*′, *r*, *r*_1_, *r*_2_ ∈ *Z*_*N*_ randomly and responds with:

$$\begin{array}{c} \left({\left(X_{1}X_{3}g_{2}\right)}^{dy^{\prime }},{\left(X_{1}X_{3}g_{2}\right)}^{y^{\prime }},{\left(X_{1}X_{3}g_{2}\right)}^{cy^{\prime }}{\left(u^{I_{j}}h\right)}^{r}{\left(Y_{2}Y_{3}\right)}^{r_{1}},g^{r}{\left(Y_{2}Y_{3}\right)}^{r_{2}}\right) \end{array}$$

And then B creats the ESF-4 secret key by using the group elements.

Upon receiving a challenge IBE-key-type query for *T* ∈ *Z*_*n*_, O chooses *y*′, *r*, *r*_1_, *r*_2_ ∈ *Z*_*N*_ randomly and responds with:

$$\begin{array}{c} \left({\left(X_{1}X_{3}g_{2}\right)}^{d_{0}y^{\prime }},{\left(X_{1}X_{3}g_{2}\right)}^{y^{\prime }},{\left(X_{1}X_{3}g_{2}\right)}^{c_{0}y^{\prime }}{\left(u_{0}^{T}h_{0}\right)}^{r}{\left(Y_{2}Y_{3}\right)}^{r_{1}},g^{r}{\left(Y_{2}Y_{3}\right)}^{r_{2}}\right) \end{array}$$

to B. And then B creats the ESF-4 update key by using the group elements.

When A requests the challenge ciphertext for messages *M*_0_, *M*_1_, identity vector $\left(I_{1}^{*},\cdots ,I_{l}^{*}\right)$ and T*, B makes a ciphertext-type query to the oracle for each $I_{i}^{*}$ and T*. In response to each query for $I_{i}^{*}$ or T*, O gets random *t*_0_, *t*_1_, …, *t*_*l*_ ∈ *Z*_*p*_, chooses *β* ∈ {0, 1} and creats the ciphertext as

$$\begin{array}{c} \left(C=M_{\beta }e{\left(X_{1}X_{3},g\right)}^{\alpha },C_{0}=X_{1}X_{3},{\left\{C_{i,1},C_{i,2},C_{i,3}\right\}}_{i=0}^{l}\right) \end{array}$$

where the ciphertext-element-group (*C*_*i*,1_, *C*_*i*,2_, *C*_*i*,3_) is defined as follows if *i* > 0:

$$\begin{array}{c} \left(T^{d}T^{ct_{3}}v^{t_{i}}g_{2}^{\sigma_{1}t_{i}},T^{t_{3}}g^{t_{i}}g_{2}^{t_{i}},T^{t_{3}\left(aI_{i}^{*}+b\right)}g_{2}^{t_{i}\left(aI_{i}^{*}+b\right)}\right) \end{array}$$

and (*C*_0,1_, *C*_0,2_, *C*_0,3_) is defined as follows

$$\begin{array}{c} \left(T^{d_{0}}T^{c_{0}t_{3}}v_{0}^{t_{0}}g_{2}^{\sigma_{2}t_{0}},T^{t_{3}}g^{t_{0}}g_{2}^{t_{0}},T^{t_{3}\left(a_{0}T^{*}+b_{0}\right)}g_{2}^{t_{0}\left(a_{0}T^{*}+b_{0}\right)}\right) \end{array}$$

We note that this is very similar to the way O behaves in the proof of Lemma 12. The only difference is the $g_{2}^{dy^{\prime }},g_{2}^{y^{\prime }},g_{2}^{cy^{\prime }}$ terms which have been added to the challenge key. As in the proof of Lemma 12, we have that if *T* ∈ *G*, the *G*_*p*_3__ components of the challenge ciphertext are properly distributed as in a response from *O*_3.5_, since the value of *c* modulo *p*_3_ is not revealed by the challenge key-type response (it is hidden by the random term $Y_{3}^{r_{1}}$). Also as in the proof of Lemma 12, we have that the *G*_*p*_2__ components of the ciphertext-type responses are properly distributed. Thus, if *T* ∈ *G*_*p*_1_*p*_2__, O has properly simulated the responses of *O*_3.6_, and when *T* ∈ *G*, O has properly simulated the responses of *O*_3.6_.

Hence, if a PPT attacker can distinguish any pair between *G*_*ESF*′−5_ and *G*_*ESF*′−6_ with non-negligible advantage, O can distinguish the corresponding pair between *O*_3.5_ and *O*_3.6_ with non-negligible advantage. It means O can use the output of B to achieve a non-negligible advantage against Assumption 4.

Thus, Under Assumptions 4, no PPT attacker can distinguish between *O*_3.5_ and *O*_3.6_ with non-negligible advantage. Thus, no PPT attacker can distinguish between *G*_*ESF*′−5_ and *G*_*ESF*′−6_ with non-negligible advantage.

**Lemma 14** *Under Assumptions 4*, *no PPT attacker can distinguish between O*_3.6_ *and O*_7/2′_ *with non-negligible advantage*. *So no PPT attacker can distinguish between F*6_*i*−1,2_ *and F*6_*i*,1_ *with non-negligible advantage*.

***Proof*** We assume B interacts with one of *O*_3.6_ and *O*_7/2′_. O receives *g*, *g*_3_, *X*_1_ *X*_2_, *Y*_2_ *Y*_3_, *T*. O will simulate either *O*_3.6_ and *O*_7/2′_ with B, depending on the value of *T* (which is either in *G* or *G*_*p*_1_*p*_3__). O picks values *a*, *b*, *c*, *d*, *a*_0_, *b*_0_, *c*_0_, *d*_0_ ∈ *Z*_*N*_ uniformly at random and sets *u* = *g*^*a*^, *h* = *g*^*b*^, *v* = *g*^*c*^, *w* = *g*^*d*^, $u_{0}=g^{a_{0}}$, $h_{0}=g^{b_{0}}$, $v_{0}=g^{c_{0}}$, $w_{0}=g^{d_{0}}$. B initially obtains the group elements

$$\begin{array}{c} \begin{array}{c} g,u,h,v,w,X_{1}X_{2},w^{y}{\left(Y_{2}Y_{3}\right)}^{y\psi_{1}},g^{y}{\left(Y_{2}Y_{3}\right)}^{y},v^{y}{\left(Y_{2}Y_{3}\right)}^{y\sigma_{1}}\\ u_{0},h_{0},v_{0},w_{0},w_{0}^{y^{\prime }}{\left(Y_{2}Y_{3}\right)}^{y^{\prime }\psi_{2}},g^{y^{\prime }}{\left(Y_{2}Y_{3}\right)}^{y^{\prime }},v_{0}^{y^{\prime }}{\left(Y_{2}Y_{3}\right)}^{y^{\prime }\sigma_{2}} \end{array} \end{array}$$ (89)

from its oracle simulator who additionally chooses *ψ*_1_, *ψ*_2_, *σ*_1_, *σ*_2_, *y*, *y*′ ∈ *Z*_*N*_ randomly. These are properly distributed with *g*^*s*^ implicitly set to be *X*_1_.

When A requests the challenge ciphertext for messages *M*_0_, *M*_1_, identity vector $\left(I_{1}^{*},\cdots ,I_{l}^{*}\right)$ and T*, B makes a ciphertext-type query to the oracle for each $I_{i}^{*}$ and T*. When B makes a ciphertext-type query for some identity $I_{i}^{*}$, O responds by choosing a random $t_{i}^{\prime }\in Z_{N}$ and returning $\left({\left(X_{1}X_{2}\right)}^{d}{\left(X_{1}X_{2}\right)}^{ct_{i}^{\prime }},{\left(X_{1}X_{2}\right)}^{t_{i}^{\prime }\left(aI_{i}^{*}+b\right)},{\left(X_{1}X_{2}\right)}^{t_{i}^{\prime }}\right)$ to B. When B makes a ciphertext-type query for some time T*, O responds by choosing a random *t*_0_ ∈ *Z*_*N*_ and returning $\left({\left(X_{1}X_{2}\right)}^{d_{0}}{\left(X_{1}X_{2}\right)}^{c_{0}t_{0}^{\prime }},{\left(X_{1}X_{2}\right)}^{t_{0}^{\prime }\left(a_{0}T^{*}+b_{0}\right)},{\left(X_{1}X_{2}\right)}^{t_{0}^{\prime }}\right)$ to B. This sets $X_{2}^{d}=g_{2}^{\delta_{1}}$ and $X_{2}^{d_{0}}=g_{2}^{\delta_{2}}$, which is uniformly random because the value of *d* and *d*_0_ modulo *p*_2_ will not appear elsewhere. It implicitly sets $g^{t_{i}}=X_{1}^{t_{i}^{\prime }}$. This is identically distributed to a response from *O*_6_ and *O*_7/2′_, with *a*′, *b*′ equal to *a*, *b* modulo *p*_2_, and *σ*_1_ = *c* modulo *p*_2_, *σ*_2_ = *c*_0_ modulo *p*_2_. We note that this is in the only context in which the values of *a*, *b* modulo *p*_2_ appear, so this is equivalent to choosing *a*′, *b*′ independently at random. Then B creats the ESF-1 ciphertexts successfully.

When A requests the secret key of an identity vector *ID*|_*j*_ = (*I*_1_, ⋯, *I*_*j*_), B creats the ESF-4-SK key by the HIBE-type query responses from O who randomly chooses *y*_1_, ⋯, *y*_*j*_, λ_1_, ⋯, λ_*j*−1_, *r*_1_, ⋯, *r*_*j*_, *z*, *z*′ ∈ *Z*_*n*_ and generates a ESF-4-SK *PSK*_*HIBE*,*h*_*θ*__ for every *θ*

$$\begin{array}{c} \begin{array}{c} K_{i,0}=g^{\lambda_{i}}w^{y_{i}},K_{i,1}=g^{y_{i}},K_{i,2}=g^{r_{i}},K_{i,3}={\left(u^{I_{i}}h\right)}^{r_{i}}v^{y_{i}},i\in \left\{1,\cdots ,j-1\right\}\\ K_{j,0}=g^{\gamma_{\theta }-\sum_{i=1}^{j-1}\lambda_{i}}w^{y_{j}}{\left(Y_{2}Y_{3}\right)}^{y_{j}\psi_{1}},K_{j,1}=g^{y_{j}}{\left(Y_{2}Y_{3}\right)}^{y_{j}},\\ K_{j,2}=v^{y_{j}}{\left(u^{I_{j}}h\right)}^{r_{j}}{\left(Y_{2}Y_{3}\right)}^{z},K_{j,3}=g^{r_{j}}{\left(Y_{2}Y_{3}\right)}^{z^{\prime }} \end{array} \end{array}$$

When B creats the IBE private key with the index pair (*h*, *i*_*c*_) for some time T for the identity vector (*I*_1_, ⋯, *I*_*j*−1_) in the index *h* node, the update key with an index pair (*h*, *i*_*c*_) is generated as follows:

1. *i*_*c*_ < *h*_*c*_: It randomly chooses *y*_0_, ⋯, *y*_*j*−1_, λ_1_, ⋯, λ_*j*−1_, $r_{0}^{\prime },r_{1},\cdots ,r_{j-1}$, *z*, *z*′ ∈*Z*_*n*_ and generates a semi-functional update key *TUK*_*ID*|_*l*_,*T*,*θ*_*h*__.
  $$\begin{array}{c} \begin{array}{c} U_{0,0}=g^{\alpha -\gamma_{\theta_{h}}-\sum_{i=1}^{j-1}\lambda_{i}}w_{0}^{y_{0}}{\left(Y_{2}Y_{3}\right)}^{y_{0}\psi_{2}},U_{0,1}=g^{y_{0}}{\left(Y_{2}Y_{3}\right)}^{y_{0}},U_{0,2}=g^{r_{0}},\\ U_{0,3}=v_{0}^{y_{0}}{\left(Y_{2}Y_{3}\right)}^{y_{0}\sigma_{2}}{\left(u_{0}^{T}h_{0}\right)}^{r_{0}}\\ U_{i,0}=g^{\lambda_{i}}w^{y_{i}},U_{i,1}=g^{y_{i}},U_{i,2}=g^{r_{i}},U_{i,3}={\left(u^{I_{i}}h\right)}^{r_{i}}v^{y_{i}},i\in \left\{1,\cdots ,j-1\right\} \end{array} \end{array}$$
2. *i*_*c*_ = *h*_*c*_: B chooses random values *y*_1_, ⋯, *y*_*j*−1_, λ_1_, ⋯, λ_*j*−1_, *r*_1_, ⋯, *r*_*j*−1_ ∈ *Z*_*n*_. B forms the challenge key as:
  $$\begin{array}{c} \begin{array}{c} U_{0,0}=g^{\alpha -\gamma_{\theta_{h}}-\sum_{i=1}^{j-1}\lambda_{i}}T_{0},U_{0,1}=T_{1},U_{0,2}=T_{3},U_{0,3}=T_{2}\\ U_{i,0}=g^{\lambda_{i}}w^{y_{i}},U_{i,1}=g^{y_{i}},U_{i,2}=g^{r_{i}},U_{i,3}={\left(u^{I_{i}}h\right)}^{r_{i}}v^{y_{i}},i\in \left\{1,\cdots ,j-1\right\} \end{array} \end{array}$$
  where (*T*_0_, *T*_1_, *T*_2_, *T*_3_) is the challenge IBE key queried to O who chooses a random *y*_0_ ∈ *Z*_*N*_ and returns (*T*_0_, *T*_1_, *T*_2_, *T*_3_) = $\left(w_{0}^{y_{0}}{\left(Y_{2}Y_{3}\right)}^{\psi_{2}y_{0}},g^{y_{0}}{\left(Y_{2}Y_{3}\right)}^{y_{0}},v_{0}^{y_{0}}{\left(Y_{2}Y_{3}\right)}^{\sigma_{2}y_{0}}T^{a_{0}T+b_{0}},T\right)$ to B.
3. *i*_*c*_ > *h*_*c*_: It generates a ESF-4-UK as
  $$\begin{array}{c} \begin{array}{c} U_{0,0}=g^{\alpha -\gamma_{\theta_{h}}-\sum_{i=1}^{j-1}\lambda_{i}}(w_{0}^{y_{0}}{\left(Y_{2}Y_{3}\right)}^{\psi_{2}y_{0}},U_{0,1}=g^{y_{0}}{\left(Y_{2}Y_{3}\right)}^{y_{0}},U_{0,2}=g^{r_{0}}{\left(Y_{2}Y_{3}\right)}^{z},\\ U_{0,3}=v_{0}^{y_{0}}{\left(u_{0}^{T}h_{0}\right)}^{r_{0}}{\left(Y_{2}Y_{3}\right)}^{z^{\prime }}\\ U_{i,0}=g^{\lambda_{i}}w^{y_{i}},U_{i,1}=g^{y_{i}},U_{i,2}=g^{r_{i}},U_{i,3}={\left(u^{I_{i}}h\right)}^{r_{i}}v^{y_{i}},i\in \left\{1,\cdots ,j-1\right\} \end{array} \end{array}$$
  where *z*, *z*′ ∈ *Z*_*p*_ are randomly chosen.

In the challenge IBE key, it implicitly sets *g*^*r*^ to be the *G*_*p*_1__ part of *T*. We note that *a*_0_, *b*_0_ modulo *p*_2_,*p*_3_ are uniformly random and do not appear elsewhere. If *T* ∈ *G*_*p*_1_*p*_3__, then this matches the distribution of *O*_6_, and so this will be a properly distributed normal key and B is playing Game *F*6_*h*_*c*_−1,2_. If *T* ∈ *G*, then this matches the distribution of *O*_7/2′_ (note random *G*_*p*_3__ terms attached to the last two group elements) and then B is playing Game *F*6_*h*_*c*_,1_.

Hence, if a PPT attacker can distinguish between *F*6_*h*_*c*_−1,2_ and *F*6_*h*_*c*_,1_ with non-negligible advantage, O can distinguish between *O*_6_ and *O*_7/2′_ with non-negligible advantage. It means O can gain a non-negligible advantage against Assumption 4.

Thus, Under Assumptions 4, no PPT attacker can distinguish between *O*_6_ and *O*_7/2′_ with non-negligible advantage. Thus, no PPT attacker can distinguish between *F*6_*h*_*c*_−1,2_ and *F*6_*h*_*c*_,1_ with non-negligible advantage.

**Lemma 15** *Under Assumptions 3*, *no PPT attacker can distinguish between O*_7/2′_ *and* ${\tilde{O}}_{q_{c}}^{*}$ *with non-negligible advantage*. *So no PPT attacker can distinguish between F*6_*i*,1_ *and F*6_*i*,2_ *with non-negligible advantage*.

***Proof*** We assume B interacts with one of *O*_7/2′_, and ${\tilde{O}}_{q_{c}}^{*}$. O receives *g*, *g*_2_, *X*_1_ *X*_3_, *T*. O will simulate either *O*_7/2′_ or ${\tilde{O}}_{q_{c}}^{*}$ with B, depending on the value of *T* (which is either in *G*_*p*_1__ or *G*_*p*_1_*p*_3__). O picks values *a*, *b*, *c*, *d*, *a*_0_, *b*_0_, *c*_0_, *d*_0_ ∈ *Z*_*N*_ uniformly at random and sets *u* = *g*^*a*^, *h* = *g*^*b*^, *v* = *g*^*c*^, *w* = *g*^*d*^, $u_{0}=g^{a_{0}}$, $h_{0}=g^{b_{0}}$, $v_{0}=g^{c_{0}}$, $w_{0}=g^{d_{0}}$. B initially obtains the group elements

$$\begin{array}{c} \begin{array}{c} g,u,h,v,w,g^{s}g_{2}^{\gamma },{\left(X_{1}X_{3}\right)}^{d}g_{2}^{dy},\left(X_{1}X_{3}\right)g_{2}^{y},{\left(X_{1}X_{3}\right)}^{c}g_{2}^{y^{\prime }},\\ u_{0},h_{0},v_{0},w_{0},{\left(X_{1}X_{3}\right)}^{zd_{0}}g_{2}^{d_{0}y^{\prime }},{\left(X_{1}X_{3}\right)}^{z}g_{2}^{y^{\prime }},{\left(X_{1}X_{3}\right)}^{zc_{0}}g_{2}^{y^{\prime }} \end{array} \end{array}$$ (90)

from its oracle simulator, and gives A the public parameters in Eq 83.

When A requests the challenge ciphertext for messages *M*_0_, *M*_1_, identity vector $\left(I_{1}^{*},\cdots ,I_{l}^{*}\right)$ and T*, B makes a ciphertext-type query to the oracle for each $I_{i}^{*}$ and T*. When B makes a ciphertext-type query for some identity I*, O responds by choosing a random *t* ∈ *Z*_*N*_ and returning $\left(w^{s}g_{2}^{\delta_{1}}v^{t}g_{2}^{\sigma_{1}t},g^{t}g_{2}^{t},{\left(u^{I^{*}}h\right)}^{t}g_{2}^{t(a^{\prime }I^{*}+b^{\prime })}\right)$ to B as same as Eq 10. When B makes a ciphertext-type query for some time T*, O responds by choosing a random *t*_0_ ∈ *Z*_*N*_ and returning $\left(w_{0}^{s}g_{2}^{\delta_{2}}v_{0}^{t_{0}}g_{2}^{\sigma_{2}t_{0}},g^{t_{0}}g_{2}^{t_{0}},{\left(u_{0}^{T^{*}}h_{0}\right)}^{t_{0}}g_{2}^{t_{0}\left(a^{\prime }T^{*}+b^{\prime }\right)}\right)$ to B. Then B creats the ESF-1 ciphertexts successfully.

When A requests the secret key of an identity vector *ID*|_*j*_ = (*I*_1_, ⋯, *I*_*j*_), B creats the ESF-4-SK key by the HIBE-type query response from O and the secret key for *ID*|_*j*_ in some node *θ* is

$$\begin{array}{c} \begin{array}{c} K_{i,0}=g^{\lambda_{i}}w^{y_{i}},K_{i,1}=g^{y_{i}},K_{i,2}=g^{r_{i}},K_{i,3}={\left(u^{I_{i}}h\right)}^{r_{i}}v^{y_{i}},i\in \left\{1,\cdots ,j-1\right\}\\ K_{j,0}=g^{\gamma_{\theta }-\sum_{i=1}^{j-1}\lambda_{i}}{\left(X_{1}X_{3}g_{2}\right)}^{dy_{j}^{\prime }},K_{j,1}={\left(X_{1}X_{3}g_{2}\right)}^{y_{j}^{\prime }},\\ K_{j,2}={\left(X_{1}X_{3}\right)}^{r_{j}^{\prime }}g_{2}^{z^{\prime }},K_{j,3}={\left(X_{1}X_{3}g_{2}\right)}^{cy_{j}^{\prime }}{\left(X_{1}X_{3}\right)}^{r_{j}^{\prime }\left(aI_{j}+b\right)}g_{2}^{z} \end{array} \end{array}$$

where $y_{1},\cdots ,y_{j-1},y_{j}^{\prime }$, $\lambda_{1},\cdots ,\lambda_{j-1},r_{1},\cdots ,r_{j-1},r_{j}^{\prime },z,z^{\prime }\in Z_{n}$ are randomly chosen.

When B creats the IBE private key with the index pair (*h*, *i*_*c*_) for some time T for the identity vector (*I*_1_, ⋯, *I*_*j*−1_) in the index *h* node, the update key with an index pair (*h*, *i*_*c*_) is generated as follows:

1. *i*_*c*_ < *h*_*c*_: It randomly chooses $y_{0}^{\prime },y_{1},\cdots ,y_{j-1}$, λ_1_, ⋯, λ_*j*−1_, *r*_0_, *r*_1_, ⋯, *r*_*j*−1_, *z*, *z*′ ∈*Z*_*n*_ and generates a semi-functional update key *TUK*_*ID*|_*l*_,*T*,*θ*_*h*__
  $$\begin{array}{c} \begin{array}{c} U_{0,0}=g^{\alpha -\gamma_{\theta_{h}}-\sum_{i=1}^{j-1}\lambda_{i}}{\left(X_{1}X_{3}g_{2}\right)}^{d_{0}y_{0}^{\prime }},U_{0,1}={\left(X_{1}X_{3}g_{2}\right)}^{y_{0}^{\prime }},U_{0,2}=g^{r_{0}},\\ U_{0,3}={\left(X_{1}X_{3}g_{2}\right)}^{c_{0}y_{0}^{\prime }}{\left(u_{0}^{T}h_{0}\right)}^{r_{0}}\\ U_{i,0}=g^{\lambda_{i}}w^{y_{i}},U_{i,1}=g^{y_{i}},U_{i,2}=g^{r_{i}},U_{i,3}={\left(u^{I_{i}}h\right)}^{r_{i}}v^{y_{i}},i\in \left\{1,\cdots ,j-1\right\} \end{array} \end{array}$$
2. *i*_*c*_ = *h*_*c*_: B chooses random values *y*_1_, ⋯, *y*_*j*−1_, λ_1_, ⋯, λ_*j*−1_, *r*_1_, ⋯, *r*_*j*−1_ ∈ *Z*_*n*_. B forms the challenge key as:
  $$\begin{array}{c} \begin{array}{c} U_{0,0}=g^{\alpha -\gamma_{\theta_{h}}-\sum_{i=1}^{j-1}\lambda_{i}}T_{0},U_{0,1}=T_{1},U_{0,2}=T_{3},U_{0,3}=T_{2}\\ U_{i,0}=g^{\lambda_{i}}w^{y_{i}},U_{i,1}=g^{y_{i}},U_{i,2}=g^{r_{i}},U_{i,3}={\left(u^{I_{i}}h\right)}^{r_{i}}v^{y_{i}},i\in \left\{1,\cdots ,j-1\right\} \end{array} \end{array}$$
  where (*T*_0_, *T*_1_, *T*_2_, *T*_3_) is the challenge IBE key queried to O who chooses a random *y*_0_ ∈ *Z*_*N*_ and returns (*T*_0_, *T*_1_, *T*_2_, *T*_3_) = $({\left(X_{1}X_{3}g_{2}\right)}^{d_{0}y_{0}^{\prime }},{\left(X_{1}X_{3}g_{2}\right)}^{y_{0}^{\prime }},$ ${\left(X_{1}X_{3}g_{2}\right)}^{c_{0}y_{0}^{\prime }}T^{a_{0}T+b_{0}},$ *T*). This implicitly sets *g*^*r*^ to be the *G*_*p*_1__ part of *T*.
3. *i*_*c*_ > *h*_*c*_: It generates a ESF-4-UK as
  $$\begin{array}{c} \begin{array}{c} U_{0,0}=g^{\alpha -\gamma_{\theta_{h}}-\sum_{i=1}^{j-1}\lambda_{i}}{\left(X_{1}X_{3}g_{2}\right)}^{d_{0}y_{0}^{\prime }},U_{0,1}={\left(X_{1}X_{3}g_{2}\right)}^{y_{0}^{\prime }},\\ U_{0,2}={\left(X_{1}X_{3}\right)}^{r_{0}^{\prime }}g_{2}^{z^{\prime }},U_{0,3}={\left(X_{1}X_{3}g_{2}\right)}^{c_{0}y_{0}^{\prime }}{\left(X_{1}X_{3}\right)}^{r_{0}^{\prime }\left(a_{0}T+b_{0}\right)}g_{2}^{z}\\ U_{i,0}=g^{\lambda_{i}}w^{y_{i}},U_{i,1}=g^{y_{i}},U_{i,2}=g^{r_{i}},U_{i,3}={\left(u^{I_{i}}h\right)}^{r_{i}}v^{y_{i}},i\in \left\{1,\cdots ,j-1\right\} \end{array} \end{array}$$
  where *z*, *z*′ ∈ *Z*_*p*_ are randomly chosen.

In the challenge IBE key, it implicitly sets $g^{r_{0}}$ to be the *G*_*p*_1__ part of *T*. We note that *a*_0_, *b*_0_ modulo *p*_2_,*p*_3_ are uniformly random and do not appear elsewhere. If *T* ∈ *G*_*p*_1_*p*_3__, then this matches the distribution of *O*_7/2′_, and so this will be a properly distributed normal key and B is playing Game *F*6_*h*_*c*_,1_. If *T* ∈ *G*_*p*_1__, then this matches the distribution of ${\tilde{O}}_{q_{c}}^{*}$ and then B is playing Game *F*6_*h*_*c*_,2_.

Hence, if a PPT attacker can distinguish between *F*6_*h*_*c*_,1_ and *F*6_*h*_*c*_,2_ with non-negligible advantage, O can distinguish between *O*_7/2′_ and ${\tilde{O}}_{q_{c}}^{*}$ with non-negligible advantage. It means O can gain a non-negligible advantage against Assumption 3.

Thus, Under Assumptions 3, no PPT attacker can distinguish between *O*_7/2′_ and ${\tilde{O}}_{q_{c}}^{*}$ with non-negligible advantage. Thus, no PPT attacker can distinguish between *F*6_*h*_*c*_,1_ and *F*6_*h*_*c*_,2_ with non-negligible advantage.

**Lemma 16** *Under Assumptions 3*, *no PPT attacker can distinguish between* ${\tilde{O}}_{k}^{*}$ *and* ${\tilde{O}}_{k}^{\prime \prime }$ *with non-negligible advantage*. *So no PPT attacker can distinguish between* $S_{k,1}^{\prime }$ *and* $S_{k,3}^{\prime }$ *with non-negligible advantage*.

***Proof*** We assume B interacts with one of ${\tilde{O}}_{k}^{\prime \prime },{\tilde{O}}_{k}^{*}$. O receives *g*, *g*_2_, *X*_1_ *X*_3_, *T*. O will simulate either ${\tilde{O}}_{k}^{\prime \prime }$ or ${\tilde{O}}_{k}^{*}$ with B, depending on the value of *T* (which is either in *G*_*p*_1__ or *G*_*p*_1_*p*_3__). B initially obtains the group elements in Eq 82

$$\begin{array}{c} \begin{array}{c} g,u,h,v,w,g^{s}g_{2}^{\gamma },{\left(X_{1}X_{3}\right)}^{d}g_{2}^{y_{2}d},\left(X_{1}X_{3}\right)g_{2}^{y_{2}},{\left(X_{1}X_{3}\right)}^{c}g_{2}^{cy_{2}}\\ u_{0},h_{0},v_{0},w_{0},{\left(X_{1}X_{3}\right)}^{zd_{0}}g_{2}^{y_{2}^{\prime }d_{0}},{\left(X_{1}X_{3}\right)}^{z}g_{2}^{y_{2}^{\prime }},{\left(X_{1}X_{3}\right)}^{zc_{0}}g_{2}^{c_{0}y_{2}^{\prime }} \end{array} \end{array}$$

from its oracle simulator.

When A requests the secret key of an identity vector *ID*|_*j*_ = (*I*_1_, ⋯, *I*_*j*_), B creats the ESF-4-SK key by the HIBE-type query response from O and the secret key for *ID*|_*j*_ in some node *θ* is

$$\begin{array}{c} \begin{array}{c} K_{i,0}=g^{\lambda_{i}}w^{y_{i}},K_{i,1}=g^{y_{i}},K_{i,2}=g^{r_{i}},K_{i,3}={\left(u^{I_{i}}h\right)}^{r_{i}}v^{y_{i}},i\in \left\{1,\cdots ,j-1\right\}\\ K_{j,0}=g^{\gamma_{\theta }-\sum_{i=1}^{j-1}\lambda_{i}}{\left(X_{1}X_{3}g_{2}\right)}^{dy_{j}^{\prime }},K_{j,1}={\left(X_{1}X_{3}g_{2}\right)}^{y_{j}^{\prime }},\\ K_{j,2}={\left(X_{1}X_{3}\right)}^{r_{j}^{\prime }}g_{2}^{z^{\prime }},K_{j,3}={\left(X_{1}X_{3}g_{2}\right)}^{cy_{j}^{\prime }}{\left(X_{1}X_{3}\right)}^{r_{j}^{\prime }\left(aI_{j}+b\right)}g_{2}^{z} \end{array} \end{array}$$

where $y_{1},\cdots ,y_{j-1},y_{j}^{\prime }$, $\lambda_{1},\cdots ,\lambda_{j-1},r_{1},\cdots ,r_{j-1},r_{j}^{\prime },z,z^{\prime }\in Z_{n}$ are randomly chosen.

When A requests the update key of an identity vector *ID*|_*j*_ = (*I*_1_, ⋯, *I*_*j*_) and the ime *T*, B creats the UK key by the IBE-type query response from O and the secret key for *ID*|_*j*_ in some node *θ* is generated as follows: O randomly chooses $y_{0}^{\prime },y_{1},\cdots ,y_{j-1}$, λ_1_, ⋯, λ_*j*−1_, *r*_0_, *r*_1_, ⋯, *r*_*j*−1_, *z*, *z*′ ∈*Z*_*n*_ and generates a semi-functional update key *TUK*_*ID*|_*l*_,*T*,*θ*_*h*__

$$\begin{array}{c} \begin{array}{c} U_{0,0}=g^{\alpha -\gamma_{\theta_{h}}-\sum_{i=1}^{j-1}\lambda_{i}}{\left(X_{1}X_{3}g_{2}\right)}^{d_{0}y_{0}^{\prime }},U_{0,1}={\left(X_{1}X_{3}g_{2}\right)}^{y_{0}^{\prime }},U_{0,2}=g^{r_{0}},\\ U_{0,3}={\left(X_{1}X_{3}g_{2}\right)}^{c_{0}y_{0}^{\prime }}{\left(u_{0}^{T}h_{0}\right)}^{r_{0}}\\ U_{i,0}=g^{\lambda_{i}}w^{y_{i}},U_{i,1}=g^{y_{i}},U_{i,2}=g^{r_{i}},U_{i,3}={\left(u^{I_{i}}h\right)}^{r_{i}}v^{y_{i}},i\in \left\{1,\cdots ,j-1\right\} \end{array} \end{array}$$

When A requests the challenge ciphertext for messages *M*_0_, *M*_1_, identity vector $\left(I_{1}^{*},\cdots ,I_{l}^{*}\right)$ and T*, B makes a ciphertext-type query to the oracle for each $I_{i}^{*}$ and T*. In response to each query for $I_{i}^{*}$ or T*, O gets random *t*_0_, *t*_1_, …, *t*_*l*_ ∈ *Z*_*p*_, chooses *β* ∈ {0, 1} and creats the ciphertext as

$$\begin{array}{c} \left(C=M_{\beta }e{\left(g^{s}g_{2}^{\gamma },g\right)}^{\alpha },C_{0}=g^{s}g_{2}^{\gamma },{\left\{C_{i,1},C_{i,2},C_{i,3}\right\}}_{i=0}^{l}\right) \end{array}$$

where the ciphertext-element-group (*C*_*i*,1_, *C*_*i*,2_, *C*_*i*,3_) is defined as follows:

1. *i* < *k*: If *i* = 0, O responds with the ciphertext-element-group $\left(w_{0}^{s}g_{2}^{\delta_{2}}v_{0}^{t_{0}}g_{2}^{c_{0}t_{0}},{\left(u_{0}^{T^{*}}h_{0}\right)}^{t_{0}}g_{2}^{\left(a^{\prime }T^{*}+b^{\prime }\right)t_{0}},g^{t_{0}}g_{2}^{t_{0}}\right)$, else the element group is $\left(w^{s}g_{2}^{\delta_{1}}v^{t_{i}}g_{2}^{ct_{i}},{\left(u^{I_{i}^{*}}h\right)}^{t_{i}}g_{2}^{\left(a^{\prime }I_{i}^{*}+b^{\prime }\right)t_{i}},g^{t_{i}}g_{2}^{t_{i}}\right)$. This is identically distributed to a response from *O*_2_.
2. *i* = *k*: O choses *z* ∈ *Z*_*p*_ randomly and responds with the ciphertext-element-group (*T*_1_, *T*_3_, *T*_2_) = $\left(w^{s}g_{2}^{\delta_{1}}T^{c}\cdot g_{2}^{cz},T^{t_{i}\left(aI_{i}^{*}+b\right)}g_{2}^{z(a^{\prime }I_{i}^{*}+b^{\prime })},Tg_{2}^{z}\right)$ if *k* > 0. Else if *k* = 0, O responds with $\left(w_{0}^{s}g_{2}^{\delta_{2}}T^{c_{0}}\cdot g_{2}^{c_{0}z},T^{t_{0}\left(a_{0}T^{*}+b_{0}\right)}g_{2}^{z(a^{\prime }T^{*}+b^{\prime })},Tg_{2}^{z}\right)$. We note that the *G*_*p*_2__ parts here are properly distributed, since *σ*_1_ = *c* modulo *p*_2_ and *σ*_2_ = *c*_0_ modulo *p*_2_.
3. *i* > *k*: The ciphertext-element-group is $\left(w^{s}g_{2}^{\delta_{1}}v^{t_{i}},{\left(u^{I_{i}^{*}}h\right)}^{t_{i}},g^{t_{i}}\right)$. This is identically distributed to a response from *O*_1_.

When *T* ∈ *G*_*p*_1__ the values of *a*, *b* modulo *p*_3_ only appear in the response to the challenge key-type query, which means that the *G*_*p*_3__ terms on the last two group elements there are uniformly random. Also, the response to the *k*^*th*^ ciphertext-type query is distributed exactly like a response from *O*_2_. In this case, O has properly simulated the responses of $O_{k}^{*}$ and this will be a properly distributed EST-2^k^-CT and so B is playing Game $S_{k,1}^{\prime }$.

When *T* ∈ *G*_*p*_1_*p*_3__, we must argue that the values *aI* + *b* and $aI_{k}^{*}+b$ appear uniformly random modulo *p*_3_: this follows by pairwise independence of *aI* + *b* as a function of *I* modulo *p*_3_, since we have restricted the **Type-1** adversary to choose *I* and $I_{k}^{*}$ so that $I\neq I_{k}^{*}$ modulo *p*_3_ and *a*, *b* modulo *p*_3_ only appear in these two values. Hence, O has produced a properly distributed EST-4^k^-CT and O has properly simulated the response of $O_{k}^{\prime \prime }$ in this case. So B is playing Game $S_{k,3}^{\prime }$. We have thus shown that O can use the output of B to achieve non-negligible advantage against Assumption 3.

Hence, if a PPT attacker can distinguish any pair between $S_{k,3}^{\prime }$ and $S_{k,1}^{\prime }$ with non-negligible advantage, O can distinguish the corresponding pair between ${\tilde{O}}_{k}^{\prime \prime }$ and ${\tilde{O}}_{k}^{*}$ with non-negligible advantage. It means O can use the output of B to achieve a non-negligible advantage against Assumption 3.

Thus, Under Assumptions 3, no PPT attacker can distinguish between ${\tilde{O}}_{k}^{\prime \prime }$ and ${\tilde{O}}_{k}^{*}$ with non-negligible advantage. Thus, no PPT attacker can distinguish between $S_{k,3}^{\prime }$ and $S_{k,1}^{\prime }$ with non-negligible advantage.

**Lemma 17** *Under Assumptions 4*, *no PPT attacker can distinguish between* ${\tilde{O}}_{k}^{\prime \prime }$ *and* ${\tilde{O}}_{k}^{\prime }$ *with non-negligible advantage*. *So no PPT attacker can distinguish between* $S_{k,3}^{\prime }$ *and* $S_{k,2}^{\prime }$ *with non-negligible advantage*.

***Proof*** We assume B interacts with one of ${\tilde{O}}_{k}^{\prime },{\tilde{O}}_{k}^{\prime \prime }$. O receives *g*, *g*_3_, *X*_1_ *X*_2_, *Y*_2_ *Y*_3_, *T*. O will simulate either ${\tilde{O}}_{k}^{\prime }$ or ${\tilde{O}}_{k}^{\prime \prime }$ with B, depending on the value of *T* (which is either in *G* or *G*_*p*_1_*p*_3__). O picks values *a*, *b*, *c*, *d*, *a*_0_, *b*_0_, *c*_0_, *d*_0_ ∈ *Z*_*N*_ uniformly at random and sets *u* = *g*^*a*^, *h* = *g*^*b*^, *v* = *g*^*c*^, *w* = *g*^*d*^, $u_{0}=g^{a_{0}}$, $h_{0}=g^{b_{0}}$, $v_{0}=g^{c_{0}}$, $w_{0}=g^{d_{0}}$. B initially obtains the group elements in Eq 85

$$\begin{array}{c} \begin{array}{c} g,u,v,w,X_{1}X_{2},w^{y}{\left(Y_{2}Y_{3}\right)}^{\psi },g^{y}\left(Y_{2}Y_{3}\right),v^{y}{\left(Y_{2}Y_{3}\right)}^{c},\\ u_{0},v_{0},w_{0},w_{0}^{y_{0}}{\left(Y_{2}Y_{3}\right)}^{z\psi },g^{y_{0}}{\left(Y_{2}Y_{3}\right)}^{z},v_{0}^{y_{0}}{\left(Y_{2}Y_{3}\right)}^{zc_{0}} \end{array} \end{array}$$

from its oracle simulator where *z*, *y*_0_, *y*, *ψ* ∈ *Z*_*p*_ are randomly chosen.

When A makes a secret key query for the identity *ID*|_*j*_ = (*I*_1_, ⋯, *I*_*j*_), then B makes its challenge HIBE-key-type query for *I*_*j*_, O chooses *r*, *y*′, *z*, *z*′ ∈ *Z*_*n*_ randomly and returns the group elements

$$\begin{array}{c} \left(w^{y^{\prime }}{\left(Y_{2}Y_{3}\right)}^{y^{\prime }\psi_{1}},g^{y^{\prime }}{\left(Y_{2}Y_{3}\right)}^{y^{\prime }},g^{r}{\left(Y_{2}Y_{3}\right)}^{z},v^{y^{\prime }}{\left(u^{I}h\right)}^{r}{\left(Y_{2}Y_{3}\right)}^{z^{\prime }}\right) \end{array}$$

to B. And then B creats the ESF-4 secret key by using the group elements.

Upon receiving a challenge IBE-key-type query for *T* ∈ *Z*_*n*_, O chooses *r*_0_, *y*_0_ ∈ *Z*_*n*_ randomly and returns the group elements

$$\begin{array}{c} \left(w_{0}^{y_{0}}{\left(Y_{2}Y_{3}\right)}^{y_{0}\psi_{2}},g^{y_{0}}{\left(Y_{2}Y_{3}\right)}^{y_{0}},g^{r_{0}},v_{0}^{y_{0}}{\left(Y_{2}Y_{3}\right)}^{y_{0}\sigma_{2}}{\left(u_{0}^{T}h_{0}\right)}^{r_{0}}\right) \end{array}$$

to B. And then B creats the semi-functional update key by using the group elements.

When A requests the challenge ciphertext for messages *M*_0_, *M*_1_, identity vector $\left(I_{1}^{*},\cdots ,I_{l}^{*}\right)$ and T*, B makes a ciphertext-type query to the oracle for each $I_{i}^{*}$ and T*. In response to each query for $I_{i}^{*}$ or T*, O gets random $t_{0}^{\prime },t_{1}^{\prime },\cdots ,t_{k}^{\prime },t_{k+1}\cdots ,t_{l}\in Z_{p}$, chooses *β* ∈ {0, 1} and creats the ciphertext as

$$\begin{array}{c} \left(C=M_{\beta }e{\left(X_{1}X_{2},g\right)}^{\alpha },C_{0}=X_{1}X_{2},{\left\{C_{i,1},C_{i,2},C_{i,3}\right\}}_{i=0}^{l}\right) \end{array}$$

where the ciphertext-element-group (*C*_*i*,1_, *C*_*i*,2_, *C*_*i*,3_) is defined as follows:

1. *i* < *k*: If *i* = 0, the ciphertext-element-group is
  $$\begin{array}{c} \left({\left(X_{1}X_{2}\right)}^{d_{0}}{\left(X_{1}X_{2}\right)}^{c_{0}t_{0}^{\prime }},{\left(X_{1}X_{2}\right)}^{t_{0}^{\prime }\left(a_{0}T^{*}+b_{0}\right)},{\left(X_{1}X_{2}\right)}^{t_{0}^{\prime }}\right) \end{array}$$
  else the ciphertext-element-group is
  $$\begin{array}{c} \left({\left(X_{1}X_{2}\right)}^{d}{\left(X_{1}X_{2}\right)}^{ct_{i}^{\prime }},{\left(X_{1}X_{2}\right)}^{t_{i}^{\prime }\left(aI_{i}^{*}+b\right)},{\left(X_{1}X_{2}\right)}^{t_{i}^{\prime }}\right) \end{array}$$
  This sets $X_{2}^{d}=g_{2}^{\delta_{1}}$ and $X_{2}^{d_{0}}=g_{2}^{\delta_{2}}$, which is uniformly random because the value of *d* and *d*_0_ modulo *p*_2_ will not appear elsewhere. It implicitly sets $g^{t_{i}}=X_{1}^{t_{i}^{\prime }}$. This is identically distributed to a response from *O*_2_, with *a*′, *b*′ equal to *a*, *b* modulo *p*_2_, and *σ*_1_ = *c* modulo *p*_2_, *σ*_2_ = *c*_0_ modulo *p*_2_. We note that this is in the only context in which the values of *a*, *b* modulo *p*_2_ appear, so this is equivalent to choosing *a*′, *b*′ independently at random.
2. *i* = *k*: If *k* = 0, the ciphertext-element-group is (*T*_1_, *T*_3_, *T*_2_) = $\left({\left(X_{1}X_{2}\right)}^{d_{0}}T^{c_{0}},T^{\left(a_{0}T^{*}+b_{0}\right)},T\right)$; If *k* > 0, the ciphertext-element-group is (*T*_1_, *T*_3_, *T*_2_) = $\left({\left(X_{1}X_{2}\right)}^{d}T^{c},T^{\left(aI_{i}^{*}+b\right)},T\right)$;
3. *i* > *k*: The ciphertext-element-group is $\left({\left(X_{1}X_{2}\right)}^{d}v^{t_{i}},{\left(u^{I_{i}^{*}}h\right)}^{t_{i}},g^{t_{i}}\right)$.

If *T* ∈ *G*_*p*_1_*p*_3__, then the response for ciphertext-type query *i* is identically distributed to a response from ${\tilde{O}}_{k}^{\prime }$.

In this case, O has produced a properly distributed EST-3^k^-CT and B is playing Game $S_{k,2}^{\prime }$.

If *T* ∈ *G*, then this response additionally has terms in *G*_*p*_2__ which are appropriately distributed with *c* = *σ*_1_, *a* = *a*′, *b* = *b*′ modulo *p*_2_ or *c*_0_ = *σ*_2_.*a*_0_ = *a*′, *b*_0_ = *b*′ modulo *p*_2_. Thus, the response is identically distributed to a response from ${\tilde{O}}_{k}^{\prime \prime }$. In this case, O has produced a properly distributed EST-4^k^-CT and B is playing Game $S_{k,3}^{\prime }$.

Hence, if a PPT attacker can distinguish any pair between $S_{k,2}^{\prime }$ and $S_{k,3}^{\prime }$ with non-negligible advantage, O can distinguish the corresponding pair between ${\tilde{O}}_{k}^{\prime }$ and ${\tilde{O}}_{k}^{\prime \prime }$ with non-negligible advantage. It means O can use the output of B to achieve a non-negligible advantage against Assumption 4.

Thus, Under Assumptions 4, no PPT attacker can distinguish between ${\tilde{O}}_{k}^{\prime }$ and ${\tilde{O}}_{k}^{\prime \prime }$ with non-negligible advantage. Thus, no PPT attacker can distinguish between $S_{k,2}^{\prime }$ and $S_{k,3}^{\prime }$ with non-negligible advantage.

**Lemma 18** *Under Assumptions 3*, *no PPT attacker can distinguish between* ${\tilde{O}}_{k}^{\prime }$ *and* ${\tilde{O}}_{k-1}^{*}$ *with non-negligible advantage*. *So no PPT attacker can distinguish between* $S_{k,2}^{\prime }$ *and* $S_{k-1,1}^{\prime }$ *with non-negligible advantage*.

***Proof*** We assume B interacts with one of ${\tilde{O}}_{k-1}^{*},{\tilde{O}}_{k}^{\prime }$. O receives *g*, *g*_2_, *X*_1_ *X*_3_, *Y*_1_ *Y*_3_, *T*. O will simulate either ${\tilde{O}}_{k-1}^{*}$ or ${\tilde{O}}_{k}^{\prime }$ with B, depending on the value of *T* (which is either in *G*_*p*_1__ or *G*_*p*_1_*p*_3__). B initially obtains the group elements in Eq 82

$$\begin{array}{c} \begin{array}{c} g,u,h,v,w,g^{s}g_{2}^{\gamma },{\left(X_{1}X_{3}\right)}^{d}g_{2}^{y_{2}d},\left(X_{1}X_{3}\right)g_{2}^{y_{2}},{\left(X_{1}X_{3}\right)}^{c}g_{2}^{cy_{2}}\\ u_{0},h_{0},v_{0},w_{0},{\left(X_{1}X_{3}\right)}^{zd_{0}}g_{2}^{y_{2}^{\prime }d_{0}},{\left(X_{1}X_{3}\right)}^{z}g_{2}^{y_{2}^{\prime }},{\left(X_{1}X_{3}\right)}^{zc_{0}}g_{2}^{c_{0}y_{2}^{\prime }} \end{array} \end{array}$$

from its oracle simulator. It chooses *α* ∈ *Z*_*n*_ randomly, and gives A the public parameters in Eq 83. B can responds by using the normal update key generation and the normal decryption key derivation algorithm, since it knows *α*.

When A requests the secret key of an identity vector *ID*|_*j*_ = (*I*_1_, ⋯, *I*_*j*_), B creats the ESF-4-SK key by the HIBE-type query response from O and the secret key for *ID*|_*j*_ in some node *θ* is

$$\begin{array}{c} \begin{array}{c} K_{i,0}=g^{\lambda_{i}}w^{y_{i}},K_{i,1}=g^{y_{i}},K_{i,2}=g^{r_{i}},K_{i,3}={\left(u^{I_{i}}h\right)}^{r_{i}}v^{y_{i}},i\in \left\{1,\cdots ,j-1\right\}\\ K_{j,0}=g^{\gamma_{\theta }-\sum_{i=1}^{j-1}\lambda_{i}}{\left(X_{1}X_{3}g_{2}\right)}^{dy_{j}^{\prime }},K_{j,1}={\left(X_{1}X_{3}g_{2}\right)}^{y_{j}^{\prime }},\\ K_{j,2}={\left(X_{1}X_{3}\right)}^{r_{j}^{\prime }}g_{2}^{z^{\prime }},K_{j,3}={\left(X_{1}X_{3}g_{2}\right)}^{cy_{j}^{\prime }}{\left(X_{1}X_{3}\right)}^{r_{j}^{\prime }\left(aI_{j}+b\right)}g_{2}^{z} \end{array} \end{array}$$

where $y_{1},\cdots ,y_{j-1},y_{j}^{\prime }$, $\lambda_{1},\cdots ,\lambda_{j-1},r_{1},\cdots ,r_{j-1},r_{j}^{\prime },z,z^{\prime }\in Z_{n}$ are randomly chosen.

When A requests the update key of an identity vector *ID*|_*j*_ = (*I*_1_, ⋯, *I*_*j*_) and the ime *T*, B creats the UK key by the IBE-type query response from O and the secret key for *ID*|_*j*_ in some node *θ* is generated as follows: O randomly chooses $y_{0}^{\prime },y_{1},\cdots ,y_{j-1}$, λ_1_, ⋯, λ_*j*−1_, *r*_0_, *r*_1_, ⋯, *r*_*j*−1_, *z*, *z*′ ∈*Z*_*n*_ and generates a semi-functional update key *TUK*_*ID*|_*l*_,*T*,*θ*_*h*__

$$\begin{array}{c} \begin{array}{c} U_{0,0}=g^{\alpha -\gamma_{\theta_{h}}-\sum_{i=1}^{j-1}\lambda_{i}}{\left(X_{1}X_{3}g_{2}\right)}^{d_{0}y_{0}^{\prime }},U_{0,1}={\left(X_{1}X_{3}g_{2}\right)}^{y_{0}^{\prime }},U_{0,2}=g^{r_{0}},\\ U_{0,3}={\left(X_{1}X_{3}g_{2}\right)}^{c_{0}y_{0}^{\prime }}{\left(u_{0}^{T}h_{0}\right)}^{r_{0}}\\ U_{i,0}=g^{\lambda_{i}}w^{y_{i}},U_{i,1}=g^{y_{i}},U_{i,2}=g^{r_{i}},U_{i,3}={\left(u^{I_{i}}h\right)}^{r_{i}}v^{y_{i}},i\in \left\{1,\cdots ,j-1\right\} \end{array} \end{array}$$

When A requests the challenge ciphertext for messages *M*_0_, *M*_1_, identity vector $\left(I_{1}^{*},\cdots ,I_{l}^{*}\right)$ and T*, B makes a ciphertext-type query to the oracle for each $I_{i}^{*}$ and T*. In response to each query for $I_{i}^{*}$ or T*, O gets random *t*_0_, *t*_1_, …, *t*_*l*_ ∈ *Z*_*p*_, chooses *β* ∈ {0, 1} and creats the ciphertext as

$$\begin{array}{c} \left(C=M_{\beta }e{\left(g^{s}g_{2}^{\gamma },g\right)}^{\alpha },C_{0}=g^{s}g_{2}^{\gamma },{\left\{C_{i,1},C_{i,2},C_{i,3}\right\}}_{i=0}^{l}\right) \end{array}$$

where the ciphertext-element-group (*C*_*i*,1_, *C*_*i*,2_, *C*_*i*,3_) is defined as follows:

1. *i* < *k*: If *i* = 0, O and responds with the ciphertext-element-group $\left(w_{0}^{s}g_{2}^{\delta_{2}}v_{0}^{t_{0}}g_{2}^{c_{0}t_{0}},{\left(u_{0}^{T^{*}}h_{0}\right)}^{t_{0}}g_{2}^{\left(a^{\prime }T^{*}+b^{\prime }\right)t_{0}},g^{t_{0}}g_{2}^{t_{0}}\right)$, else the element group is $\left(w^{s}g_{2}^{\delta_{1}}v^{t_{i}}g_{2}^{ct_{i}},{\left(u^{I_{i}^{*}}h\right)}^{t_{i}}g_{2}^{\left(a^{\prime }I_{i}^{*}+b^{\prime }\right)t_{i}},g^{t_{i}}g_{2}^{t_{i}}\right)$;
2. *i* = *k*: The ciphertext-element-group is (*T*_1_, *T*_3_, *T*_2_) = $\left(w^{s}g_{2}^{\delta_{1}}T^{c},T^{t_{i}\left(aI_{i}^{*}+b\right)},T\right)$;
3. *i* > *k*: The ciphertext-element-group is $\left(w^{s}g_{2}^{\delta_{1}}v^{t_{i}},{\left(u^{I_{i}^{*}}h\right)}^{t_{i}},g^{t_{i}}\right)$.

We must now argue that the challenge key-type query and the *k*^*th*^ ciphertext-type query responses are properly distributed. If *T* ∈ *G*_*p*_1__, then the response to the *k* ciphertext type query is identically distributed to a response from *O*_1_, and the values *a*, *b* modulo *p*_3_ only appear in the response to the challenge key-type query, hence the *G*_*p*_3__ parts on the last two group elements here appear random in *G*_*p*_3__. This will be a properly distributed EST-2^*k*−1^-CT which means that the responses of O properly simulate the responses of ${\tilde{O}}_{k-1}^{*}$ and B is playing Game ${}^{\prime }S_{k-1,1}$.

If *T* ∈ *G*_*p*_1_*p*_3__, then we must argue that *aI* + *b* and $aI_{k}^{*}+b$ both appear to be uniformly random modulo *p*_3_: this follows from pairwise independence of the function *aI* + *b* modulo *p*_3_, since we have restricted the **Type-1** adversary to choose *I* and $I_{k}^{*}$ so that $I\neq I_{k}^{*}$ modulo *p*_3_. This means that the *G*_*p*_3__ components on the last two group elements of the challenge key-type query response and on the *k* ciphertext-type query response are uniformly random in the attacker’s view. In this case, O has produced a properly distributed EST-3^k^-CT which means that O has properly simulated the responses of ${\tilde{O}}_{k}^{\prime }$ and B is playing Game $S_{k,2}^{\prime }$.

Hence, if a PPT attacker can distinguish any pair between $S_{k-1,1}^{\prime }$ and $S_{k,2}^{\prime }$ with non-negligible advantage, O can distinguish the corresponding pair between ${\tilde{O}}_{k-1}^{*}$ and ${\tilde{O}}_{k}^{\prime }$ with non-negligible advantage. It means O can use the output of B to achieve a non-negligible advantage against Assumption 3.

Thus, Under Assumptions 3, no PPT attacker can distinguish between ${\tilde{O}}_{k-1}^{*}$ and ${\tilde{O}}_{k}^{\prime }$ with non-negligible advantage. Thus, no PPT attacker can distinguish between $S_{k-1,1}^{\prime }$ and $S_{k,2}^{\prime }$ with non-negligible advantage.

**Lemma 19** *Under Assumptions 4*, *no PPT attacker can distinguish between* ${\tilde{O}}_{0}^{*}$ *and O*_7/2_ *with non-negligible advantage*. *So no PPT attacker can distinguish between I*_*h*_*c*_−1,2_ *and I*_*h*_*c*_,1_ *with non-negligible advantage*.

***Proof*** We assume B interacts with one of ${\tilde{O}}_{0}^{*},O_{7/2}$. O receives *g*, *g*_3_, *X*_1_ *X*_2_, *Y*_2_ *Y*_3_, *T*. O will simulate either ${\tilde{O}}_{0}^{*}$ or *O*_7/2_ with B, depending on the value of *T* (which is either in *G* or *G*_*p*_1_*p*_3__). O picks values *a*, *b*, *c*, *d*, *a*_0_, *b*_0_, *c*_0_, *d*_0_ ∈ *Z*_*N*_ uniformly at random and sets *u* = *g*^*a*^, *h* = *g*^*b*^, *v* = *g*^*c*^, *w* = *g*^*d*^, $u_{0}=g^{a_{0}}$, $h_{0}=g^{b_{0}}$, $v_{0}=g^{c_{0}}$, $w_{0}=g^{d_{0}}$. B initially obtains the group elements in Eq 89

$$\begin{array}{c} \begin{array}{c} g,u,h,v,w,X_{1}X_{2},w^{y}{\left(Y_{2}Y_{3}\right)}^{y\psi_{1}},g^{y}{\left(Y_{2}Y_{3}\right)}^{y},v^{y}{\left(Y_{2}Y_{3}\right)}^{y\sigma_{1}}\\ u_{0},h_{0},v_{0},w_{0},w_{0}^{y^{\prime }}{\left(Y_{2}Y_{3}\right)}^{y^{\prime }\psi_{2}},g^{y^{\prime }}{\left(Y_{2}Y_{3}\right)}^{y^{\prime }},v_{0}^{y^{\prime }}{\left(Y_{2}Y_{3}\right)}^{y^{\prime }\sigma_{2}} \end{array} \end{array}$$

from its oracle simulator who additionally chooses *ψ*_1_, *ψ*_2_, *σ*_1_, *σ*_2_, *y*, *y*′ ∈ *Z*_*N*_ randomly. These are properly distributed with *g*^*s*^ implicitly set to be *X*_1_.

When A requests the challenge ciphertext for messages *M*_0_, *M*_1_, identity vector $\left(I_{1}^{*},\cdots ,I_{l}^{*}\right)$ and T*, B makes a ciphertext-type query to the oracle for each $I_{i}^{*}$ and T*. When B makes a ciphertext-type query for some identity $I_{i}^{*}$, O responds by choosing a random $t_{i}^{\prime }\in Z_{N}$ and returning $\left({\left(X_{1}X_{2}\right)}^{d}v^{t_{i}},{\left(u^{I_{i}^{*}}h\right)}^{t_{i}},g^{t_{i}}\right)$ to B. When B makes a ciphertext-type query for some time T*, O responds by choosing a random *t*_0_ ∈ *Z*_*N*_ and returning $\left({\left(X_{1}X_{2}\right)}^{d_{0}}v_{0}^{t_{0}},{\left(u_{0}^{T^{*}}h\right)}^{t_{0}},g^{t_{0}}\right)$ to B. (Note that this implicitly sets $g_{2}^{\delta_{1}}=X_{2}^{d}$ and $g_{2}^{\delta_{2}}=X_{2}^{d_{0}}$, which is uniformly random because the value of *d* and *d*_0_ modulo *p*_2_ does not occur elsewhere.) Then B creats the semi-functional ciphertexts successfully.

Upon receiving a challenge IBE-key-type query for *T* ∈ *Z*_*n*_, O chooses *r*_0_, *y*_0_ ∈ *Z*_*n*_ randomly and returns the group elements

$$\begin{array}{c} \left(w_{0}^{y_{0}}{\left(Y_{2}Y_{3}\right)}^{y_{0}\psi_{2}},g^{y_{0}}{\left(Y_{2}Y_{3}\right)}^{y_{0}},g^{r_{0}},v_{0}^{y_{0}}{\left(Y_{2}Y_{3}\right)}^{y_{0}\sigma_{2}}{\left(u_{0}^{T}h_{0}\right)}^{r_{0}}\right) \end{array}$$

to B. And then B creats the semi-functional update key by using the group elements.

When B creats the HIBE private key with the index pair (*h*, *i*_*c*_) for the identity vector (*I*_1_, ⋯, *I*_*j*−1_) in the index *h* node, the secret key with an index pair (*h*, *i*_*c*_) is generated as follows:

1. *i*_*c*_ < *h*_*c*_: It randomly chooses *y*_1_, ⋯, *y*_*j*_, λ_1_, ⋯, λ_*j*−1_, *r*_1_, ⋯, *r*_*j*_, *z*, *z*′ ∈*Z*_*n*_ and generates a semi-functional secret key *TUK*_*ID*|_*l*_,*T*,*θ*_*h*__.
  $$\begin{array}{c} \begin{array}{c} K_{i,0}=g^{\lambda_{i}}w^{y_{i}},K_{i,1}=g^{y_{i}},K_{i,2}=g^{r_{i}},K_{i,3}={\left(u^{I_{i}}h\right)}^{r_{i}}v^{y_{i}},i\in \left\{1,\cdots ,j-1\right\}\\ K_{j,0}=g^{\gamma_{\theta_{h}}-\sum_{i=1}^{j-1}\lambda_{i}}w^{y_{j}}{\left(Y_{2}Y_{3}\right)}^{y_{j}\psi_{1}},K_{j,1}=g^{y_{j}}{\left(Y_{2}Y_{3}\right)}^{y_{j}},K_{j,2}=g^{r_{j}},\\ K_{j,3}=v^{y_{j}}{\left(Y_{2}Y_{3}\right)}^{y_{j}\sigma_{1}}{\left(u^{I_{j}}h\right)}^{r_{j}} \end{array} \end{array}$$
2. *i*_*c*_ = *h*_*c*_: B chooses random values *y*_1_, ⋯, *y*_*j*−1_, λ_1_, ⋯, λ_*j*−1_, *r*_1_, ⋯, *r*_*j*−1_ ∈ *Z*_*n*_. B forms the challenge key as:
  $$\begin{array}{c} \begin{array}{c} K_{i,0}=g^{\lambda_{i}}w^{y_{i}},K_{i,1}=g^{y_{i}},K_{i,2}=g^{r_{i}},K_{i,3}={\left(u^{I_{i}}h\right)}^{r_{i}}v^{y_{i}},i\in \left\{1,\cdots ,j-1\right\}\\ K_{j,0}=g^{\gamma_{\theta_{h}}-\sum_{i=1}^{j-1}\lambda_{i}}T_{0},K_{j,1}=T_{1},K_{j,2}=T_{3},K_{j,3}=T_{2} \end{array} \end{array}$$
  where (*T*_0_, *T*_1_, *T*_2_, *T*_3_) is the challenge HIBE key queried to O who chooses a random *y*_*j*_ ∈ *Z*_*N*_ and returns (*T*_0_, *T*_1_, *T*_2_, *T*_3_) = $\left(w^{y_{j}}{\left(Y_{2}Y_{3}\right)}^{\psi_{1}y_{j}},g^{y_{j}}{\left(Y_{2}Y_{3}\right)}^{y_{j}},v^{y_{j}}{\left(Y_{2}Y_{3}\right)}^{\sigma_{1}y_{j}}T^{aI_{j}+b},T\right)$ to B.
3. *i*_*c*_ > *h*_*c*_: It generates a ESF-4-SK as
  $$\begin{array}{c} \begin{array}{c} K_{i,0}=g^{\lambda_{i}}w^{y_{i}},K_{i,1}=g^{y_{i}},K_{i,2}=g^{r_{i}},K_{i,3}={\left(u^{I_{i}}h\right)}^{r_{i}}v^{y_{i}},i\in \left\{1,\cdots ,j-1\right\}\\ K_{j,0}=g^{\gamma_{\theta_{h}}-\sum_{i=1}^{j-1}\lambda_{i}}(w^{y_{j}}{\left(Y_{2}Y_{3}\right)}^{\psi_{1}y_{j}},K_{j,1}=g^{y_{j}}{\left(Y_{2}Y_{3}\right)}^{y_{j}},K_{j,2}=g^{r_{j}}{\left(Y_{2}Y_{3}\right)}^{z},\\ K_{j,3}=v^{y_{j}}{\left(u^{I_{j}}h\right)}^{r_{j}}{\left(Y_{2}Y_{3}\right)}^{z^{\prime }} \end{array} \end{array}$$
  where *z*, *z*′ ∈ *Z*_*p*_ are randomly chosen.

In the challenge HIBE key, it implicitly sets $g^{r_{j}}$ to be the *G*_*p*_1__ part of *T*. We note that *a*, *b* modulo *p*_2_,*p*_3_ are uniformly random and do not appear elsewhere. If *T* ∈ *G*_*p*_1_*p*_3__, then this matches the distribution of ${\tilde{O}}_{0}^{*}$, and so this will be a properly distributed ESF-4-SK key and B is playing Game *I*_*h*_*c*_−1,2_. If *T* ∈ *G*, then this matches the distribution of *O*_7/2_ (note random *G*_*p*_3__ terms attached to the last two group elements) and then B is playing Game *I*_*h*_*c*_,1_.

Hence, if a PPT attacker can distinguish between *I*_*h*_*c*_−1,2_ and *I*_*h*_*c*_,1_ with non-negligible advantage, O can distinguish between ${\tilde{O}}_{0}^{*}$ and *O*_7/2_ with non-negligible advantage. It means O can gain a non-negligible advantage against Assumption 4.

Thus, Under Assumptions 4, no PPT attacker can distinguish between ${\tilde{O}}_{0}^{*}$ and *O*_7/2_ with non-negligible advantage. Thus, no PPT attacker can distinguish between *I*_*h*_*c*_−1,2_ and *I*_*h*_*c*_,1_ with non-negligible advantage.

**Lemma 20** *Under Assumptions 3*, *no PPT attacker can distinguish between O*_7/2_ *and O*_4_ *with non-negligible advantage*. *So no PPT attacker can distinguish between I*_*h*_*c*_,1_ *and I*_*h*_*c*_,2_ *with non-negligible advantage*.

***Proof*** We assume B interacts with one of *O*_7/2_, and *O*_4_. O receives *g*, *g*_2_, *X*_1_ *X*_3_, *T*. O will simulate either *O*_7/2_ or *O*_4_ with B, depending on the value of *T* (which is either in *G*_*p*_1__ or *G*_*p*_1_*p*_3__). O picks values *a*, *b*, *c*, *d*, *a*_0_, *b*_0_, *c*_0_, *d*_0_ ∈ *Z*_*N*_ uniformly at random and sets *u* = *g*^*a*^, *h* = *g*^*b*^, *v* = *g*^*c*^, *w* = *g*^*d*^, $u_{0}=g^{a_{0}}$, $h_{0}=g^{b_{0}}$, $v_{0}=g^{c_{0}}$, $w_{0}=g^{d_{0}}$. B initially obtains the group elements in Eq 90

$$\begin{array}{c} \begin{array}{c} g,u,h,v,w,g^{s}g_{2}^{\gamma },{\left(X_{1}X_{3}\right)}^{d}g_{2}^{dy},\left(X_{1}X_{3}\right)g_{2}^{y},{\left(X_{1}X_{3}\right)}^{c}g_{2}^{y^{\prime }},\\ u_{0},h_{0},v_{0},w_{0},{\left(X_{1}X_{3}\right)}^{zd_{0}}g_{2}^{d_{0}y^{\prime }},{\left(X_{1}X_{3}\right)}^{z}g_{2}^{y^{\prime }},{\left(X_{1}X_{3}\right)}^{zc_{0}}g_{2}^{y^{\prime }} \end{array} \end{array}$$

from its oracle simulator, and gives A the public parameters in Eq 83.

When A requests the challenge ciphertext for messages *M*_0_, *M*_1_, identity vector $\left(I_{1}^{*},\cdots ,I_{l}^{*}\right)$ and T*, B makes a ciphertext-type query to the oracle for each $I_{i}^{*}$ and T*. When B makes a ciphertext-type query for some identity I*, O responds by choosing a random *t* ∈ *Z*_*N*_ and returning $\left(w^{s}g_{2}^{\delta_{1}}v^{t},g^{t},{\left(u^{I^{*}}h\right)}^{t}\right)$ to B. When B makes a ciphertext-type query for some time T*, O responds by choosing a random *t*_0_ ∈ *Z*_*N*_ and returning $\left(w_{0}^{s}g_{2}^{\delta_{2}}v_{0}^{t_{0}},g^{t_{0}},{\left(u_{0}^{T^{*}}h_{0}\right)}^{t_{0}}\right)$ to B. Then B creats the semi-functional ciphertexts successfully.

Upon receiving a challenge IBE-key-type query for *T* ∈ *Z*_*n*_, O chooses $r_{0},y_{0}^{\prime }\in Z_{n}$ randomly and returns the group elements

$$\begin{array}{c} {\left(X_{1}X_{3}g_{2}\right)}^{d_{0}y_{0}^{\prime }},{\left(X_{1}X_{3}g_{2}\right)}^{y_{0}^{\prime }},g^{r_{0}},{\left(X_{1}X_{3}g_{2}\right)}^{c_{0}y_{0}^{\prime }}{\left(u_{0}^{T}h_{0}\right)}^{r_{0}} \end{array}$$

to B. And then B creats the semi-functional update key by using the group elements.

When B creats the HIBE private key with the index pair (*h*, *i*_*c*_) for the identity vector (*I*_1_, ⋯, *I*_*j*−1_) in the index *h* node, the secret key with an index pair (*h*, *i*_*c*_) is generated as follows:

1. *i*_*c*_ < *h*_*c*_: It randomly chooses $y_{j}^{\prime },y_{1},\cdots ,y_{j-1}$, λ_1_, ⋯, λ_*j*−1_, *r*_1_, ⋯, *r*_*j*_, *z*, *z*′ ∈*Z*_*n*_ and generates a semi-functional secret key *PSK*_*ID*|_*j*_,*θ*_*h*__
  $$\begin{array}{c} \begin{array}{c} K_{i,0}=g^{\lambda_{i}}w^{y_{i}},K_{i,1}=g^{y_{i}},K_{i,2}=g^{r_{i}},K_{i,3}={\left(u^{I_{i}}h\right)}^{r_{i}}v^{y_{i}},i\in \left\{1,\cdots ,j-1\right\}\\ K_{j,0}=g^{\gamma_{\theta_{h}}-\sum_{i=1}^{j-1}\lambda_{i}}{\left(X_{1}X_{3}g_{2}\right)}^{dy_{j}^{\prime }},K_{j,1}={\left(X_{1}X_{3}g_{2}\right)}^{y_{j}^{\prime }},K_{j,2}=g^{r_{j}},\\ K_{j,3}={\left(X_{1}X_{3}g_{2}\right)}^{cy_{j}^{\prime }}{\left(u^{I_{j}}h\right)}^{r_{j}} \end{array} \end{array}$$
2. *i*_*c*_ = *h*_*c*_: B chooses random values *y*_1_, ⋯, *y*_*j*−1_, λ_1_, ⋯, λ_*j*−1_, *r*_1_, ⋯, *r*_*j*−1_ ∈ *Z*_*n*_. B forms the challenge key as:
  $$\begin{array}{c} \begin{array}{c} K_{i,0}=g^{\lambda_{i}}w^{y_{i}},K_{i,1}=g^{y_{i}},K_{i,2}=g^{r_{i}},K_{i,3}={\left(u^{I_{i}}h\right)}^{r_{i}}v^{y_{i}},i\in \left\{1,\cdots ,j-1\right\}\\ K_{j,0}=g^{\gamma_{\theta_{h}}-\sum_{i=1}^{j-1}\lambda_{i}}T_{0},K_{j,1}=T_{1},K_{j,2}=T_{3},K_{j,3}=T_{2} \end{array} \end{array}$$
  where (*T*_0_, *T*_1_, *T*_2_, *T*_3_) is the challenge HIBE key queried to O who chooses a random $y_{j}^{\prime }\in Z_{N}$ and returns (*T*_0_, *T*_1_, *T*_2_, *T*_3_) = $({\left(X_{1}X_{3}g_{2}\right)}^{dy_{j}^{\prime }},{\left(X_{1}X_{3}g_{2}\right)}^{y_{j}^{\prime }},$ ${\left(X_{1}X_{3}g_{2}\right)}^{cy_{j}^{\prime }}T^{aI_{j}+b},$ *T*). This implicitly sets $g^{r_{j}}$ to be the *G*_*p*_1__ part of *T*.
3. *i*_*c*_ > *h*_*c*_: It generates a ESF-4-SK as
  $$\begin{array}{c} \begin{array}{c} K_{i,0}=g^{\lambda_{i}}w^{y_{i}},K_{i,1}=g^{y_{i}},K_{i,2}=g^{r_{i}},K_{i,3}={\left(u^{I_{i}}h\right)}^{r_{i}}v^{y_{i}},i\in \left\{1,\cdots ,j-1\right\}\\ K_{j,0}=g^{\gamma_{\theta_{h}}-\sum_{i=1}^{j-1}\lambda_{i}}{\left(X_{1}X_{3}g_{2}\right)}^{dy_{j}^{\prime }},K_{j,1}={\left(X_{1}X_{3}g_{2}\right)}^{y_{j}^{\prime }},\\ K_{j,2}={\left(X_{1}X_{3}\right)}^{r_{j}^{\prime }}g_{2}^{z^{\prime }},K_{j,3}={\left(X_{1}X_{3}g_{2}\right)}^{cy_{j}^{\prime }}{\left(X_{1}X_{3}\right)}^{r_{j}^{\prime }\left(aI_{j}+b\right)}g_{2}^{z} \end{array} \end{array}$$
  where *z*, *z*′ ∈ *Z*_*p*_ are randomly chosen.

In the challenge HIBE key, it implicitly sets $g^{r_{j}}$ to be the *G*_*p*_1__ part of *T*. We note that *a*, *b* modulo *p*_2_,*p*_3_ are uniformly random and do not appear elsewhere. If *T* ∈ *G*_*p*_1_*p*_3__, then this matches the distribution of *O*_7/2_, and so this will be a properly distributed normal key and B is playing Game *I*_*h*_*c*_,1_. If *T* ∈ *G*_*p*_1__, then this matches the distribution of *O*_4_ and then B is playing Game *I*_*h*_*c*_,2_.

Hence, if a PPT attacker can distinguish between *I*_*h*_*c*_,1_ and *I*_*h*_*c*_,2_ with non-negligible advantage, O can distinguish between *O*_7/2_ and *O*_4_ with non-negligible advantage. It means O can gain a non-negligible advantage against Assumption 3.

Thus, Under Assumptions 3, no PPT attacker can distinguish between *O*_7/2_ and *O*_4_ with non-negligible advantage. Thus, no PPT attacker can distinguish between *I*_*h*_*c*_,1_ and *I*_*h*_*c*_,2_ with non-negligible advantage.

## References

[1] Seo JH, Emura K. Efficient Delegation of Key Generation and Revocation Functionalities in Identity-Based Encryption In: CT-RSA. vol. 7779 Springer; 2013 p. 343–358.

[2] Horwitz J, Lynn B. Toward hierarchical identity-based encryption In: Advances in Cryptology-EUROCRYPT 2002. Springer; 2002 p. 466–481.

[3] Seo JH, Emura K. Revocable hierarchical identity-based encryption: history-free update, security against insiders, and short ciphertexts. In: Cryptographers Track at the RSA Conference. Springer; 2015. p. 106–123.

[4] Tsai TT, Tseng YM, Wu TY. RHIBE: constructing revocable hierarchical ID-based encryption from HIBE. Informatica. 2014;25(2):299–326. doi: 10.15388/Informatica.2014.16

[5] Lee K. Revocable Hierarchical Identity-Based Encryption with Adaptive Security. IACR Cryptology ePrint Archive. 2016;2016:749.

[6] Seo JH, Emura K. Adaptive-ID secure revocable hierarchical identity-based encryption. In: International Workshop on Security. Springer; 2015. p. 21–38.

[7] Ryu G, Lee K, Park S, Lee DH. Unbounded hierarchical identity-based encryption with efficient revocation. In: International Workshop on Information Security Applications. Springer; 2015. p. 122–133.

[8] Rouselakis Y, Waters B. Practical constructions and new proof methods for large universe attribute-based encryption. In: Proceedings of the 2013 ACM SIGSAC conference on Computer & communications security. ACM; 2013. p. 463–474.

[9] Xing Q, Wang B, Wang X, Chen P, Yu B, Tang Y, et al. Unbounded Revocable Hierarchical Identity-Based Encryption with Adaptive-ID Security. In: High Performance Computing and Communications; IEEE 14th International Conference on Smart City; IEEE 2nd International Conference on Data Science and Systems (HPCC/SmartCity/DSS), 2016 IEEE 18th International Conference on. IEEE; 2016. p. 430–437.

[10] Waters B, et al Dual System Encryption: Realizing Fully Secure IBE and HIBE under Simple Assumptions In: Crypto. vol. 5677 Springer; 2009 p. 619–636.

[11] Lewko AB, Waters B. New Techniques for Dual System Encryption and Fully Secure HIBE with Short Ciphertexts In: TCC. vol. 5978 Springer; 2010 p. 455–479.

[12] Lewko AB, Waters B. Unbounded HIBE and Attribute-Based Encryption In: Eurocrypt. vol. 6632 Springer; 2011 p. 547–567.

[13] Lee K, Park S. Revocable Hierarchical Identity-Based Encryption with Shorter Private Keys and Update Keys. IACR Cryptology ePrint Archive. 2016;2016:460.

[14] Seo JH, Emura K. Revocable identity-based cryptosystem revisited: Security models and constructions. IEEE Transactions on Information Forensics and Security. 2014;9(7):1193–1205. doi: 10.1109/TIFS.2014.2327758

[15] Naor D, Naor M, Lotspiech J. Revocation and tracing schemes for stateless receivers In: Advances in Cryptology-CRYPTO 2001. Springer; 2001 p. 41–62.

[16] Boldyreva A, Goyal V, Kumar V. Identity-based encryption with efficient revocation. In: Proceedings of the 15th ACM conference on Computer and communications security. ACM; 2008. p. 417–426.

[17] Boldyreva A, Goyal V, Kumar V. Adaptive-ID Secure Revocable Identity-Based Encryption. In: Proceedings of the 15th ACM conference on Computer and communications security. ACM; 2008. p. 417–426.

[18] Boldyreva A, Goyal V, Kumar V. Constructions o f CCA-Secure Revo cable Identity-Based Encryption. In: Proceedings of the 15th ACM conference on Computer and communications security. ACM; 2008. p. 417–426.

[19] Boldyreva A, Goyal V, Kumar V. An Efficient and Provable Secure Revocable Identity-Based Encryption Scheme. In: Proceedings of the 15th ACM conference on Computer and communications security. ACM; 2008. p. 417–426.

[20] Lee K, Lee DH, Park JH. Efficient revocable identity-based encryption via subset difference methods. Designs, Codes and Cryptography. 2017;85(1):39–76. doi: 10.1007/s10623-016-0287-3

[21] Watanabe Y, Emura K, Seo JH. New revocable IBE in prime-order groups: Adaptively secure, decryption key exposure resistant, and with short public parameters. In: Cryptographers Track at the RSA Conference. Springer; 2017. p. 432–449.

[22] Lee K, Choi SG, Lee DH, Park JH, Yung M. Self-updatable encryption: Time constrained access control with hidden attributes and better efficiency. In: International Conference on the Theory and Application of Cryptology and Information Security. Springer; 2013. p. 235–254.

